# Controllable copper-catalysed photo-induced carbonylative cyclization to access dihydroquinolinones and oxindoles

**DOI:** 10.1039/d5sc09434h

**Published:** 2026-01-08

**Authors:** Yan-Hua Zhao, Le-Cheng Wang, Xiao-Feng Wu

**Affiliations:** a Leibniz-Institut für Katalyse e.V. Albert-Einstein-Straße 29a Rostock 18059 Germany Xiao-Feng.Wu@catalysis.de; b Dalian National Laboratory for Clean Energy, Dalian Institute of Chemical Physics, Chinese Academy of Sciences Dalian 116023 Liaoning China xwu2020@dicp.ac.cn

## Abstract

Oxindoles and dihydroquinolinones are pivotal heterocyclic scaffolds in medicinal and synthetic chemistry. Herein, we describe a controllable visible-light-induced, copper-catalyzed carbonylative cyclization of arylthianthrenium salts with alkenes, enabling the efficient synthesis of structurally diverse oxindoles and dihydroquinolinones. Notably, this transformation proceeds under mild conditions without the need for expensive photocatalysts, and regioselective acyl radical addition is achieved simply by tuning the substitution pattern of the alkene, which enables the switchable synthesis of carbonylated five- and six-membered heterocycles. Mechanistic studies indicate that blue-light irradiation promotes the copper-mediated reduction of arylthianthrenium salts, generating aryl radicals that subsequently capture CO to afford acyl radicals and initiate a tandem cyclization sequence. This method exhibits broad functional-group tolerance and offers a versatile platform for the late-stage functionalization of bioactive molecules.

## Abstract 2

A controllable visible-light-induced, copper-catalyzed carbonylative cyclization of arylthianthrenium salts with alkenes, enabling the efficient synthesis of structurally diverse oxindoles and dihydroquinolinones has been developed.

## Graphical abstract

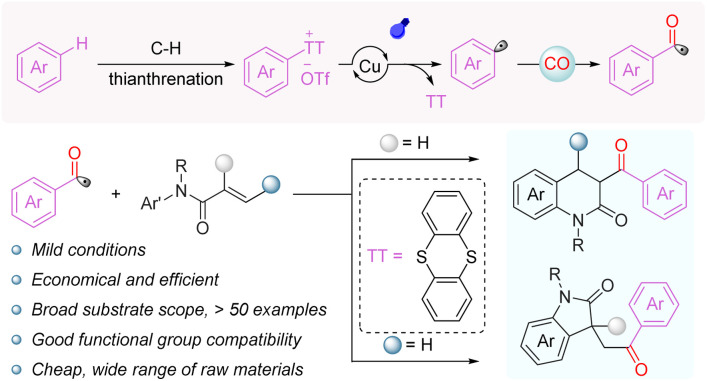

## Introduction

**Scheme 1:**
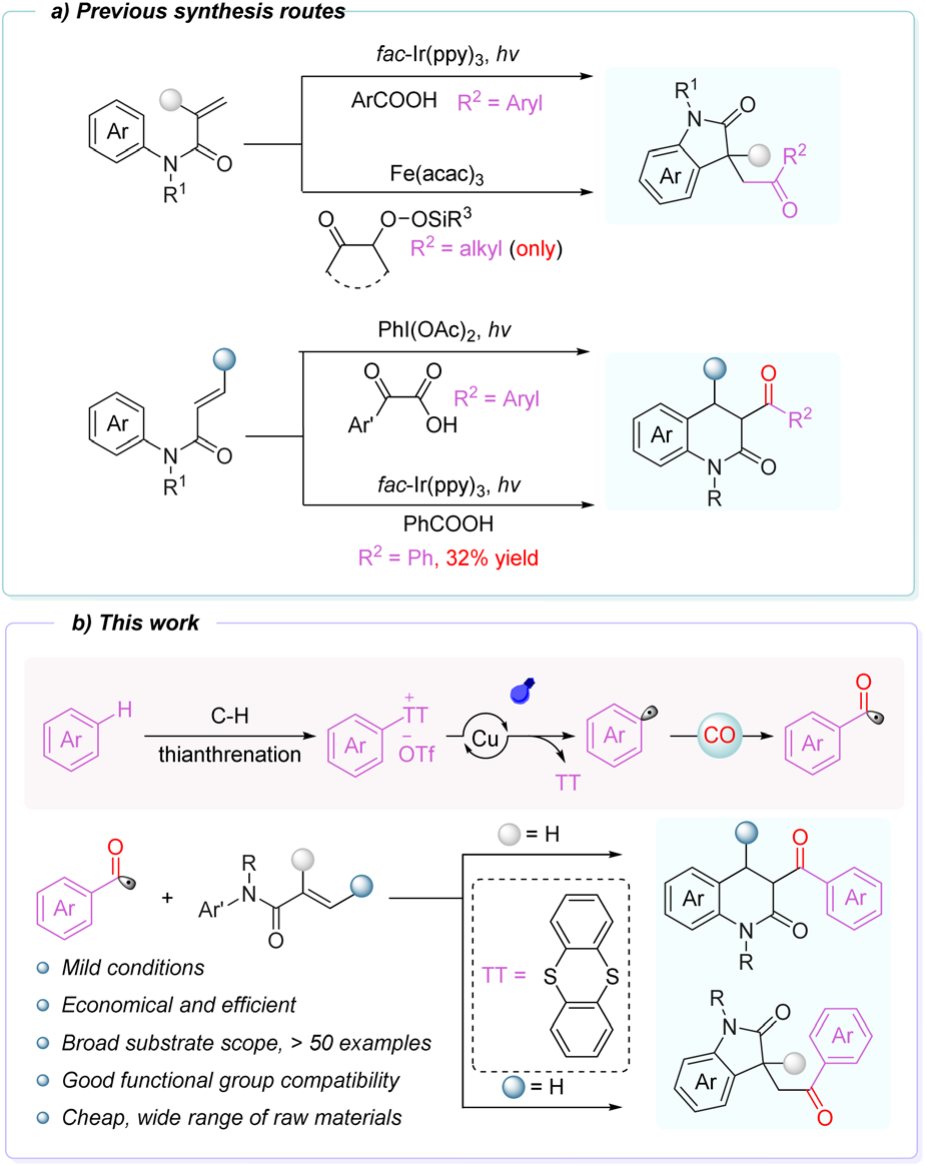
Previous synthesis routes and this work.

3,3-Disubstituted oxindoles and dihydroquinolinones are important nitrogen-containing heterocycles that are widely found in natural products and bioactive molecules,^1–8^ and their efficient synthesis continues to attract significant attention.^9–15^ Over the past decades, numerous transition-metal-catalyzed and metal-free oxidative strategies have been developed for assembling these frameworks.^16–20^ Among them, the palladium-catalyzed domino-Heck reaction is typically one of the most efficient methods for constructing heterocycles. This approach employs (pseudo)halides, chloroform, aldehydes, and other substrates, coupling them with alkenes to form oxygen-containing indoles and dihydroquinolones through ring-closure reactions.^21–24^ In recent years, significant progress has been made in radical-based cyclizations (Scheme 1a). Wallentin, Banerjee, and others developed visible-light-driven strategies for generating acyl radicals that subsequently undergo transformations with *N*-aryl acrylamides.^25,26^ In 2023, the Maruoka group reported an iron-catalyzed method in which alkyl silyl peroxides serve as precursors to acyl radicals, enabling cascade reactions with *N*-aryl acrylamides.^27^ Shortly thereafter, Liu and co-workers reported a visible-light-mediated radical cascade between *N-*aryl acrylamides or *N-*methylacrylamide benzamides and carbon dioxide.^28^ However, these approaches still suffer from limitations such as limited substrate scope. Therefore, the development of a direct, efficient, sustainable, and controllable strategy for the construction of carbonyl-containing heterocyclic frameworks remains highly desirable.

Carbonylation chemistry offers one of the most direct and versatile platforms for accessing carbonyl-containing molecules.^29–35^ Since Heck's pioneering report of palladium-catalyzed carbonylation in 1974, carbon monoxide (CO) has become an essential C1 synthon for transforming simple and readily available substrates into diverse carbonylated derivatives.^36–42^ Despite its broad synthetic utility, achieving chemoselective and regioselective carbonylation remains challenging, as conventional protocols often rely on noble-metal catalysts, strong Brønsted acids, and high temperatures, thereby limiting functional-group tolerance and substrate scope.^43–49^*N*-arylacrylamides, as structurally privileged activated alkenes, offer an attractive platform for achieving regioselective carbonylation *via* substitution-controlled reactivity, enabling streamlined access to carbonylated oxindoles and dihydroquinolinones.^50–56^ Meanwhile, arylthianthrenium salts have attracted considerable interest due to their high reactivity and ready accessibility, which allows them to be readily prepared from simple arenes *via* C(sp^2^)–H thianthrenation.^57–64^ In recent years, Ritter and co-workers have systematically investigated thianthrenium salts, revealing their broad utility in diverse C–H functionalization reactions.^65–70^ However, only a few examples of carbonylation reactions using thianthrenium salts have been reported, and there are no related reports on the selective controllable carbonylation associated with them.

Building on our previous investigations into the photochemical behavior of thianthrenium salts,^68,71–75^ we demonstrated that these reagents undergo efficient visible-light-induced homolytic C(sp^2^)-S bond cleavage to generate aryl radicals. Leveraging this reactivity, we developed a controllable single electron carbonylative cyclization manifold. Under visible-light-promoted copper catalysis, the *in situ* formed aryl radicals readily capture CO to produce acyl radicals, which undergo selective addition with *N*-aryl acrylamides to deliver oxindoles and dihydroquinolinones. *N*-aryl acrylamides are readily accessible from a variety of α,β-unsaturated carboxylic acid derivatives, and their tunable substitution patterns provide an ideal platform for controlling reaction selectivity. Remarkably, tuning the alkene substitution pattern alone dictates the regioselectivity of acyl-radical addition, granting switchable access to carbonylated five- and six-membered heterocycles without altering reaction conditions. This platform thus provides a general, mild, and highly controllable approach to carbonylation, addressing key limitations in current radical carbonylation chemistry (Scheme 1b).

## Results and discussion

We began our investigation using *N*-arylacrylamide 1a and arylthianthrenium salt 2a as the model substrates to evaluate the feasibility of the carbonylative cyclization (Table 1). Under blue-light irradiation at 60 °C in EtOAc, with CuCl as the catalyst, (*S*)-DM-BINAP as the ligand, and triisobutylamine as the base under 40 bar CO for 24 h, the desired oxindole 3aa was obtained in 80% yield (78% isolated, entry 1). Control experiments confirmed that the reaction did not proceed in the absence of the copper catalyst, ligand, base, or light (Table 1, entries 3–5). Screening of copper salts revealed that Cu(acac)_2_ could also promote the reaction, even with a slightly lower yield, while other copper catalysts gave comparable results (Table 1, entries 6–8). Bidentate phosphine ligands facilitated product formation but did not improve the yield, and the reaction outcomes for (*R*)-DM-BINAP and (*S*)-DM-BINAP were similar (Table 1, entries 9 and 12). In contrast, nitrogen-based or monodentate phosphine ligands failed to produce detectable amounts of 3aa (Table 1, entries 10 and 11). The choice of base significantly affected the reaction outcome, with organic bases outperforming inorganic bases (Table 1, entries 13 and 14). Solvent screening indicated that EtOAc was the optimal, while acetonitrile or toluene led to decreased yields (Table 1, entries 15 and 16). Reducing the amount of 2aa or increasing the reaction concentration also lowered the yield (Table 1, entries 17 and 18). The reaction proceeded at 40 °C to give 3aa in 73% yield (Table 1, entry 19), and lowering the CO pressure similarly decreased the reaction efficiency (Table 1, entry 20). For the reaction time, the conversion cannot be completed after 15 hours and no better yield can be produced if we extend the reaction time to 36 hours.

**Table 1:** Optimization of reaction conditions^a^

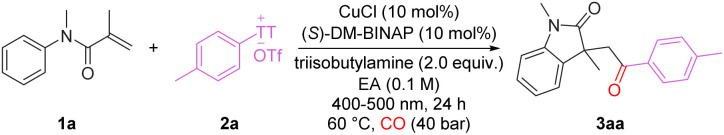		
Entry	Variation from the standard conditions	Yield (%)^b^
1	None	80 (78)^c^^,^^d^
2	No catalyst	n.d.
3	No ligand	n.d.
4	No base	Trace
5	No light irradiation	n.d.
6	CuBr instead of CuCl	36
7	Cu(CH_3_CN)_4_BF_4_ instead of CuCl	56
8	Cu(acac)_2_ instead of CuCl	31
9	BINAP instead of (*S*)-DM-BINAP	69
10	PCy_3_ instead of (*S*)-DM-BINAP	n.d.
11	1,10-Phen instead of (*S*)-DM-BINAP	n.d.
12	(*R*)-DM-BINAP instead of (*S*)-DM-BINAP	79
13	DIPEA instead of triisobutylamine	74
14	K_3_PO_4_ instead of triisobutylamine	16
15	CH_3_CN as solvent	25
16	Toluene as solvent	56
17	1.0 equiv. 2a	50
18	0.2 mol L^−1^ EA	75
19	40 °C instead of 60 °C	73
20	20 bar CO	69

a Reaction conditions: 1a (0.1 mmol), 2a (0.15 mmol), CO (40 bar), catalyst (10 mol%), ligand (10 mol%), base (0.2 mmol), solvent (1.0 mL), blue-light irradiation at 60 °C, 24 h.

b Yield was determined by GC using ^*n*^Hexadecane as the internal standard.

c Isolated yield.

d 70% yield on 10 mmol scale.

**Scheme 2:**
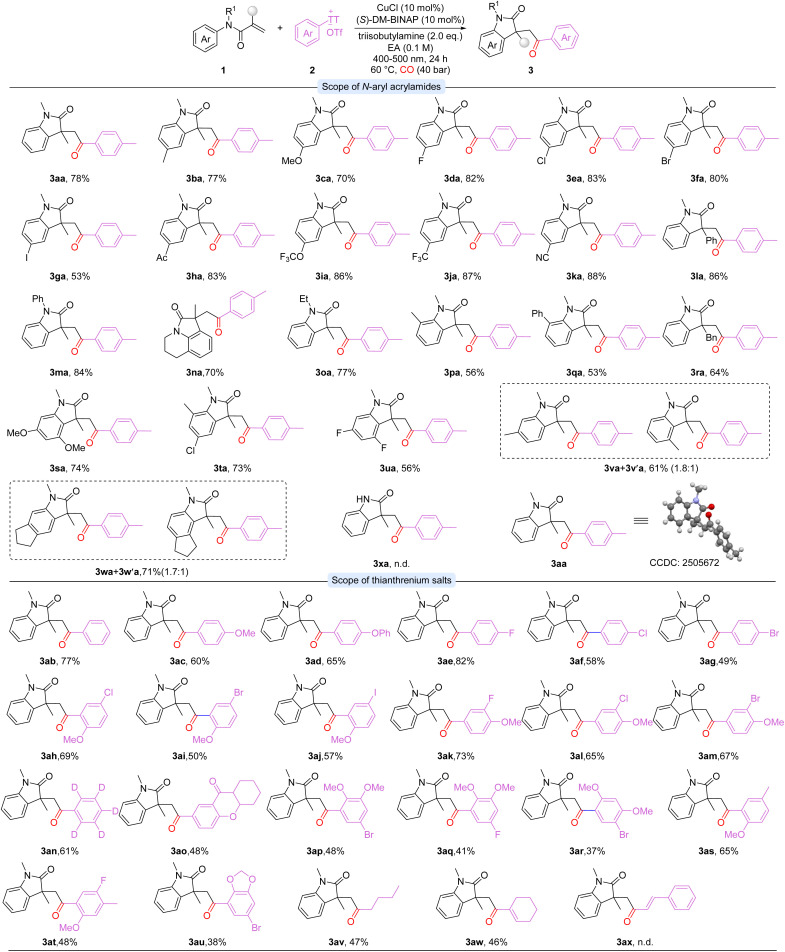
Reaction conditions: 1 (0.1 mmol), 2 (0.15 mmol), CuCl (10 mol%), (*S*)-DM-BINAP (10 mol%), triisobutylamine (0.2 mmol), EA (1.0 mL), CO (40 bar), under blue-light irradiation at 60 °C, 24 h.; isolated yields.

With the optimal conditions established, the substrate scope of *N*-aryl acrylamides was investigated at the first stage (Scheme 2). Unsubstituted and electron-donating substrates, such as methyl- and methoxy-substituted *N*-aryl acrylamides, were well tolerated, affording the desired products in good to high yields (3aa-3ca). Halogen-substituted substrates (F, Cl, Br, I) were also compatible, providing the corresponding products in moderate to excellent yields (3da-3ga). Notably, substrates bearing electron-withdrawing groups, including acyl, trifluoromethoxy, trifluoromethyl, and cyano, proceeded smoothly to give oxindoles in excellent yields (3ha-3ka). Alkenes bearing other substituents delivered products 3la and 3ra in 86% and 64% yields, respectively. *N*-protected acrylamides with phenyl or ethyl groups were also compatible, yielding 3ma and 3oa in 84% and 77% yields. In contrast, unprotected *N*-aryl acrylamides failed to afford the desired product (3xa). The ester analogue was also tested, but no lactone product could be detected. Tetrahydroquinoline derivatives were tolerated, providing the target product 3na in 70% yield. *Ortho*-Substituted substrates furnished the products 3pa and 3qa in moderate yields, while *meta*-substituted substrates led to regioisomeric mixtures (3va/3v′a and 3wa/3w′a). Furthermore, multi-substituted substrates were also well tolerated, afforded the corresponding products in excellent yields (3sa-3ua).

The substrate scope of thianthrenium salts was further investigated (Scheme 2). Unsubstituted and electron-rich aryl thianthrenium salts gave the desired products in good yields (3ab-3ad, 3as). Aryl thianthrenium salts bearing C(sp^2^)-X bonds (*X* = F, Cl, Br) were also compatible, providing the corresponding products in moderate to excellent yields (3ae-3ag). Similarly, di- and tri-substituted aryl thianthrenium salts (3ah-3am and 3ap-3ar, 3at-3au) performed well under the reaction conditions, delivering the target products in good yields in general. These results highlight the high chemoselectivity of the catalytic system and its potential for subsequent structural modifications. Complex scaffolds, including flavone-derived salts, also participated smoothly, delivering the corresponding product in moderate yield (3ao). Deuterated substrates afforded the desired oxindole 3an in 61% yield. Notably, thianthrenium salts bearing alkyl and alkenyl substituents reacted smoothly under the standard conditions, delivering the desired products (3av-3aw) in moderate yields. However, the styrenyl-substituted thianthrenium salt showed no reactivity under the standard reaction conditions (3ax). These results indicate that the catalytic system features broad substrate scope and high chemoselectivity. Additionally, instead of thianthrenium salt, iodobenzene was checked as well but lead to no desired product formed.

**Scheme 3:**
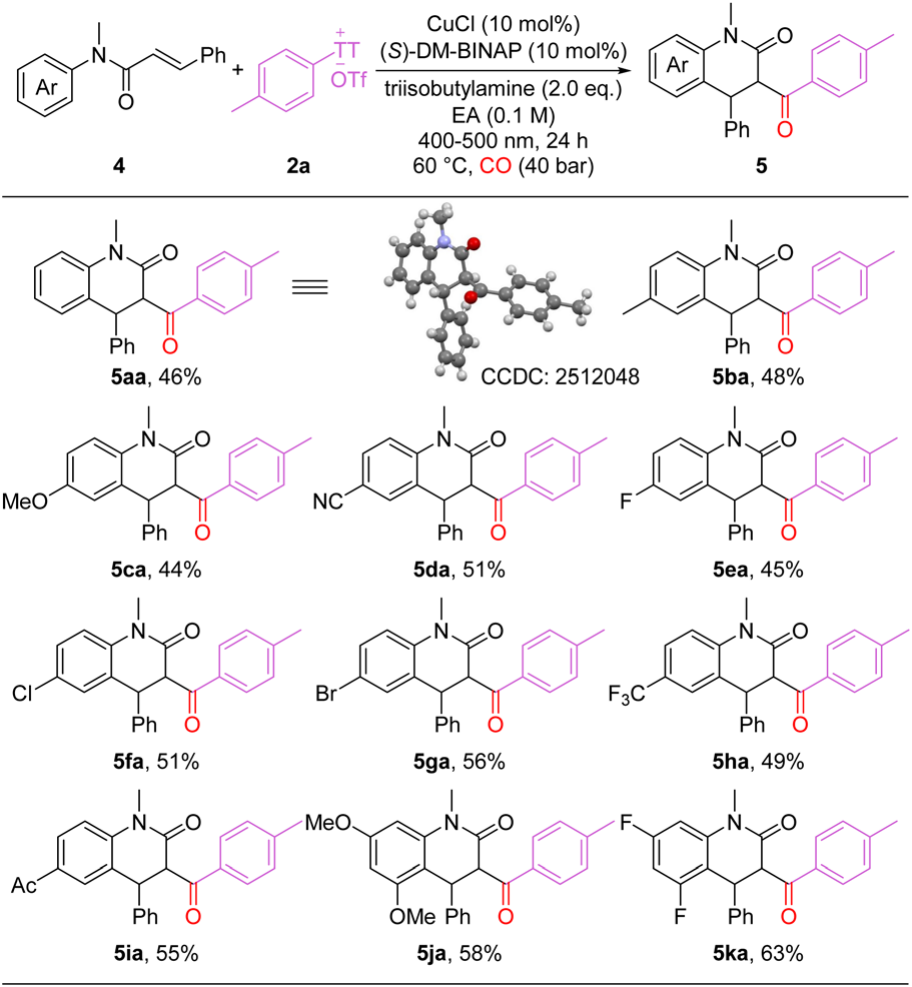
Reaction conditions: 4 (0.1 mmol), 2a (0.15 mmol), CuCl (10 mol%), (*S*)-DM-BINAP (10 mol%), triisobutylamine (0.2 mmol), EA (1.0 mL), CO (40 bar), under blue-light irradiation at 60 °C, 24 h.; isolated yields.

Under the standard reaction conditions, employing alkenes with different substitution patterns, six-membered dihydroquinolinones were selectively obtained rather than five-membered oxindoles (Scheme 3). As shown in the scheme, a variety of carbonylated six-membered heterocycles bearing diverse functional groups were synthesized in moderate to good yields (5aa-5ka). Unsubstituted and electron-rich substrates afforded the corresponding products in moderate yields (5aa-5ca). Internal alkenes bearing halogens (F, Cl, Br) or electron-withdrawing groups (cyano, acyl, trifluoromethyl) were compatible with the reaction, delivering the desired products in moderate yields (5da-5ia). Multi-substituted substrates also performed well, providing the target products in good yields (5ja-5ka). It's worth to mention that non-carbonylation product was the main by-product during this process and response for the moderate yields.

**Scheme 4:**
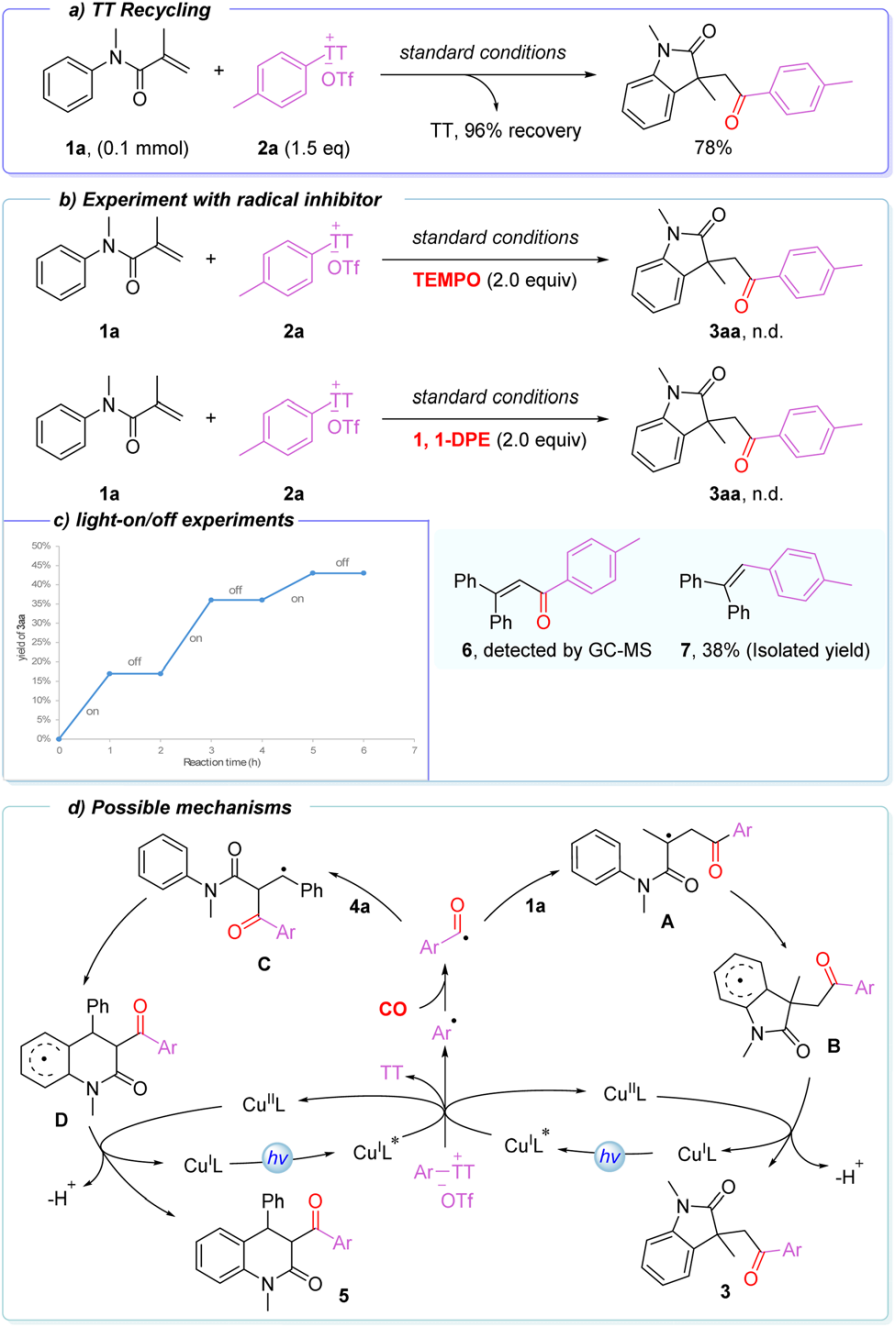
Control experiments and possible mechanisms.

The atom economy of the reaction was also evaluated, and thianthrene was recovered in yields of up to 96% (Scheme 4a). To gain some insight into the reaction mechanism, several mechanistic experiments were conducted. Under the standard conditions, the addition of 2.0 equiv. of 2,2,6,6-tetramethylpiperidine-1-oxyl (TEMPO) completely suppressed the formation of the desired product. Similarly, the addition of 2.0 equiv. of 1,1-diphenylethylene (1,1-DPE) led to no detectable target product, while radical adducts 6 and 7 were observed (Scheme 4b). Furthermore, light-on/off experiments revealed that the reaction proceeds only under visible-light irradiation, and no conversion occurs in the dark (Scheme 4c). Collectively, these results indicate that the transformation does not follow a radical chain process, but rather proceeds *via* an aryl radical intermediate.

Based on the above results and literature precedents, a plausible mechanism is proposed (Scheme 4d).^76–80^ Under blue-light irradiation, the Cu^I^L complex is excited to Cu^I^L*, which reacts with the aryl thianthrenium salt to generate an aryl radical and copper(ii) complex Cu^II^L. The aryl radical captures CO to form an acyl radical, which adds to the alkene of *N*-methyl-*N*-phenylacrylamide 1a, affording intermediate A. Intramolecular cyclization on the aryl ring then gives intermediate B, which undergoes single-electron transfer with Cu^II^L and subsequent deprotonation to yield oxindole 3. Similarly, when the acyl radical reacts with *N*-methyl-*N*-phenylcinnamamide, addition to the alkene forms intermediate C, followed by intramolecular cyclization to intermediate D. Subsequent single-electron transfer with Cu^II^L and deprotonation furnishes the six-membered dihydroquinolinone 5. This mechanism highlights the key roles of aryl radicals, CO trapping, and Cu-mediated single-electron transfer in enabling selective carbonylative cyclization.

## Conclusions

In summary, we have developed a visible-light-induced, copper-catalyzed controllable carbonylative cyclization that enables selective synthesis of carbonylated five- and six-membered heterocycles by simply tuning the substitution pattern of the starting alkenes. The method employs inexpensive CO as the carbonyl source, proceeds under mild conditions, and allows efficient one-pot access to 3,3-disubstituted oxindoles and dihydroquinolinones. It features broad substrate scope, generally good yields, excellent functional-group tolerance, and potential for downstream derivatization, providing a practical, economical, and sustainable approach to nitrogen-containing carbonylated heterocycles.

## Author contributions

Y. H. Z. and L. C. W. designed and carried out the reactions and analyzed the data. X.-F. W. designed and supervised the project. X.-F. W. and Y. H. Z. wrote and revised the manuscript.

## Conflicts of interest

There are no conflicts to declare.

## Supplementary Material

SC-OLF-D5SC09434H-s001

## Data Availability

The data supporting this article have been included as part of the supplementary information (SI). Supplementary information: general comments, general procedure, analytic data, and NMR spectra. See DOI: https://doi.org/10.1039/d5sc09434h.
