# Supplementary material for: Controllable copper-catalysed photo-induced carbonylative cyclization to access dihydroquinolinones and oxindoles
Source: Chem Sci. 2026 Jan 8;17(9):4632–9. doi: 10.1039/d5sc09434h (PMC12794647; doi:10.1039/d5sc09434h)

Supplementary Information (SI) for Chemical Science.
This journal is © The Royal Society of Chemistry 2026

Supporting Information

Controllable Copper-Catalysed Photo-Induced Carbonylative Cyclization to Access Dihydroquinolinones and Oxindoles

Yan-Hua Zhao, [a] Le-Cheng Wang, [a,b] and Xiao-Feng Wu* [a,b]

a. Leibniz-Institut für Katalyse e.V., Albert-Einstein-Straße 29a, 18059 Rostock, Germany, E-mail: Xiao-Feng.Wu@catalysis.de

b. Dalian National Laboratory for Clean Energy, Dalian Institute of Chemical Physics, Chinese Academy of Sciences, 116023 Dalian, Liaoning, China, E-mail: xwu2020@dicp.ac.cn

Contents

1. General Experimental 1
2 General Procedure 1
  2.1 General Procedure for the Synthesis of *N*-arylacrylamides 1
  2.2 General Procedure for the Synthesis of *N*-arylacrylamides 2
  2.3 General Procedure for the Carbonylation 2
3. Optimization of Reaction Conditions[a] 3
  3.1 The Effect of Catalyst on the Reaction 3
  3.2 The Effect of Ligand on the Reaction 3
  3.3 The Effect of Base on the Reaction 4
  3.4 The Effect of Solvent on the Reaction 4
  3.5 The Effect of other factors on the reaction 5
  3.6 Control experiment 5
4. Characterization and Procedure of the Products 6
5. Control Experiments 27
  5.1 Radical Inhibition Experiment 27
  5.2 Light on/off experiment 31
6. X-ray Crystal Analysis of 3aa and 5aa 32
7. Reference 34
8. NMR Spectra of Products 35

1. General Experimental

Reagents and solvents: Unless otherwise noted, the chemicals were commercially available from Sigma-Aldrich, TCI or Alfa Aesar and were used without further purification. Dioxane bought from Alfa Aesar, HPLC grade, 99% min, packaged under argon in resealable ChemSeal bottles. The reaction does not require the glovebox.

Purification: The products were isolated from the reaction mixture by column chromatography on silica gel 60, 0.063-0.2 mm, 70-230 mesh (Merck). Gradient flash chromatography was conducted eluting with PE/EA, PE refers to pentane and EA refers to ethyl acetate, they were listed as volume/volume ratios.

Data collection: GC-yields were calculated using hexadecane as internal standard. GC analysis was performed on an Agilent HP-7890A instrument with FID detector and HP-5 capillary column (polydimethylsiloxane with 5% phenyl groups, 30 m, 0.32 mm i.d., 0.25 µm film thickness) using argon as carrier gas. High resolution mass spectra (HRMS) were recorded on Agilent 6210. NMR spectra were recorded on Bruker Avance 300 and Bruker ARX 400 spectrometers. Chemical shifts (ppm) are given relative to solvent: references for $CDCl_3$ were 7.26 ppm (^1H NMR) and 77.00 ppm (^{13}C NMR). All measurements were carried out at room temperature unless otherwise stated.

2 General Procedure

2.1 General Procedure for the Synthesis of *N*-arylacrylamides

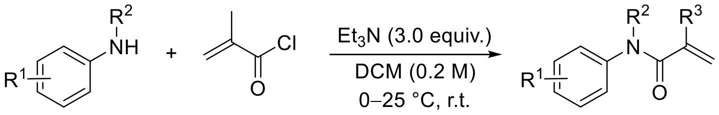

General procedure A: Synthesis of acrylamides via methacryloyl chloride.[1] To a mixture of the aniline derivative (1.0 equiv.), triethylamine (3.0 equiv.) in DCM (0.2 M) at 0 °C under Ar, was added methacryloyl chloride (1.2 equiv.) dropwise and the reaction mixture was stirred at room temperature for 12 h. Then the mixture was diluted with DCM, washed with sat. aqueous NH_4Cl, sat. aqueous NaCl, dried over anhydrous Na_2SO_4 and concentrated to dryness under reduced pressure. The crude product was purified by column chromatography on silica gel (petroleum ether:EtOAc gradient) to give acrylamide.

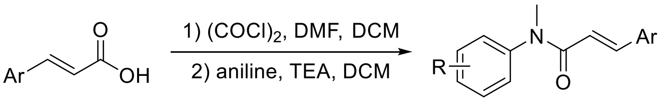

General procedure B: To a mixture of the acid (1.0 equiv) in DCM (0.3 M) was added a catalytic amount of DMF (0.1 mL/mmol acid). At ambient temperature, oxalylchloride (1.5 equiv) was added dropwise over a period of 0.5 h, forming a homogenous solution. The resulting solution was kept at room temperature for 3 h. Then, the solvent was removed under reduced pressure.

To a suspension of the aniline derivative (1.0 equiv.), triethylamine (3.0 equiv.) in DCM was the residue at 0 °C under Ar and the reaction mixture allowed to warm slowly to room temperature. The reaction mixture was stirred at room temperature for 12 h, washed with aqueous HCl (1.0 M), followed by sat. aqueous Na_2CO_3. The organic solvent was dried over anhydrous Na_2SO_4 and concentrated to dryness under reduced pressure. The crude product was purified by column chromatography on silica gel (petroleum ether:EtOAc gradient) to give acrylamide.[2]

2.2 General Procedure for the Synthesis of *N*-arylacrylamides

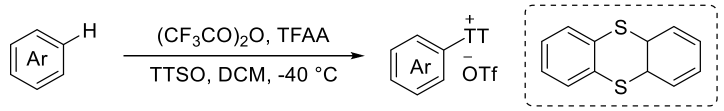

A 25 mL schlenk tube was charged with thianthrene S-oxide (1.0 equiv), DCM (0.25 M) and arenes (1.0 equiv.) under a nitrogen atmosphere. The reaction mixture was then cooled to -40 °C. Then trifluoroacetic anhydride (TFAA, 3.0 equiv.) and trifluoromethanesulfonic acid (TfOH, 1.5 equiv) were added dropwise. The reaction mixture was stirred at -40 °C for 30 min and then allowed to stir at room temperature for 12 h, neutralized by a saturated aqueous $NaHCO_3$ solution, and extracted with DCM. The combined organic layers were washed with aqueous NaOTf solution (3 × 20 mL, 5% (w/w)), dried over anhydrous Na_2SO_4, and concentrated to dryness under reduced pressure. The crude product was purified by crystallization from DCM/Et_2O system to afford aryl thianthrenium salts.[3, 4]

2.3 General Procedure for the Carbonylation

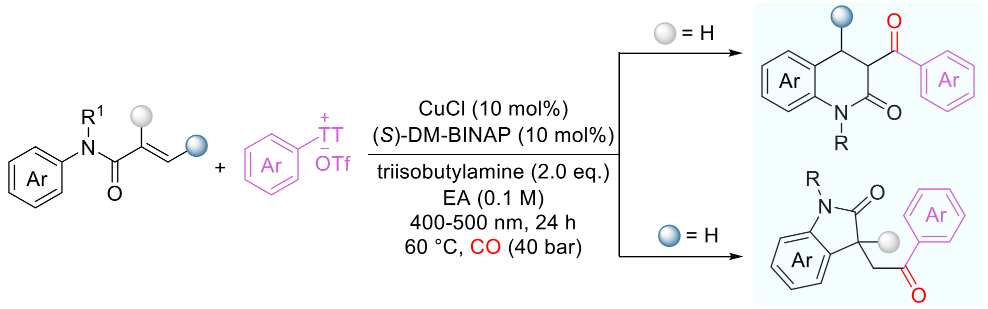

A 4 mL snap vial was charged with CuCl (10 mol%), (*S*)-DM-BINAP (10 mol%), thianthrenium salts (1.5 equiv.), *N*-acrylamides (1.0 equiv.) and closed with a rubber-based septum. The vial was evacuated and backfilled with argon. Degassed EA (0.1 M) and triisobutylamine (2.0 equiv.) were added via syringe. The vial was then connected to atmosphere with a cannula and transferred into a 300 mL Parr 4560 series autoclave, under argon counterflow. The closed autoclave was flushed three times with nitrogen (~ 5 bar), three times with CO (~ 5 bar), and 40 bar of carbon monoxide (measured by pressure meter) was charged. The autoclave was then placed into an aluminum block on a magnetic stirrer. The reaction mixture was stirred (500 rpm) under blue-light irradiation at 60 °C for 24 h. The crude product was purified by silica gel chromatography (pentane/EA) to afford the corresponding product.

3. Optimization of Reaction Conditions[a]

3.1 The Effect of Catalyst on the Reaction

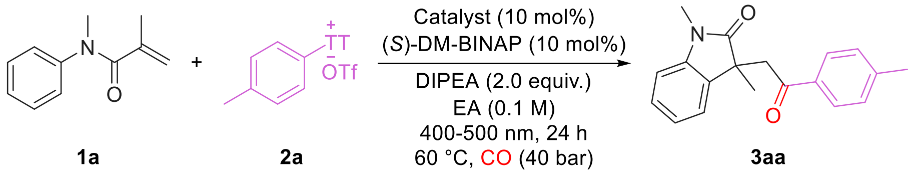

Entry	**Catalyst**	Yield (%)[b]
1	$Cu(OAc)_2$	39
2	CuI	41
3	$Cu(CH_3CN)_4BF_4$	38
4	$CuCl_2$	60
5	CuCl	74
6	CuTC	35
7	$CuBr·Me_2S$	42
8	$CuSO_4$	32
9	CuOTf	43

[a]The reaction was conducted using **1a** (0.1 mmol), **2a** (1.5 equiv.), catalyst (10 mol%), (*S*)-DM-BINAP (10 mol%), DIPEA (2.0 equiv.), EA (0.1 M), CO (40 bar), under blue-light irradiation at 60 °C for 24 h. [b]Determined by GC with hexadecane as internal standard.

3.2 The Effect of Ligand on the Reaction

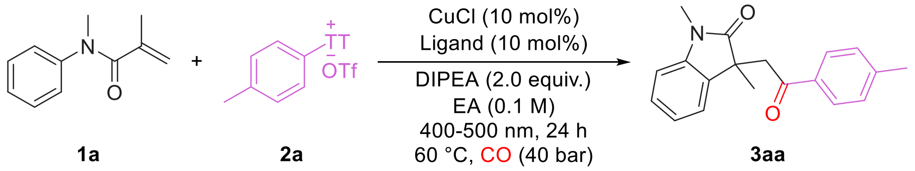

Entry	**Ligand**	Yield (%)[b]
1	(*S*)-DM-BINAP	74
2	BINAP	42
3	Xantphos	trace
4	Dppbz	trace
5	Tol-BINAP	61
6	Dppe	trace
7	Bpy	n.d.
8	1,10-phen	n.d.

[a]The reaction was conducted using **1a** (0.1 mmol), **2a** (1.5 equiv.), CuCl (10 mol%), Ligand (10 mol%), DIPEA (2.0 equiv.), EA (0.1 M), CO (40 bar), under blue-light irradiation at 60 °C for 24 h. [b]Determined by GC with hexadecane as internal standard.

3.3 The Effect of Base on the Reaction

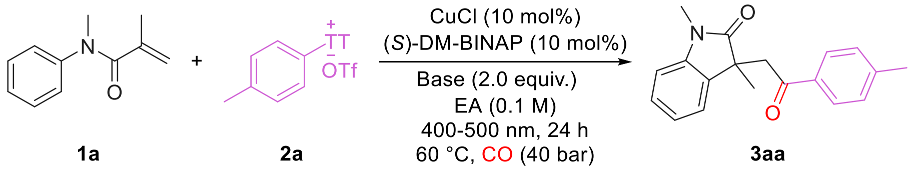

Entry	**Base**	Yield (%)^{b}
1	DIPEA	74
2	Et_3N	51
3	TMEDA	32
4	DABCO	26
5	DMAP	16
6	K_3PO_4	16
7	CsF	14
8	triisobutylamine	80
9	$NaHCO_3$	trace

^{a}The reaction was conducted using **1a** (0.1 mmol), **2a** (1.5 equiv.), CuCl (10 mol%), (*S*)-DM-BINAP (10 mol%), base (2.0 equiv.), EA (0.1 M), CO (40 bar), under blue-light irradiation at 60 °C for 24 h. ^{b}Determined by GC with hexadecane as internal standard.

3.4 The Effect of Solvent on the Reaction

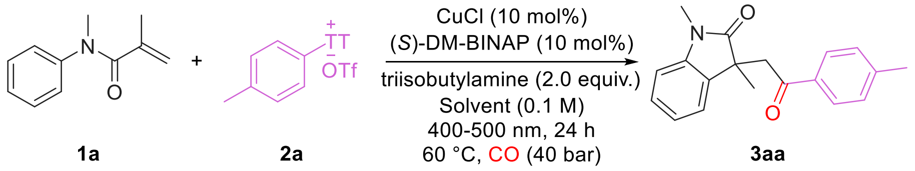

Entry	**Solvent**	Yield (%)^{b}
1	EA	80
2	DMC	66
3	DMF	11
4	DMSO	28
5	DMAc	17
6	$PhCF_3$	53
7	Et_2O	17
8	CH_3CN	25
9	DCE	35

^{a}The reaction was conducted using **1a** (0.1 mmol), **2a** (1.5 equiv.), CuCl (10 mol%), (*S*)-DM-BINAP (10 mol%), triisobutylamine (2.0 equiv.), solvent (0.1 M), CO (40 bar), under blue-light irradiation at 60 °C for 24 h. ^{b}Determined by GC with hexadecane as internal standard.

3.5 The Effect of other factors on the reaction

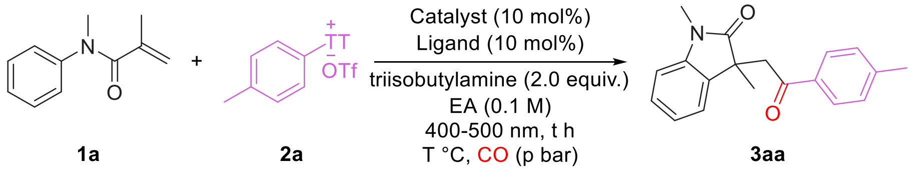

Entry	**2a** (eq.)	**Catalyst**	**Ligand**	**T** (°C)	**P** (bar)	**T** (h)	Yield (%)^b
1	1.5	CuCl	(*R*)-DM-BINAP	60	40	24	79
2	1.5	CuCl	(*S*)-DM-BINAP	60	40	24	80
3	1.5	CuCl	(*S*)-DM-BINAP	60	50	24	79
4	1.5	CuCl	(*S*)-DM-BINAP	60	30	24	71
5	1.5	CuCl	(*S*)-DM-BINAP	60	20	24	70
6	1.5	CuCl	1,10-Phen	60	40	24	n.d.
8	1.5	CuCl	bpy	60	40	24	n.d.
9	1.5	CuCl	PCy_3	60	40	24	n.d.
10	1.2	CuCl	(*S*)-DM-BINAP	60	40	24	69
11	2.0	CuCl	(*S*)-DM-BINAP	60	40	24	79
12	1.5	$Cu(CH_3CN)_4BF_4$	(*S*)-DM-BINAP	60	40	24	56
13	1.5	$Cu(acac)_2$	(*S*)-DM-BINAP	60	40	24	31
14	1.5	CuBr	(*S*)-DM-BINAP	60	40	24	36
15	1.5	CuCl	(*S*)-DM-BINAP	r.t.	40	24	65
16	1.5	CuCl	(*S*)-DM-BINAP	40	40	24	73
17	1.5	CuCl	(*S*)-DM-BINAP	40	40	13	68
18	1.5	CuCl	(*S*)-DM-BINAP	40	40	36	81

^{a}The reaction was conducted using **1a** (0.1 mmol), **2a** (X equiv.), catalyst (10 mol%), ligand (10 mol%), triisobutylamine (2.0 equiv.), EA (0.1 M), CO (P bar), under blue-light irradiation at T °C for t h. ^{b}Determined by GC with hexadecane as internal standard.

3.6 Control experiment

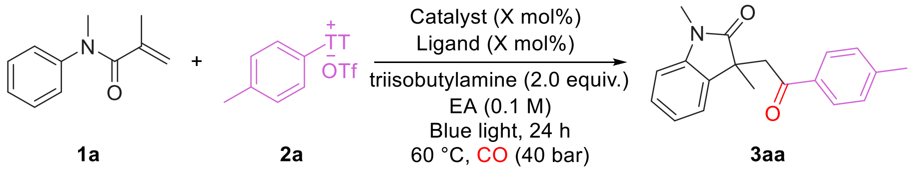

Entry	**CuCl**	**(*S*)-DM-BINAP**	**Base**	**Light**	Yield (%)^b
1	5	5	++	++	72
2	10	10	++	--	n.d.
3	--	10	++	++	n.d.
4	10	--	++	++	n.d.
5	10	10	--	++	trace

^{a}The reaction was conducted using **1a** (0.1 mmol), **2a** (x equiv.), CuCl (X mol%), (*S*)-DM-BINAP (X mol%), triisobutylamine (2.0 equiv.), EA (0.1 M), CO (40 bar), at 60 °C for 24 h. ^{b}Determined by GC with hexadecane as internal standard.

4. Characterization and Procedure of the Products

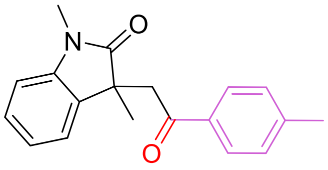

3aa

1,3-dimethyl-3-(2-oxo-2-(*p*-tolyl)ethyl)indolin-2-one

White solid (22.9 mg, 78% yield), purified by column chromatography (SiO_2, Pentane/EA= 3:1).

^{1}H NMR (300 MHz, $CDCl_3$) δ 7.84 – 7.67 (m, 2H), 7.55 – 7.10 (m, 4H), 6.99 (td, *J* = 7.5, 1.0 Hz, 1H), 6.94 – 6.88 (m, 1H), 3.78 – 3.55 (m, 2H), 3.33 (s, 3H), 2.39 (s, 3H), 1.46 (s, 3H).

^{13}C NMR (75 MHz, $CDCl_3$) δ 195.7, 180.6, 143.9, 143.8, 133.9, 133.8, 129.1, 128.0, 127.7, 122.1, 121.7, 108.1, 45.8, 45.2, 26.4, 24.9, 21.6.

HRMS (ESI) calcd for $C_{19}H_{19}NNaO_2$ [M+Na]$^+$: 316.1308, Found: 316.1305.

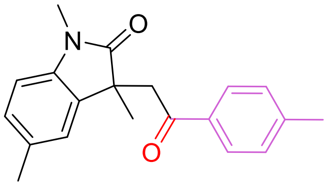

3ba

1,3,5-trimethyl-3-(2-oxo-2-(*p*-tolyl)ethyl)indolin-2-one

White solid (23.5 mg, 77% yield), purified by column chromatography (SiO_2, Pentane/EA= 3:1).

^{1}H NMR (300 MHz, $CDCl_3$) δ 7.82 – 7.69 (m, 2H), 7.19 (dt, *J* = 8.0, 0.7 Hz, 2H), 7.06 – 7.02 (m, 1H), 6.95 – 6.94 (m, 1H), 6.78 (d, *J* = 7.8 Hz, 1H), 3.76 – 3.51 (m, 2H), 3.29 (s, 3H), 2.37 (s, 3H), 2.27 (s, 3H), 1.42 (s, 3H).

^{13}C NMR (75 MHz, $CDCl_3$) δ 195.7, 180.6, 143.9, 141.4, 133.9, 133.8, 131.5, 129.1, 128.1, 128.0, 122.7, 107.8, 45.9, 45.3, 26.5, 25.0, 21.6, 21.1.

HRMS (ESI) calcd for $C_{20}H_{21}NNaO_2$ [M+Na]$^+$: 330.1464, Found: 330.1465.

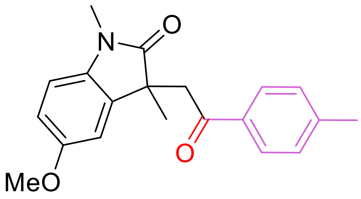

3ca

5-methoxy-1,3-dimethyl-3-(2-oxo-2-(*p*-tolyl)ethyl)indolin-2-one

White solid (22.7 mg, 70% yield), purified by column chromatography (SiO_2, Pentane/EA= 1:1).

^{1}H NMR (300 MHz, $CDCl_3$) δ 7.78 – 7.69 (m, 2H), 7.20 – 7.17 (dt, *J* = 7.9, 0.7 Hz, 2H), 6.88 – 6.72 (m, 3H), 3.73 (s, 3H), 3.64 (d, *J* = 2.5 Hz, 2H), 3.29 (s, 3H), 2.37 (s, 3H), 1.42 (s, 3H).

^{13}C NMR (75 MHz, $CDCl_3$) δ 195.7, 180.3, 155.7, 144.0, 137.4, 135.3, 133.9, 129.1, 128.1, 111.4, 109.9, 108.2, 55.7, 45.8, 45.7, 26.5, 24.9, 21.6.

HRMS (ESI) calcd for $C_{20}H_{21}NNaO_3$ [M+Na]$^+$: 346.1414, Found: 346.1404.

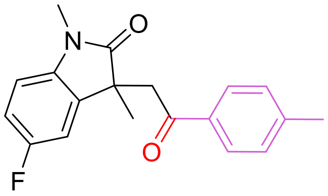

3da

5-fluoro-1,3-dimethyl-3-(2-oxo-2-(*p*-tolyl)ethyl)indolin-2-one

White solid (25.0 mg, 82% yield), purified by column chromatography (SiO_2, Pentane/EA= 4:1).

^{1}H NMR (300 MHz, $CDCl_3$) δ 7.81 – 7.71 (m, 2H), 7.27 – 7.17 (m, 2H), 7.01 – 6.89 (m, 2H), 6.87 – 6.79 (m, 1H), 3.67 (d, *J* = 1.1 Hz, 2H), 3.32 (s, 3H), 2.40 (s, 3H), 1.45 (s, 3H).

^{13}C NMR (75 MHz, $CDCl_3$) δ 195.5, 180.3, 159.1 (d, *J* = 239.7 Hz), 144.2, 139.8, 135.5 (d, *J* = 7.8 Hz), 133.7, 129.2, 128.1, 113.8 (d, *J* = 23.4 Hz), 110.1 (d, *J* = 24.8 Hz), 108.4 (d, *J* = 8.1 Hz), 45.8, 45.7 (d, *J* = 1.9 Hz), 26.6, 24.8, 21.6.

^{19}F NMR (282 MHz, $CDCl_3$) δ -121.3 (td, *J* = 8.5, 4.0 Hz).

HRMS (ESI) calcd for $C_{19}H_{18}FNNaO_2$ [M+Na]$^+$: 334.1214, Found: 334.1210.

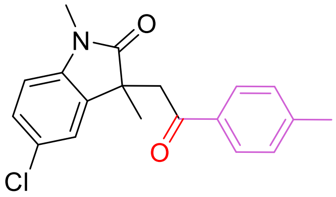

3ea

5-chloro-1,3-dimethyl-3-(2-oxo-2-(*p*-tolyl)ethyl)indolin-2-one

White solid (27.2 mg, 83% yield), purified by column chromatography (SiO_2, Pentane/EA= 3:1).

^{1}H NMR (300 MHz, $CDCl_3$) δ 7.83 – 7.66 (m, 2H), 7.24 – 7.16 (m, 3H), 7.09 (dd, *J* = 2.1, 0.4 Hz, 1H), 6.81 (d, *J* = 8.2 Hz, 1H), 3.66 (d, *J* = 0.8 Hz, 2H), 3.30 (s, 3H), 2.37 (s, 3H), 1.41 (s, 3H).

^{13}C NMR (75 MHz, $CDCl_3$) δ 195.4, 180.2, 144.2, 142.5, 135.6, 133.6, 129.2, 128.1, 127.6, 127.4, 122.2, 109.0, 45.9, 45.4, 26.6, 24.8, 21.6.

HRMS (ESI) calcd for $C_{19}H_{18}ClNNaO_2$ [M+Na]$^+$: 350.0918, Found: 350.0921.

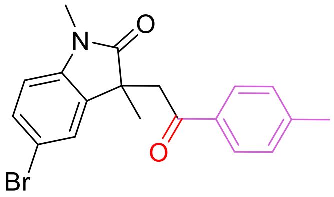

3fa

5-bromo-1,3-dimethyl-3-(2-oxo-2-(*p*-tolyl)ethyl)indolin-2-one

White solid (29.9 mg, 80% yield), purified by column chromatography (SiO_2, Pentane/EA= 3:1).

^{1}H NMR (300 MHz, $CDCl_3$) δ 7.83 – 7.69 (m, 2H), 7.39 (dd, *J* = 8.2, 2.0 Hz, 1H), 7.27 – 7.18 (m, 2H), 6.80 (d, *J* = 8.2 Hz, 1H), 3.69 (s, 2H), 3.32 (s, 3H), 2.40 (s, 3H), 1.44 (s, 3H).

^{13}C NMR (75 MHz, $CDCl_3$) δ 195.4, 180.1, 144.2, 143.0, 136.0, 133.6, 130.5, 129.2, 128.1, 124.9, 114.7, 109.6, 46.0, 45.4, 26.5, 24.9, 21.6.

HRMS (ESI) calcd for $C_{19}H_{18}BrNNaO_2$ $[M+Na]^+$: 394.0413, Found: 394.0415.

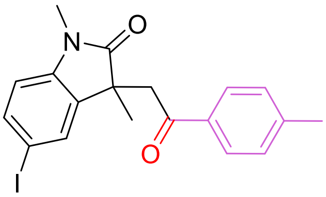

3ga

5-iodo-1,3-dimethyl-3-(2-oxo-2-(*p*-tolyl)ethyl)indolin-2-one

White solid (22.4 mg, 53% yield), purified by column chromatography (SiO_2, Pentane/EA= 3:1).

^{1}H NMR (300 MHz, $CDCl_3$) δ 7.78 – 7.59 (m, 2H), 7.49 (dd, *J* = 8.1, 1.8 Hz, 1H), 7.31 (dd, *J* = 1.8, 0.4 Hz, 1H), 7.17 – 7.08 (m, 2H), 6.68 – 6.55 (m, 1H), 3.58 (s, 2H), 3.22 (s, 3H), 2.31 (s, 3H), 1.33 (s, 3H).

^{13}C NMR (75 MHz, $CDCl_3$) δ 195.4, 179.9, 144.2, 143.7, 136.6, 136.4, 133.6, 130.4, 129.2, 128.1, 110.2, 84.6, 46.0, 45.2, 26.5, 24.9, 21.6.

HRMS (ESI) calcd for $C_{19}H_{18}INNaO_2$ $[M+Na]^+$: 442.0274, Found: 442.0283.

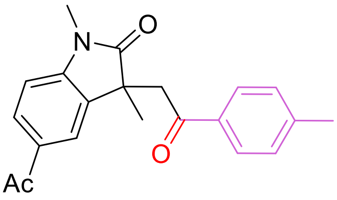

3ha

5-acetyl-1,3-dimethyl-3-(2-oxo-2-(*p*-tolyl)ethyl)indolin-2-one

White solid (27.0 mg, 83% yield), purified by column chromatography (SiO_2, Pentane/EA= 3:1).

^{1}H NMR (300 MHz, $CDCl_3$) δ 7.93 (dd, *J* = 8.2, 1.7 Hz, 1H), 7.84 – 7.64 (m, 3H), 7.24 – 7.15 (m, 2H), 6.96 (dd, *J* = 8.2, 0.5 Hz, 1H), 3.94 – 3.61 (m, 2H), 3.38 (s, 3H), 2.55 (s, 3H), 2.39 (s, 3H), 1.45 (s, 3H).

^{13}C NMR (75 MHz, $CDCl_3$) δ 197.0, 195.5, 181.0, 148.5, 144.3, 134.4, 133.4, 131.7, 130.2, 129.2, 128.1, 121.1, 107.4, 46.1, 44.9, 26.7, 26.3, 24.9, 21.6.

HRMS (ESI) calcd for $C_{21}H_{21}NNaO_3$ $[M+Na]^+$: 358.1413, Found: 358.1416.

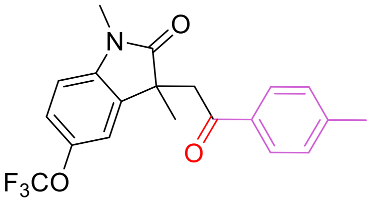

3ia

1,3-dimethyl-3-(2-oxo-2-(*p*-tolyl)ethyl)-5-(trifluoromethoxy)indolin-2-one

White solid (32.4 mg, 86% yield), purified by column chromatography (SiO_2, Pentane/EA= 4:1).

^{1}H NMR (300 MHz, $CDCl_3$) δ 7.92 – 7.62 (m, 2H), 7.25 – 7.20 (m, 2H), 7.17 – 7.12 (m, 1H), 6.89 (d, *J* = 8.4 Hz, 1H), 3.69 (d, *J* = 1.9 Hz, 2H), 3.34 (s, 3H), 2.40 (s, 3H), 1.46 (s, 3H).

^{13}C NMR (75 MHz, CDCl3) δ 195.5, 180.4, 144.5 (q, *J* = 2.0 Hz), 144.3, 142.5, 135.4, 133.6, 129.2, 128.0, 120.7, 120.5 (q, *J* = 256.4 Hz), 115.9, 108.4, 45.9, 45.6, 26.6, 24.7, 21.6.

^{19}F NMR (282 MHz, $CDCl_3$) δ -58.30.

HRMS (ESI) calcd for $C_{20}H_{18}F_3NNaO_3$ $[M+Na]^+$: 400.1131, Found: 400.1129.

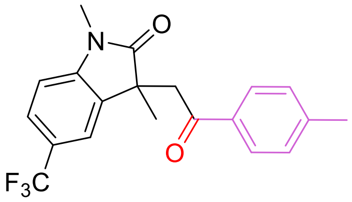

3ja

1,3-dimethyl-3-(2-oxo-2-(*p*-tolyl)ethyl)-5-(trifluoromethyl)indolin-2-one

White solid (31.4 mg, 87% yield), purified by column chromatography (SiO_2, Pentane/EA= 4:1).

^{1}H NMR (300 MHz, $CDCl_3$) δ 7.78 – 7.71 (m, 2H), 7.60 – 7.50 (m, 1H), 7.38 (dd, *J* = 1.8, 0.4 Hz, 1H), 7.23 – 7.18 (m, 2H), 6.75 – 6.62 (m, 1H), 3.65 (s, 2H), 3.29 (s, 3H), 2.38 (s, 3H), 1.40 (s, 3H).

^{13}C NMR (75 MHz, $CDCl_3$) δ 195.4, 180.6, 146.9, 144.3, 134.5, 133.5, 129.2, 128.1, 125.7 (q, *J* = 4.0 Hz), 124.5 (q, *J* = 271.4 Hz), 124.2 (q, *J* = 32.2 Hz), 118.5 (q, *J* = 3.7 Hz), 107.8, 46.0, 45.1, 26.6, 24.8, 21.6.

^{19}F NMR (282 MHz, $CDCl_3$) δ -61.2.

HRMS (ESI) calcd for $C_{20}H_{18}F_3NNaO_2$ $[M+Na]^+$: 384.1182, Found: 384.1186.

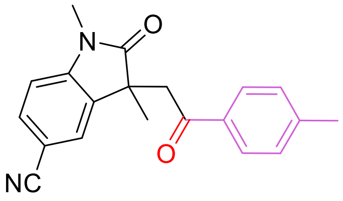

3ka

1,3-dimethyl-2-oxo-3-(2-oxo-2-(*p*-tolyl)ethyl)indoline-5-carbonitrile

White solid (28.0 mg, 88% yield), purified by column chromatography (SiO_2, Pentane/EA= 2:1).

^{1}H NMR (300 MHz, $CDCl_3$) δ 7.78 – 7.67 (m, 2H), 7.59 (dd, *J* = 8.1, 1.6 Hz, 1H), 7.35 (dd, *J* = 1.7, 0.5 Hz, 1H), 7.21 (dt, *J* = 8.0, 0.7 Hz, 2H), 6.96 (dd, *J* = 8.1, 0.5 Hz, 1H), 3.71 (d, *J* = 1.3 Hz, 2H), 3.35 (s, 3H), 2.39 (s, 3H), 1.43 (s, 3H).

^{13}C NMR (75 MHz, $CDCl_3$) δ 195.3, 180.4, 147.9, 144.5, 135.0, 133.3, 129.3, 128.1, 124.8, 119.4, 108.5, 105.0, 46.0, 44.9, 26.7, 24.7, 21.6.

HRMS (ESI) calcd for $C_{20}H_{18}N_2NaO_2$ $[M+Na]^+$: 341.1260, Found: 341.1260.

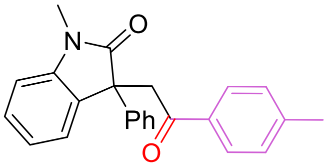

3la

1-methyl-3-(2-oxo-2-(*p*-tolyl)ethyl)-3-phenylindolin-2-one

White solid (30.5 mg, 86% yield), purified by column chromatography (SiO_2, Pentane/EA= 3:1).

^{1}H NMR (300 MHz, $CDCl_3$) δ 7.82 – 7.75 (m, 2H), 7.61 – 7.42 (m, 2H), 7.39 – 7.25 (m, 5H), 7.24 – 7.16 (m, 2H), 7.06 (td, *J* = 7.6, 1.1 Hz, 1H), 6.98 – 6.95 (m, 1H), 4.46 – 3.90 (m, 2H), 3.32 (s, 3H), 2.40 (s, 3H).

^{13}C NMR (75 MHz, $CDCl_3$) δ 195.4, 178.7, 144.8, 144.1, 139.6, 133.9, 131.6, 129.2, 128.6, 128.3, 128.1, 127.5, 126.7, 124.1, 122.1, 108.4, 53.1, 46.9, 26.7, 21.6.

HRMS (ESI) calcd for $C_{24}H_{21}NNaO_2$ $[M+Na]^+$: 378.1464, Found: 378.1460.

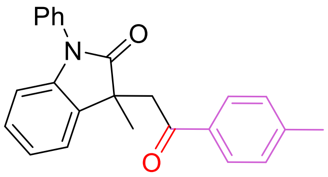

3ma

3-methyl-3-(2-oxo-2-(*p*-tolyl)ethyl)-1-phenylindolin-2-one

White solid (29.8 mg, 84% yield), purified by column chromatography (SiO_2, Pentane/EA= 4:1).

^{1}H NMR (300 MHz, $CDCl_3$) δ 7.87 – 7.71 (m, 2H), 7.61 – 7.49 (m, 4H), 7.47 – 7.36 (m, 1H), 7.22 – 7.09 (m, 4H), 6.99 (ddd, *J* = 7.7, 7.2, 1.0 Hz, 1H), 6.89 – 6.79 (m, 1H), 3.90 – 3.61 (m, 2H), 2.38 (s, 3H), 1.56 (s, 3H).

^{13}C NMR (75 MHz, $CDCl_3$) δ 195.6, 180.2, 144.1, 143.9, 135.1, 133.9, 133.6, 129.5, 129.1, 128.1, 127.8, 127.6, 127.0, 122.5, 121.8, 109.3, 46.6, 45.3, 25.3, 21.6.

HRMS (ESI) calcd for $C_{24}H_{21}NNaO_2$ $[M+Na]^+$: 378.1464, Found: 378.1469.

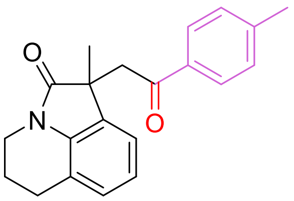

3na

1-methyl-1-(2-oxo-2-(*p*-tolyl)ethyl)-5,6-dihydro-4*H*-pyrrolo[3,2,1-*ij*]quinolin-2(1*H*)-one

White solid (22.5 mg, 70% yield), purified by column chromatography (SiO_2, Pentane/EA= 4:1).

^{1}H NMR (300 MHz, $CDCl_3$) δ 7.74 (d, *J* = 8.3 Hz, 2H), 7.22 – 7.10 (m, 2H), 7.08 – 6.96 (m, 2H), 6.93 – 6.81 (m, 1H), 3.82 – 3.78 (m, 2H), 3.71 – 3.46 (m, 2H), 2.85 – 2.79 (m, 2H), 2.37 (s, 3H), 2.17 – 1.96 (m, 2H), 1.45 (s, 3H).

^{13}C NMR (75 MHz, $CDCl_3$) δ 195.9, 179.5, 143.8, 139.6, 134.0, 132.3, 129.1, 128.1, 126.6, 121.6, 120.0, 119.8, 46.6, 45.7, 38.9, 24.7, 24.5, 21.6, 21.2.

HRMS (ESI) calcd for $C_{21}H_{21}NNaO_2$ $[M+Na]^+$: 342.1464, Found: 342.1459.

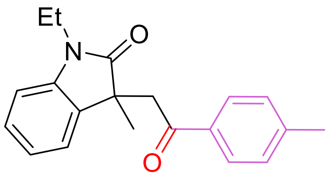

3oa

1-ethyl-3-methyl-3-(2-oxo-2-(*p*-tolyl)ethyl)indolin-2-one

White solid (23.8 mg, 77% yield), purified by column chromatography (SiO_2, Pentane/EA= 4:1).

^{1}H NMR (300 MHz, $CDCl_3$) δ 7.82 – 7.67 (m, 2H), 7.51 – 7.13 (m, 4H), 7.10 – 6.85 (m, 2H), 4.01 – 3.77 (m, 2H), 3.76 – 3.58 (m, 2H), 2.39(s, 3H), 1.45 (s, 3H), 1.37 (t, J = 7.2 Hz, 3H).

^{13}C NMR (75 MHz, $CDCl_3$) δ 195.7, 180.2, 143.8, 142.8, 134.1, 134.0, 129.1, 128.1, 127.7, 121.9, 121.8, 108.3, 45.8, 45.2, 34.7, 25.0, 21.6, 12.4.

HRMS (ESI) calcd for $C_{20}H_{21}NNaO_2$ $[M+Na]^+$: 330.1464, Found: 330.1469.

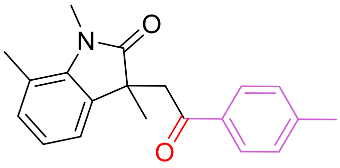

3pa

1,3,7-trimethyl-3-(2-oxo-2-(*p*-tolyl)ethyl)indolin-2-one

White solid (17.1 mg, 57% yield), purified by column chromatography (SiO_2, Pentane/EA= 3:1).

^{1}H NMR (300 MHz, $CDCl_3$) δ 7.87 – 7.58 (m, 2H), 7.24 – 7.12 (m, 2H), 6.98 – 6.92 (m, 2H), 6.84 (t, J = 7.4 Hz, 1H), 3.65 (d, J = 4.0 Hz, 2H), 3.59 (s, 3H), 2.62 (s, 3H), 2.37 (s, 3H), 1.39 (s, 3H).

^{13}C NMR (75 MHz, $CDCl_3$) δ 195.8, 181.5, 143.9, 141.6, 134.5, 134.0, 131.6, 129.2, 128.1, 122.0, 119.7, 119.5, 46.2, 44.7, 29.9, 25.5, 21.6, 19.2.

HRMS (ESI) calcd for $C_{20}H_{21}NNaO_2$ $[M+Na]^+$: 330.1464, Found: 330.1473.

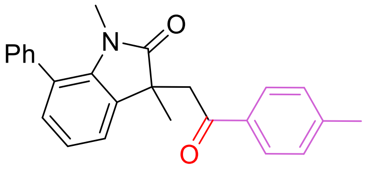

3qa

1,3-dimethyl-3-(2-oxo-2-(*p*-tolyl)ethyl)-7-phenylindolin-2-one

White solid (19.6 mg, 53% yield), purified by column chromatography (SiO_2, Pentane/EA= 3:1).

^{1}H NMR (300 MHz, $CDCl_3$) δ 7.85 – 7.69 (m, 2H), 7.49 – 7.34 (m, 5H), 7.23 – 7.17 (m, 2H), 7.15 – 7.03 (m, 2H), 7.00 – 6.95 (m, 1H), 3.83 – 3.58 (m, 2H), 2.83 (s, 3H), 2.38 (s, 3H), 1.48 (s, 3H).

^{13}C NMR (75 MHz, $CDCl_3$) δ 195.8, 181.8, 143.9, 140.8, 139.2, 134.9, 134.0, 130.8, 130.0, 129.1, 128.1, 127.5, 125.4, 121.4, 120.7, 46.3, 44.6, 30.5, 25.4, 21.6.

HRMS (ESI) calcd for $C_{25}H_{23}NNaO_2$ $[M+Na]^+$: 392.1621, Found: 392.1623.

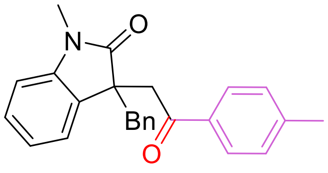

3ra

3-benzyl-1-methyl-3-(2-oxo-2-(*p*-tolyl)ethyl)indolin-2-one

White solid (23.8 mg, 64% yield), purified by column chromatography (SiO_2, Pentane/EA= 3:1).

^{1}H NMR (300 MHz, $CDCl_3$) δ 7.89 – 7.66 (m, 2H), 7.22 – 7.01 (m, 7H), 6.94 (td, *J* = 7.5, 1.0 Hz, 1H), 6.89 – 6.77 (m, 2H), 6.62 (ddd, *J* = 7.8, 1.0, 0.6 Hz, 1H), 3.89 – 3.68 (m, 2H), 3.20 – 3.05 (m, 2H), 3.04 (s, 3H), 2.38 (s, 3H).

^{13}C NMR (75 MHz, $CDCl_3$) δ 195.4, 179.2, 144.2, 144.0, 134.9, 134.0, 131.0, 130.0, 129.1, 128.1, 127.9, 127.4, 126.6, 122.6, 121.6, 107.8, 50.9, 44.8, 44.5, 26.0, 21.6.

HRMS (ESI) calcd for $C_{25}H_{23}NNaO_2$ $[M+Na]^+$: 392.1621, Found: 392.1630.

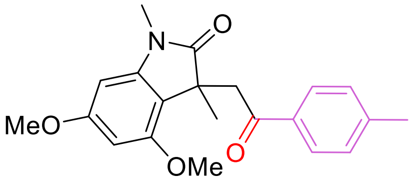

3sa

4,6-dimethoxy-1,3-dimethyl-3-(2-oxo-2-(*p*-tolyl)ethyl)indolin-2-one

White solid (26.0 mg, 74% yield), purified by column chromatography (SiO_2, Pentane/EA= 1:1).

^{1}H NMR (300 MHz, $CDCl_3$) δ 7.78 – 7.72 (m, 2H), 7.18 (dt, *J* = 8.0, 0.7 Hz, 2H), 6.15 (d, *J* = 2.0 Hz, 1H), 6.05 (d, *J* = 2.0 Hz, 1H), 4.07 (d, *J* = 17.5 Hz, 1H), 3.80 (s, 3H), 3.70 (s, 3H), 3.43 (d, *J* = 17.6 Hz, 1H), 3.27 (s, 3H), 2.36 (s, 3H), 1.44 (s, 3H).

^{13}C NMR (75 MHz, $CDCl_3$) δ 196.7, 181.6, 161.1, 155.8, 145.70, 143.6, 134.2, 129.0, 128.1, 111.0, 92.0, 88.4, 55.5, 55.2, 45.1, 44.5, 26.6, 22.9, 21.6.

HRMS (ESI) calcd for $C_{21}H_{23}NNaO_4$ $[M+Na]^+$: 376.1519, Found: 376.1523.

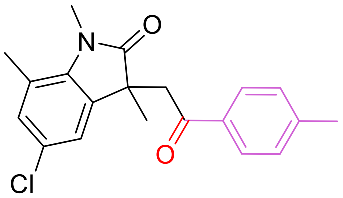

3ta

5-chloro-1,3,7-trimethyl-3-(2-oxo-2-(*p*-tolyl)ethyl)indolin-2-one

White solid (24.9 mg, 73% yield), purified by column chromatography (SiO_2, Pentane/EA= 1:1).

^{1}H NMR (300 MHz, $CDCl_3$) δ 7.74 (d, *J* = 8.2 Hz, 2H), 7.20 (dt, *J* = 8.0, 0.7 Hz, 2H), 6.97 – 6.87 (m, 2H), 3.65 (d, *J* = 0.8 Hz, 2H), 3.57 (s, 3H), 2.59 (s, 3H), 2.38 (s, 3H), 1.38 (s, 3H).

^{13}C NMR (75 MHz, $CDCl_3$) δ 195.5, 181.1, 144.2, 140.4, 136.4, 133.7, 131.0, 129.2, 128.1, 127.0, 121.2, 119.8, 46.3, 44.8, 29.8, 25.4, 21.6, 18.9.

HRMS (ESI) calcd for $C_{20}H_{20}ClNNaO_2$ $[M+Na]^+$: 364.1075, Found: 364.1071.

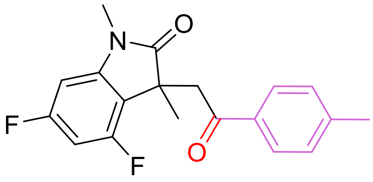

3ua

4,6-difluoro-1,3-dimethyl-3-(2-oxo-2-(*p*-tolyl)ethyl)indolin-2-one

White solid (18.3 mg, 56% yield), purified by column chromatography (SiO_2, Pentane/EA= 8:1).

^{1}H NMR (300 MHz, $CDCl_3$) δ 7.75 (d, J = 8.2 Hz, 2H), 7.20 (dt, J = 8.0, 0.7 Hz, 2H), 6.52 – 6.44 (m, 1H), 6.37 (td, J = 9.8, 2.1 Hz, 1H), 3.95 (dd, J = 18.0, 0.6 Hz, 1H), 3.62 (d, J = 18.0 Hz, 1H), 3.29 (s, 3H), 2.38 (s, 3H), 1.49 (s, 3H).

^{13}C NMR (75 MHz, $CDCl_3$) δ 195.9, 180.3, 164.7, 156.2, 146.7, 144.3, 133.5, 129.2, 128.1, 97.2 (t, J = 25.8 Hz), 93.5 (dd, J = 27.5, 3.5 Hz), 45.2, 44.6 (d, J = 2.1 Hz), 27.0, 23.4, 21.6.

^{19}F NMR (282 MHz, $CDCl_3$) δ -109.5 (dd, J = 16.4, 8.8 Hz), -120.4 (dd, J = 9.6, 6.8 Hz).

HRMS (ESI) calcd for $C_{19}H_{17}F_2NNaO_2$ [M+Na]$^+$: 352.1119, Found: 352.1123.

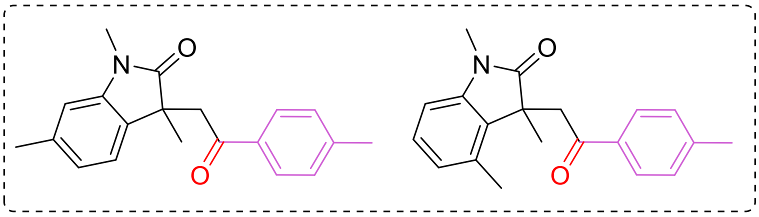

3va+3v′a (1.8:1)

1,3,6-trimethyl-3-(2-oxo-2-(*p*-tolyl)ethyl)indolin-2-one (3va)

1,3,4-trimethyl-3-(2-oxo-2-(*p*-tolyl)ethyl)indolin-2-one (3v´a)

White solid (18.8 mg, 61% yield), purified by column chromatography (SiO_2, Pentane/EA= 3:1).

^{1}H NMR (300 MHz, $CDCl_3$) δ 7.80 – 7.66 (m, 2H), 7.24 – 7.00 (m, 3H), 6.85 – 6.69 (m, 2H), 4.02 – 3.45 (m, 2H), 3.27 (s, 3H), 2.37 (s, 6H), 1.48 (s, 3H).

^{13}C NMR (75 MHz, $CDCl_3$) δ 195.8, 195.8, 180.9, 180.5, 144.1, 143.9, 137.7, 133.9, 133.7, 132.7, 130.8, 130.5, 129.1, 129.1, 128.0, 128.0, 127.5, 124.7, 122.5, 121.5, 109.1, 105.9, 46.0, 45.8, 45.0, 44.8, 26.5, 26.4, 24.9, 22.8, 21.8, 21.5, 18.2.

HRMS (ESI) calcd for $C_{20}H_{21}NNaO_2$ [M+Na]$^+$: 330.1464, Found: 330.1464.

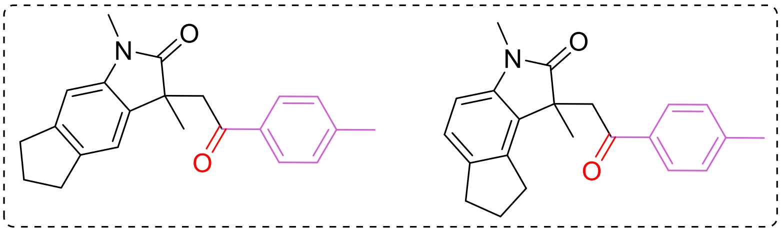

3wa+3w′a (1.7:1)

1,3-dimethyl-3-(2-oxo-2-(*p*-tolyl)ethyl)-3,5,6,7-tetrahydrocyclopenta[*f*]indol-2(1*H*)-one (3wa)

1,3-dimethyl-1-(2-oxo-2-(*p*-tolyl)ethyl)-3,6,7,8-tetrahydrocyclopenta[e]indol-2(1*H*)-one (3w´a)

White solid (23.5 mg, 71% yield), purified by column chromatography (SiO_2, Pentane/EA= 4:1).

^{1}H NMR (300 MHz, $CDCl_3$) δ 7.79 – 7.69 (m, 2H), 7.23 – 7.15 (m, 2H), 7.12 – 6.97 (m, 1H), 6.80 – 6.66 (m, 1H), 3.88 – 3.55 (m, 2H), 3.29 (s, 3H), 2.97 – 2.64 (m, 4H), 2.37 (s, 3H), 2.13 – 1.94 (m, 2H), 1.43 (s, 3H).

^{13}C NMR (75 MHz, $CDCl_3$) δ 271.6, 195.8, 195.8, 180.9, 143.9, 142.3, 139.0, 138.2, 133.7, 129.1, 128.1, 128.1, 123.0, 118.0, 106.1, 104.8, 45.9, 45.5, 45.2, 44.7, 33.2, 32.4, 31.6, 30.0, 26.7, 26.5, 25.7, 25.5, 25.2, 23.0, 21.6.

HRMS (ESI) calcd for $C_{22}H_{23}NNaO_2$ $[M+Na]^+$: 356.1621, Found: 356.1626.

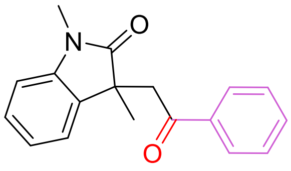

3ab

1,3-dimethyl-3-(2-oxo-2-phenylethyl)indolin-2-one[5]

White solid (21.1mg, 77% yield), purified by column chromatography (SiO_2, Pentane/EA= 5:1).

^{1}H NMR (300 MHz, $CDCl_3$) δ 7.86 – 7.80 (m, 2H), 7.55 – 7.49 (m, 1H), 7.43 – 7.35 (m, 2H), 7.29 – 7.21 (m, 1H), 7.17 – 7.11 (m, 1H), 6.97 (td, J = 7.5, 1.0 Hz, 1H), 6.93 – 6.87 (m, 1H), 3.82 – 3.58 (m, 2H), 3.31 (s, 3H), 1.44 (s, 3H).

^{13}C NMR (75 MHz, $CDCl_3$) δ 196.1, 180.6, 143.8, 136.3, 133.7, 133.1, 128.5, 127.9, 127.8, 122.1, 121.7, 108.1, 46.0, 45.3, 26.4, 24.9.

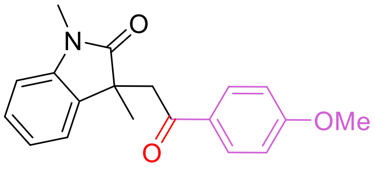

3ac

3-(2-(4-methoxyphenyl)-2-oxoethyl)-1,3-dimethylindolin-2-one

White solid (18.5 mg, 60% yield), purified by column chromatography (SiO_2, Pentane/EA= 3:1).

^{1}H NMR (300 MHz, $CDCl_3$) δ 7.86 – 7.78 (m, 2H), 7.25 (td, J = 7.7, 1.3 Hz, 1H), 7.16 – 7.11 (m, 1H), 6.97 (td, J = 7.5, 1.0 Hz, 1H), 6.91 – 6.83 (m, 3H), 3.83 (s, 3H), 3.72 – 3.56 (m, 2H), 3.31 (s, 3H), 1.43 (s, 3H).

^{13}C NMR (75 MHz, $CDCl_3$) δ 194.6, 180.7, 163.5, 143.8, 133.9, 130.2, 129.5, 127.7, 122.1, 121.7, 113.6, 108.1, 55.4, 45.6, 45.3, 26.4, 24.9.

HRMS (ESI) calcd for $C_{19}H_{19}NNaO_3$ $[M+Na]^+$: 332.1257, Found: 332.1253.

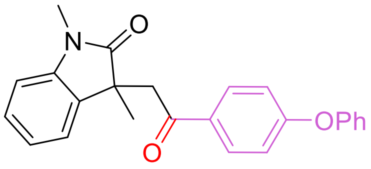

3ad

1,3-dimethyl-3-(2-oxo-2-(4-phenoxyphenyl)ethyl)indolin-2-one

White solid (24.1 mg, 65% yield), purified by column chromatography (SiO_2, Pentane/EA= 5:1).

^{1}H NMR (300 MHz, $CDCl_3$) δ 7.85 – 7.79 (m, 2H), 7.42 – 7.34 (m, 2H), 7.28 – 7.12 (m, 3H), 7.07 – 6.88 (m, 6H), 3.72 – 3.57 (m, 2H), 3.31 (s, 3H), 1.43 (s, 3H).

^{13}C NMR (75 MHz, $CDCl_3$) δ 194.6, 180.6, 162.0, 155.4, 143.8, 133.8, 131.0, 130.2, 130.0, 127.8, 124.6, 122.1, 121.7, 120.1, 117.1, 108.1, 45.8, 45.3, 26.4, 24.9.

HRMS (ESI) calcd for $C_{24}H_{21}NNaO_3$ $[M+Na]^+$: 394.1413, Found: 394.1417.

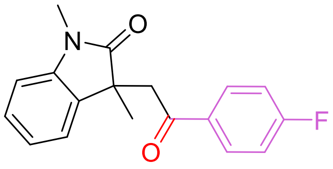

3ae

3-(2-(4-fluorophenyl)-2-oxoethyl)-1,3-dimethylindolin-2-one

White solid (24.3 mg, 82% yield), purified by column chromatography (SiO_2, Pentane/EA= 5:1).

^{1}H NMR (300 MHz, $CDCl_3$) δ 7.92 – 7.84 (m, 2H), 7.32 – 7.25 (m, 1H), 7.18 – 6.97 (m, 4H), 6.92 (dt, *J* = 7.8, 0.7 Hz, 1H), 3.67 (d, *J* = 2.0 Hz, 2H), 3.33 (s, 3H), 1.46 (s, 3H).

^{13}C NMR (75 MHz, CDCl3) δ 194.5, 180.5, 165.7 (d, *J* = 255.1 Hz), 143.8, 133.6, 132.8 (d, *J* = 3.0 Hz), 130.6 (d, *J* = 9.4 Hz), 127.9, 122.2, 121.7, 115.6 (d, *J* = 21.9 Hz).108.2, 45.9, 45.3, 26.4, 24.9.

^{19}F NMR (282 MHz, $CDCl_3$) δ -104.9 (ddd, *J* = 13.7, 8.3, 5.3 Hz).

HRMS (ESI) calcd for $C_{18}H_{16}FNNaO_2$ $[M+Na]^+$: 320.1057, Found: 320.1052.

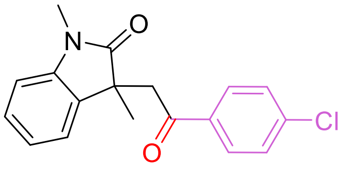

3af

3-(2-(4-chlorophenyl)-2-oxoethyl)-1,3-dimethylindolin-2-one

White solid (18.3 mg, 58% yield), purified by column chromatography (SiO_2, Pentane/EA= 5:1).

^{1}H NMR (300 MHz, $CDCl_3$) δ 7.83 – 7.76 (m, 2H), 7.42 – 7.36 (m, 2H), 7.32 – 7.26 (m, 1H), 7.18 – 7.12 (m, 1H), 7.01 (td, *J* = 7.5, 1.0 Hz, 1H), 6.95 – 6.90 (m, 1H), 3.66 (d, *J* = 1.8 Hz, 2H), 3.33 (s, 3H), 1.46 (s, 3H).

^{13}C NMR (75 MHz, $CDCl_3$) δ 194.9, 143.8, 139.6, 134.6, 133.5, 129.4, 128.8, 127.9, 122.2, 121.7, 108.2, 45.9, 45.3, 26.4, 24.9.

HRMS (ESI) calcd for $C_{18}H_{16}ClNNaO_2$ $[M+Na]^+$: 336.0761, Found: 336.0764.

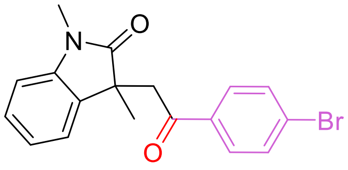

3ag

3-(2-(4-bromophenyl)-2-oxoethyl)-1,3-dimethylindolin-2-one

White solid (17.6 mg, 49% yield), purified by column chromatography (SiO_2, Pentane/EA= 5:1).

^{1}H NMR (300 MHz, $CDCl_3$) δ 7.74 – 7.68 (m, 2H), 7.56 (d, *J* = 8.6 Hz, 2H), 7.32 – 7.25 (m, 1H), 7.18 – 7.12 (m, 1H), 7.00 (td, *J* = 7.5, 1.0 Hz, 1H), 6.92 (dt, *J* = 7.8, 0.7 Hz, 1H), 3.65 (d, *J* = 1.7 Hz, 2H), 3.32 (s, 3H), 1.46 (s, 3H).

^{13}C NMR (75 MHz, $CDCl_3$) δ 195.1, 180.4, 143.8, 135.0, 133.5, 131.8, 129.5, 128.4, 127.9, 122.2, 121.7, 108.2, 45.9, 45.2, 26.4, 24.9.

HRMS (ESI) calcd for $C_{18}H_{16}BrNNaO_2$ $[M+Na]^+$: 380.0256, Found: 380.0266.

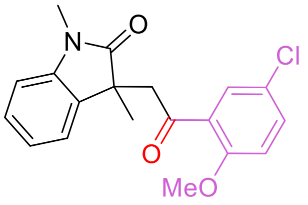

3ah

3-(2-(5-chloro-2-methoxyphenyl)-2-oxoethyl)-1,3-dimethylindolin-2-one

White solid (23.7 mg, 69% yield), purified by column chromatography (SiO_2, Pentane/EA= 3:1).

^1H NMR (300 MHz, $CDCl_3$) δ 7.36 – 7.31 (m, 2H), 7.28 – 7.23 (m, 1H), 7.13 – 7.09 (m, 1H), 6.98 (td, J = 7.5, 1.0 Hz, 1H), 6.90 – 6.83 (m, 2H), 3.91 (s, 3H), 3.67 (s, 2H), 3.26 (s, 3H), 1.39 (s, 3H).

^{13}C NMR (75 MHz, $CDCl_3$) δ 196.7, 180.6, 157.2, 143.8, 133.7, 133.1, 130.0, 128.3, 127.8, 125.9, 122.1, 121.8, 112.9, 108.0, 55.9, 51.2, 45.7, 26.4, 24.9.

HRMS (ESI) calcd for $C_{19}H_{18}ClNNaO_3$ $[M+Na]^+$: 366.0867, Found: 366.0869.

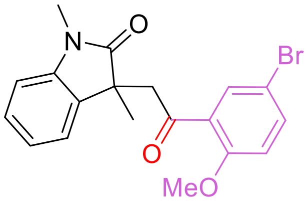

3ai

3-(2-(5-bromo-2-methoxyphenyl)-2-oxoethyl)-1,3-dimethylindolin-2-one

White solid (19.4 mg, 50% yield), purified by column chromatography (SiO_2, Pentane/EA= 3:1).

^1H NMR (300 MHz, $CDCl_3$) δ 7.50 – 7.44 (m, 2H), 7.26 (td, J = 7.7, 1.3 Hz, 1H), 7.13 – 7.09 (m, 1H), 6.98 (td, J = 7.5, 0.9 Hz, 1H), 6.87 (dt, J = 7.8, 0.7 Hz, 1H), 6.82 – 6.78 (m, 1H), 3.91 (s, 3H), 3.67 (d, J = 0.6 Hz, 2H), 3.26 (s, 3H), 1.39 (s, 3H).

^{13}C NMR (75 MHz, $CDCl_3$) δ 196.6, 180.5, 157.6, 143.8, 136.0, 133.7, 132.9, 128.7, 127.8, 122.1, 121.8, 113.3, 113.1, 108.0, 55.8, 51.2, 45.7, 26.4, 24.9.

HRMS (ESI) calcd for $C_{19}H_{18}BrNNaO_3$ $[M+Na]^+$: 410.0362, Found: 410.0370.

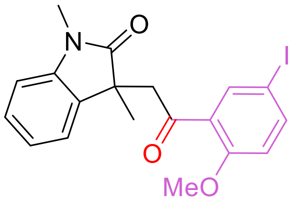

3aj

3-(2-(5-iodo-2-methoxyphenyl)-2-oxoethyl)-1,3-dimethylindolin-2-one

White solid (24.8 mg, 57% yield), purified by column chromatography (SiO_2, Pentane/EA= 3:1).

^{1}H NMR (300 MHz, $CDCl_3$) δ 8.27 (d, J = 2.2 Hz, 1H), 7.83 (dd, J = 8.6, 2.2 Hz, 1H), 7.28 – 7.22 (m, 1H), 7.15 – 7.09 (m, 1H), 6.97 (td, J = 7.5, 1.0 Hz, 1H), 6.89 (d, J = 7.8 Hz, 1H), 6.77 (d, J = 8.7 Hz, 1H), 3.91 (s, 3H), 3.68 – 3.53 (m, 2H), 3.31 (s, 3H), 1.43 (s, 3H).

^{13}C NMR (75 MHz, $CDCl_3$) δ 193.5, 180.6, 161.8, 143.9, 139.8, 133.7, 131.0, 130.2, 127.9, 122.2, 121.8, 110.0, 108.2, 85.8, 56.6, 45.7, 45.3, 26.5, 24.9.

HRMS (ESI) calcd for $C_{19}H_{18}INNaO_3$ $[M+Na]^+$: 458.0224, Found: 458.0229.

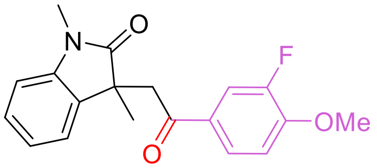

3ak

3-(2-(3-fluoro-4-methoxyphenyl)-2-oxoethyl)-1,3-dimethylindolin-2-one

White solid (24.0 mg, 73% yield), purified by column chromatography (SiO_2, Pentane/EA= 3:1).

^{1}H NMR (300 MHz, $CDCl_3$) δ 7.67 – 7.61 (m, 1H), 7.55 (dd, J = 11.9, 2.1 Hz, 1H), 7.29 – 7.22 (m, 1H), 7.15 – 7.10 (m, 1H), 7.01 – 6.88 (m, 3H), 3.92 (s, 3H), 3.69 – 3.53 (m, 2H), 3.31 (s, 3H), 1.43 (s, 3H).

^{13}C NMR (75 MHz, CDCl3) δ 193.76, 180.51, 151.9 (d, J = 10.9 Hz), 151.8 (d, J = 248.1 Hz), 143.8, 133.6, 129.6 (d, J = 5.0 Hz),127.9, 125.3 (d, J = 3.4 Hz), 122.1, 121.7, 115.6 (d, J = 19.1 Hz), 112.2 (d, J = 1.9 Hz), 108.1, 56.2, 45.6, 45.3, 26.4, 24.9.

^{19}F NMR (282 MHz, $CDCl_3$) δ -134.2.

HRMS (ESI) calcd for $C_{19}H_{18}FNNaO_3$ $[M+Na]^+$: 350.1163, Found: 350.1165.

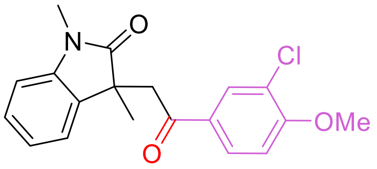

3al

3-(2-(3-chloro-4-methoxyphenyl)-2-oxoethyl)-1,3-dimethylindolin-2-one

White solid (22.4 mg, 65% yield), purified by column chromatography (SiO_2, Pentane/EA= 3:1).

^{1}H NMR (300 MHz, $CDCl_3$) δ 7.87 (d, J = 2.2 Hz, 1H), 7.75 (dd, J = 8.7, 2.2 Hz, 1H), 7.28 – 7.23 (m, 1H), 7.15 – 7.09 (m, 1H), 6.97 (td, J = 7.5, 1.0 Hz, 1H), 6.92 – 6.87 (m, 2H), 3.93 (s, 3H), 3.71 – 3.54 (m, 2H), 3.31 (s, 3H), 1.43 (s, 3H).

^{13}C NMR (75 MHz, $CDCl_3$) δ 193.7, 180.6, 158.8, 143.8, 133.7, 130.4, 130.0, 128.4, 127.9, 122.8, 122.2, 121.7, 111.2, 108.2, 56.4, 45.7, 45.3, 26.5, 24.9.

HRMS (ESI) calcd for $C_{19}H_{18}ClNNaO_3$ $[M+Na]^+$: 366.0867, Found: 366.0876.

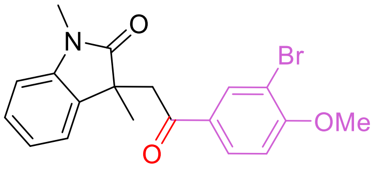

3am

3-(2-(3-bromo-4-methoxyphenyl)-2-oxoethyl)-1,3-dimethylindolin-2-one

White solid (24.8 mg, 57% yield), purified by column chromatography (SiO_2, Pentane/EA= 3:1).

^{1}H NMR (300 MHz, $CDCl_3$) δ 8.05 (d, J = 2.2 Hz, 1H), 7.79 (dd, J = 8.6, 2.2 Hz, 1H), 7.28 – 7.21 (m, 1H), 7.16 – 7.10 (m, 1H), 6.97 (td, J = 7.5, 1.0 Hz, 1H), 6.92 – 6.83 (m, 2H), 3.92 (s, 3H), 3.69 – 3.50 (m, 2H), 3.30 (s, 3H), 1.43 (s, 3H).

^{13}C NMR (75 MHz, $CDCl_3$) δ 193.6, 180.5, 159.6, 143.8, 133.6, 133.5, 130.4, 129.1, 127.9, 122.1, 121.7, 111.8, 111.0, 108.1, 56.4, 45.6, 45.3, 26.4, 24.9.

HRMS (ESI) calcd for $C_{19}H_{18}BrNNaO_3$ $[M+Na]^+$: 410.0362, Found: 410.0368.

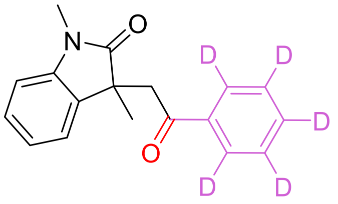

3an

1,3-dimethyl-3-(2-oxo-2-(phenyl-*d*$_5$)ethyl)indolin-2-one

White solid (17.2 mg, 61% yield), purified by column chromatography (SiO_2, Pentane/EA= 5:1).

^{1}H NMR (300 MHz, $CDCl_3$) δ 7.31 – 7.25 (m, 1H), 7.19 – 7.14 (m, 1H), 7.00 (td, J = 7.5, 1.0 Hz, 1H), 6.94 – 6.90 (m, 1H), 3.80 – 3.62 (m, 2H), 3.33 (s, 3H), 1.47 (s, 3H).

^{13}C NMR (75 MHz, $CDCl_3$) δ 196.1, 180.6, 143.8, 133.7, 127.8, 122.1, 121.7, 108.1, 46.0, 45.3, 26.4, 24.9.

HRMS (ESI) calcd for $C_{18}H_{12}D_5NNaO_2$ $[M+Na]^+$: 307.1465, Found: 307.1465.

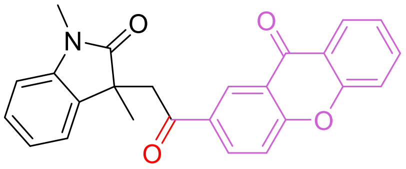

3ao

1,3-dimethyl-3-(2-oxo-2-(9-oxo-2,3,4,4a,6,7,9,9a-octahydro-1*H*-xanthen-7-yl)ethyl)indolin-2-one

White solid (18.9 mg, 48% yield), purified by column chromatography (SiO_2, Pentane/EA= 1:1).

^{1}H NMR (300 MHz, $CDCl_3$) δ 8.86 (d, J = 2.3 Hz, 1H), 8.39 – 8.31 (m, 1H), 8.15 (dd, J = 8.9, 2.3 Hz, 1H), 7.82 – 7.71 (m, 1H), 7.54 – 7.40 (m, 3H), 7.30 – 7.23 (m, 1H), 7.18 – 7.13 (m, 1H), 6.98 (td, J = 7.5, 1.0 Hz, 1H), 6.95 – 6.89 (m, 1H), 3.99 – 3.64 (m, 2H), 3.34 (s, 3H), 1.48 (s, 3H).

^{13}C NMR (75 MHz, $CDCl_3$) δ 194.4, 180.5, 176.6, 158.8, 155.9, 143.9, 135.4, 133.8, 133.6, 132.0, 127.9, 127.6, 126.8, 124.7, 122.2, 121.7, 121.6, 121.0, 118.8, 118.1, 108.2, 46.0, 45.2, 26.5, 25.0.

HRMS (ESI) calcd for $C_{25}H_{19}NNaO_4$ $[M+Na]^+$: 420.1206, Found: 420.1201.

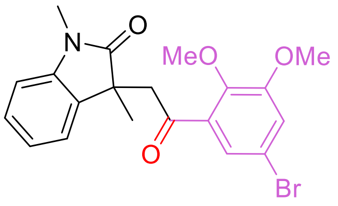

3ap

3-(2-(5-bromo-2,3-dimethoxyphenyl)-2-oxoethyl)-1,3-dimethylindolin-2-one

White solid (20.1 mg, 48% yield), purified by column chromatography (SiO_2, Pentane/EA= 1:1).

^{1}H NMR (300 MHz, $CDCl_3$) δ 7.30 – 7.22 (m, 1H), 7.19 – 7.13 (m, 1H), 6.99 (td, J = 7.5, 1.0 Hz, 1H), 6.95 (s, 1H), 6.86 (dt, J = 7.8, 0.8 Hz, 1H), 6.67 (s, 1H), 3.87 (s, 3H), 3.82 – 3.60 (m, 5H), 3.26 (s, 3H), 1.41 (s, 3H).

^{13}C NMR (75 MHz, $CDCl_3$) δ 198.7, 180.2, 151.4, 148.0, 143.8, 133.1, 132.5, 128.0, 122.4, 122.2, 116.0, 112.2, 110.9, 108.2, 56.3, 56.1, 49.8, 46.1, 26.5, 24.8.

HRMS (ESI) calcd for $C_{20}H_{20}BrNNaO_4$ $[M+Na]^+$: 440.0468, Found: 440.0474.

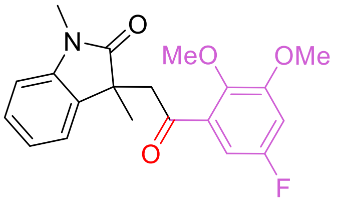

3aq

3-(2-(5-fluoro-2,3-dimethoxyphenyl)-2-oxoethyl)-1,3-dimethylindolin-2-one

White solid (14.6 mg, 41% yield; 95% purity), purified by column chromatography (SiO_2, Pentane/EA= 1:1).

^{1}H NMR (300 MHz, $CDCl_3$) δ 7.28 – 7.23 (m, 1H), 7.17 – 7.10 (m, 2H), 6.98 (td, J = 7.5, 1.0 Hz, 1H), 6.94 – 6.89 (m, 1H), 6.59 (d, J = 12.4 Hz, 1H), 3.90 (s, 3H), 3.76 (s, 3H), 3.68 (dd, J = 3.9, 2.2 Hz, 2H), 3.33 (s, 3H), 1.40 (s, 3H).

^{13}C NMR (75 MHz, CDCl3) δ 192.5, 180.8, 159.7, 154.3 (d, J = 10.9 Hz), 145.4, 143.9, 134.2, 127.7, 122.1, 121.4, 115.9 (d, J = 13.9 Hz), 110.7 (d, J = 4.3 Hz), 108.1, 99.7 (d, J = 30.8 Hz), 56.3 (d, J = 18.6 Hz), 50.7 (d, J = 9.6 Hz), 45.4 (d, J = 2.6 Hz), 26.5, 25.3, 22.0.

^{19}F NMR (282 MHz, $CDCl_3$) δ -105.5 – -118.2 (m).

HRMS (ESI) calcd for $C_{20}H_{20}FNNaO_4$ $[M+Na]^+$: 380.1269, Found: 380.1270.

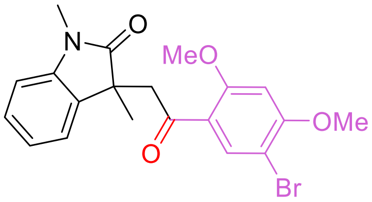

3ar

3-(2-(5-bromo-2,4-dimethoxyphenyl)-2-oxoethyl)-1,3-dimethylindolin-2-one

White solid (15.6 mg, 37% yield), purified by column chromatography (SiO_2, Pentane/EA= 1:1).

^{1}H NMR (300 MHz, $CDCl_3$) δ 7.77 (s, 1H), 7.28 – 7.22 (m, 1H), 7.13 – 7.07 (m, 1H), 6.97 (td, *J* = 7.5, 1.0 Hz, 1H), 6.88 (dt, *J* = 7.8, 0.8 Hz, 1H), 6.40 (s, 1H), 3.96 (s, 3H), 3.93 (s, 3H), 3.66 (s, 2H), 3.29 (s, 3H), 1.38 (s, 3H).

^{13}C NMR (75 MHz, $CDCl_3$) δ 194.4, 180.9, 160.2, 160.1, 143.9, 135.2, 134.2, 127.6, 122.0, 121.5, 120.6, 108.0, 102.9, 95.6, 56.4, 55.9, 51.2, 45.6, 26.4, 25.0.

HRMS (ESI) calcd for $C_{20}H_{20}BrNNaO_4$ $[M+Na]^+$: 440.0468, Found: 440.0476.

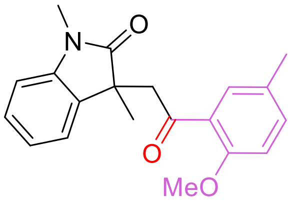

3as

3-(2-(2-methoxy-5-methylphenyl)-2-oxoethyl)-1,3-dimethylindolin-2-one

White solid (21.1 mg, 65% yield; 95% purity), purified by column chromatography (SiO_2, Pentane/EA= 3:1).

^{1}H NMR (300 MHz, $CDCl_3$) δ 7.27 – 7.17 (m, 3H), 7.14 – 7.09 (m, 1H), 6.96 (td, *J* = 7.5, 1.0 Hz, 1H), 6.86 (dt, *J* = 7.8, 0.7 Hz, 1H), 6.81 (d, *J* = 8.3 Hz, 1H), 3.89 (s, 3H), 3.79 – 3.63 (m, 2H), 3.28 (s, 3H), 2.18 (s, 3H), 1.39 (s, 3H).

^{13}C NMR (75 MHz, $CDCl_3$) δ 198.1, 180.9, 156.7, 143.9, 134.2, 134.1, 130.6, 129.8, 127.5, 126.9, 121.9, 121.8, 111.3, 107.9, 55.6, 51.3, 45.7, 26.4, 25.0, 20.0.

HRMS (ESI) calcd for $C_{20}H_{21}NNaO_3$ $[M+Na]^+$: 346.1413, Found: 346.1417.

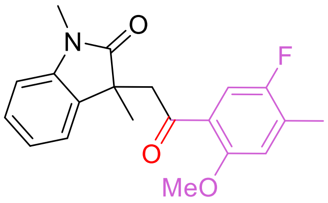

3at

3-(2-(5-fluoro-2-methoxy-4-methylphenyl)-2-oxoethyl)-1,3-dimethylindolin-2-one

White solid (16.5 mg, 48% yield), purified by column chromatography (SiO_2, Pentane/EA= 3:1).

^{1}H NMR (300 MHz, $CDCl_3$) δ 7.28 – 7.21 (m, 1H), 7.16 (d, *J* = 10.0 Hz, 1H), 7.13 – 7.08 (m, 1H), 6.96 (td, *J* = 7.5, 1.0 Hz, 1H), 6.91 – 6.85 (m, 1H), 6.74 – 6.68 (m, 1H), 3.90 (s, 3H), 3.77 – 3.60 (m, 2H), 3.29 (s, 3H), 2.26 (d, *J* = 1.9 Hz, 3H), 1.39 (s, 3H).

^{13}C NMR (101 MHz, $CDCl_3$) δ 195.9, 180.9, 155.3 (d, J = 239.1 Hz), 154.9 (d, J = 1.7 Hz), 143.9, 134.1, 131.2 (d, J = 19.3 Hz), 127.7, 125.5 (d, J = 5.8 Hz), 122.0, 121.6, 116.2 (d, J = 25.1 Hz), 114.2 (d, J = 4.4 Hz), 108.0, 56.06, 51.25, 45.64, 26.44, 25.06, 15.23, 15.20.

^{19}F NMR (376 MHz, $CDCl_3$) δ -127.7 – -127.8 (m).

HRMS (ESI) calcd for $C_{20}H_{20}FNNaO_3$ $[M+Na]^+$: 364.1319, Found: 364.1319.

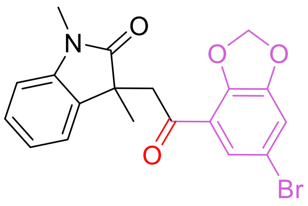

3au

3-(2-(6-bromobenzo[*d*][1,3]dioxol-4-yl)-2-oxoethyl)-1,3-dimethylindolin-2-one

White solid (15.3 mg, 38% yield; 95% purity), purified by column chromatography (SiO_2, Pentane/EA= 3:1).

^{1}H NMR (300 MHz, $CDCl_3$) δ 7.29 – 7.23 (m, 1H), 7.19 – 7.15 (m, 1H), 7.00 (td, J = 7.5, 1.0 Hz, 1H), 6.95 (s, 1H), 6.87 (dt, J = 7.7, 0.8 Hz, 1H), 6.62 (s, 1H), 5.98 (s, 2H), 3.69 – 3.51 (m, 2H), 3.27 (s, 3H), 1.40 (s, 3H).

^{13}C NMR (75 MHz, $CDCl_3$) δ 198.8, 180.1, 150.1, 147.3, 143.8, 134.2, 133.1, 128.0, 122.3, 122.2, 113.5, 111.2, 108.9, 108.2, 102.4, 49.7, 46.0, 26.5, 24.7.

HRMS (ESI) calcd for $C_{19}H_{16}BrNNaO_4$ $[M+Na]^+$: 424.0155, Found: 424.0150.

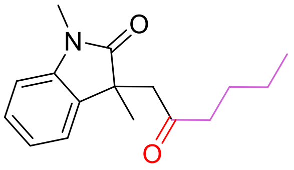

3av

1,3-dimethyl-3-(2-oxohexyl)indolin-2-one[6]

White solid (12.1 mg, 47% yield), purified by column chromatography (SiO_2, Pentane/EA= 5:1).

^{1}H NMR (300 MHz, $CDCl_3$) δ 7.26 – 7.22 (m, 1H), 7.16 – 7.09 (m, 1H), 7.00 (td, J = 7.5, 1.0 Hz, 1H), 6.86 (dt, J = 7.7, 0.8 Hz, 1H), 3.27 (s, 3H), 3.07 (s, 2H), 2.36 – 2.15 (m, 2H), 1.44 – 1.32 (m, 5H), 1.23 – 1.10 (m, 2H), 0.85 – 0.76 (m, 3H).

^{13}C NMR (75 MHz, $CDCl_3$) δ 207.1, 180.4, 127.9, 122.2, 121.7, 108.2, 49.7, 45.2, 42.5, 26.4, 25.5, 24.5, 22.1, 13.8.

HRMS (ESI) calcd for $C_{16}H_{21}NNaO_2$ $[M+Na]^+$: 282.1464, Found: 282.1466.

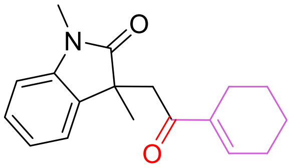

3aw

3-(2-(cyclohex-1-en-1-yl)-2-oxoethyl)-1,3-dimethylindolin-2-one

White solid (13.1 mg, 46% yield), purified by column chromatography (SiO_2, Pentane/EA= 5:1).

^{1}H NMR (300 MHz, $CDCl_3$) δ 7.26 – 7.20 (m, 1H), 7.12 – 7.07 (m, 1H), 6.98 (td, J = 7.5, 1.0 Hz, 1H), 6.90 – 6.84 (m, 2H), 3.35 (d, J = 6.8 Hz, 2H), 3.28 (s, 3H), 2.24 – 2.17 (m, 2H), 2.05 – 1.95 (m, 2H), 1.57 – 1.47 (m, 4H), 1.35 (s, 3H).

^{13}C NMR (75 MHz, $CDCl_3$) δ 197.0, 180.9, 143.8, 140.1, 139.0, 134.0, 127.7, 122.0, 121.7, 108.1, 45.3, 44.7, 26.4, 26.0, 25.0, 22.8, 21.8, 21.4.

HRMS (ESI) calcd for $C_{18}H_{21}NNaO_2$ $[M+Na]^+$: 306.1464, Found: 306.1464.

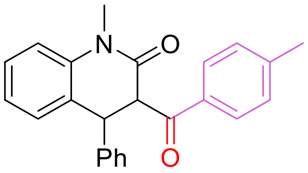

5aa

1-methyl-3-(4-methylbenzoyl)-4-phenyl-3,4-dihydroquinolin-2(1*H*)-one

White solid (19.2 mg, 46% yield), purified by column chromatography (SiO_2, Pentane/EA= 5:1).

^{1}H NMR (300 MHz, $CDCl_3$) δ 7.87 – 7.77 (m, 2H), 7.35 – 7.30 (m, 1H), 7.29 – 7.21 (m, 5H), 7.20 – 7.15 (m, 2H), 7.10 (dd, J = 8.2, 1.1 Hz, 1H), 7.00 (td, J = 7.4, 1.2 Hz, 1H), 6.91 – 6.85 (m, 1H), 4.92 (d, J = 7.4 Hz, 1H), 4.70 (d, J = 7.5 Hz, 1H), 3.45 (s, 3H), 2.39 (s, 3H).

^{13}C NMR (75 MHz, $CDCl_3$) δ 195.1, 166.7, 144.3, 140.2, 139.5, 134.0, 129.3, 129.0, 128.9, 128.7, 128.1, 128.0, 127.3, 127.3, 123.4, 114.9, 55.7, 44.9, 29.9, 21.6.

HRMS (ESI) calcd for $C_{24}H_{21}NNaO_2$ $[M+Na]^+$: 378.1464, Found: 378.1469.

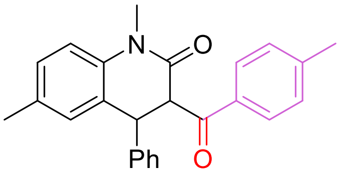

5ba

1,6-dimethyl-3-(4-methylbenzoyl)-4-phenyl-3,4-dihydroquinolin-2(1*H*)-one

White solid (17.7 mg, 48% yield), purified by column chromatography (SiO_2, Pentane/EA= 5:1).

^{1}H NMR (300 MHz, $CDCl_3$) δ 7.88 – 7.81 (m, 2H), 7.32 – 7.28 (m, 1H), 7.27 – 7.21 (m, 4H), 7.18 – 7.13 (m, 2H), 7.12 – 7.08 (m, 1H), 6.98 (d, J = 8.3 Hz, 1H), 6.71 (dd, J = 1.9, 1.0 Hz, 1H), 4.88 (d, J = 6.7 Hz, 1H), 4.62 (d, J = 6.7 Hz, 1H), 3.43 (s, 3H), 2.39 (s, 3H), 2.22 (s, 3H).

^{13}C NMR (75 MHz, $CDCl_3$) δ 195.0, 166.4, 144.3, 140.6, 137.2, 133.8, 133.0, 129.3, 129.0, 128.9, 128.6, 127.9, 127.3, 126.9, 114.8, 56.2, 45.0, 29.9, 21.7, 20.6.

HRMS (ESI) calcd for $C_{25}H_{23}NNaO_2$ $[M+Na]^+$: 392.1621, Found: 392.1628.

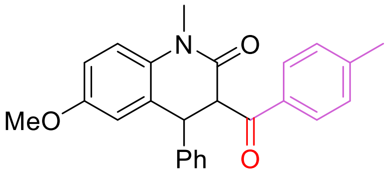

5ca

6-methoxy-1-methyl-3-(4-methylbenzoyl)-4-phenyl-3,4-dihydroquinolin-2(1*H*)-one

White solid (16.9 mg, 44% yield), purified by column chromatography (SiO_2, Pentane/EA= 5:1).

^{1}H NMR (400 MHz, $CDCl_3$) δ 7.90 – 7.82 (m, 2H), 7.33 – 7.30 (m, 1H), 7.27 – 7.23 (m, 3H), 7.20 – 7.17 (m, 2H), 7.04 (d, J = 8.9 Hz, 1H), 6.88 – 6.83 (m, 1H), 6.48 (dd, J = 2.8, 0.9 Hz, 1H), 4.90 (d, J = 7.2 Hz, 1H), 4.67 (d, J = 7.2 Hz, 1H), 3.72 (s, 3H), 3.44 (s, 3H), 2.41 (s, 3H).

^{13}C NMR (101 MHz, $CDCl_3$) δ 195.1, 166.2, 155.7, 144.3, 140.1, 134.0, 133.1, 129.3, 129.0, 128.9, 128.8, 127.9, 127.4, 115.8, 114.9, 112.4, 55.9, 55.4, 45.1, 30.0, 21.7.

HRMS (ESI) calcd for $C_{25}H_{23}NNaO_3$ $[M+Na]^+$: 408.1570, Found: 408.1560.

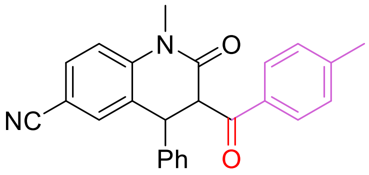

5da

1-methyl-3-(4-methylbenzoyl)-2-oxo-4-phenyl-1,2,3,4-tetrahydroquinoline-6-carbonitrile

White solid (19.3 mg, 51% yield), purified by column chromatography (SiO_2, Pentane/EA= 5:1).

^{1}H NMR (300 MHz, $CDCl_3$) δ 7.84 – 7.77 (m, 2H), 7.63 – 7.58 (m, 1H), 7.35 – 7.24 (m, 5H), 7.20 – 7.11 (m, 4H), 4.95 (d, J = 7.3 Hz, 1H), 4.69 (d, J = 7.3 Hz, 1H), 3.46 (s, 3H), 2.40 (s, 3H).

^{13}C NMR (101 MHz, $CDCl_3$) δ 194.3, 166.5, 144.9, 143.2, 138.8, 133.4, 132.4, 132.3, 129.5, 129.4, 128.9, 128.6, 128.0, 127.7, 118.5, 115.3, 106.7, 55.0, 44.5, 30.1, 21.7.

HRMS (ESI) calcd for $C_{25}H_{20}N_2NaO_2$ $[M+Na]^+$: 403.1417, Found: 403.1424.

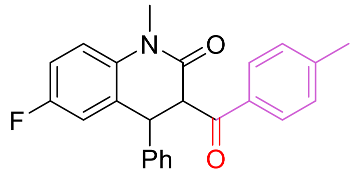

5ea

6-fluoro-1-methyl-3-(4-methylbenzoyl)-4-phenyl-3,4-dihydroquinolin-2(1*H*)-one

White solid (16.9 mg, 45% yield), purified by column chromatography (SiO_2, Pentane/EA= 5:1).

^{1}H NMR (300 MHz, $CDCl_3$) δ 7.85 – 7.79 (m, 2H), 7.33 – 7.27 (m, 2H), 7.26 – 7.21 (m, 3H), 7.18 – 7.14 (m, 2H), 7.07 – 6.95 (m, 2H), 6.65 – 6.58 (m, 1H), 4.90 (d, *J* = 7.8 Hz, 1H), 4.67 (d, *J* = 7.7 Hz, 1H), 3.43 (s, 3H), 2.39 (s, 3H).

^{13}C NMR (75 MHz, $CDCl_3$) δ 194.9, 166.4, 158.9 (d, *J* = 243.0 Hz), 144.5, 139.5, 134.0, 129.4, 129.2, 128.9, 128.0, 127.7, 116.2, 116.1, 115.8 (d, *J* = 23.7 Hz), 114.5 (d, *J* = 22.6 Hz), 55.3, 44.8, 30.2, 21.7.

^{19}F NMR (282 MHz, $CDCl_3$) δ -119.4 – -119.6 (m).

HRMS (ESI) calcd for $C_{24}H_{20}FNNaO_2$ $[M+Na]^+$: 396.1370, Found: 396.1372.

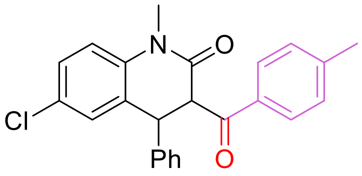

5fa

6-chloro-1-methyl-3-(4-methylbenzoyl)-4-phenyl-3,4-dihydroquinolin-2(1*H*)-one

White solid (19.8 mg, 51% yield), purified by column chromatography (SiO_2, Pentane/EA= 5:1).

^{1}H NMR (300 MHz, $CDCl_3$) δ 7.88 – 7.82 (m, 2H), 7.34 – 7.30 (m, 2H), 7.29 – 7.24 (m, 4H), 7.19 – 7.15 (m, 2H), 7.04 (d, *J* = 8.7 Hz, 1H), 6.91 (dd, *J* = 2.4, 0.9 Hz, 1H), 4.92 (d, *J* = 7.0 Hz, 1H), 4.66 (d, *J* = 7.1 Hz, 1H), 3.45 (s, 3H), 2.42 (s, 3H).

^{13}C NMR (75 MHz, $CDCl_3$) δ 194.6, 166.3, 144.5, 139.5, 138.2, 133.7, 129.4, 129.2, 129.1, 128.9, 128.7, 128.6, 128.0, 127.8, 127.7, 116.1, 55.5, 44.7, 30.0, 21.7.

HRMS (ESI) calcd for $C_{24}H_{20}ClNNaO_2$ $[M+Na]^+$: 412.1075, Found: 412.1076.

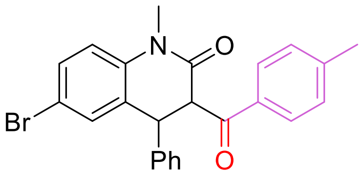

5ga

6-bromo-1-methyl-3-(4-methylbenzoyl)-4-phenyl-3,4-dihydroquinolin-2(1*H*)-one

White solid (24.4 mg, 56% yield), purified by column chromatography (SiO_2, Pentane/EA= 5:1).

^{1}H NMR (300 MHz, $CDCl_3$) δ 7.86 – 7.79 (m, 2H), 7.44 – 7.39 (m, 1H), 7.34 – 7.27 (m, 2H), 7.26 – 7.20 (m, 3H), 7.16 – 7.12 (m, 2H), 7.03 (dd, *J* = 2.3, 0.9 Hz, 1H), 6.96 (d, *J* = 8.7 Hz, 1H), 4.89 (d, *J* = 6.8 Hz, 1H), 4.63 (d, *J* = 6.9 Hz, 1H), 3.42 (s, 3H), 2.40 (s, 3H).

^{13}C NMR (75 MHz, $CDCl_3$) δ 194.6, 166.3, 144.6, 139.6, 138.7, 133.6, 131.5, 131.0, 129.5, 129.4, 129.2, 129.0, 127.8, 127.7, 116.6, 116.4, 55.7, 44.8, 30.0, 21.7.

HRMS (ESI) calcd for $C_{24}H_{20}BrNNaO_2$ $[M+Na]^+$: 456.0569, Found: 456.0566.

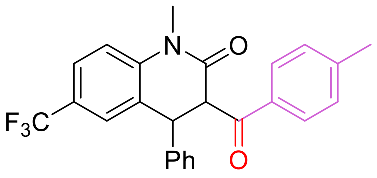

5ha

1-methyl-3-(4-methylbenzoyl)-4-phenyl-6-(trifluoromethyl)-3,4-dihydroquinolin-2(1*H*)-one

White solid (26.4 mg, 49% yield), purified by column chromatography (SiO_2, Pentane/EA= 5:1).

^{1}H NMR (300 MHz, $CDCl_3$) δ 7.84 (d, *J* = 8.3 Hz, 2H), 7.61 – 7.56 (m, 1H), 7.33 – 7.24 (m, 5H), 7.20 – 7.13 (m, 4H), 4.94 (d, *J* = 6.4 Hz, 1H), 4.70 (d, *J* = 6.5 Hz, 1H), 3.47 (s, 3H), 2.40 (s, 3H).

^{13}C NMR (75 MHz, CDCl3) δ 194.5, 166.5, 144.8, 142.4, 139.5, 133.4, 129.5, 129.3, 129.0, 127.9 (q, *J* = 23.5 Hz), 127.8, 127.7, 125.9, 125.7 (q, *J* = 3.5 Hz), 125.5 (q, *J* = 3.5 Hz), 123.7 (q, *J* = 240.3 Hz), 115.0, 55.7, 44.9, 30.1, 21.7.

^{19}F NMR (282 MHz, $CDCl_3$) δ -62.0.

HRMS (ESI) calcd for $C_{25}H_{20}F_3NNaO_2$ $[M+Na]^+$: 446.1338, Found: 446.1341.

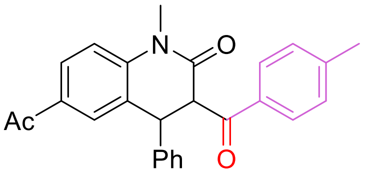

5ia

6-acetyl-1-methyl-3-(4-methylbenzoyl)-4-phenyl-3,4-dihydroquinolin-2(1*H*)-one

White solid (26.3 mg, 55% yield), purified by column chromatography (SiO_2, Pentane/EA= 5:1).

^{1}H NMR (300 MHz, $CDCl_3$) δ 7.96 – 7.90 (m, 1H), 7.87 – 7.82 (m, 2H), 7.58 (dd, *J* = 2.1, 0.8 Hz, 1H), 7.34 – 7.23 (m, 5H), 7.18 – 7.13 (m, 3H), 4.94 (d, *J* = 5.3 Hz, 1H), 4.68 (d, *J* = 5.3 Hz, 1H), 3.49 (s, 3H), 2.47 (s, 3H), 2.41 (s, 3H).

^{13}C NMR (75 MHz, $CDCl_3$) δ 196.6, 194.4, 166.5, 144.8, 143.5, 139.9, 133.1, 132.3, 129.5, 129.2, 129.1, 129.1, 129.0, 127.7, 127.5, 126.7, 114.8, 56.1, 45.0, 30.1, 26.3, 21.7.

HRMS (ESI) calcd for $C_{26}H_{23}NNaO_3$ $[M+Na]^+$: 420.1570, Found: 420.1561.

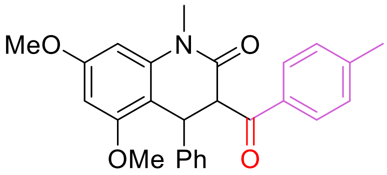

5ja

5,7-dimethoxy-1-methyl-3-(4-methylbenzoyl)-4-phenyl-3,4-dihydroquinolin-2(1*H*)-one

White solid (24.1 mg, 58% yield), purified by column chromatography (SiO_2, Pentane/EA= 1:1).

^{1}H NMR (300 MHz, $CDCl_3$) δ 8.01 – 7.92 (m, 2H), 7.34 – 7.29 (m, 3H), 7.28 – 7.23 (m, 2H), 7.21 – 7.16 (m, 2H), 6.35 (d, *J* = 2.2 Hz, 1H), 6.22 (d, *J* = 2.2 Hz, 1H), 4.82 (dd, *J* = 8.9, 1.3 Hz, 2H), 3.86 (s, 3H), 3.66 (s, 3H), 3.48 (s, 3H), 2.45 (s, 3H).

^{13}C NMR (75 MHz, $CDCl_3$) δ 194.5, 166.3, 160.4, 157.7, 144.5, 141.9, 141.4, 132.5, 129.5, 129.2, 128.9, 127.1, 127.0, 106.3, 94.1, 93.3, 57.7, 55.7, 55.4, 38.3, 30.3, 21.7.

HRMS (ESI) calcd for $C_{26}H_{25}NNaO_4$ $[M+Na]^+$: 438.1675, Found: 438.1668.

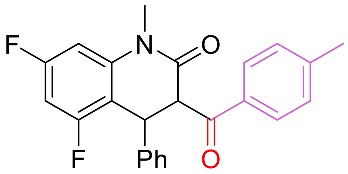

5ka

5,7-difluoro-1-methyl-3-(4-methylbenzoyl)-4-phenyl-3,4-dihydroquinolin-2(1*H*)-one

White solid (24.6 mg, 63% yield), purified by column chromatography (SiO_2, Pentane/EA= 5:1).

^{1}H NMR (300 MHz, $CDCl_3$) δ 7.92 – 7.81 (m, 2H), 7.27 – 7.18 (m, 5H), 7.12 – 7.01 (m, 2H), 6.59 (dt, *J* = 10.4, 1.9 Hz, 1H), 6.53 – 6.40 (m, 1H), 4.81 (d, *J* = 1.3 Hz, 1H), 4.67 (s, 1H), 3.35 (s, 3H), 2.36 (s, 3H).

^{13}C NMR (75 MHz, $CDCl_3$) 193.8, 165.5, 162.60 (d, *J* = 246.0 Hz),158.7, 156.9 (d, *J* = 246.2 Hz), 145.1, 142.1 (dd, *J* = 12.4, 8.4 Hz), 140.2, 132.0, 129.7, 129.3, 127.8, 126.7, 109.5 (dd, *J* = 20.8, 4.1 Hz), 99.2 (dd, *J* = 26.8, 3.3 Hz), 98.8 (t, *J* = 26.0 Hz), 56.9, 38.1 (d, *J* = 2.9 Hz), 30.3, 21.8.

^{19}F NMR (282 MHz, $CDCl_3$) δ -109.0 – -109.5 (m), -113.9 (t, *J* = 8.5 Hz).

HRMS (ESI) calcd for $C_{24}H_{19}F_2NNaO_2$ $[M+Na]^+$: 414.1276, Found: 414.1285.

5. Control Experiments

5.1 Radical Inhibition Experiment

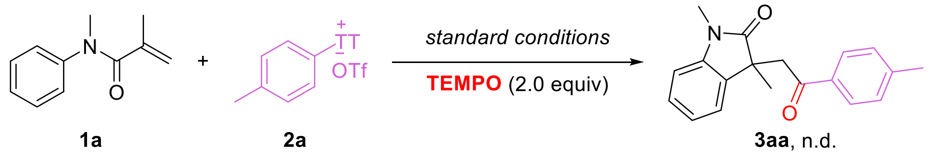

A 4 mL snap vial was charged with CuCl (10 mol%), (*S*)-DM-BINAP (10 mol%), thianthrenium Salts (1.5 equiv.), *N*-acrylamides (1.0 equiv.), 2,2,6,6-tetramethylpiperidine-1-oxyl (TEMPO) (2.0 equiv.) and closed with a rubber-based septum. The vial was evacuated and backfilled with argon. Degassed EA (0.1 M) and triisobutylamine (2.0 equiv.) were added via syringe. The vial was then connected to atmosphere with a cannula and transferred into a 300 mL Parr 4560 series autoclave, under argon counterflow. The closed autoclave was flushed three times with nitrogen (~ 5 bar), three times with CO (~ 5 bar), and 40 bar of carbon monoxide (measured by pressure meter) was charged. The autoclave was then placed into an aluminum block on a magnetic stirrer. The reaction mixture was stirred (500 rpm) under blue-light irradiation at 60 °C for 24 h. And a proper amount of solvent was taken for GC analysis. The result is shown above.

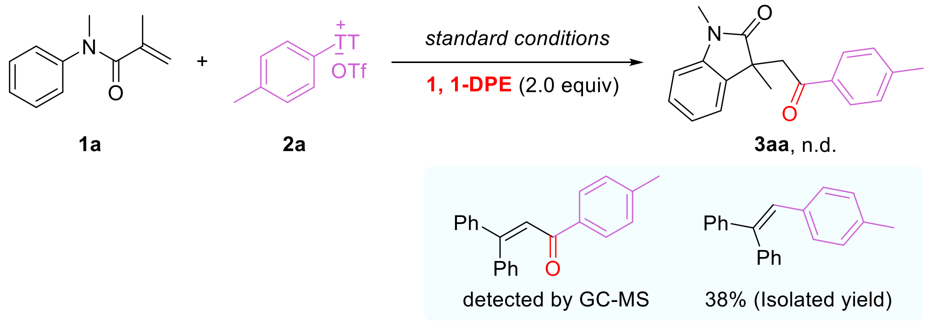

A 4 mL snap vial was charged with CuCl (10 mol%), (*S*)-DM-BINAP (10 mol%), thianthrenium Salts (1.5 equiv.), *N*-acrylamides (1.0 equiv.) and closed with a rubber-based septum. The vial was evacuated and backfilled with argon. Degassed EA (0.1 M), triisobutylamine (2.0 equiv.) and 1,1-DPE (2 equiv.) were added via syringe. The vial was then connected to atmosphere with a cannula and transferred into a 300 mL Parr 4560 series autoclave, under argon counterflow. The closed autoclave was flushed three times with nitrogen (~ 5 bar), three times with CO (~ 5 bar), and 40 bar of carbon monoxide (measured by pressure meter) was charged. The autoclave was then placed into an aluminum block on a magnetic stirrer. The reaction mixture was stirred (500 rpm) under blue-light irradiation at 60 °C for 24 h. And a proper amount of solvent was taken for GC analysis. The result is shown above.

ile :D:\MassHunter\GCMS\1\data\202510\zyh-539-2.D
perator :
cquired : 17 Oct 2025 12:16 using AcqMethod wfp-23 min.M
nstrument : GCMS
ample Name: zyh-539-2
isc Info :
ial Number: 112

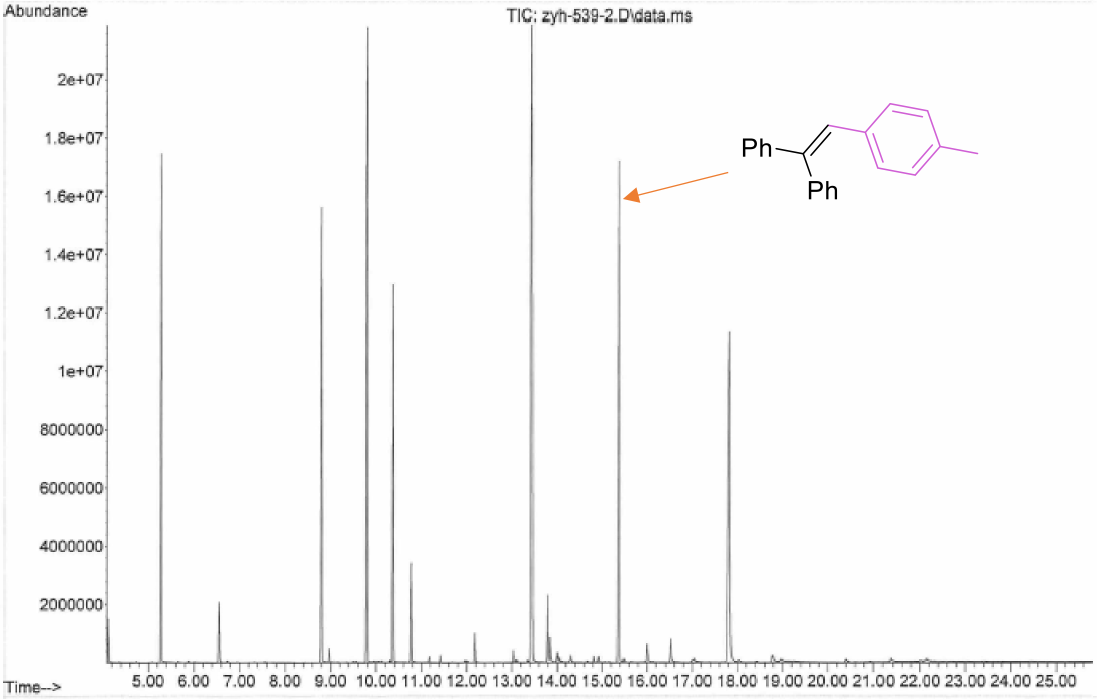

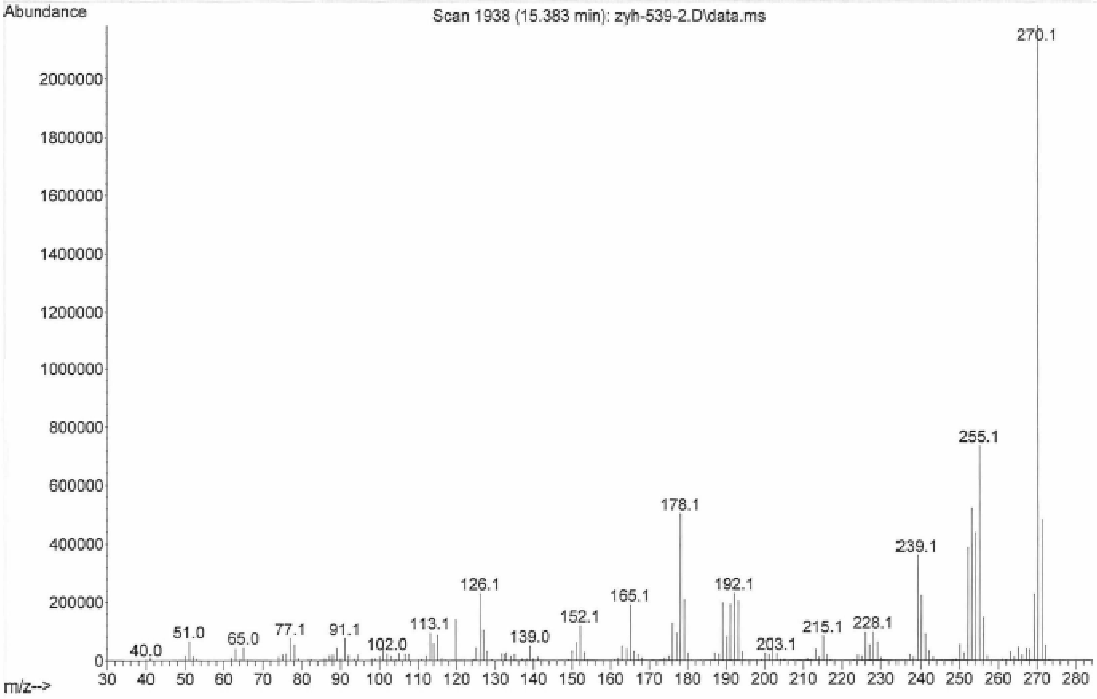

```
File        :D:\MassHunter\GCMS\1\data\202510\zyh-539-2.D
Operator    :
Acquired    : 17 Oct 2025   12:16     using AcqMethod wfp-23 min.M
Instrument  :    GCMS
Sample Name: zyh-539-2
Misc Info   :
Vial Number: 112
```

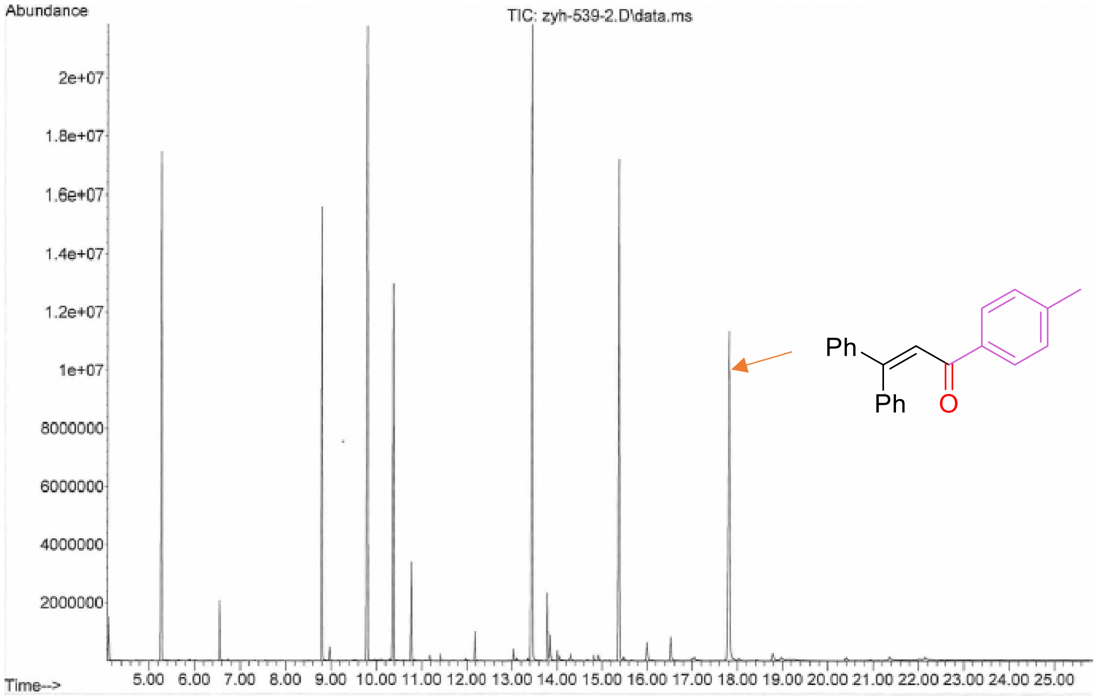

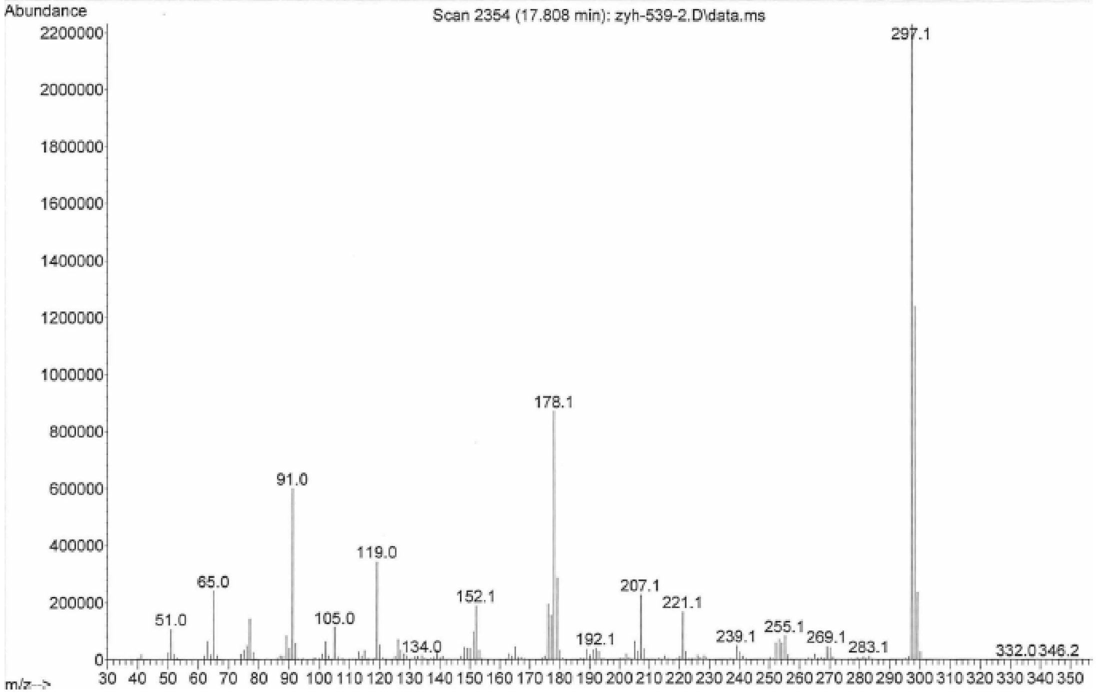

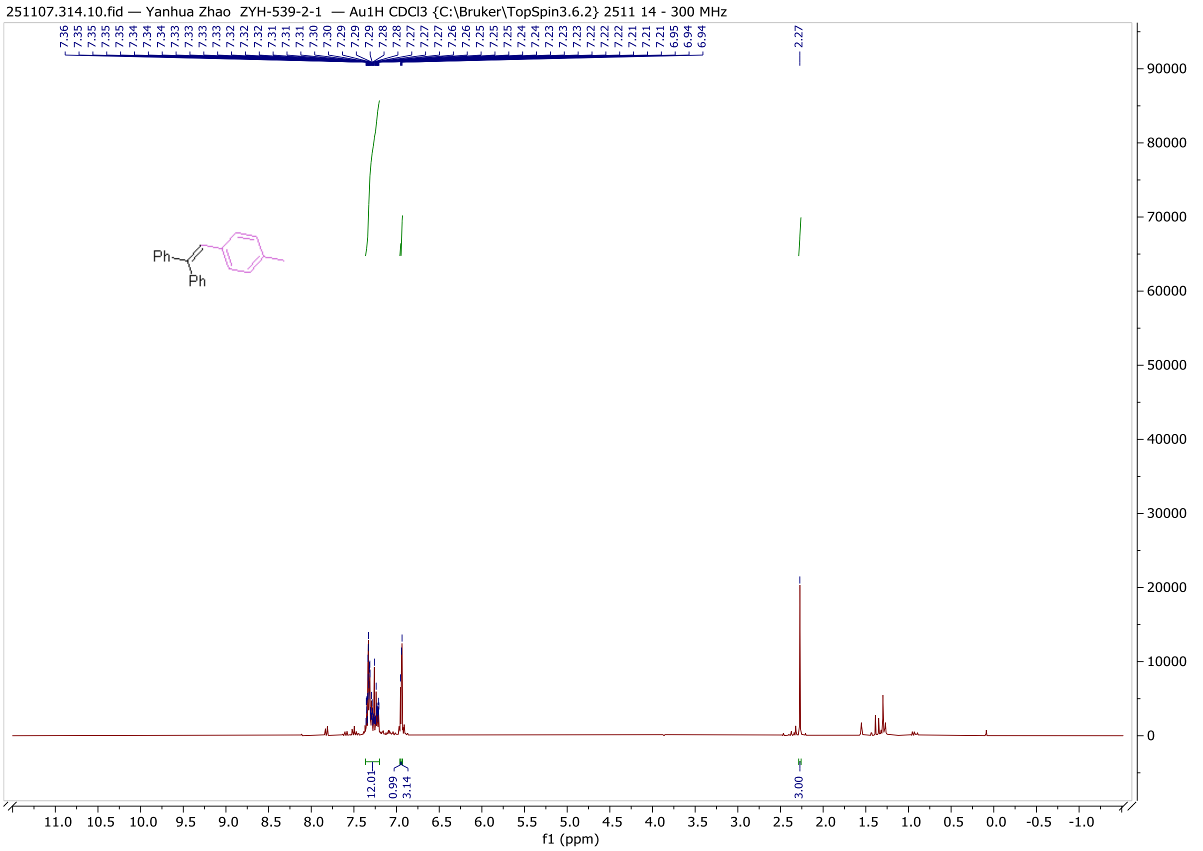
251107.314.10.fid — Yanhua Zhao ZYH-539-2-1 — Au1H CDCl3 {C:\Bruker\TopSpin3.6.2} 2511 14 - 300 MHz
Ph
Ph
f1 (ppm)

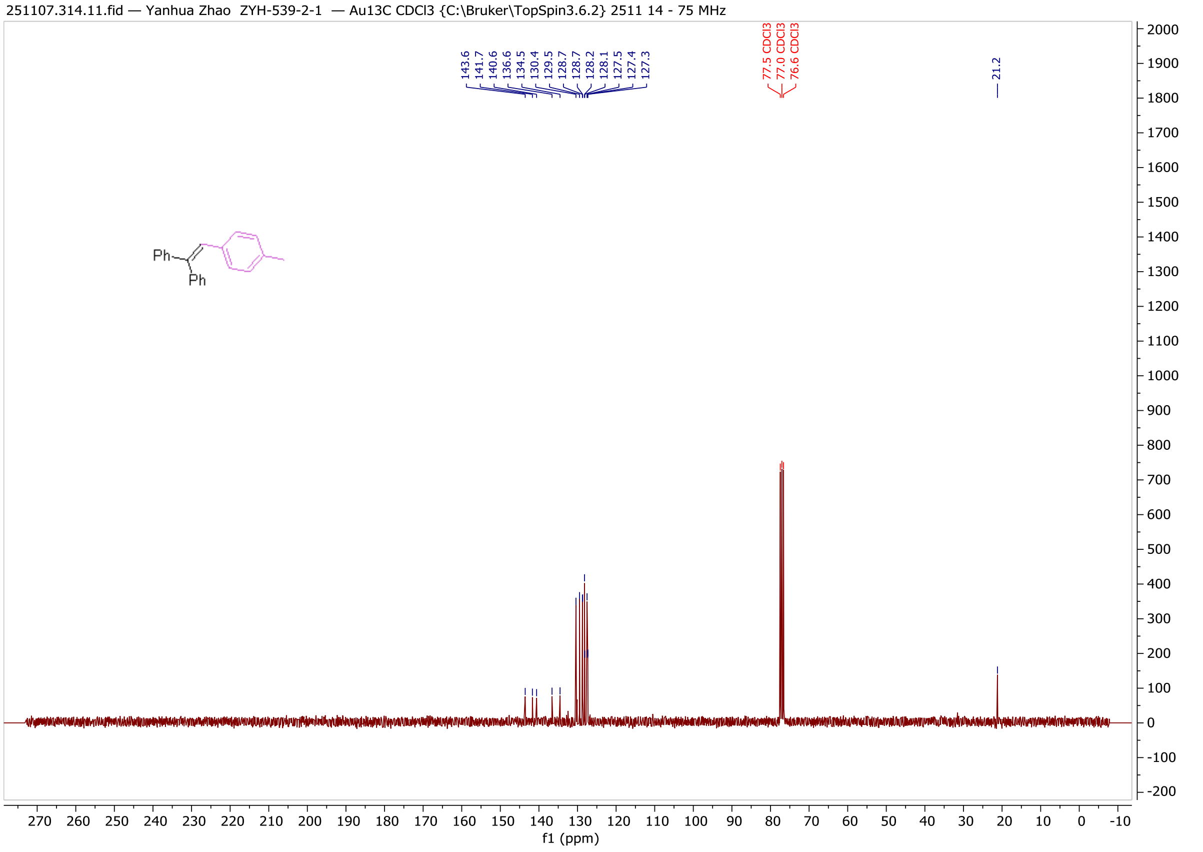
251107.314.11.fid — Yanhua Zhao ZYH-539-2-1 — Au13C CDCl3 {C:\Bruker\TopSpin3.6.2} 2511 14 - 75 MHz
143.6
141.7
140.6
136.6
134.5
130.4
129.5
128.7
128.7
128.2
128.1
127.5
127.4
127.3
77.5 CDCl3
77.0 CDCl3
76.6 CDCl3
21.2
Ph
Ph
f1 (ppm)

5.2 Light on/off experiment

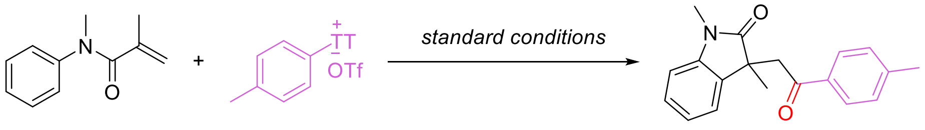

A 4 mL snap vial was charged with CuCl (10 mol%), (*S*)-DM-BINAP (10 mol%), thianthrenium salts (1.5 equiv.), *N*-acrylamides (1.0 equiv.) and closed with a rubber-based septum. The vial was evacuated and backfilled with argon. Degassed EA (0.1 M) and triisobutylamine (2.0 equiv.) were added via syringe. The vial was then connected to atmosphere with a cannula and transferred into a 300 mL Parr 4560 series autoclave, under argon counterflow. The closed autoclave was flushed three times with nitrogen (~ 5 bar), three times with CO (~ 5 bar), and 40 bar of carbon monoxide (measured by pressure meter) was charged. The autoclave was then placed into an aluminum block on a magnetic stirrer. The reaction mixture was stirred (500 rpm) under blue-light irradiation at 60 °C for 1 h. Then stirred under dark condition for 1.0 h, extracted 0.1 mL mixture again. Repeated these operations for 3 times and got 6 simples for GC yield. The result is shown above.

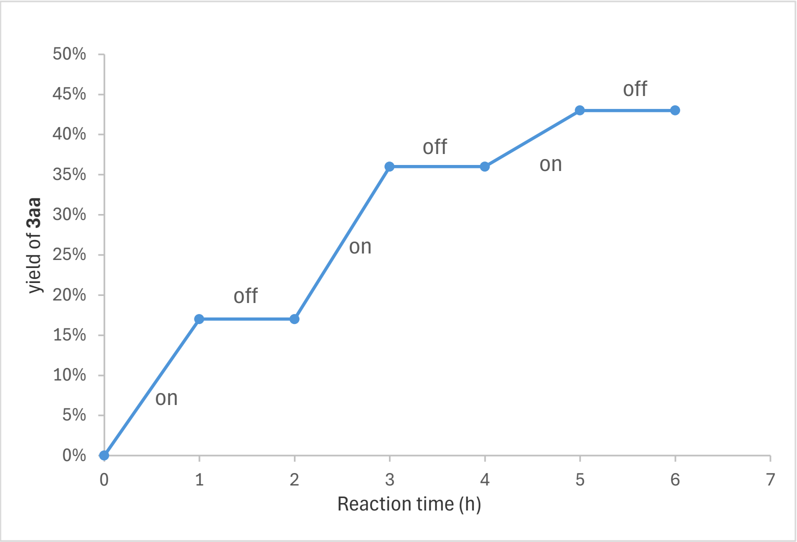

6. X-ray Crystal Analysis of 3aa and 5aa

Data were collected on a Bruker Kappa APEX II Duo diffractometer. The structure was solved by direct methods (SHELXS-97: Sheldrick, G. M. Acta Cryst. 2008, A64, 112.) and refined by full-matrix least-squares procedures on F 2 (SHELXL-2019: Sheldrick, G. M. Acta Cryst. 2015, C71, 3.). XP (Bruker AXS) was used for graphical representations.

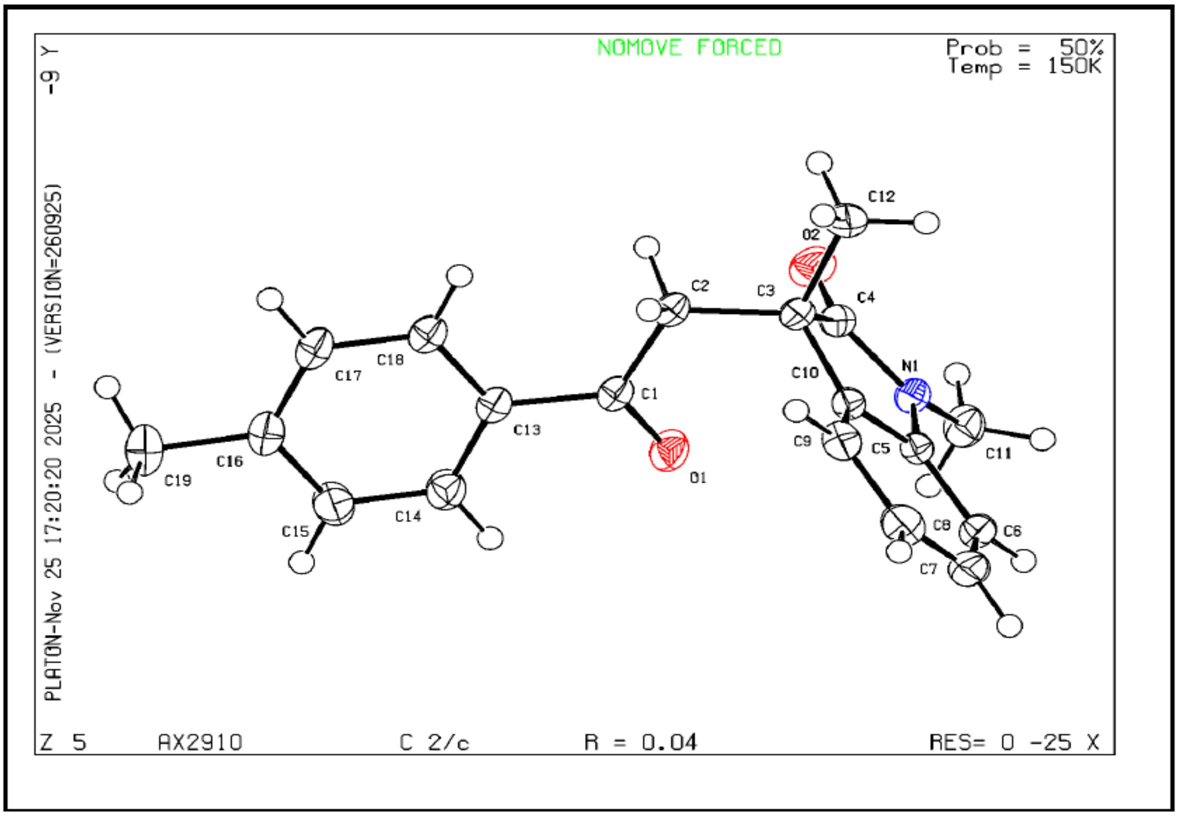

Bond precision: C-C = 0.0015 A Wavelength=0.71073

Cell: a=33.627(3) b=6.6196(5) c=14.8184(12)
alpha=90 beta=108.2773(13) gamma=90

Temperature: 150 K

	Calculated	Reported
Volume	3132.1(4)	3132.1(4)
Space group	C 2/c	C 2/c
Hall group	-C 2yc	-C 2yc
Moiety formula	C19 H19 N O2	?
Sum formula	C19 H19 N O2	C19 H19 N O2
Mr	293.35	293.35
Dx,g cm-3	1.244	1.244
Z	8	8
Mu (mm-1)	0.080	0.080
F000	1248.0	1248.0
F000'	1248.54	
h,k,lmax	46,9,20	46,9,20
Nref	4226	4223
Tmin,Tmax	0.972,0.980	0.970,0.980
Tmin'	0.972	

Correction method= # Reported T Limits: Tmin=0.970 Tmax=0.980
AbsCorr = MULTI-SCAN

Data completeness= 0.999 Theta(max)= 29.188

R(reflections)= 0.0415(3708) wR2(reflections)= 0.1170(4223)

S = 1.041 Npar= 202

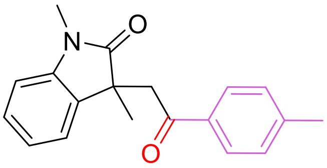

3aa

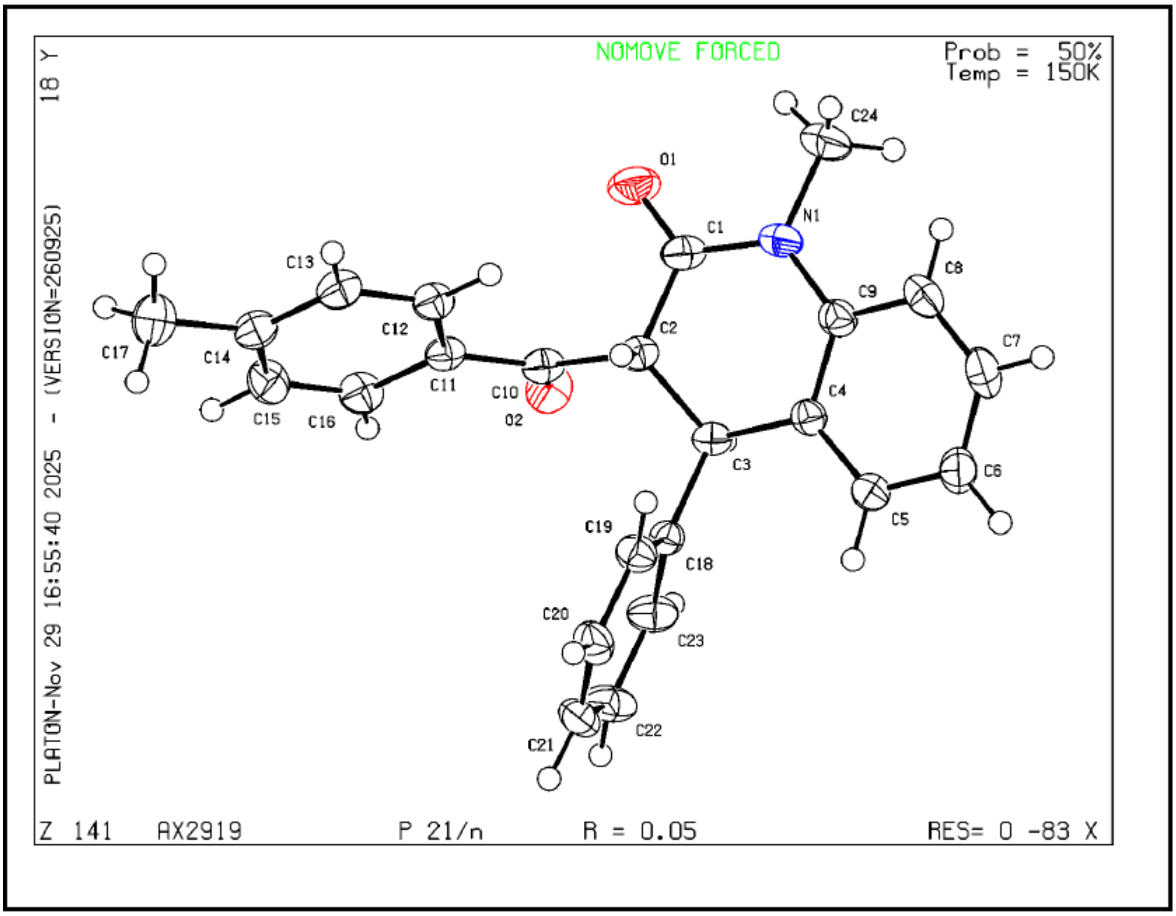

Bond precision: C-C = 0.0020 A Wavelength=0.71073

Cell: a=9.3703(8) b=5.6079(4) c=35.546(3)
alpha=90 beta=94.3917(15) gamma=90

Temperature: 150 K

	Calculated	Reported
Volume	1862.4(3)	1862.4(3)
Space group	P 21/n	P 21/n
Hall group	-P 2yn	-P 2yn
Moiety formula	C24 H21 N O2	?
Sum formula	C24 H21 N O2	C24 H21 N O2
Mr	355.42	355.42
Dx,g cm-3	1.268	1.268
Z	4	4
Mu (mm-1)	0.080	0.080
F000	752.0	752.0
F000'	752.32	
h,k,lmax	12,7,46	12,7,46
Nref	4515	4510
Tmin,Tmax	0.981,0.997	0.970,0.990
Tmin'	0.966	

Correction method= # Reported T Limits: Tmin=0.970 Tmax=0.990
AbsCorr = MULTI-SCAN

Data completeness= 0.999 Theta(max)= 28.000

R(reflections)= 0.0450(3695) wR2(reflections)= 0.1167(4510)

S = 1.056 Npar= 246

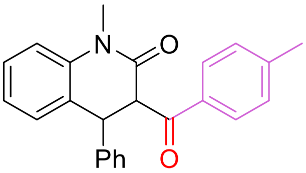

5aa

7. Reference

1. C.-C. Li and S.-D. Yang, Oxidant-Free Rhodium(I)-Catalyzed Difunctionalization of Acrylamide: An Efficient Approach To Synthesize Oxindoles, *Org. Lett.*, 2015, **17**, 2142–2145.
2. L. Liu, H. Lu, H. Wang, C. Yang, X. Zhang, D. Zhang-Negrerie, Y. Du and K. Zhao, PhI($OCOCF_3$)$_2$-Mediated C–C Bond Formation Concomitant with a 1,2-Aryl Shift in a Metal-Free Synthesis of 3-Arylquinolin-2-ones, *Org. Lett.*, 2013, **15**, 2906–2909.
3. X.-W. Gu, Y.-H. Zhao and X.-F. Wu, [3 + 2] Cycloaddition of azides with arynes formed via C–H deprotonation of aryl sulfonium salts, *Green Chem.*, 2023, **25**, 6282–6286.
4. R. A. Roberts, B. E. Metze, A. Nilova and D. R. Stuart, Synthesis of Arynes via Formal Dehydrogenation of Arenes, *J. Am. Chem. Soc.*, 2023, **145**, 3306–3311.
5. M.-B. Zhou, R.-J. Song, X.-H. Ouyang, Y. Liu, W.-T. Wei, G.-B. Deng and J.-H. Li, Metal-free oxidative tandem coupling of activated alkenes with carbonyl C(sp^2)–H bonds and aryl C(sp^2)–H bonds using TBHP, *Chem. Sci.*, 2013, **4**, 2690–2694.
6. P. Biswas, S. Mandal and J. Guin, Aerobic Acylarylation of *α,β*-Unsaturated Amides with Aldehydes, *Org. Lett.*, 2020, **22**, 4294–4299.

8. NMR Spectra of Products

251009.308.10.fid — Yanhua Zhao ZYH-538-1 — Au1H CDCl3 {C:\Bruker\TopSpin3.6.2} 2510 8 - 300 MHz

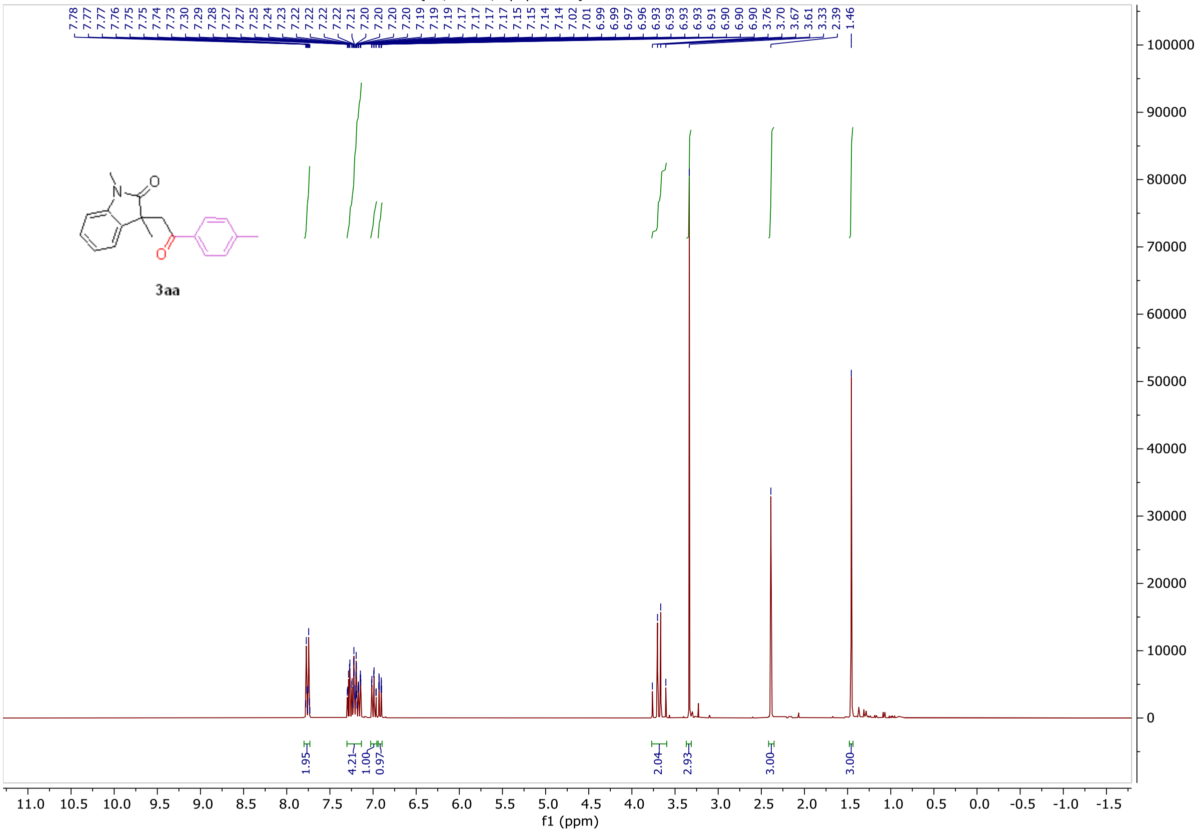

251009.308.11.fid — Yanhua Zhao ZYH-538-1 — Au13C CDCl3 {C:\Bruker\TopSpin3.6.2} 2510 8 - 75 MHz

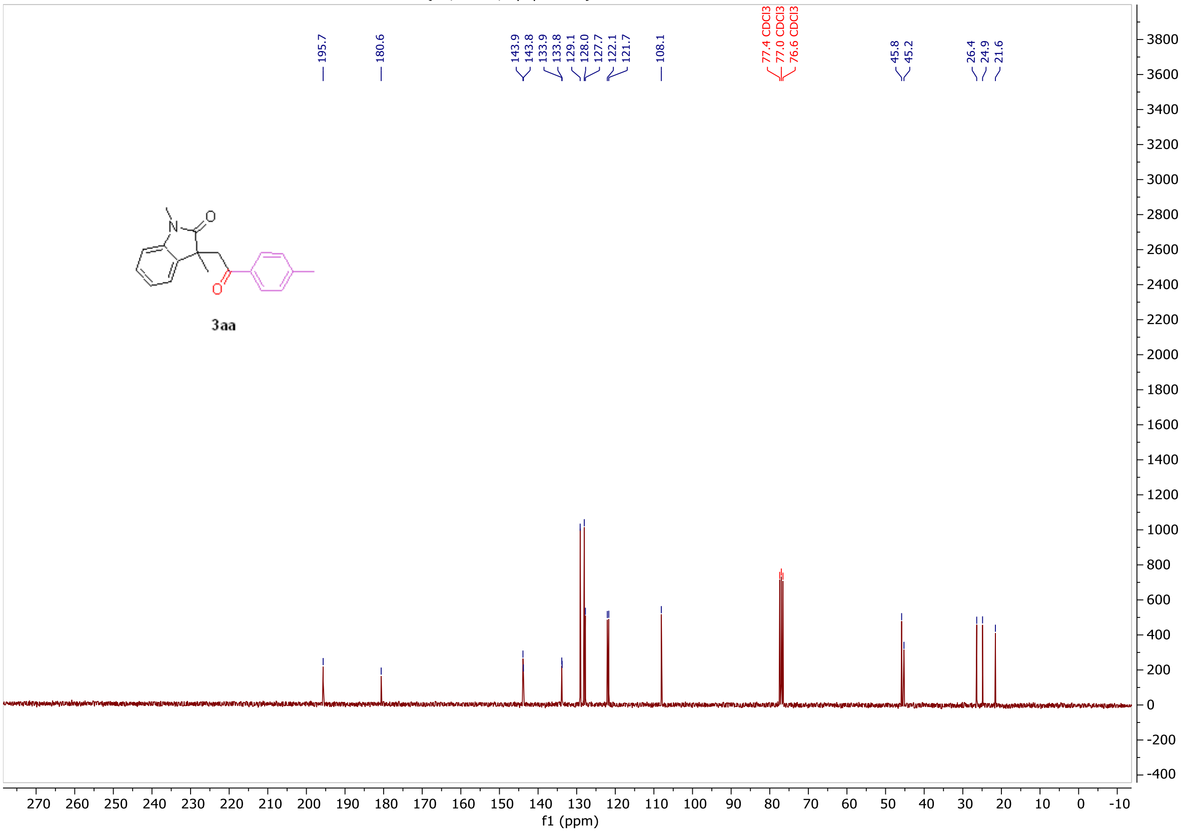

251009.309.10.fid — Yanhua Zhao ZYH-538-2 — Au1H CDCl3 {C:\Bruker\TopSpin3.6.2} 2510 9 - 300 MHz

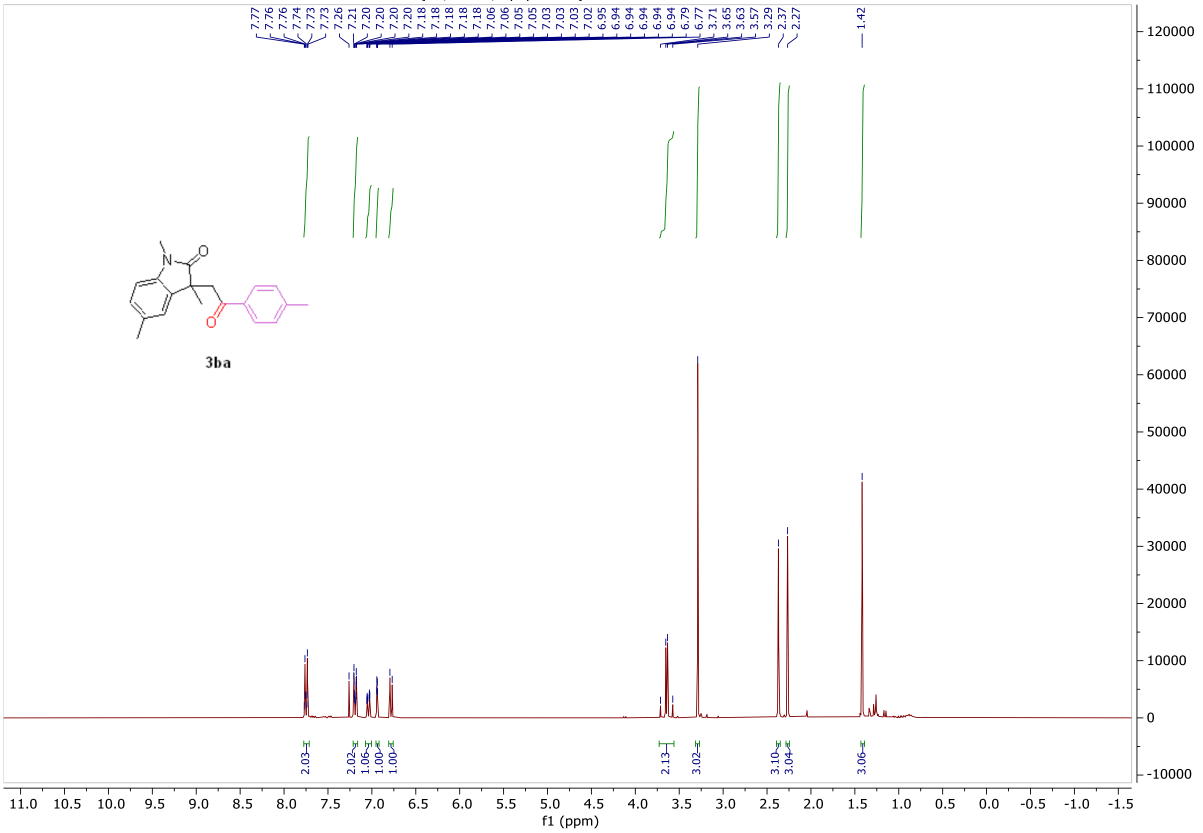

251009.309.11.fid — Yanhua Zhao ZYH-538-2 — Au13C CDCl3 {C:\Bruker\TopSpin3.6.2} 2510 9 - 75 MHz

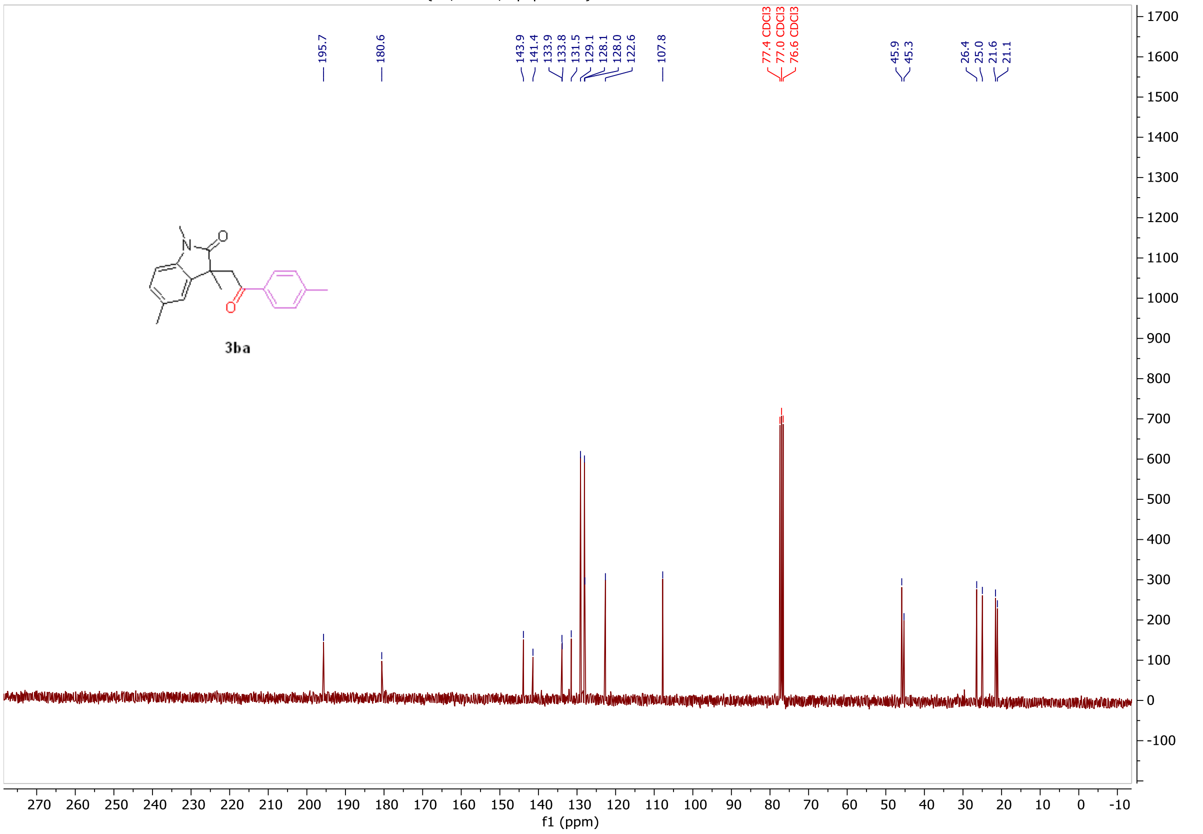

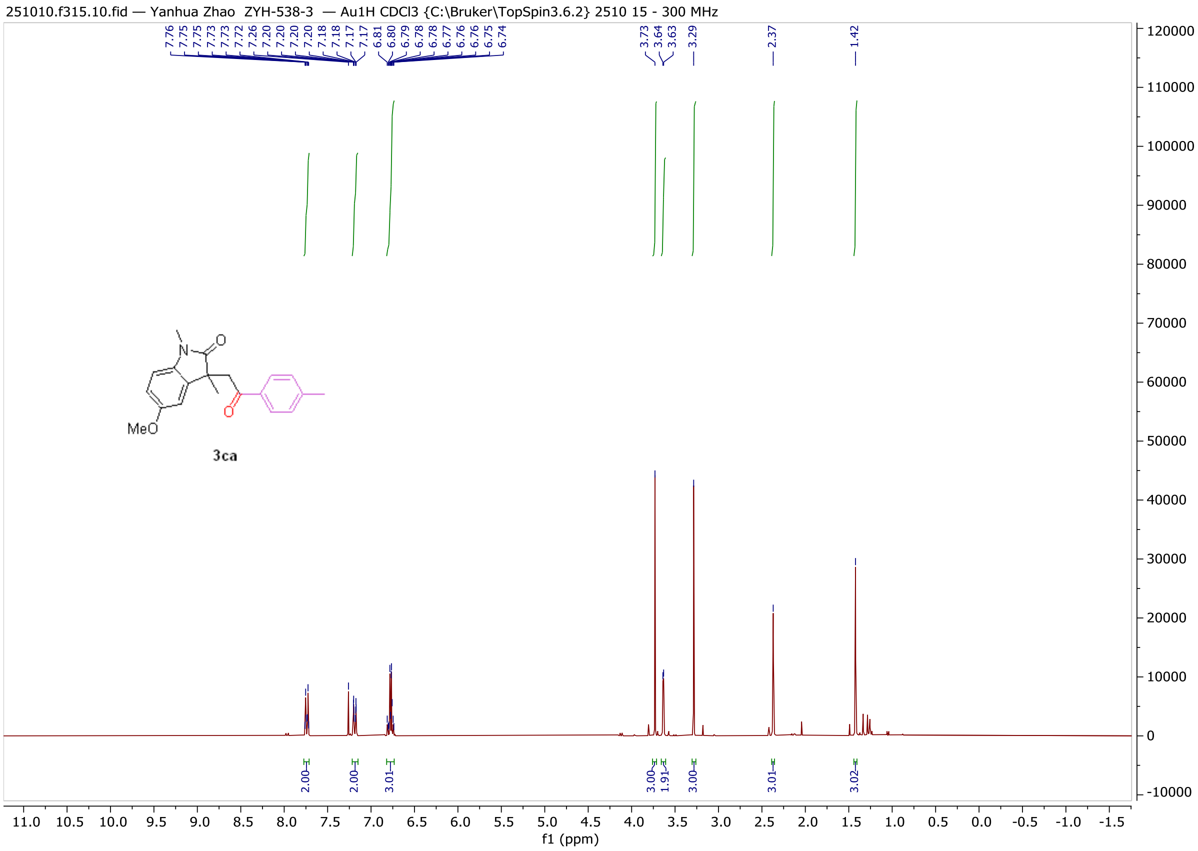

251010.f315.10.fid — Yanhua Zhao ZYH-538-3 — Au1H CDCl3 {C:\Bruker\TopSpin3.6.2} 2510 15 - 300 MHz
7.76 7.75 7.75 7.73 7.73 7.72 7.26 7.20 7.20 7.20 7.20 7.18 7.17 7.17 6.81 6.80 6.79 6.78 6.78 6.77 6.76 6.76 6.75 6.74
3.73 3.64 3.63 3.29 2.37 1.42
MeO
3ca
2.00 2.00 3.01 3.00 1.91 3.00 3.01 3.02
f1 (ppm)

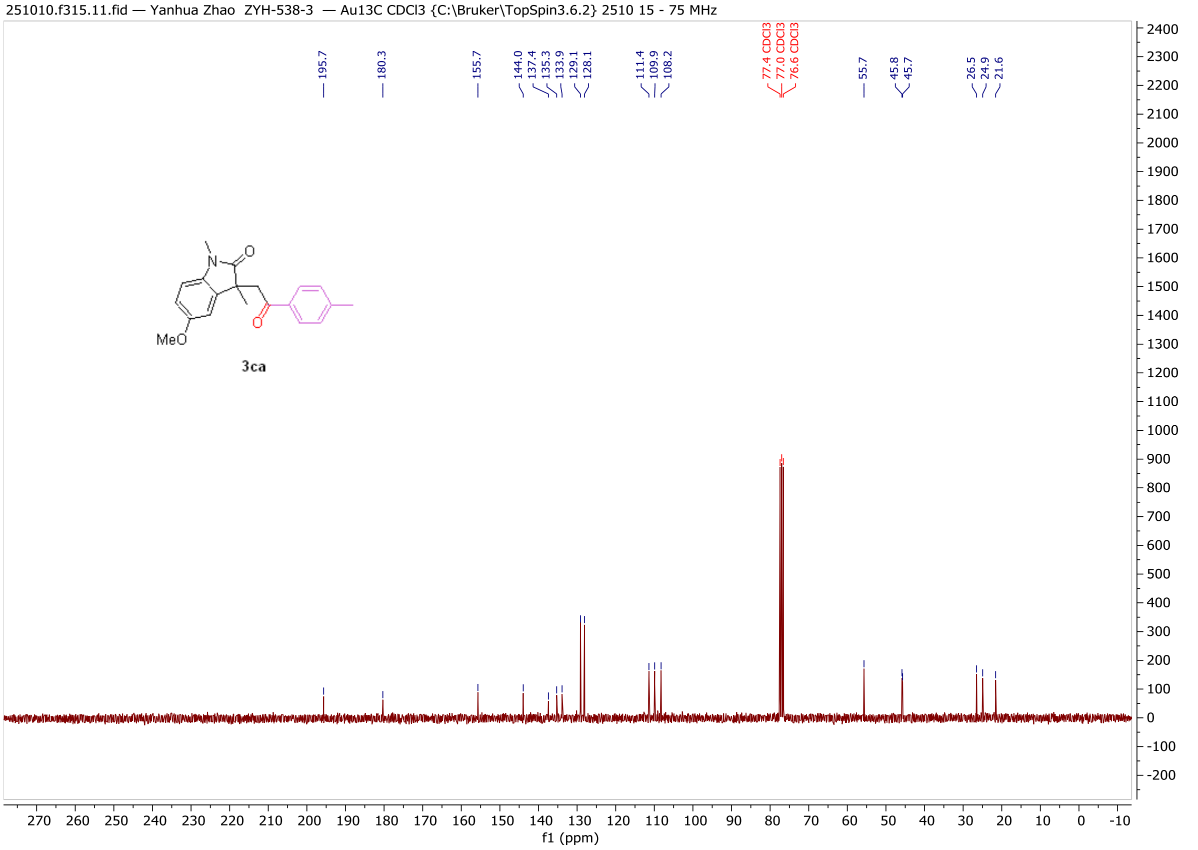

251010.f315.11.fid — Yanhua Zhao ZYH-538-3 — Au13C CDCl3 {C:\Bruker\TopSpin3.6.2} 2510 15 - 75 MHz
195.7 180.3 155.7 144.0 137.4 135.3 133.9 129.1 128.1 111.4 109.9 108.2
77.4 CDCl3 77.0 CDCl3 76.6 CDCl3
55.7 45.8 45.7 26.5 24.9 21.6
MeO
3ca
f1 (ppm)

251010.f316.10.fid — Yanhua Zhao ZYH-538-4 — Au1H CDCl3 {C:\Bruker\TopSpin3.6.2} 2510 16 - 300 MHz

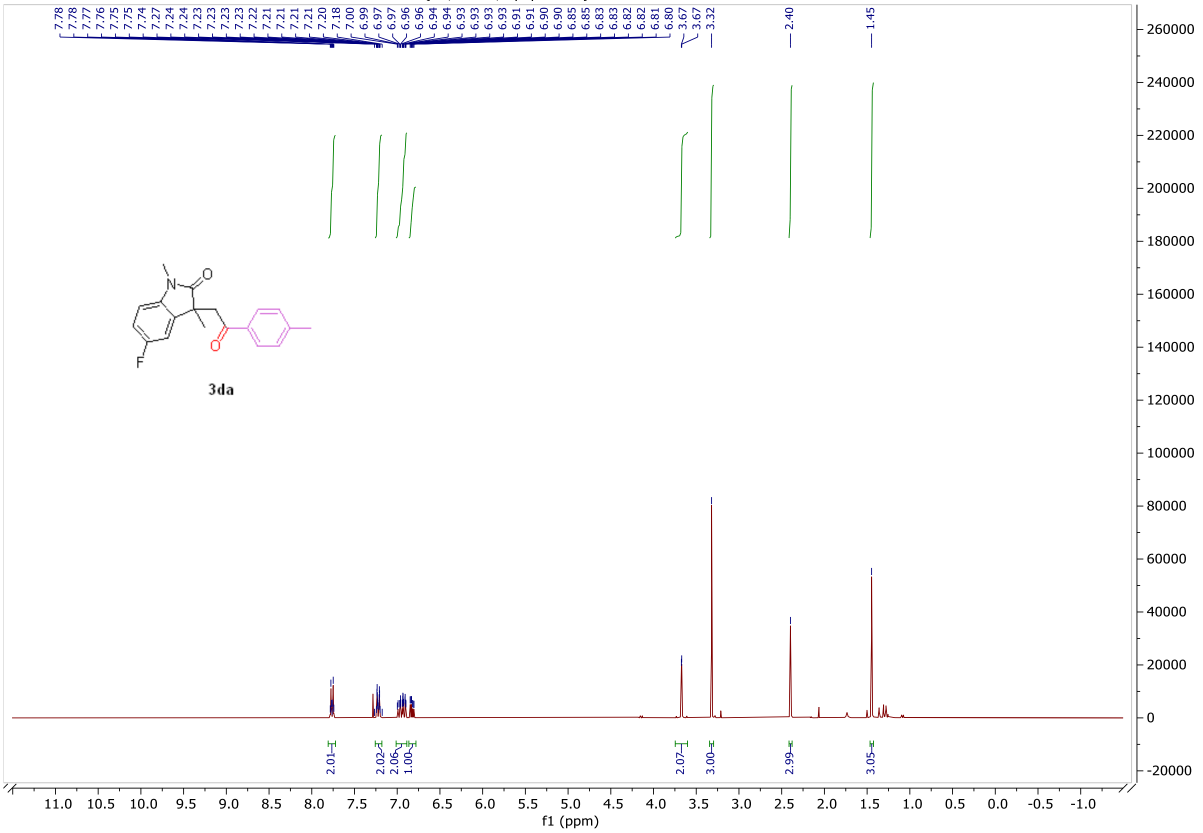

251010.f316.11.fid — Yanhua Zhao ZYH-538-4 — Au13C CDCl3 {C:\Bruker\TopSpin3.6.2} 2510 16 - 75 MHz

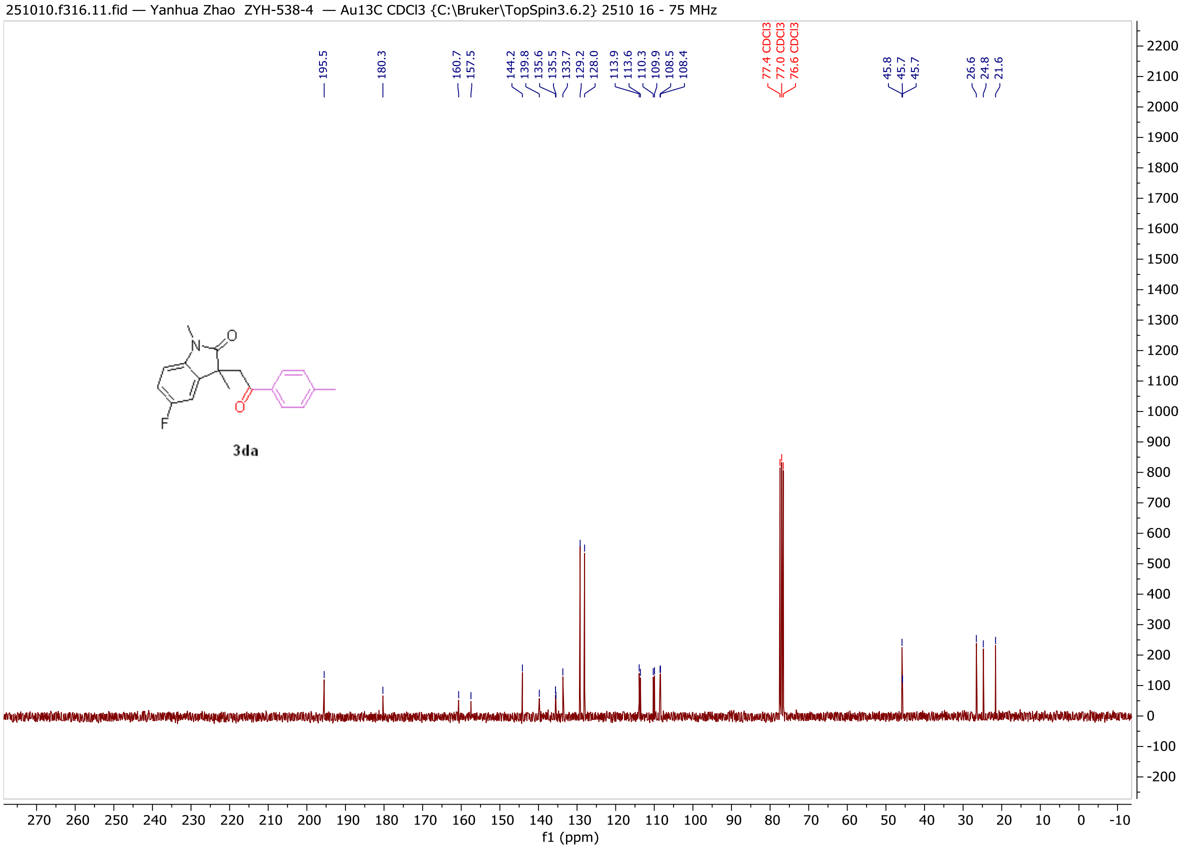

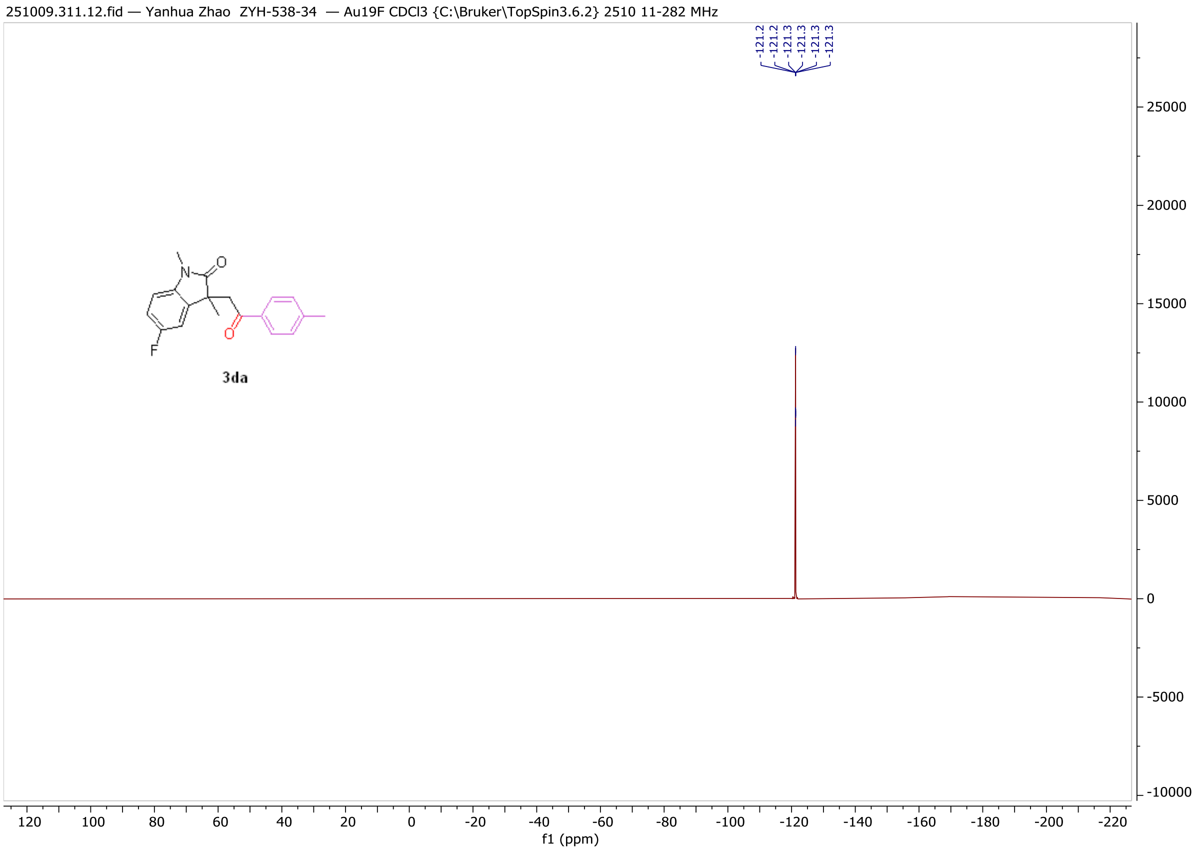
251009.311.12.fid — Yanhua Zhao ZYH-538-34 — Au19F CDCl3 {C:\Bruker\TopSpin3.6.2} 2510 11-282 MHz
-121.2
-121.2
-121.3
-121.3
-121.3
-121.3
3da
25000
20000
15000
10000
5000
0
-5000
-10000
120
100
80
60
40
20
0
-20
-40
-60
-80
-100
-120
-140
-160
-180
-200
-220
f1 (ppm)

251009.321.10.fid — Yanhua Zhao, ZYH-538-5 — Au1H CDCl3 {C:\Bruker\TopSpin3.6.2} 2510 21 - 300 MHz

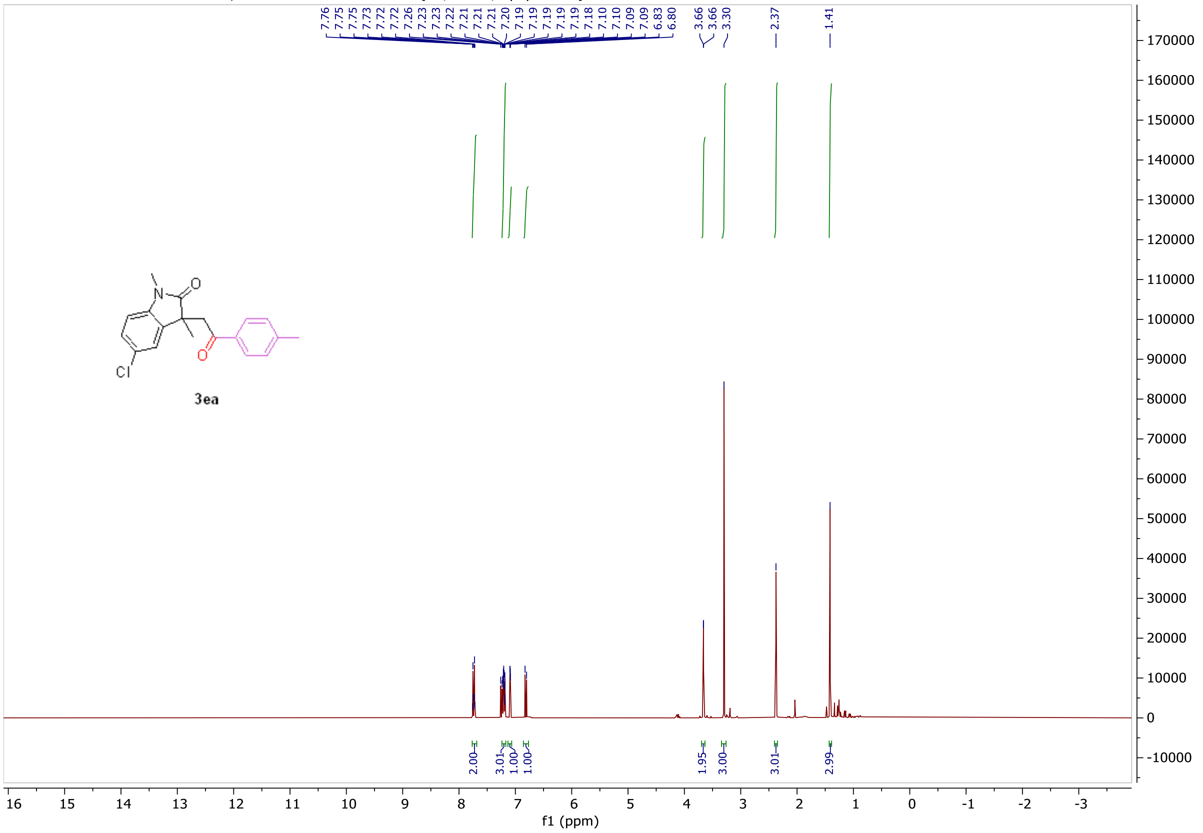

3ea

251009.321.11.fid — Yanhua Zhao, ZYH-538-5 — Au13C CDCl3 {C:\Bruker\TopSpin3.6.2} 2510 21 - 75 MHz

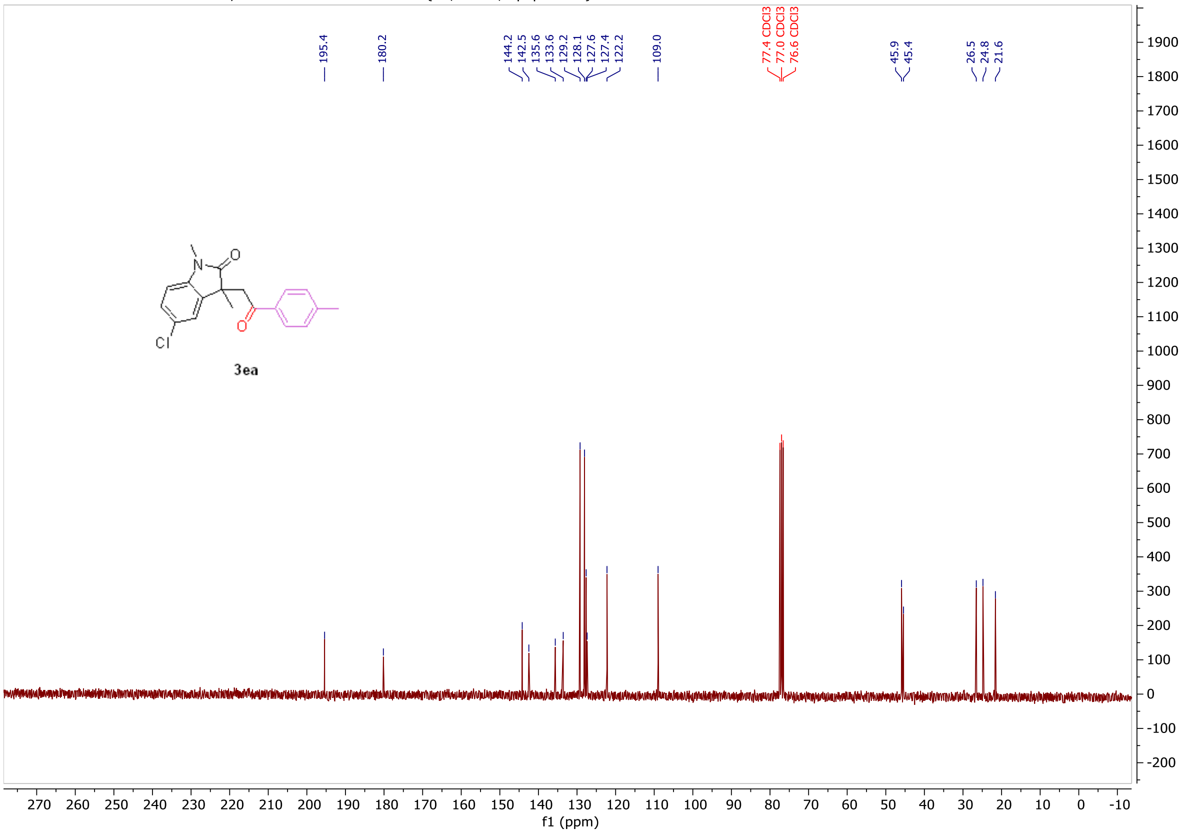

3ea

251009.322.10.fid — Yanhua Zhao, ZYH-538-6 — Au1H CDCl3 {C:\Bruker\TopSpin3.6.2} 2510 22 - 300 MHz

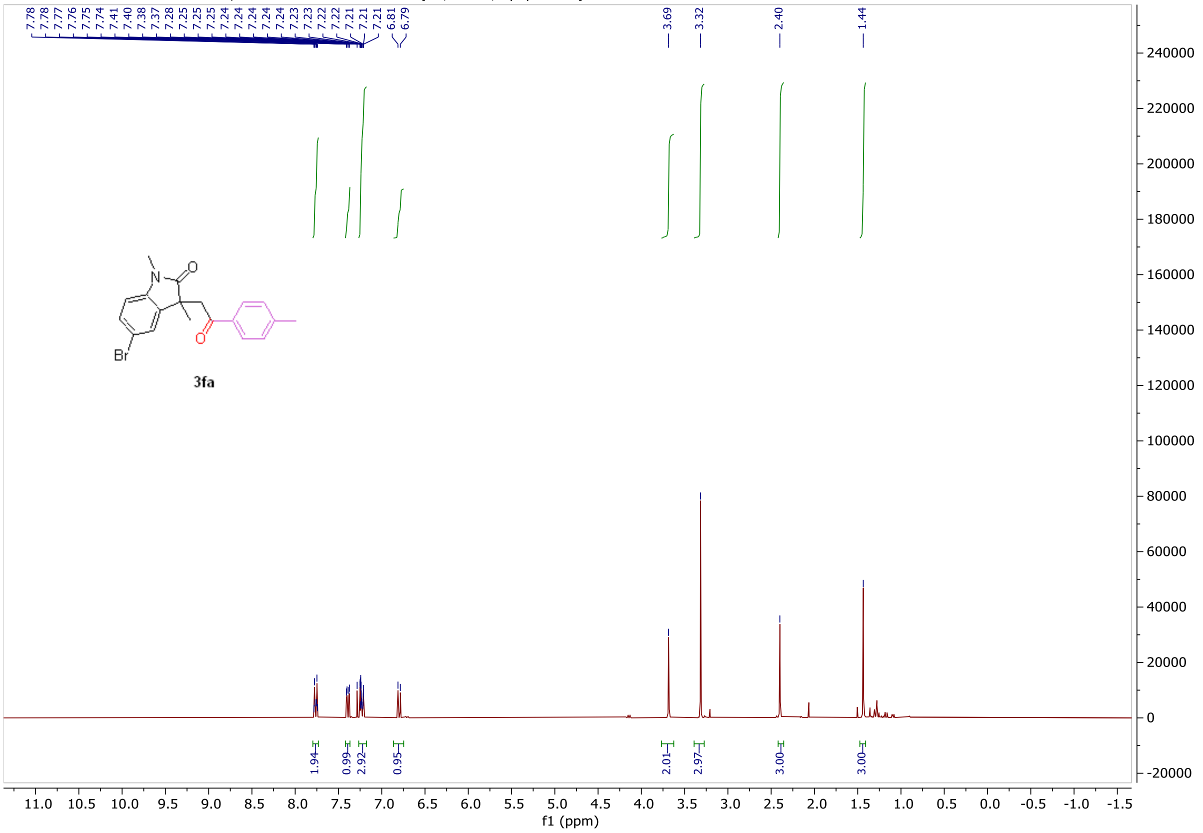

251009.322.11.fid — Yanhua Zhao, ZYH-538-6 — Au13C CDCl3 {C:\Bruker\TopSpin3.6.2} 2510 22 - 75 MHz

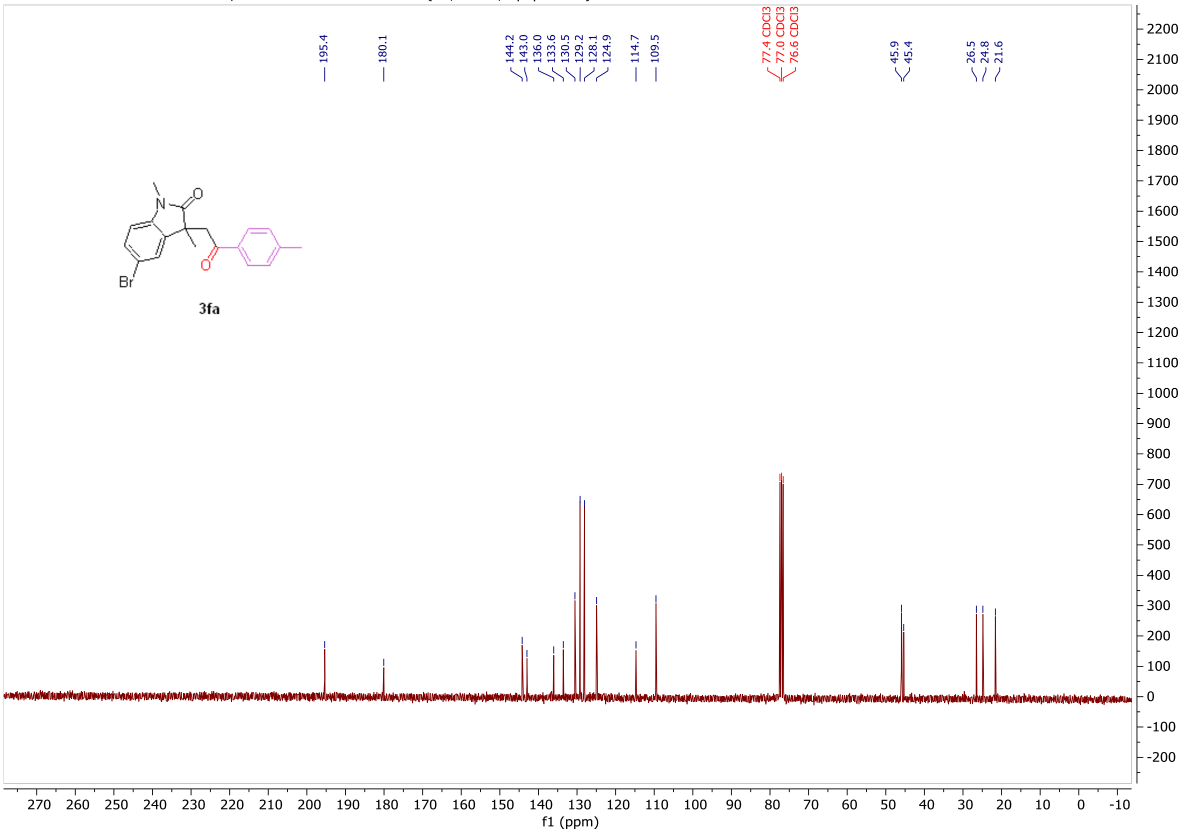

251104.f312.10.fid — Yanhua Zhao ZYH-538-7 — Au1H CDCl3 {C:\Bruker\TopSpin3.6.2} 2511 12 - 300 MHz

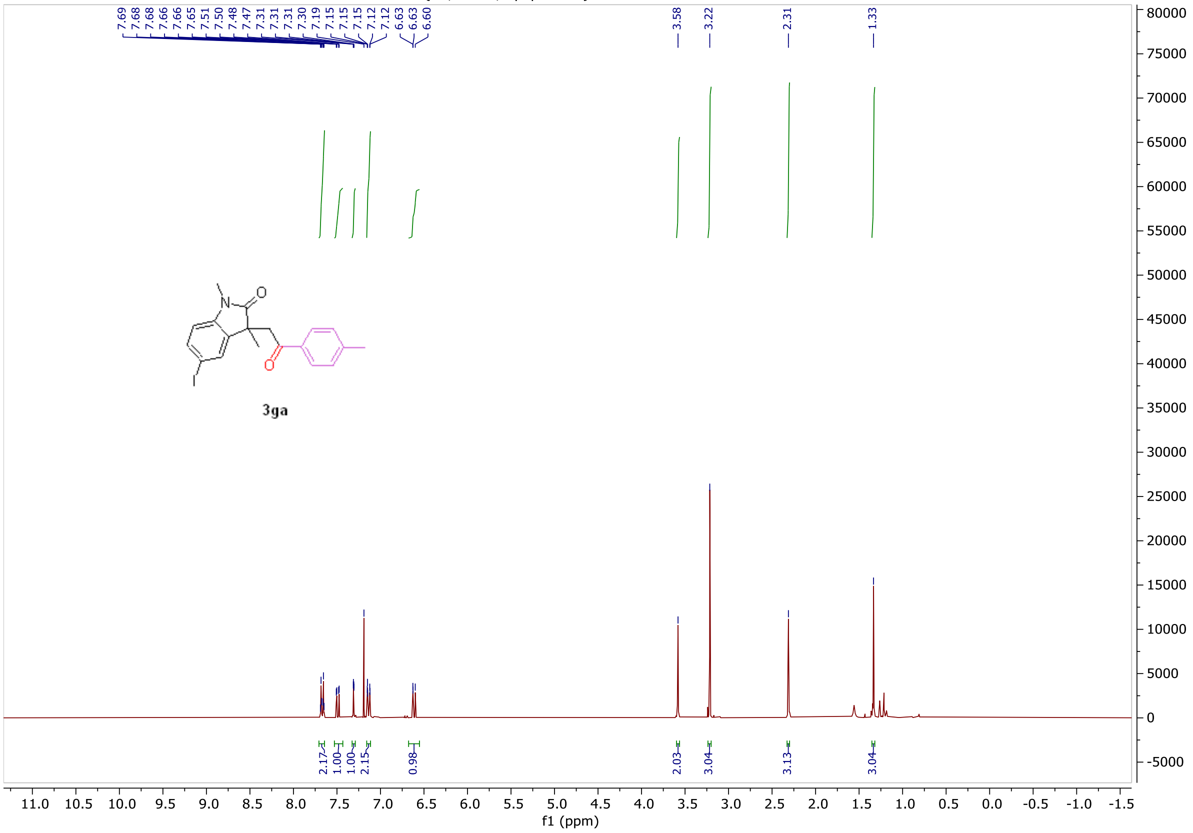

251009.323.11.fid — Yanhua Zhao, ZYH-538-7 — Au13C CDCl3 {C:\Bruker\TopSpin3.6.2} 2510 23 - 75 MHz

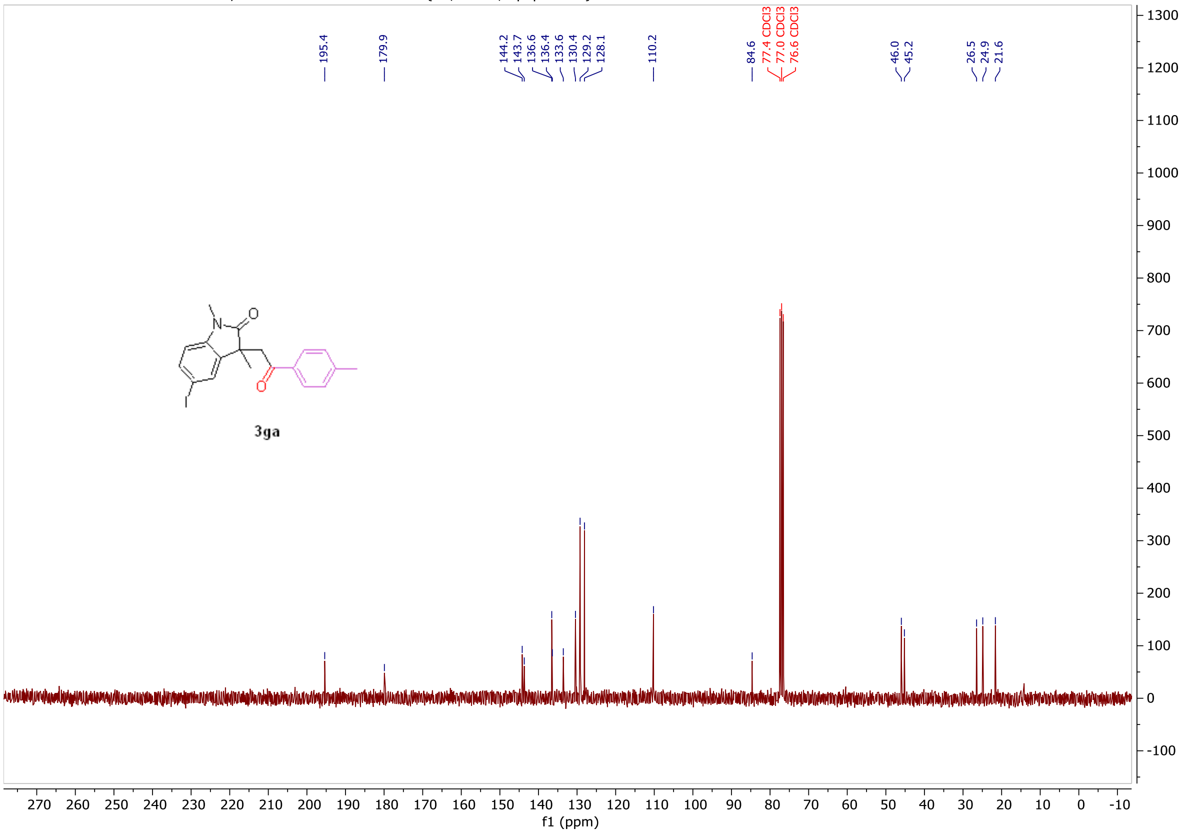

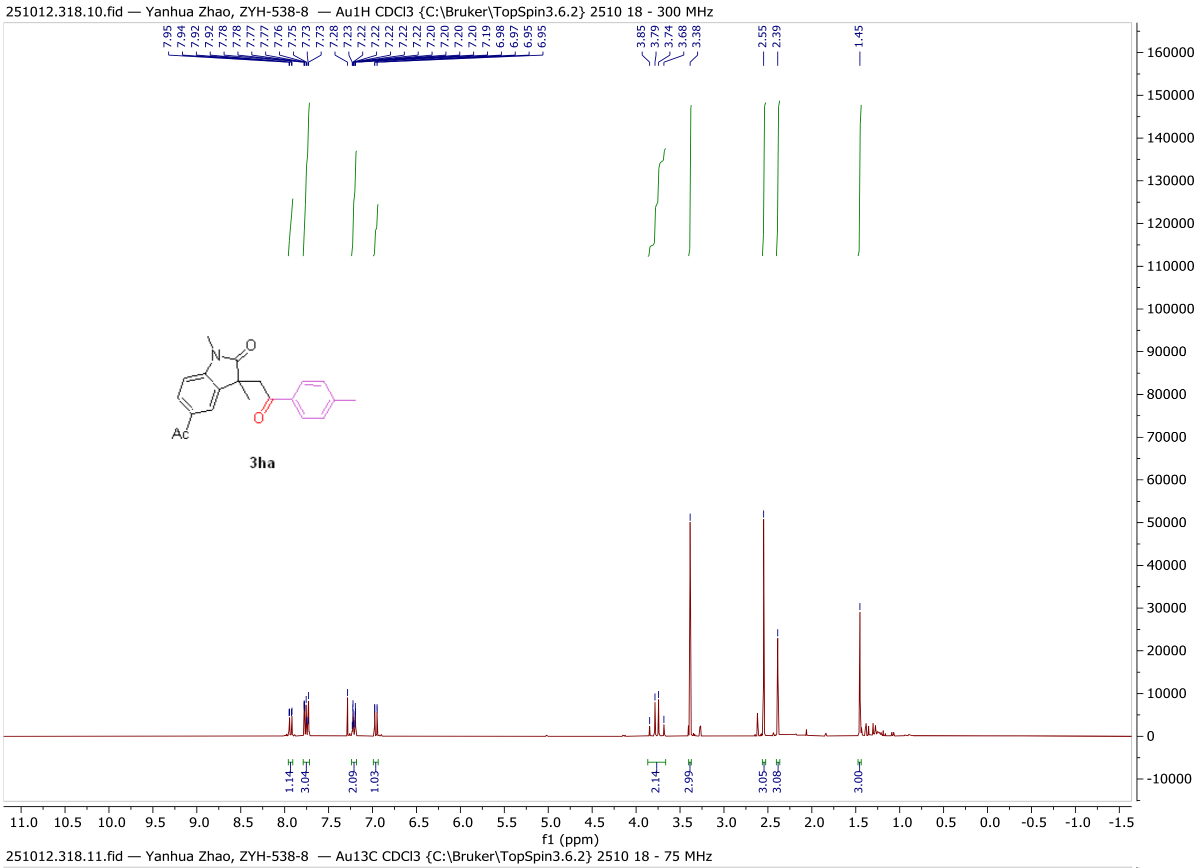
251012.318.10.fid — Yanhua Zhao, ZYH-538-8 — Au1H CDCl3 {C:\Bruker\TopSpin3.6.2} 2510 18 - 300 MHz
7.95 7.94 7.92 7.92 7.78 7.78 7.77 7.76 7.75 7.73 7.73 7.28 7.23 7.22 7.22 7.22 7.22 7.20 7.20 7.20 7.20 7.19 6.98 6.97 6.95 6.95
3.85 3.79 3.74 3.68 3.38
2.55 2.39
1.45
1.14 3.04 2.09 1.03 2.14 2.99 3.05 3.08 3.00
N
O
O
Ac
3ha
f1 (ppm)

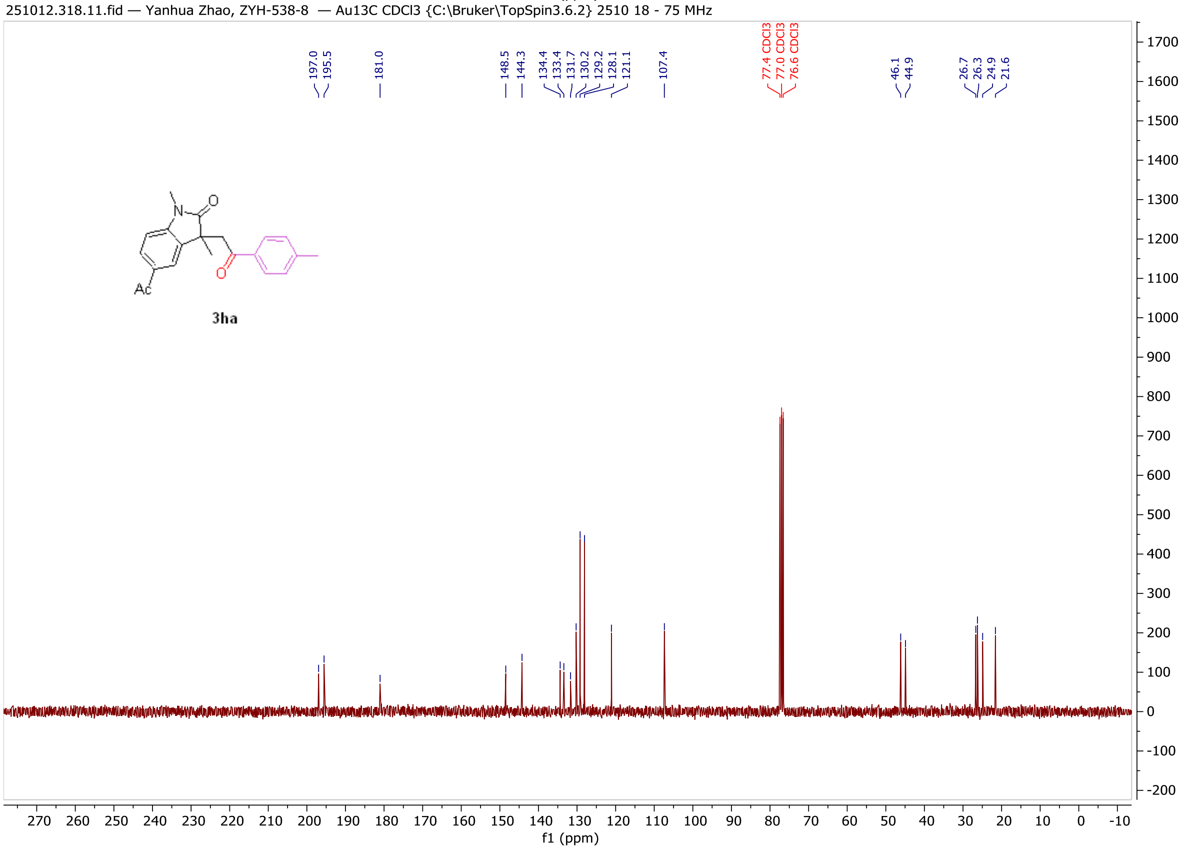
251012.318.11.fid — Yanhua Zhao, ZYH-538-8 — Au13C CDCl3 {C:\Bruker\TopSpin3.6.2} 2510 18 - 75 MHz
197.0 195.5
181.0
148.5 144.3 134.4 133.4 131.7 130.2 129.2 128.1 121.1
107.4
77.4 CDCl3 77.0 CDCl3 76.6 CDCl3
46.1 44.9
26.7 26.3 24.9 21.6
N
O
O
Ac
3ha
f1 (ppm)

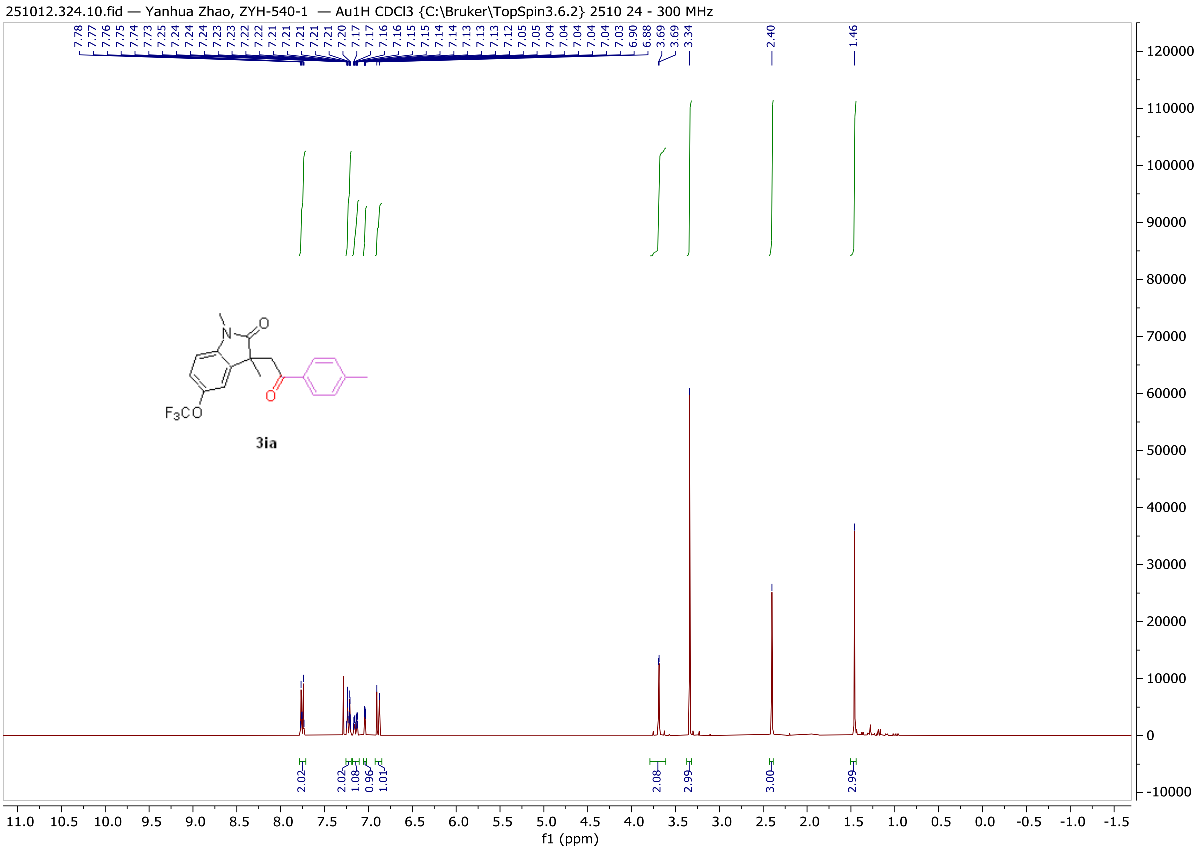
251012.324.10.fid — Yanhua Zhao, ZYH-540-1 — Au1H CDCl3 {C:\Bruker\TopSpin3.6.2} 2510 24 - 300 MHz
3ia
f1 (ppm)

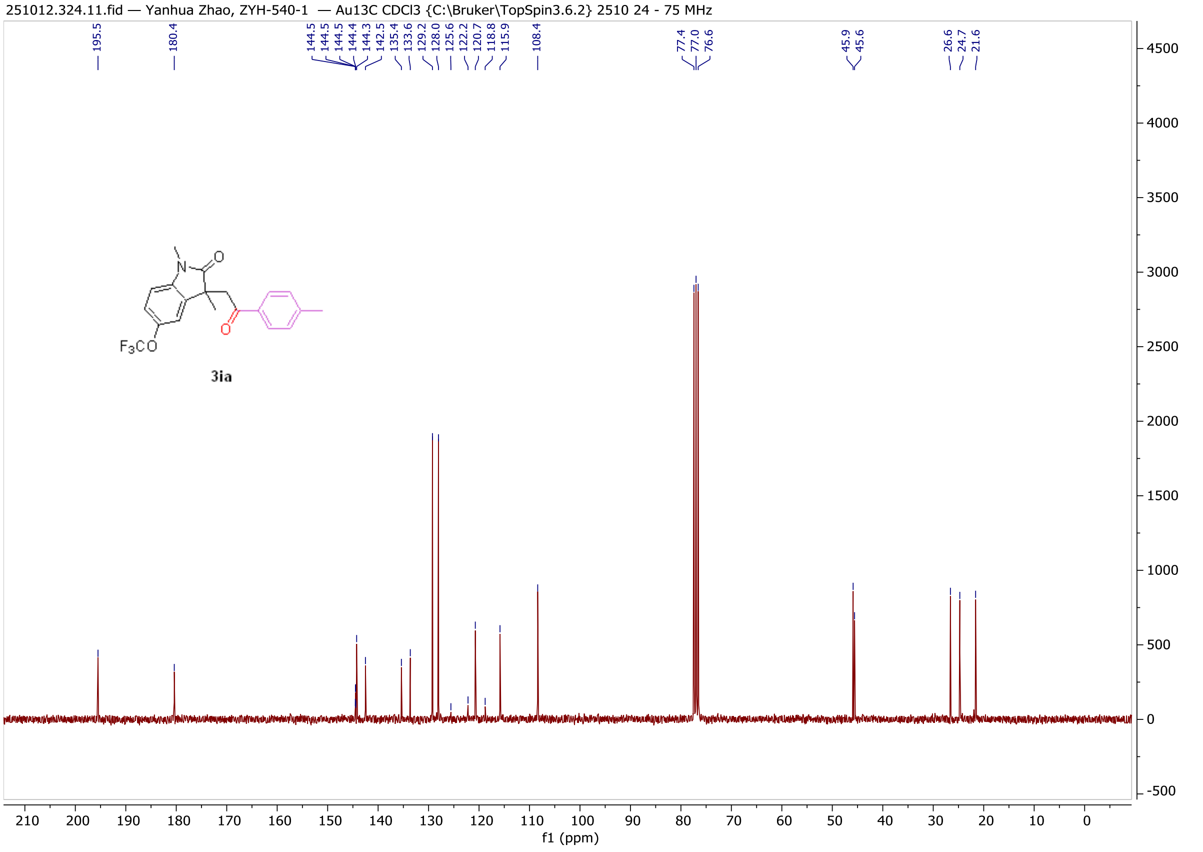
251012.324.11.fid — Yanhua Zhao, ZYH-540-1 — Au13C CDCl3 {C:\Bruker\TopSpin3.6.2} 2510 24 - 75 MHz
3ia
f1 (ppm)

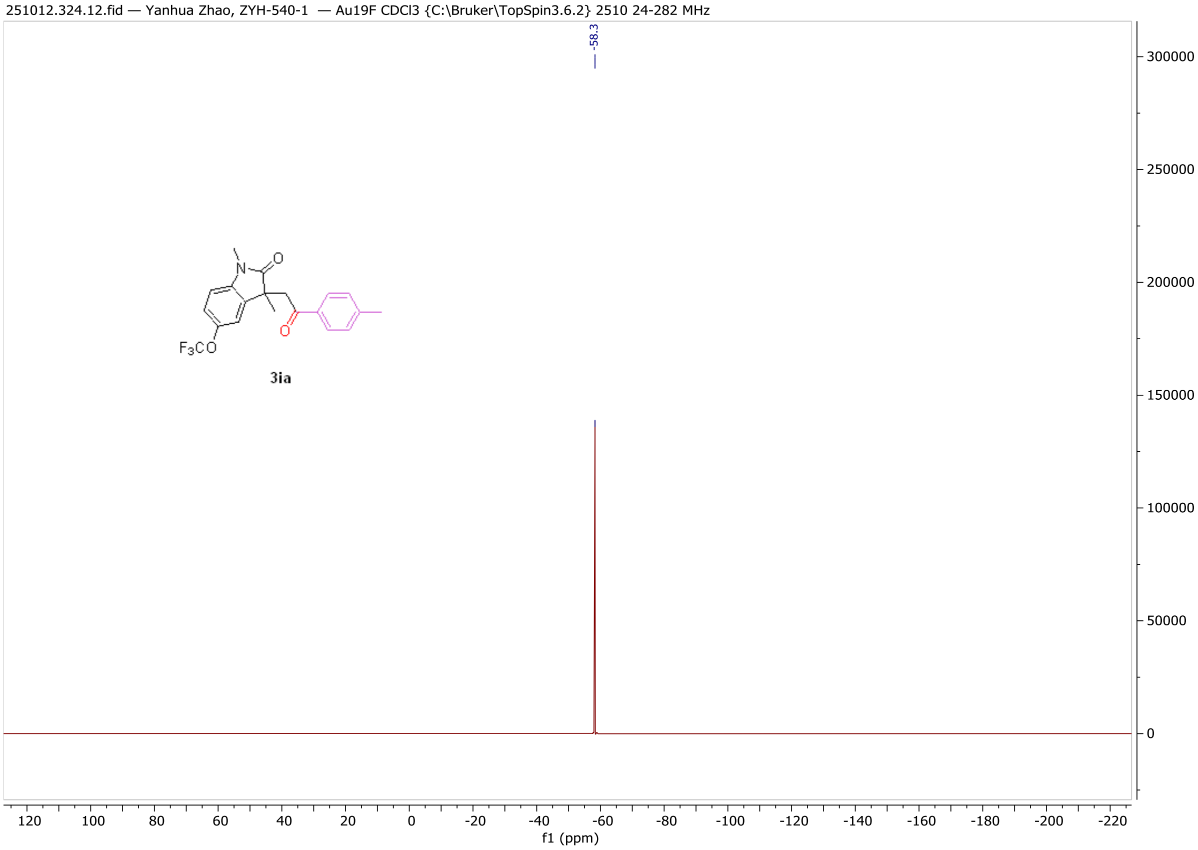
251012.324.12.fid — Yanhua Zhao, ZYH-540-1 — Au19F CDCl3 {C:\Bruker\TopSpin3.6.2} 2510 24-282 MHz
-58.3
F3CO
N
O
3ia
300000
250000
200000
150000
100000
50000
0
120
100
80
60
40
20
0
-20
-40
-60
-80
-100
-120
-140
-160
-180
-200
-220
f1 (ppm)

251014.301.10.fid — Yanhua Zhao ZYH-540-3 — Au1H CDCl3 {C:\Bruker\TopSpin3.6.2} 2510 1 - 300 MHz

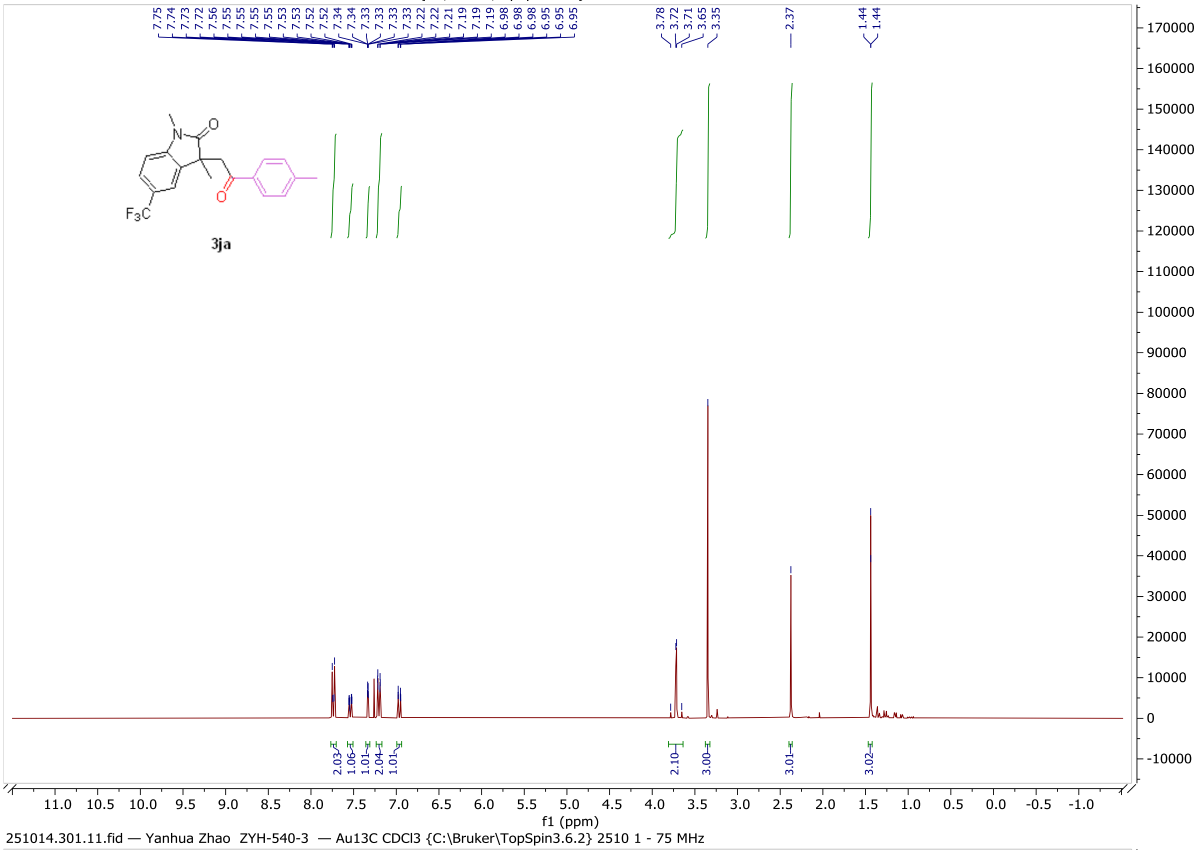

251014.301.11.fid — Yanhua Zhao ZYH-540-3 — Au13C CDCl3 {C:\Bruker\TopSpin3.6.2} 2510 1 - 75 MHz

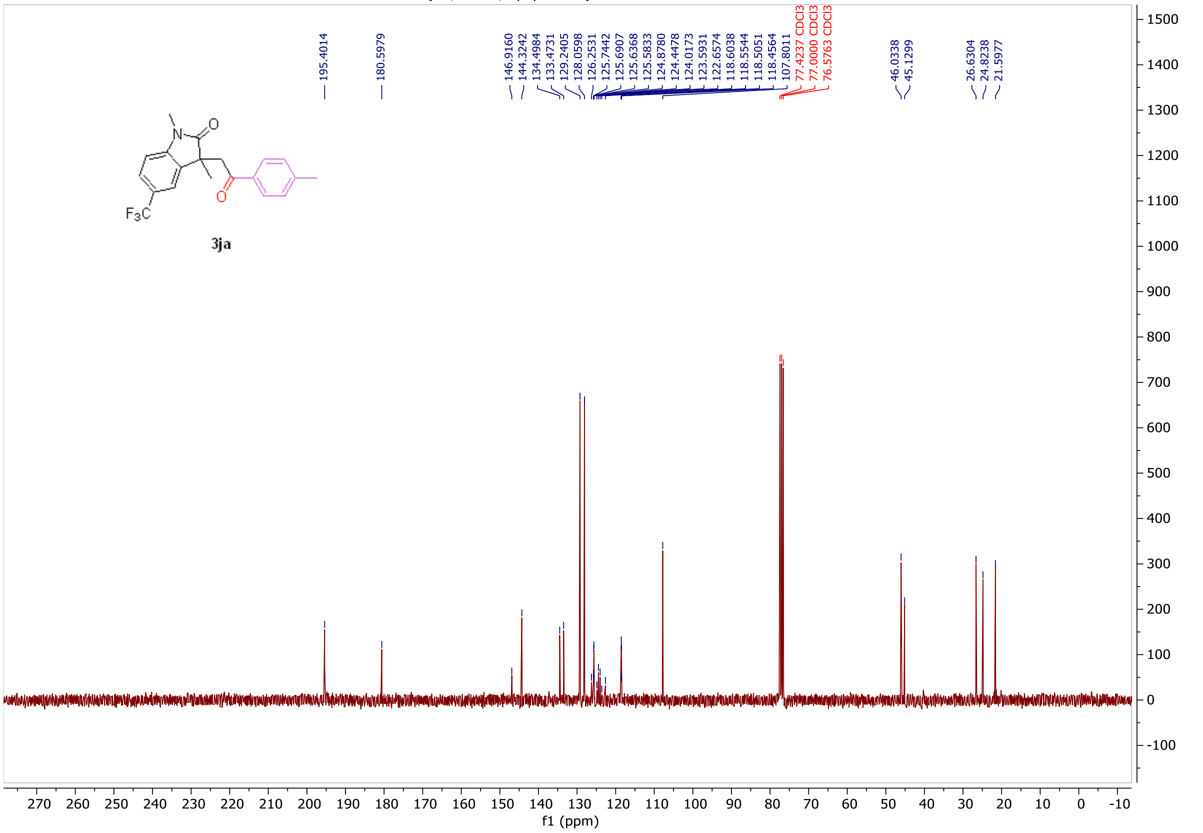

251012.325.11.fid — Yanhua Zhao, ZYH-540-3 — Au19F CDCl3 {C:\Bruker\TopSpin3.6.2} 2510 25-282 MHz

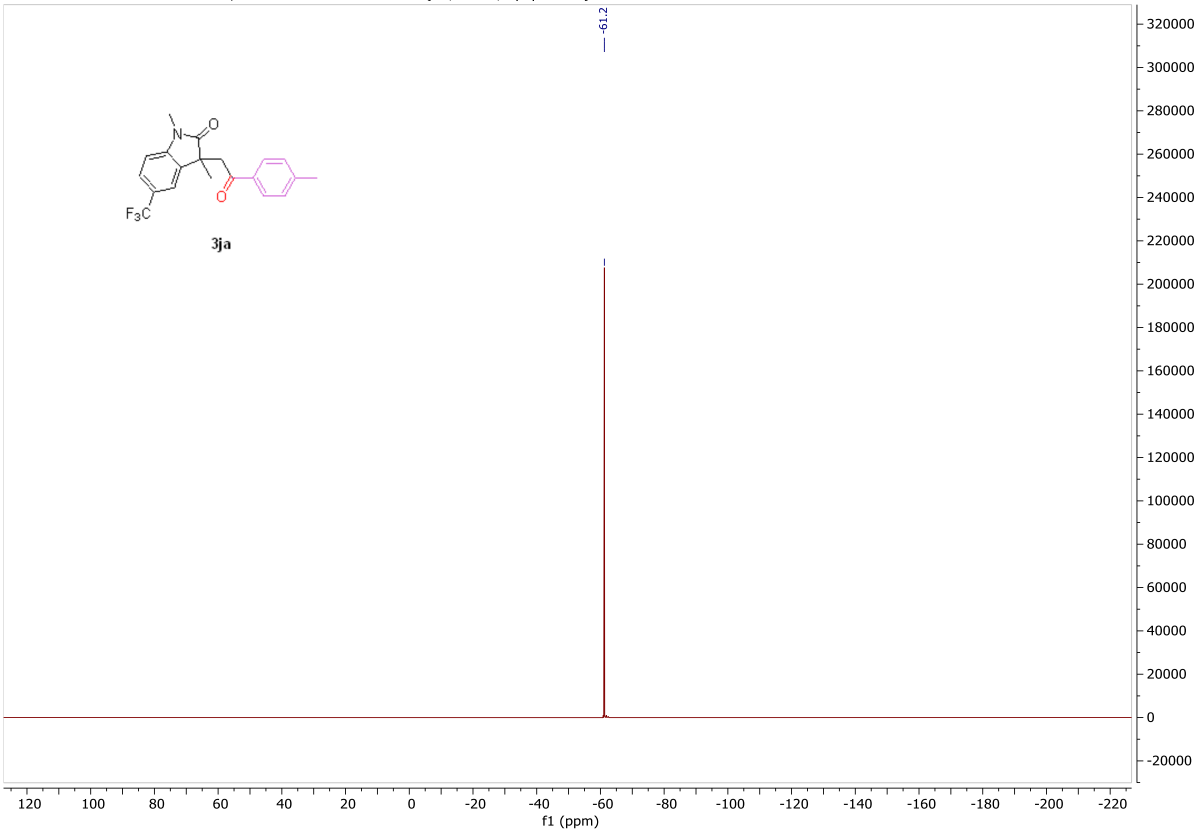

251114.f318.10.fid — Yanhua Zhao ZYH-540-7 — Au1H CDCl3 {C:\Bruker\TopSpin3.6.2} 2511 18 - 300 MHz

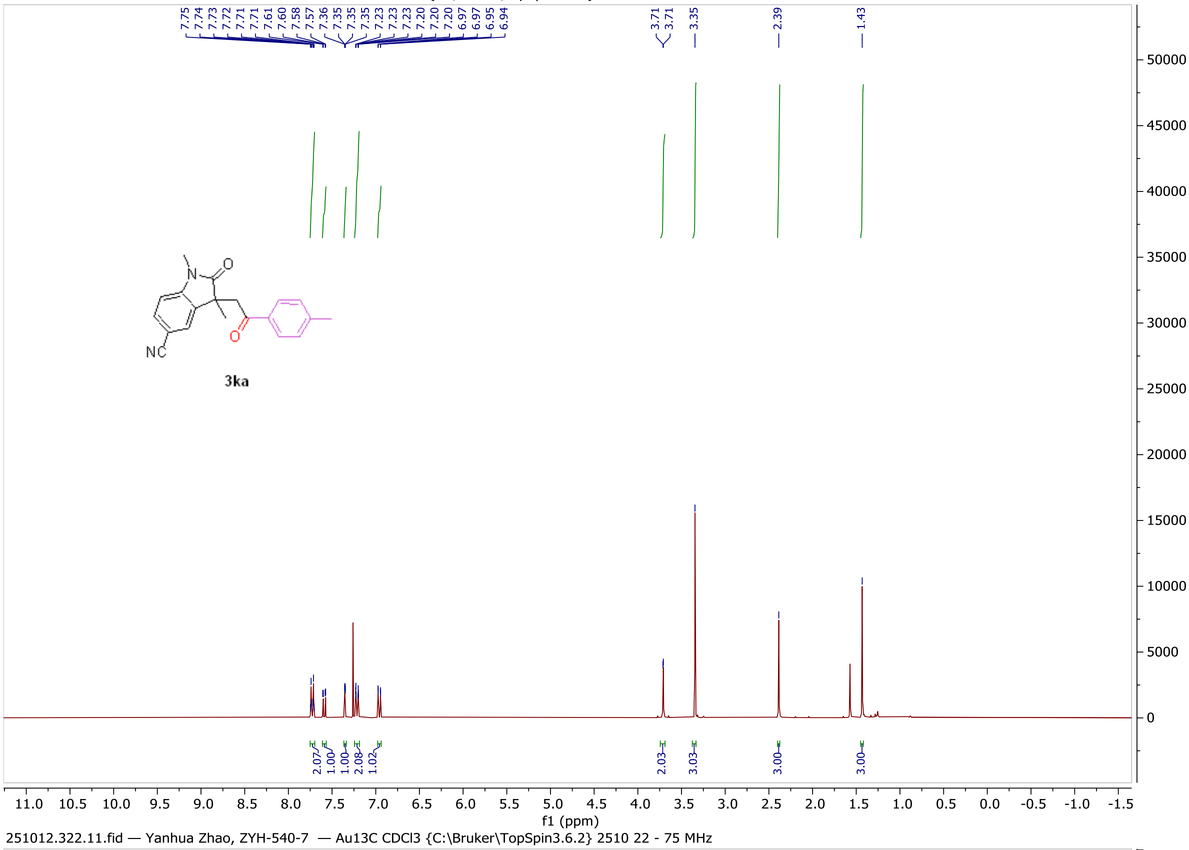

251012.322.11.fid — Yanhua Zhao, ZYH-540-7 — Au13C CDCl3 {C:\Bruker\TopSpin3.6.2} 2510 22 - 75 MHz

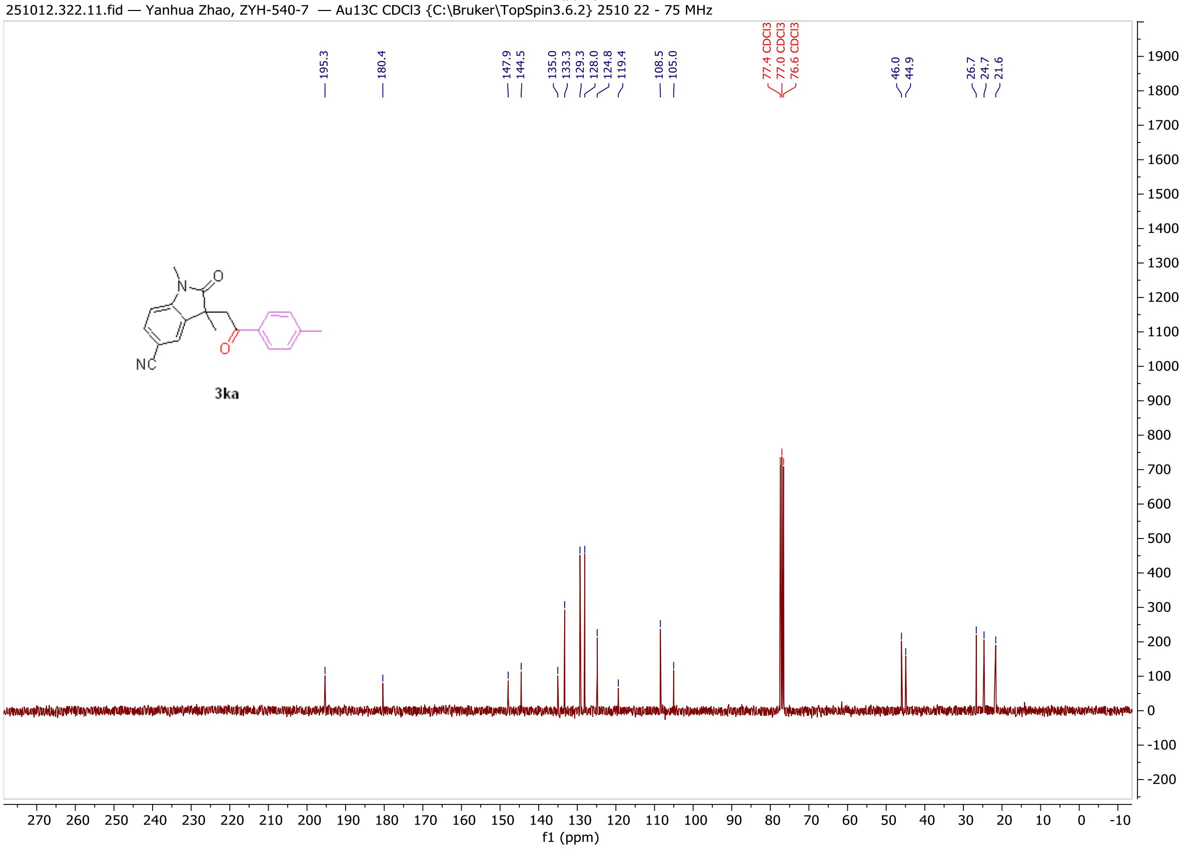

251012.319.10.fid — Yanhua Zhao, ZYH-540-4 — Au1H CDCl3 {C:\Bruker\TopSpin3.6.2} 2510 19 - 300 MHz

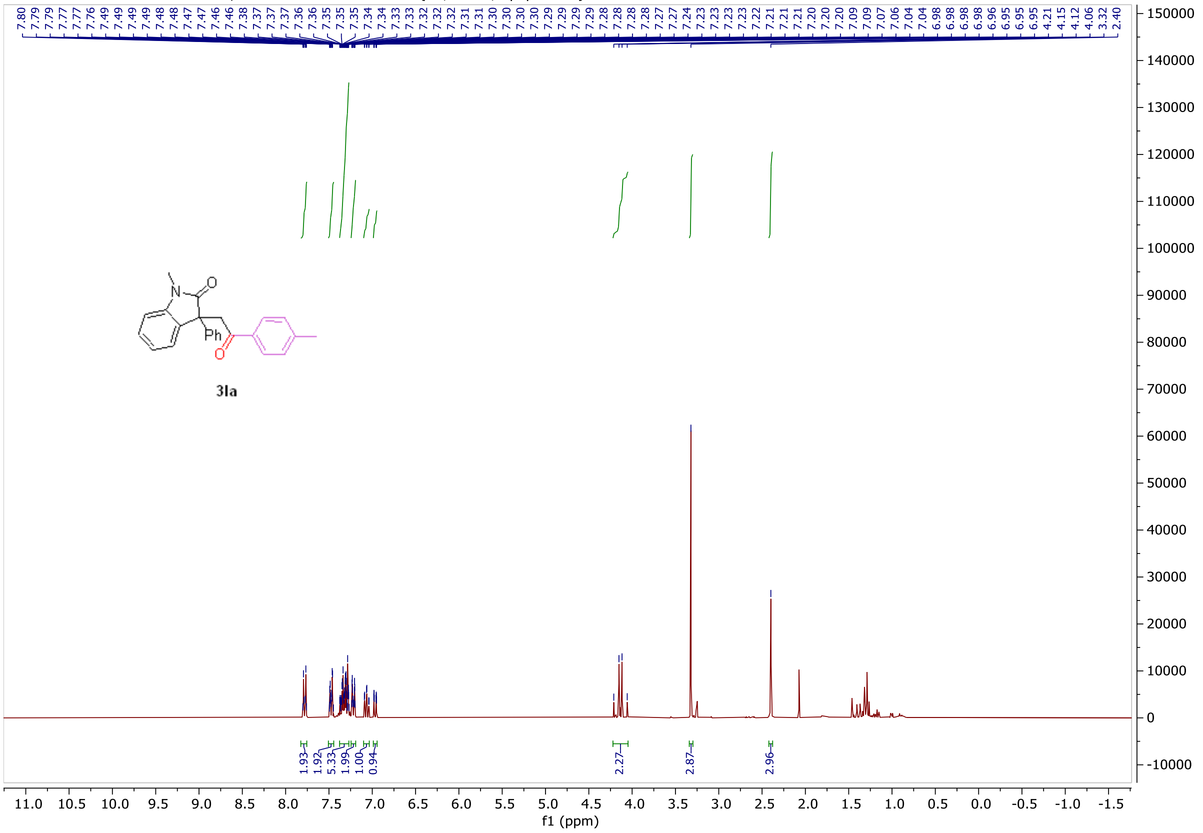

251012.319.11.fid — Yanhua Zhao, ZYH-540-4 — Au13C CDCl3 {C:\Bruker\TopSpin3.6.2} 2510 19 - 75 MHz

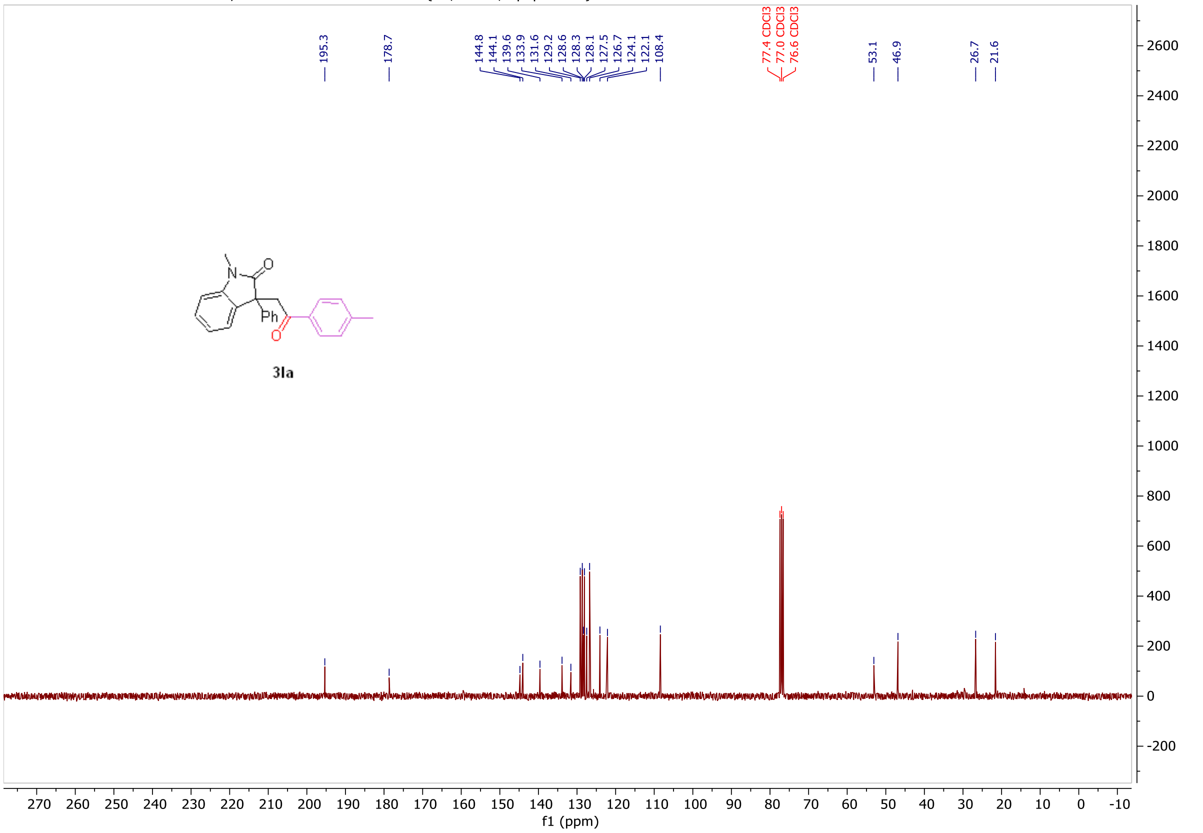

251104.f311.10.fid — Yanhua Zhao ZYH-540-5 — Au1H CDCl3 {C:\Bruker\TopSpin3.6.2} 2511 11 - 300 MHz

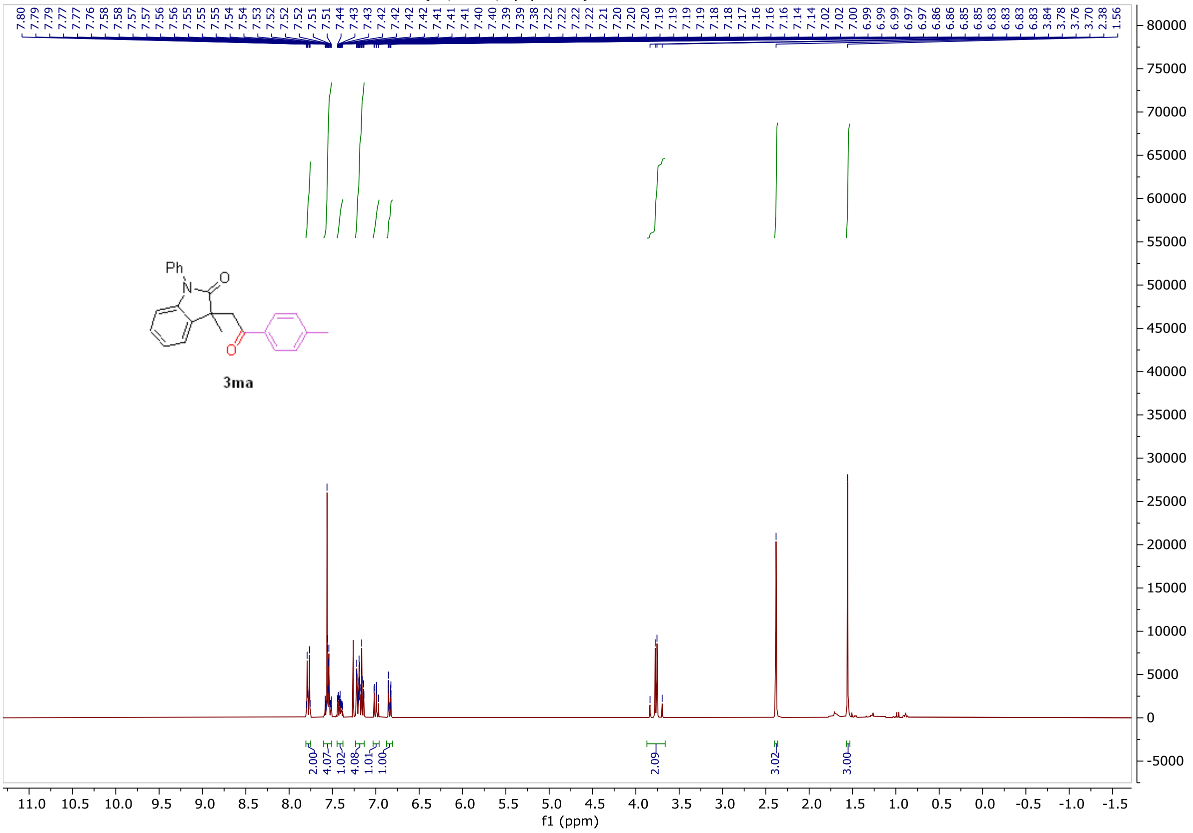

251012.320.11.fid — Yanhua Zhao, ZYH-540-5 — Au13C CDCl3 {C:\Bruker\TopSpin3.6.2} 2510 20 - 75 MHz

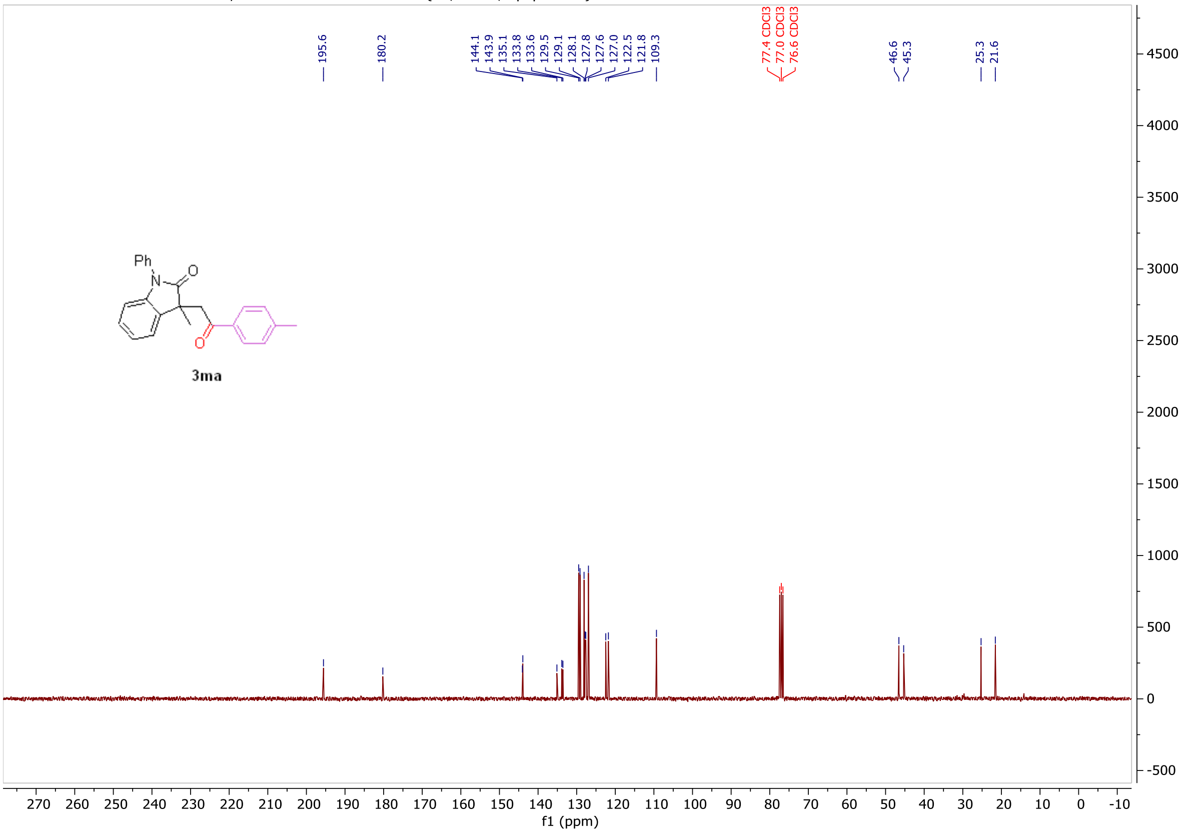

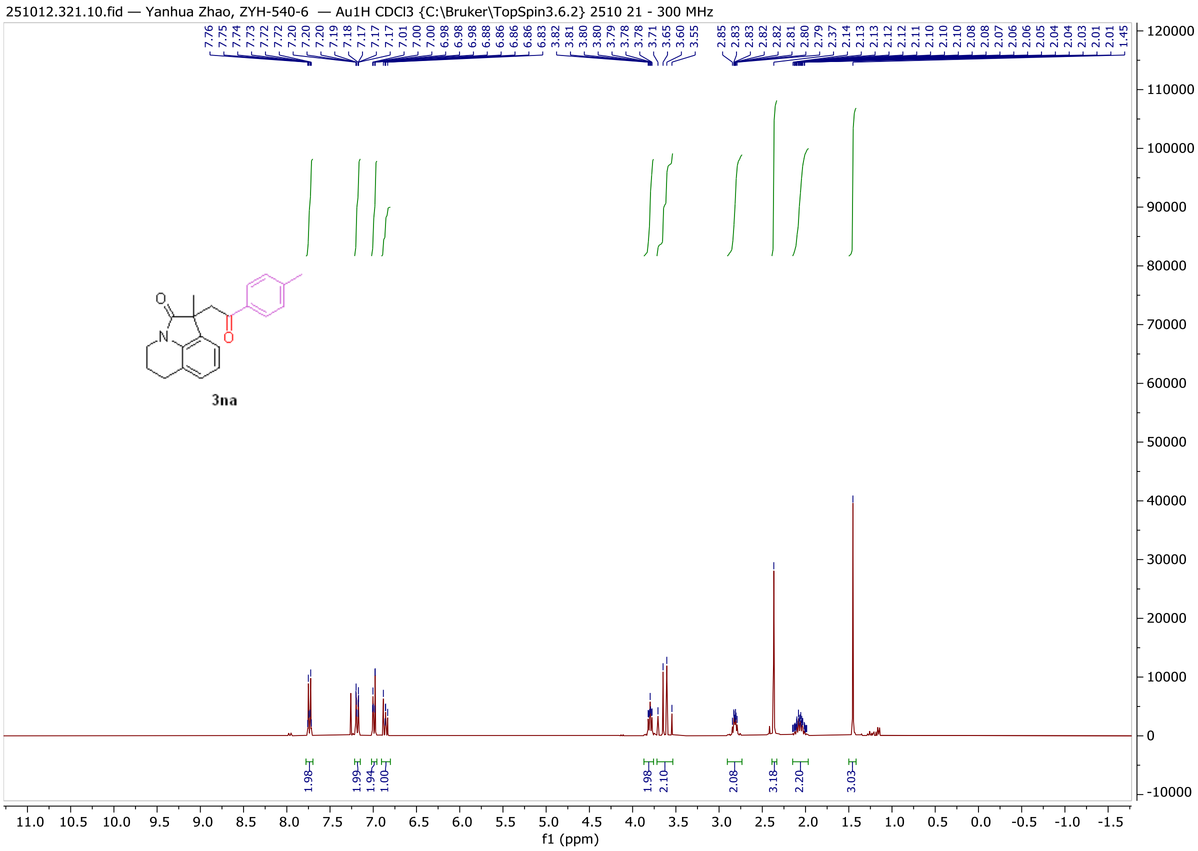
251012.321.10.fid — Yanhua Zhao, ZYH-540-6 — Au1H CDCl3 {C:\Bruker\TopSpin3.6.2} 2510 21 - 300 MHz
7.76 7.75 7.74 7.73 7.72 7.22 7.20 7.20 7.19 7.18 7.17 7.17 7.17 7.01 7.00 7.00 6.98 6.98 6.98 6.88 6.88 6.86 6.86 6.86 6.83 3.82 3.81 3.80 3.80 3.79 3.78 3.78 3.71 3.65 3.60 3.55 2.85 2.83 2.83 2.82 2.82 2.81 2.80 2.79 2.37 2.14 2.13 2.13 2.12 2.12 2.11 2.10 2.10 2.10 2.08 2.08 2.07 2.06 2.06 2.05 2.04 2.04 2.03 2.01 2.01 1.45
N
O
O
3na
1.98 1.99 1.94 1.00 1.98 2.10 2.08 3.18 2.20 3.03
f1 (ppm)

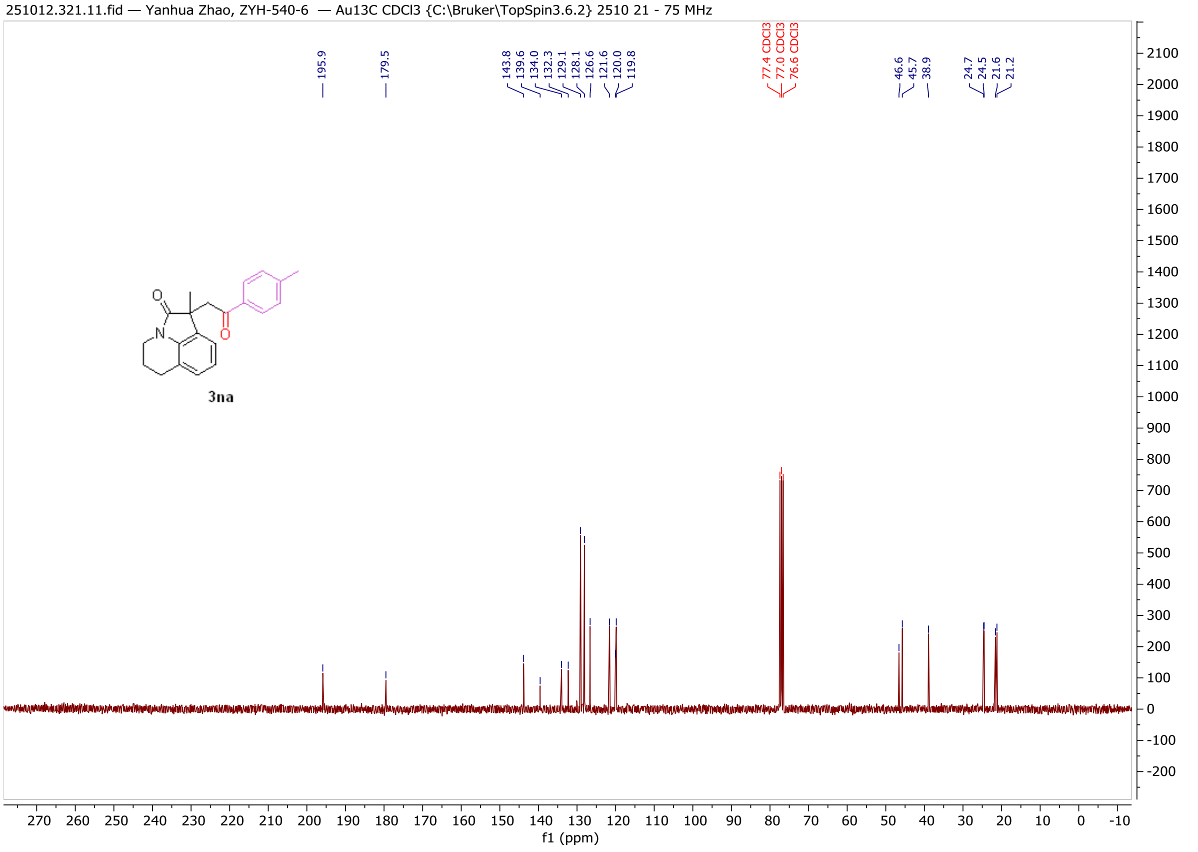
251012.321.11.fid — Yanhua Zhao, ZYH-540-6 — Au13C CDCl3 {C:\Bruker\TopSpin3.6.2} 2510 21 - 75 MHz
195.9
179.5
143.8 139.6 134.0 132.3 129.1 128.1 126.6 121.6 120.0 119.8
77.4 CDCl3 77.0 CDCl3 76.6 CDCl3
46.6 45.7 38.9
24.7 24.5 21.6 21.2
N
O
O
3na
f1 (ppm)

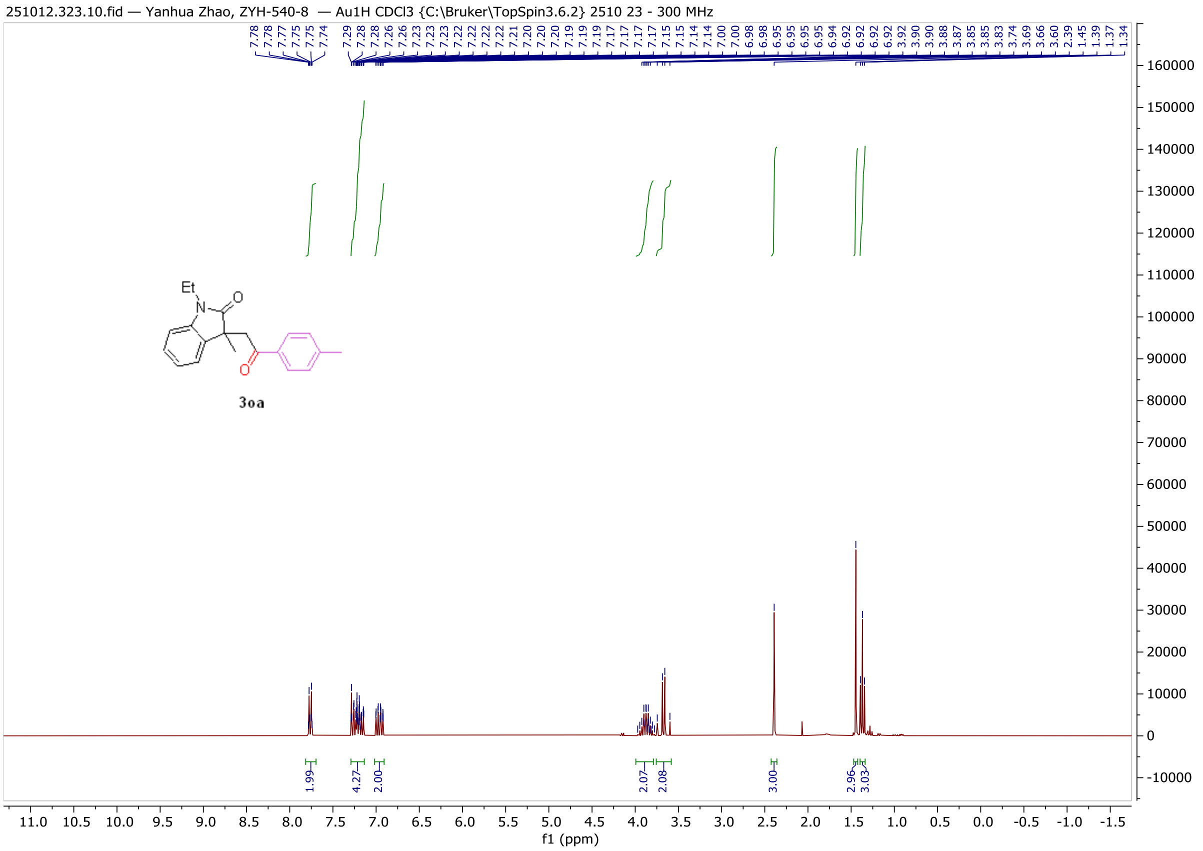
251012.323.10.fid — Yanhua Zhao, ZYH-540-8 — Au1H CDCl3 {C:\Bruker\TopSpin3.6.2} 2510 23 - 300 MHz
7.78 7.78 7.77 7.75 7.75 7.74 7.29 7.28 7.28 7.26 7.26 7.23 7.23 7.22 7.22 7.22 7.22 7.21 7.20 7.20 7.20 7.19 7.19 7.19 7.17 7.17 7.17 7.17 7.15 7.15 7.14 7.14 7.00 7.00 6.98 6.98 6.95 6.95 6.95 6.95 6.94 6.92 6.92 6.92 6.92 3.92 3.90 3.90 3.88 3.87 3.85 3.85 3.83 3.74 3.69 3.66 3.60 2.39 1.45 1.39 1.37 1.34
Et
N
O
3oa
1.99 4.27 2.00 2.07 2.08 3.00 2.96 3.03
f1 (ppm)

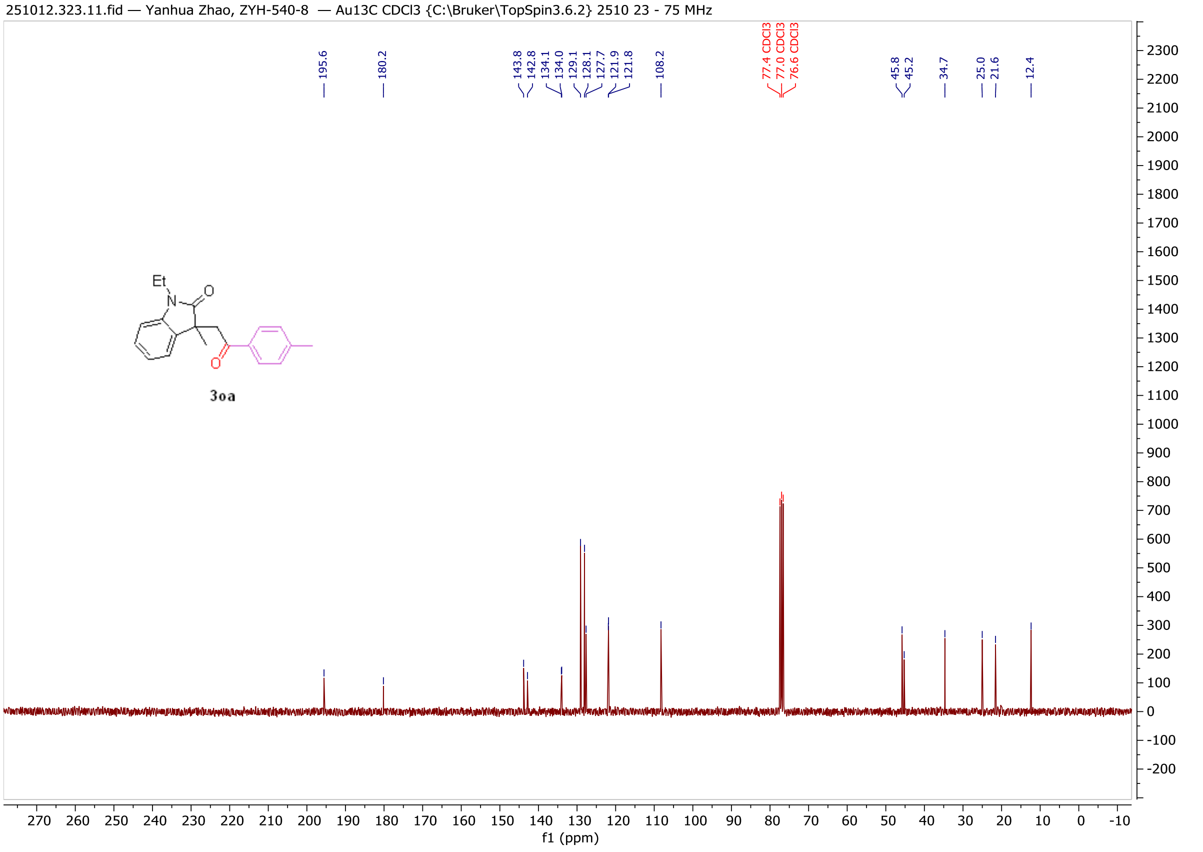
251012.323.11.fid — Yanhua Zhao, ZYH-540-8 — Au13C CDCl3 {C:\Bruker\TopSpin3.6.2} 2510 23 - 75 MHz
195.6 180.2 143.8 142.8 134.1 134.0 129.1 128.1 127.7 121.9 121.8 108.2
77.4 CDCl3 77.0 CDCl3 76.6 CDCl3
45.8 45.2 34.7 25.0 21.6 12.4
Et
N
O
3oa
f1 (ppm)

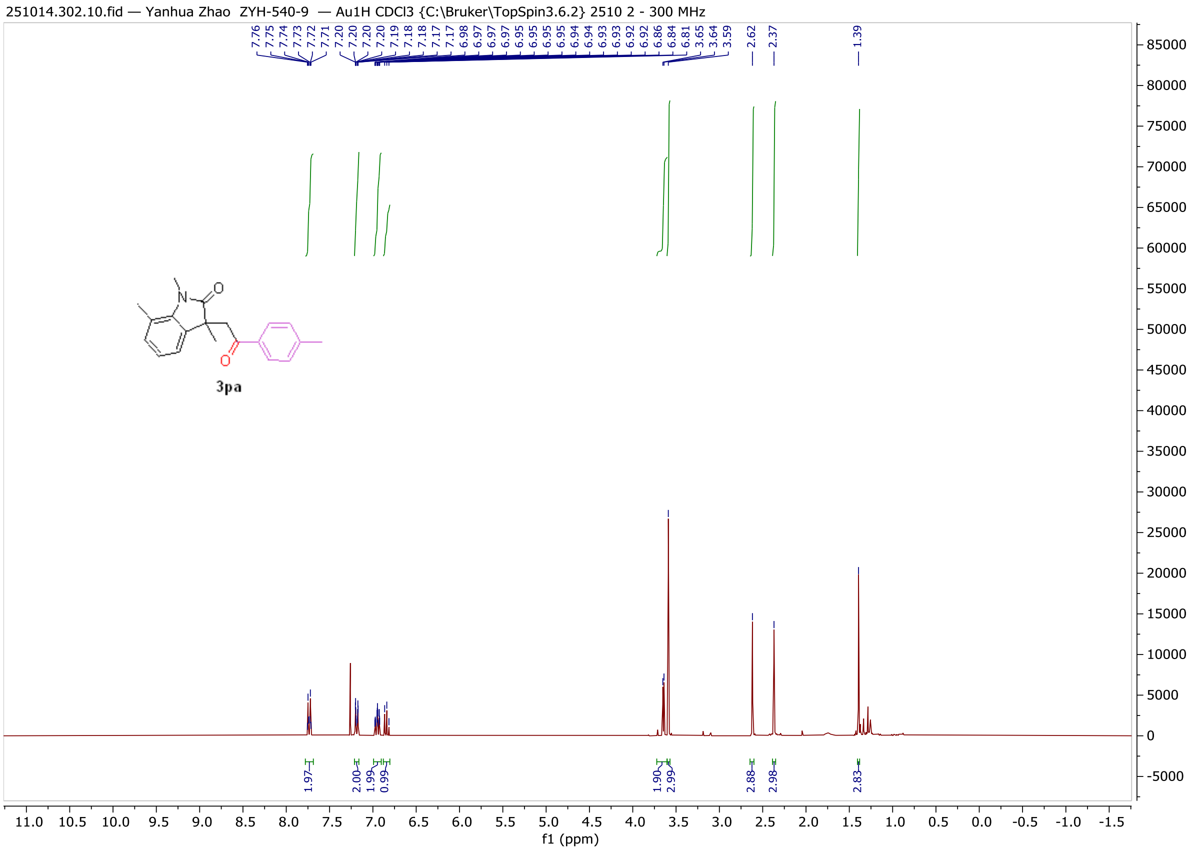
251014.302.10.fid — Yanhua Zhao ZYH-540-9 — Au1H CDCl3 {C:\Bruker\TopSpin3.6.2} 2510 2 - 300 MHz
3pa
f1 (ppm)

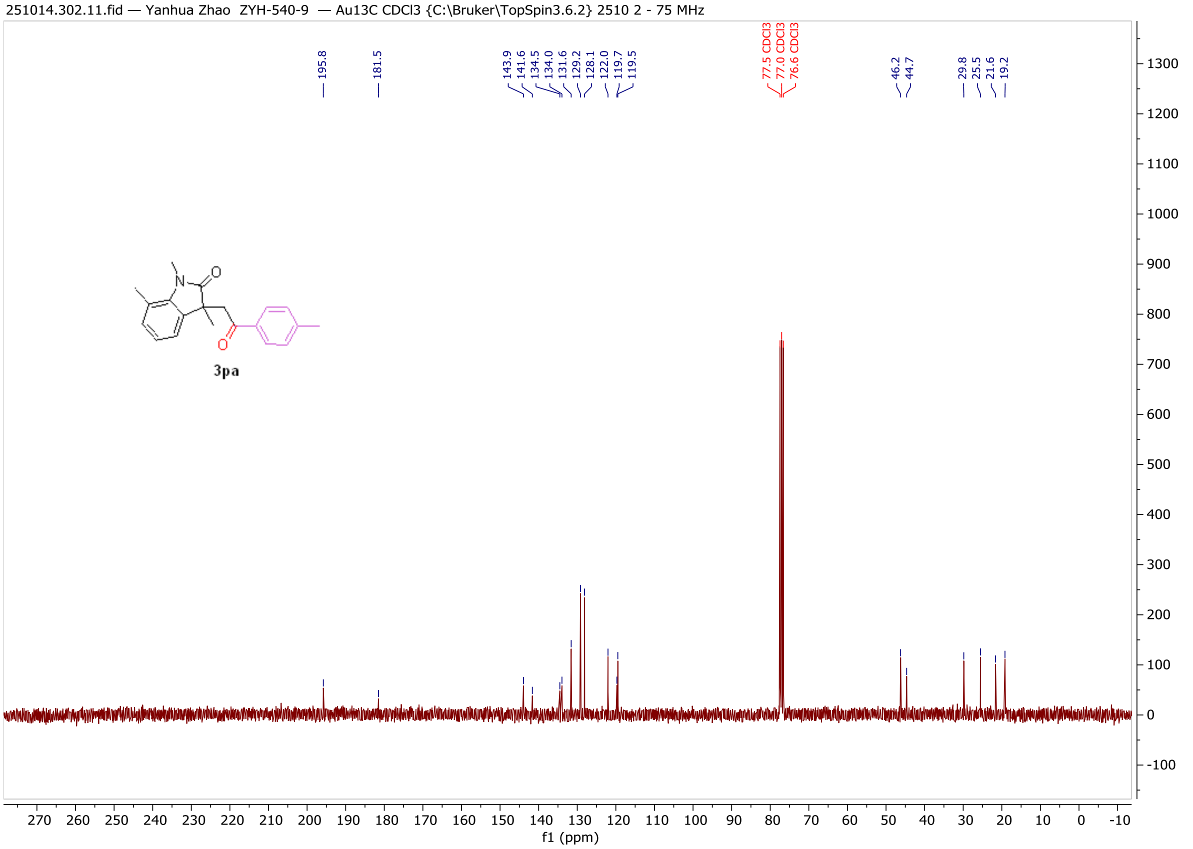
251014.302.11.fid — Yanhua Zhao ZYH-540-9 — Au13C CDCl3 {C:\Bruker\TopSpin3.6.2} 2510 2 - 75 MHz
3pa
f1 (ppm)

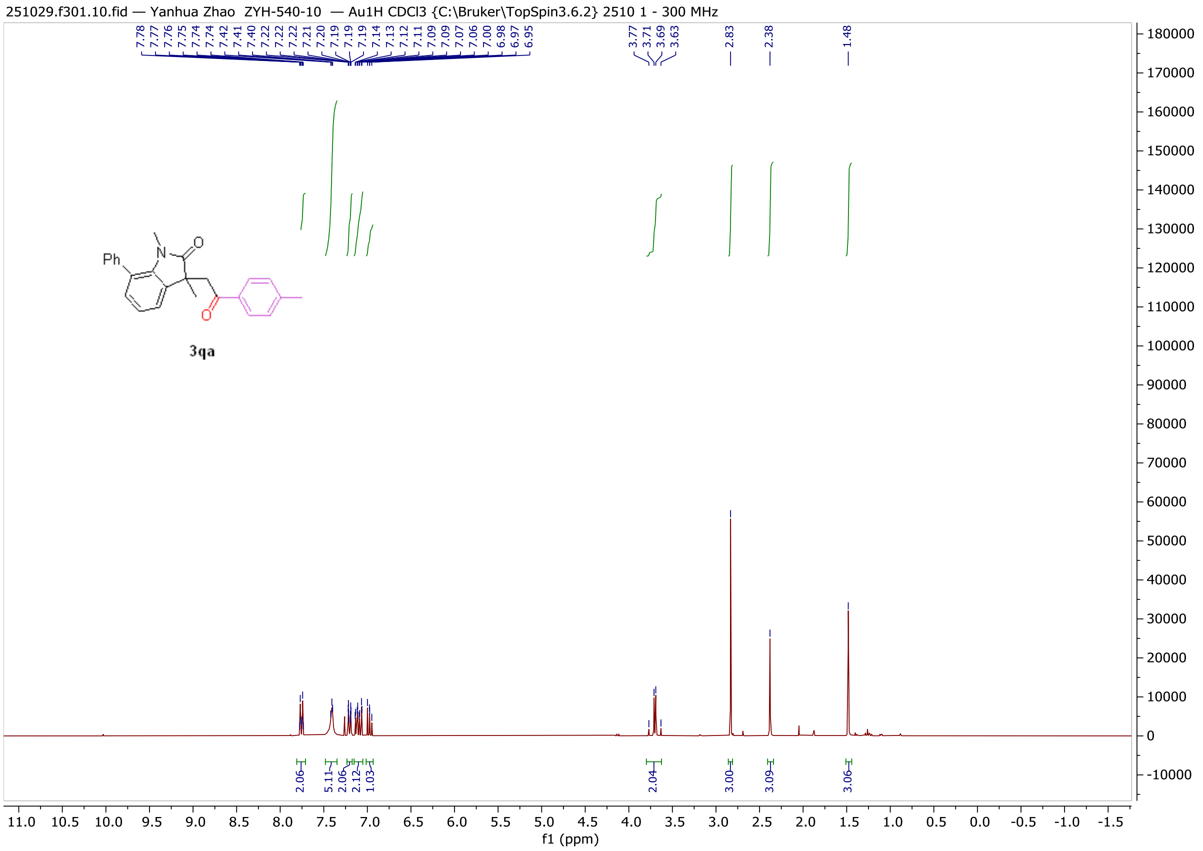
251029.f301.10.fid — Yanhua Zhao ZYH-540-10 — Au1H CDCl3 {C:\Bruker\TopSpin3.6.2} 2510 1 - 300 MHz
3qa
f1 (ppm)

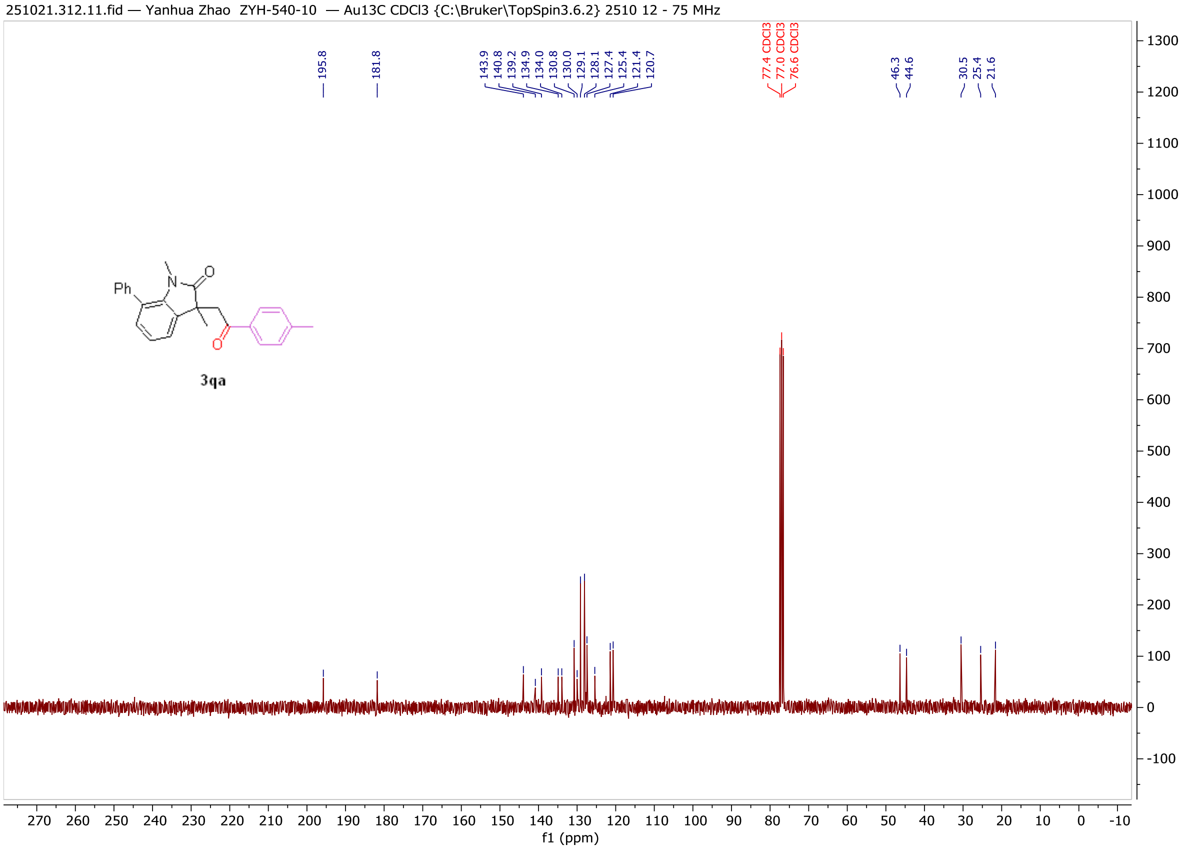
251021.312.11.fid — Yanhua Zhao ZYH-540-10 — Au13C CDCl3 {C:\Bruker\TopSpin3.6.2} 2510 12 - 75 MHz
3qa
f1 (ppm)

251029.f302.10.fid — Yanhua Zhao ZYH-540-12 — Au1H CDCl3 {C:\Bruker\TopSpin3.6.2} 2510 2 - 300 MHz

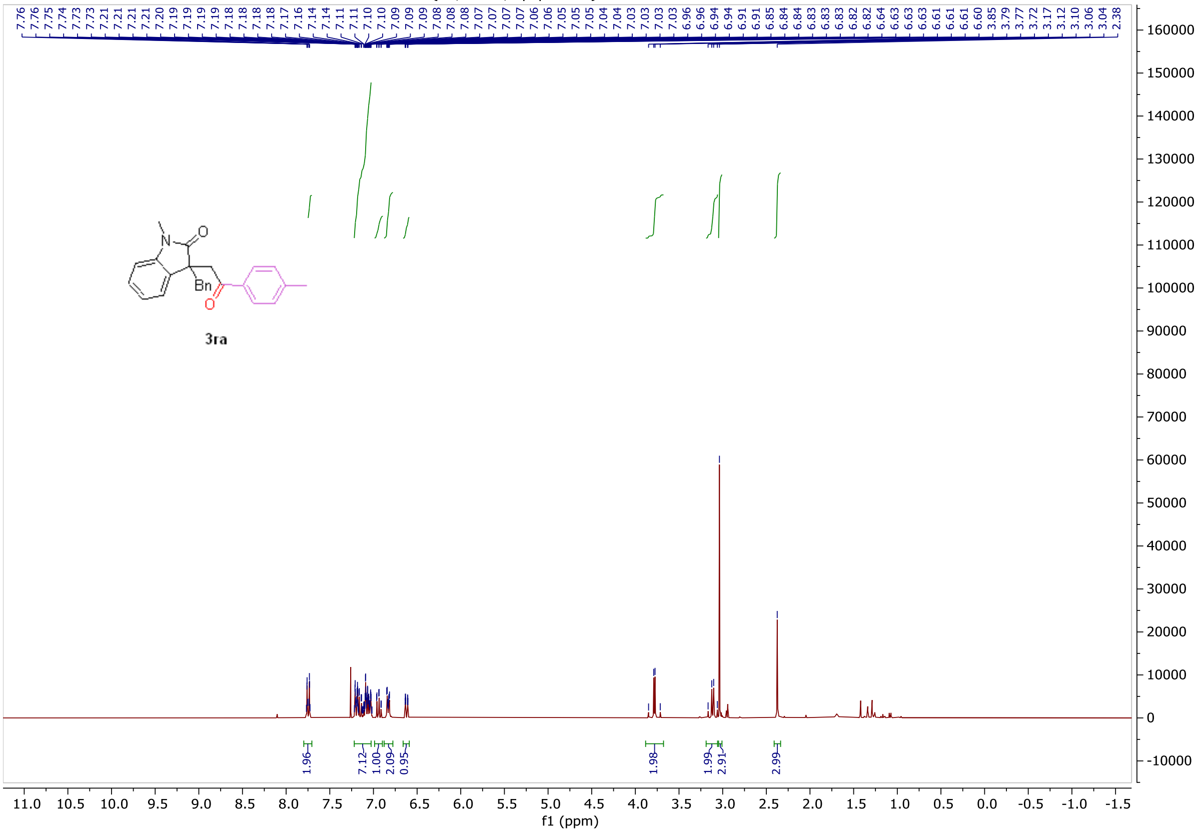

251022.f303.11.fid — Yanhua Zhao ZYH-540-12 — Au13C CDCl3 {C:\Bruker\TopSpin3.6.2} 2510 3 - 75 MHz

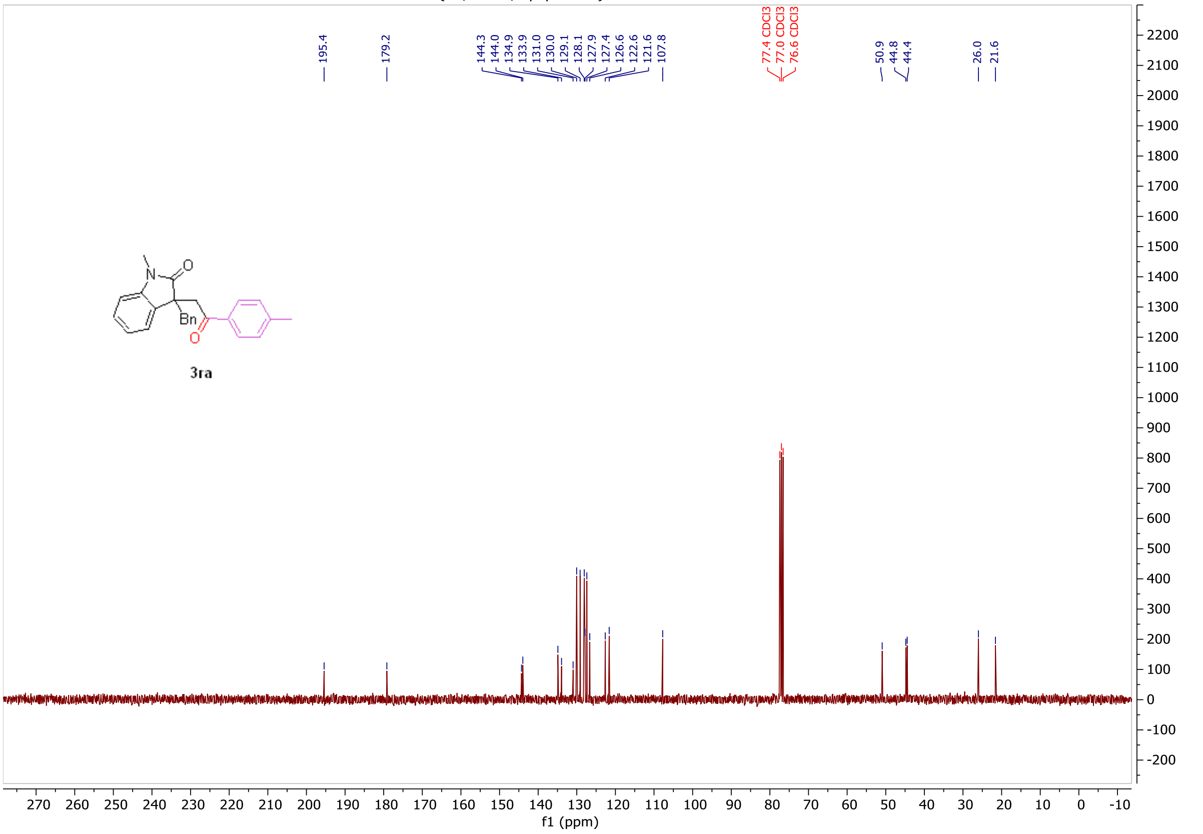

251022.f304.10.fid — Yanhua Zhao ZYH-540-13 — Au1H CDCl3 {C:\Bruker\TopSpin3.6.2} 2510 4 - 300 MHz

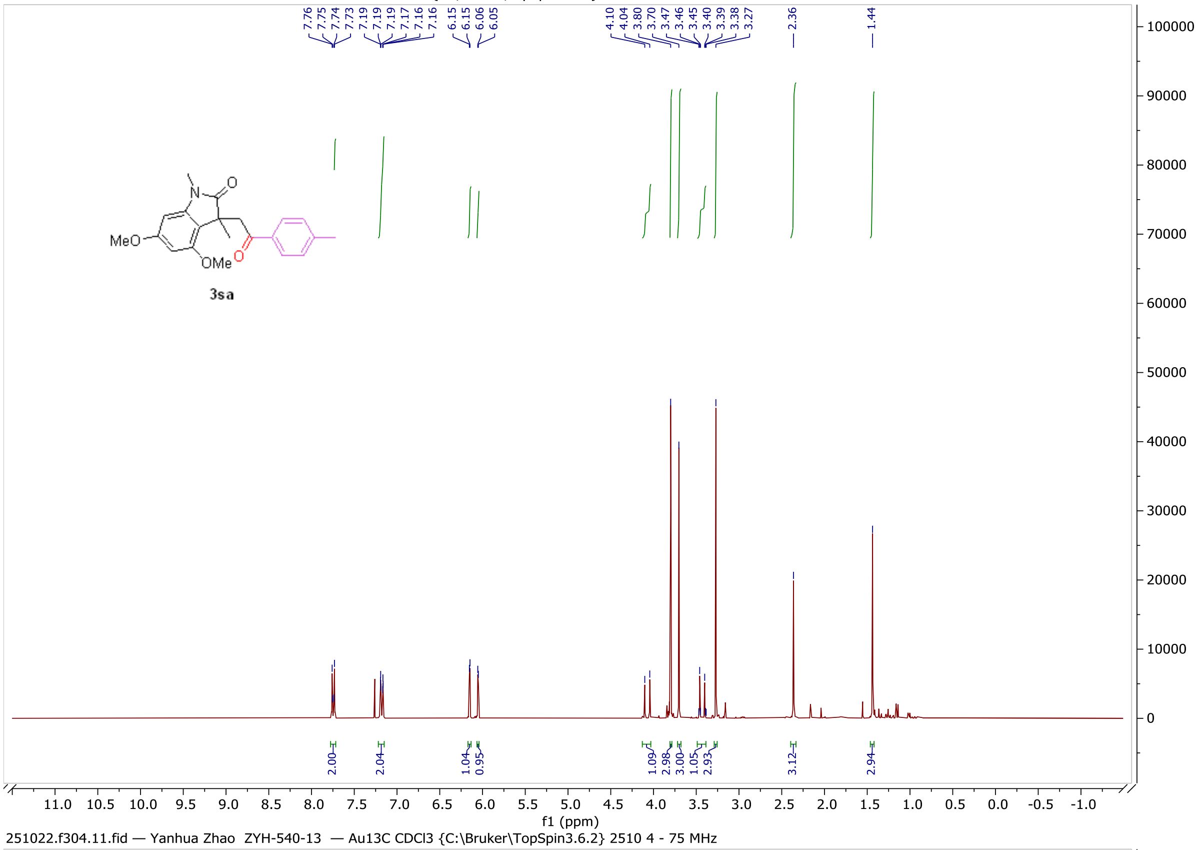

251022.f304.11.fid — Yanhua Zhao ZYH-540-13 — Au13C CDCl3 {C:\Bruker\TopSpin3.6.2} 2510 4 - 75 MHz

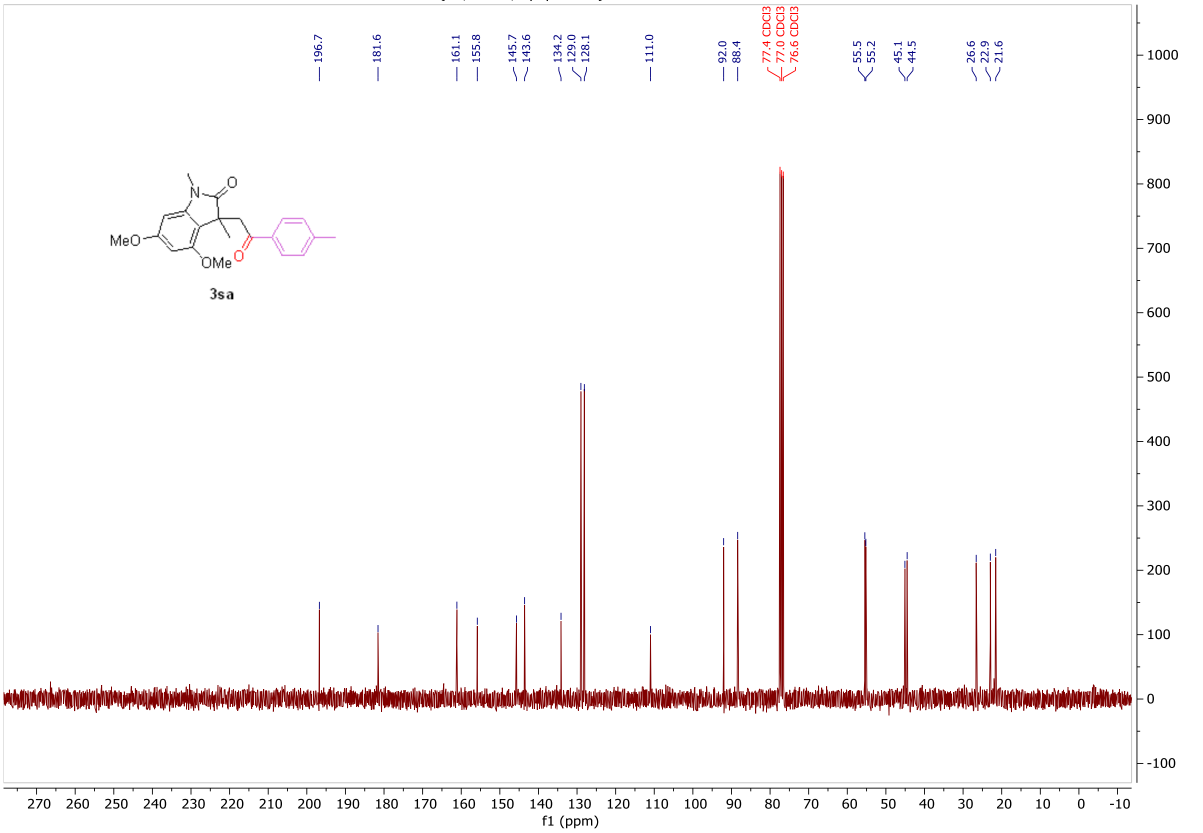

251023.323.10.fid — Yanhua Zhao, ZYH-540-14 — Au1H CDCl3 {C:\Bruker\TopSpin3.6.2} 2510 23 - 75 MHz

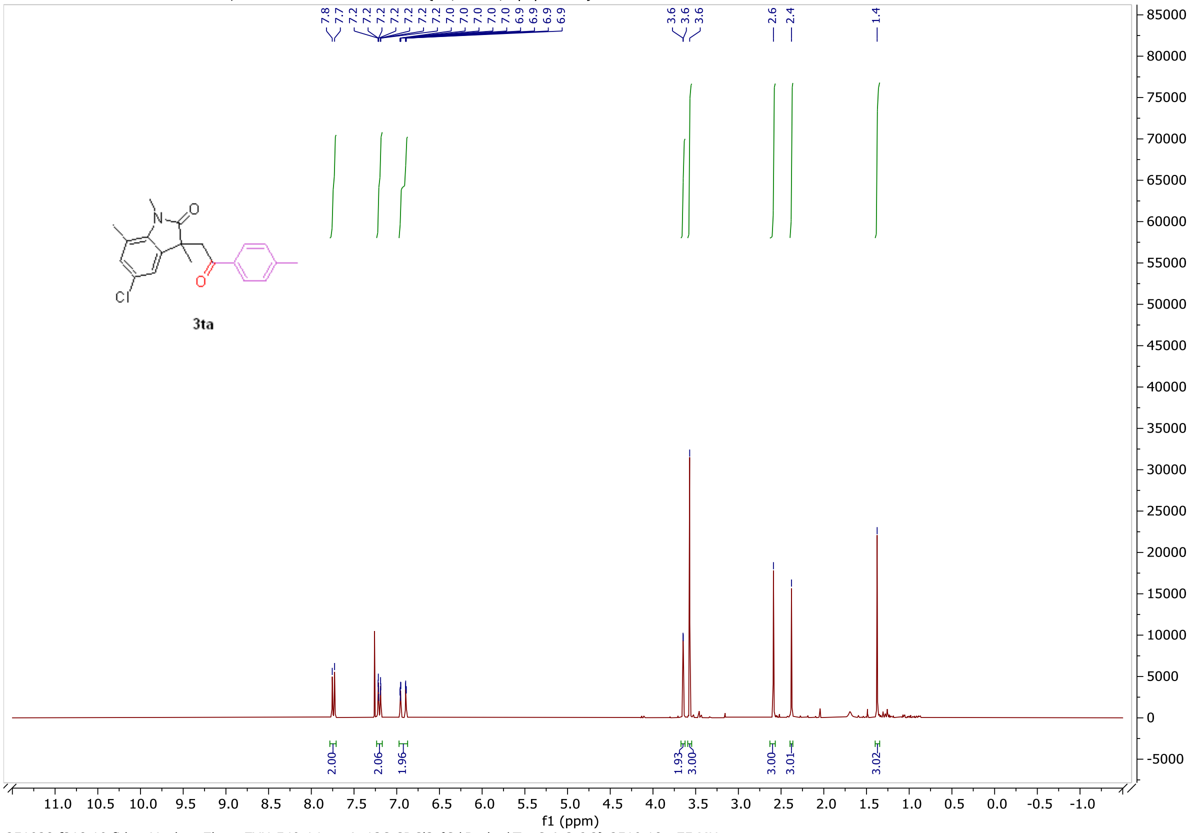

251028.f318.10.fid — Yanhua Zhao ZYH-540-14 — Au13C CDCl3 {C:\Bruker\TopSpin3.6.2} 2510 18 - 75 MHz

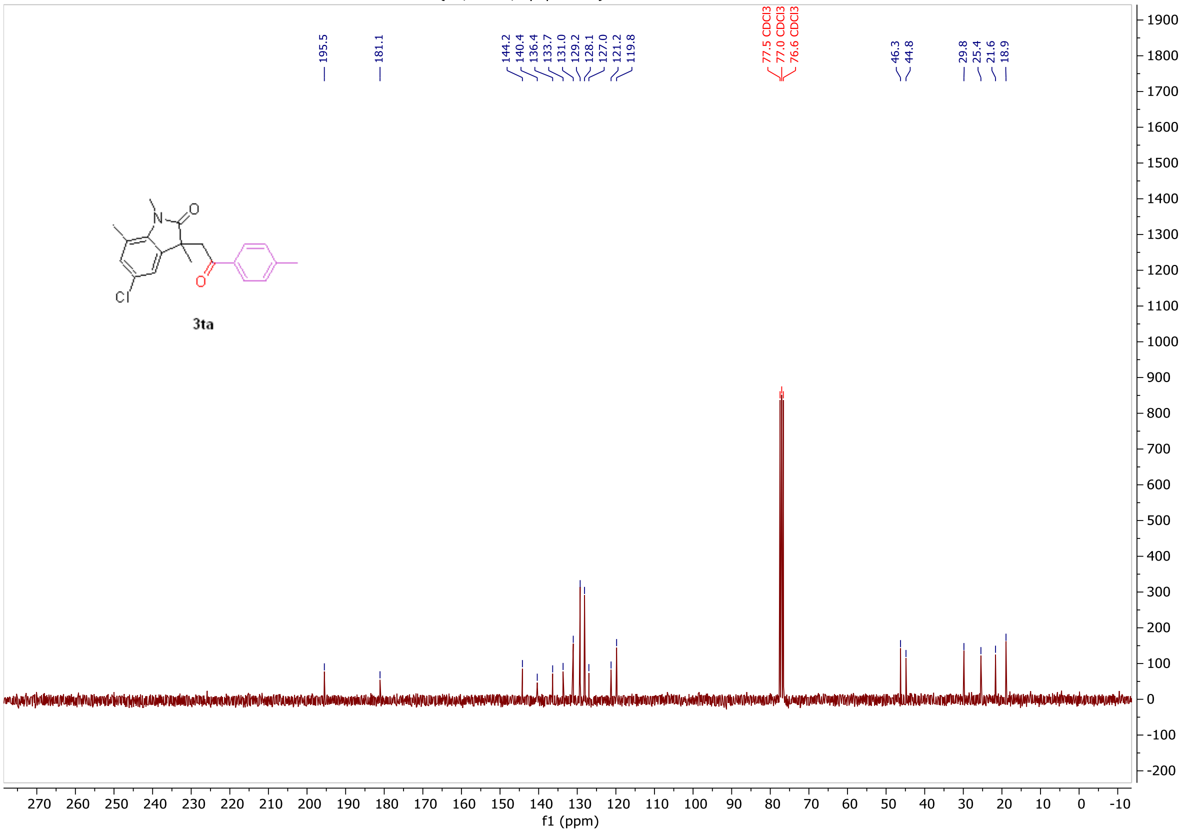

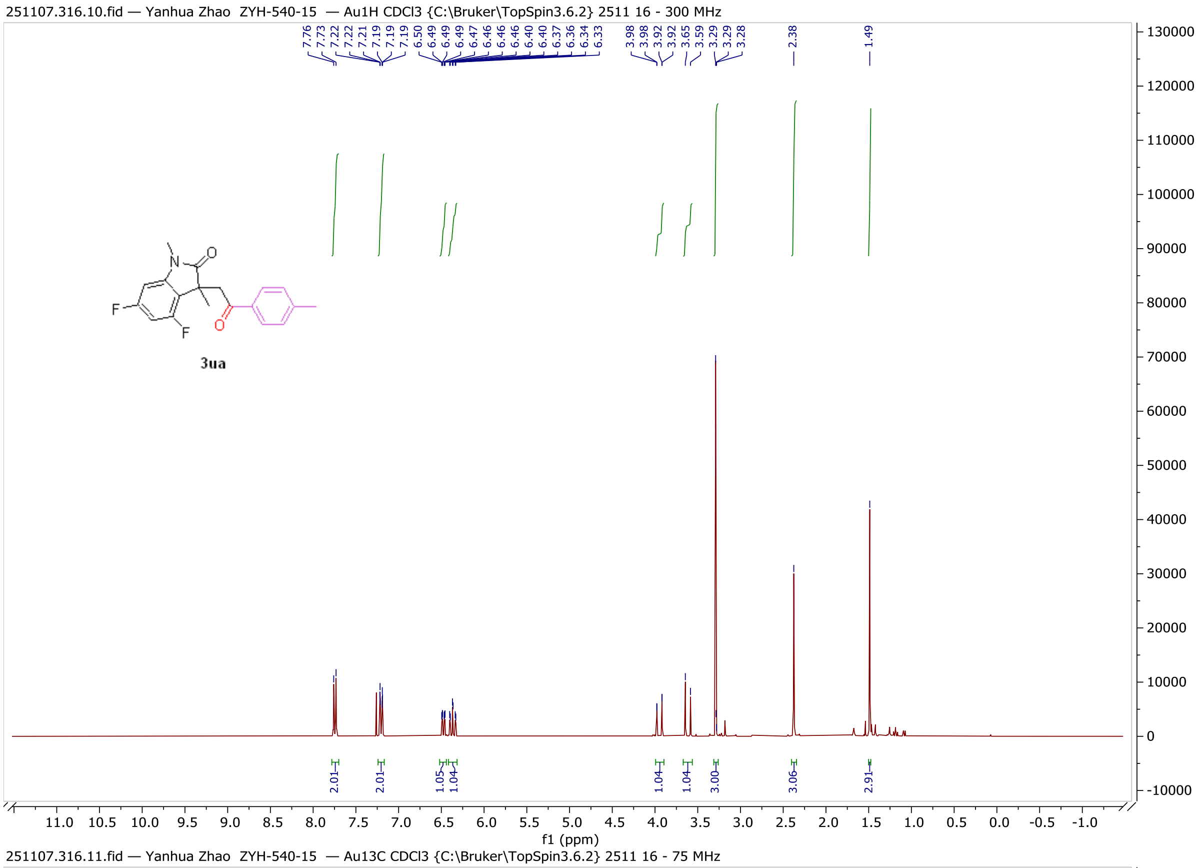
251107.316.10.fid — Yanhua Zhao ZYH-540-15 — Au1H CDCl3 {C:\Bruker\TopSpin3.6.2} 2511 16 - 300 MHz
7.76
7.73
7.22
7.22
7.21
7.19
7.19
7.19
6.50
6.49
6.49
6.49
6.47
6.46
6.46
6.46
6.40
6.40
6.37
6.36
6.34
6.33
3.98
3.98
3.92
3.92
3.65
3.59
3.29
3.29
3.28
2.38
1.49
2.01
2.01
1.05
1.04
1.04
1.04
3.00
3.06
2.91
3ua
f1 (ppm)

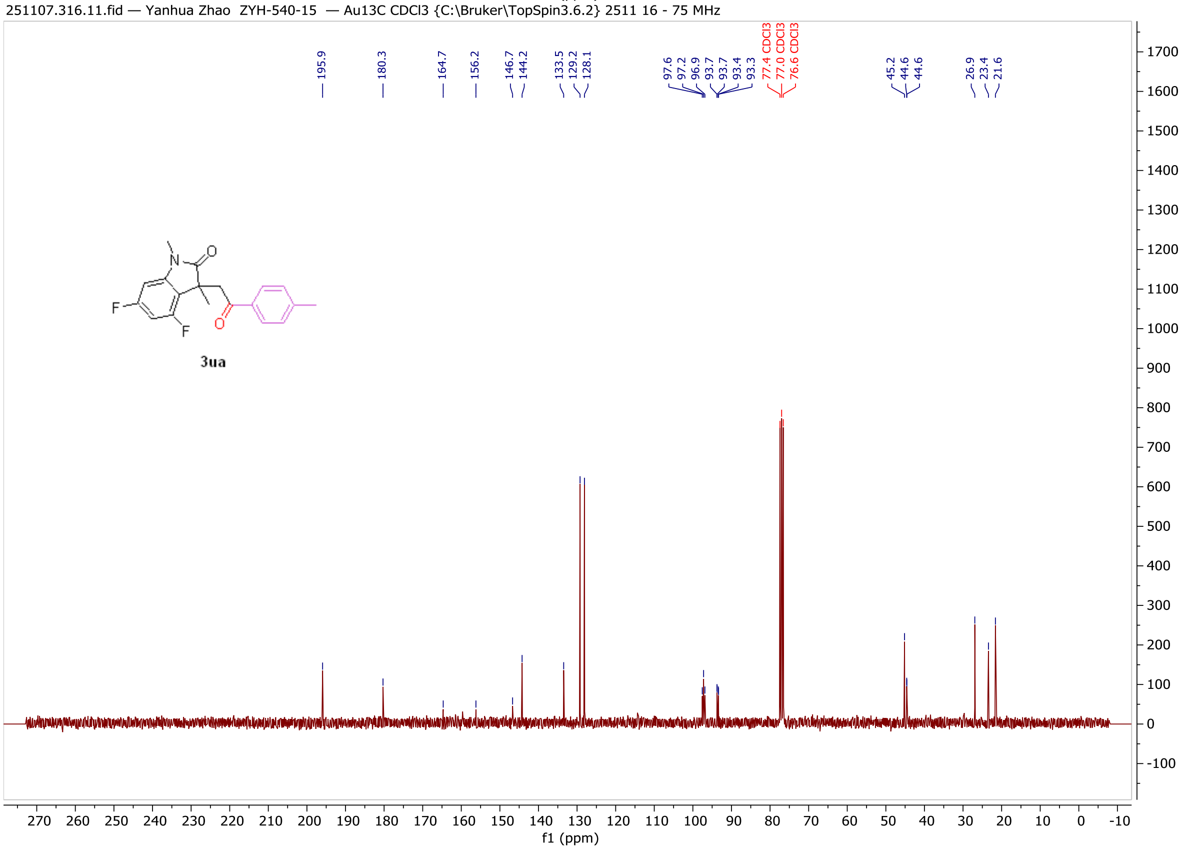
251107.316.11.fid — Yanhua Zhao ZYH-540-15 — Au13C CDCl3 {C:\Bruker\TopSpin3.6.2} 2511 16 - 75 MHz
195.9
180.3
164.7
156.2
146.7
144.2
133.5
129.2
128.1
97.6
97.2
96.9
93.7
93.7
93.4
93.3
77.4 CDCl3
77.0 CDCl3
76.6 CDCl3
45.2
44.6
44.6
26.9
23.4
21.6
3ua
f1 (ppm)

251113.326.10.fid — Yanhua Zhao ZYH-540-15 — Au19F CDCl3 {C:\Bruker\TopSpin3.6.2} 2511 26-282 MHz

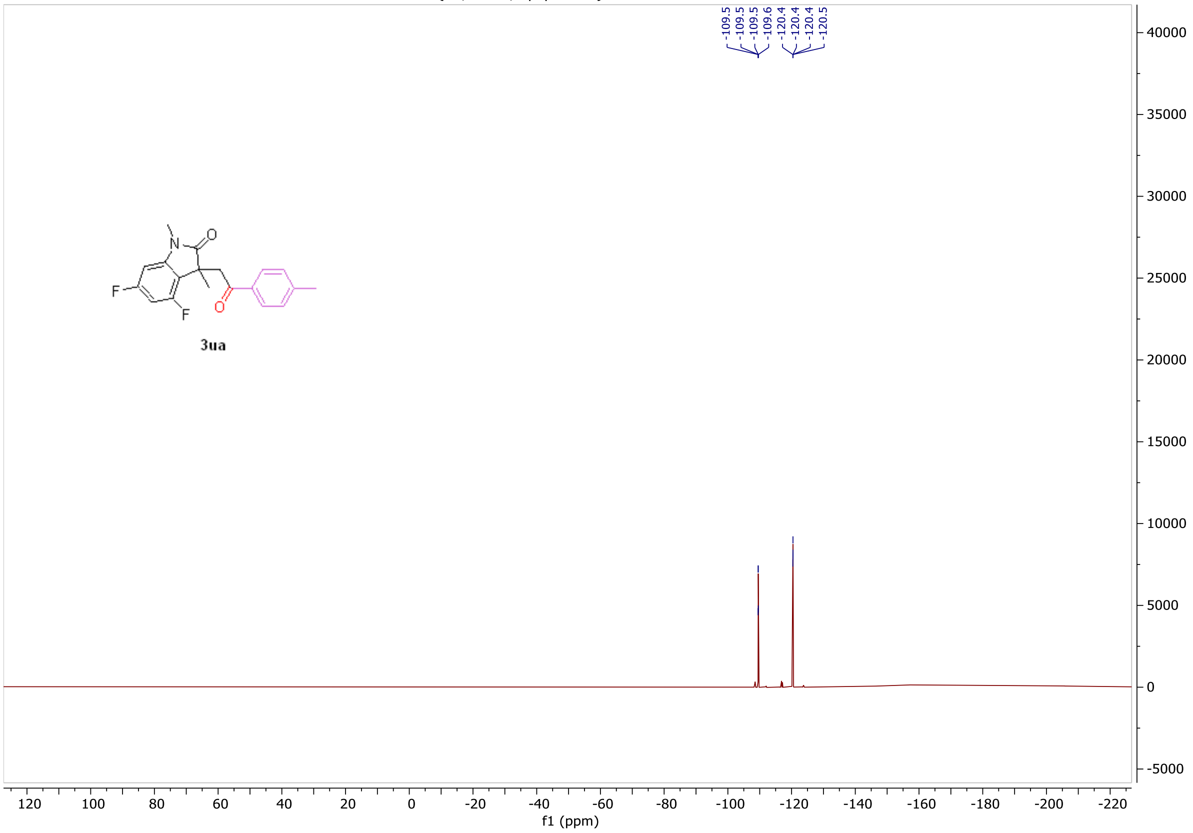

251029.f318.10.fid — Yanhua Zhao ZYH-540-16 — Au1H CDCl3 {C:\Bruker\TopSpin3.6.2} 2510 18 - 300 MHz

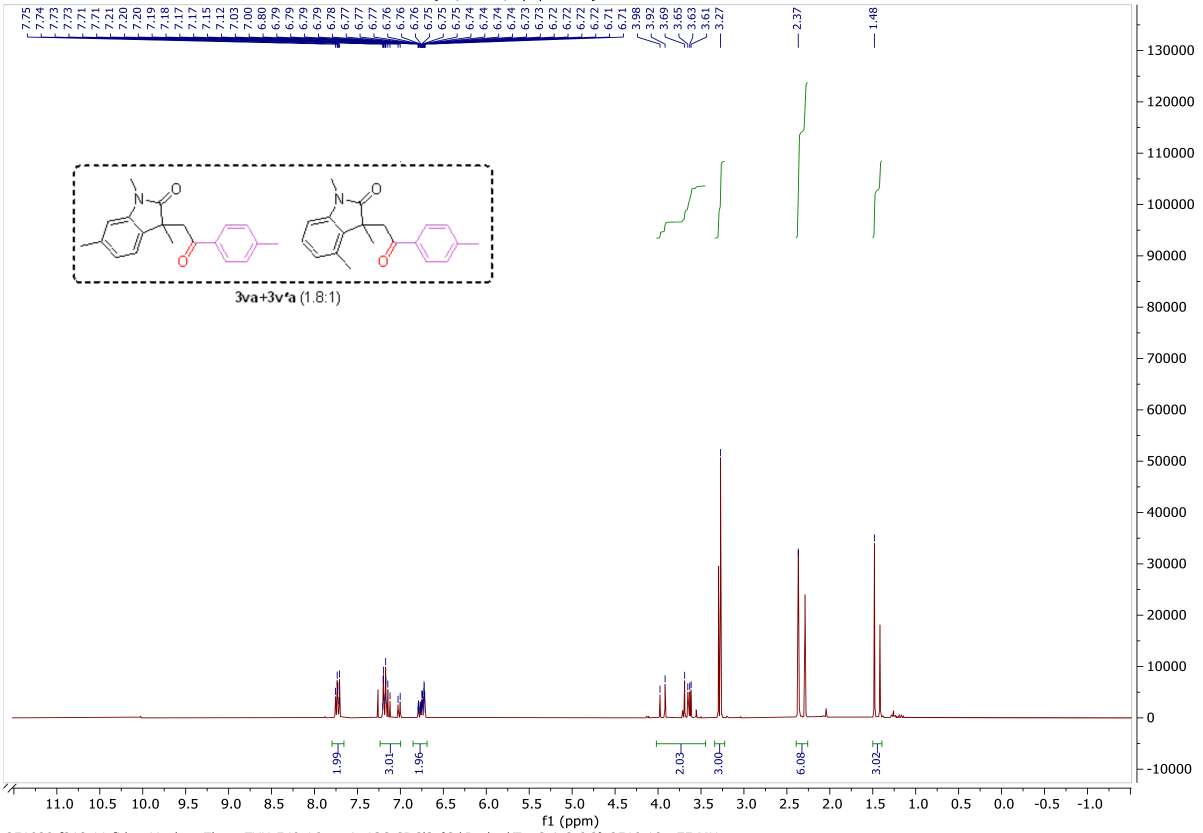

251029.f318.11.fid — Yanhua Zhao ZYH-540-16 — Au13C CDCl3 {C:\Bruker\TopSpin3.6.2} 2510 18 - 75 MHz

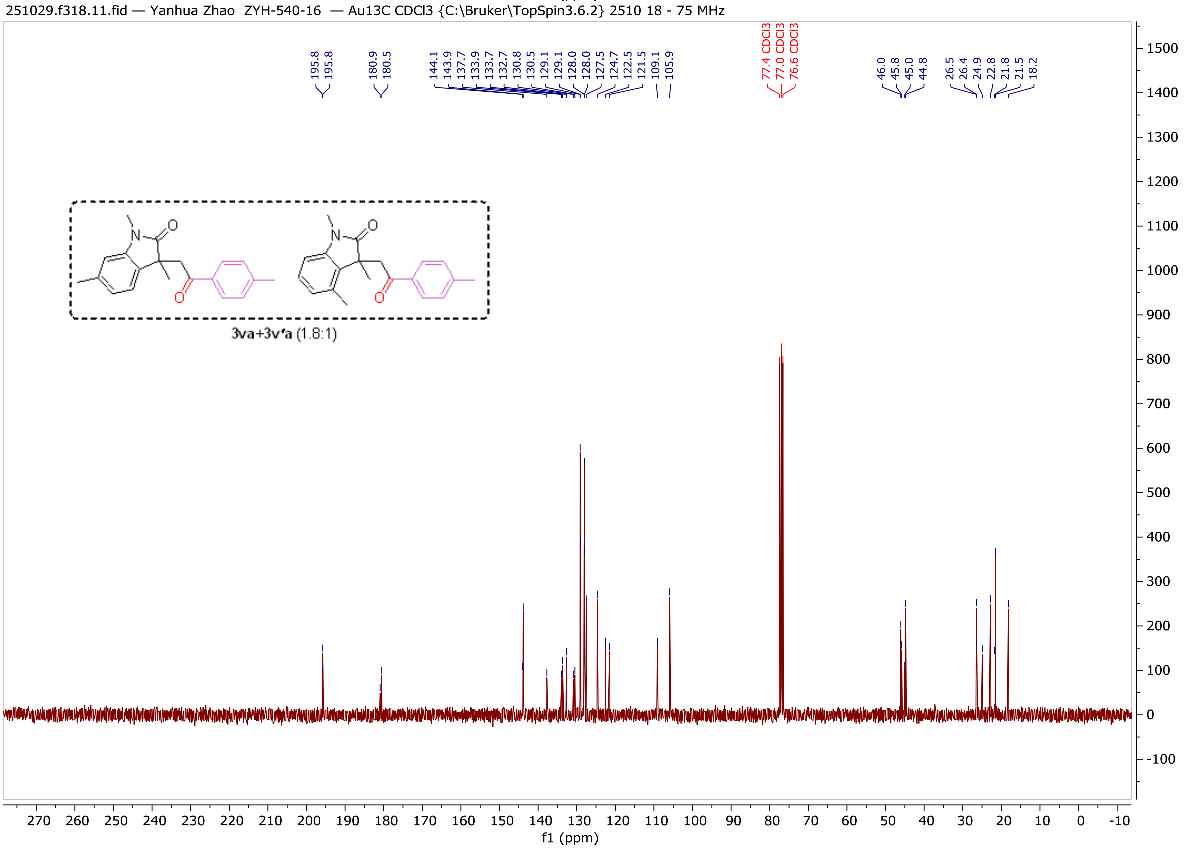

251021.315.10.fid — Yanhua Zhao ZYH-540-18 — Au1H CDCl3 {C:\Bruker\TopSpin3.6.2} 2510 15 - 75 MHz

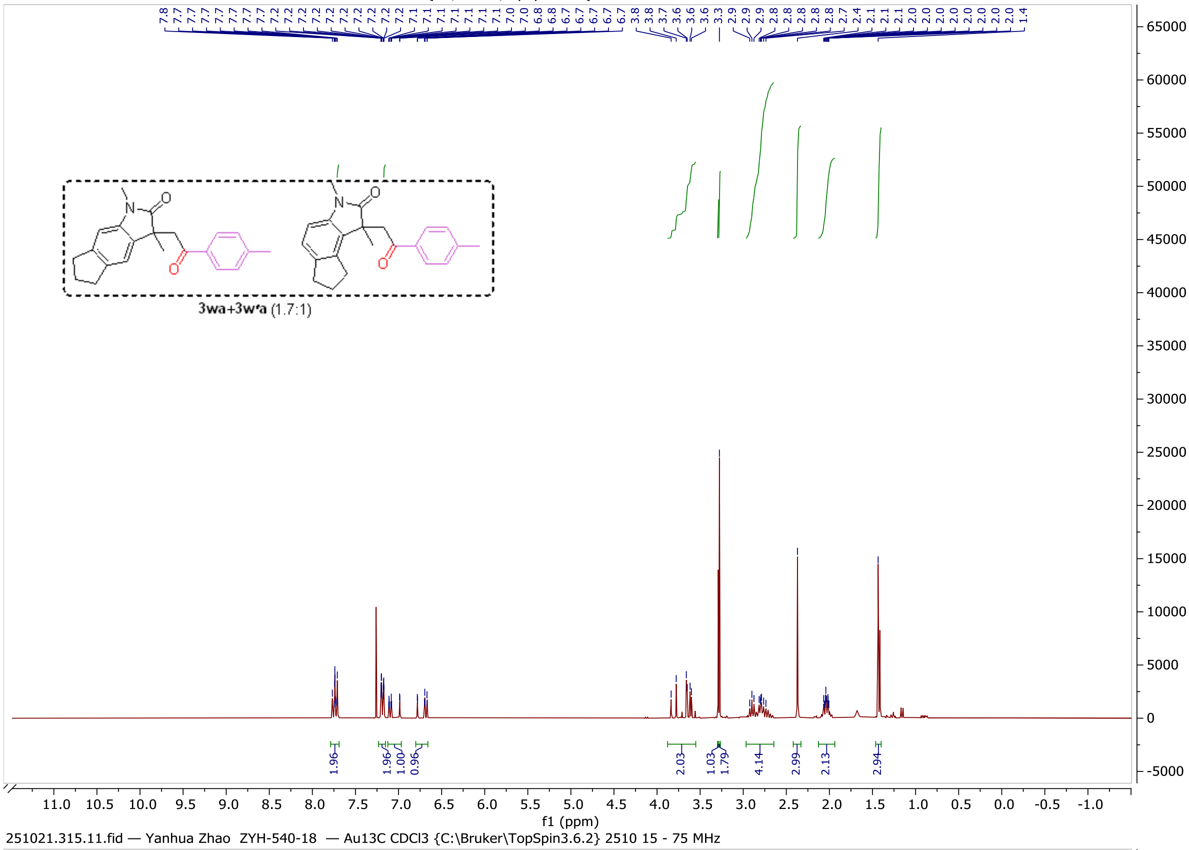

251021.315.11.fid — Yanhua Zhao ZYH-540-18 — Au13C CDCl3 {C:\Bruker\TopSpin3.6.2} 2510 15 - 75 MHz

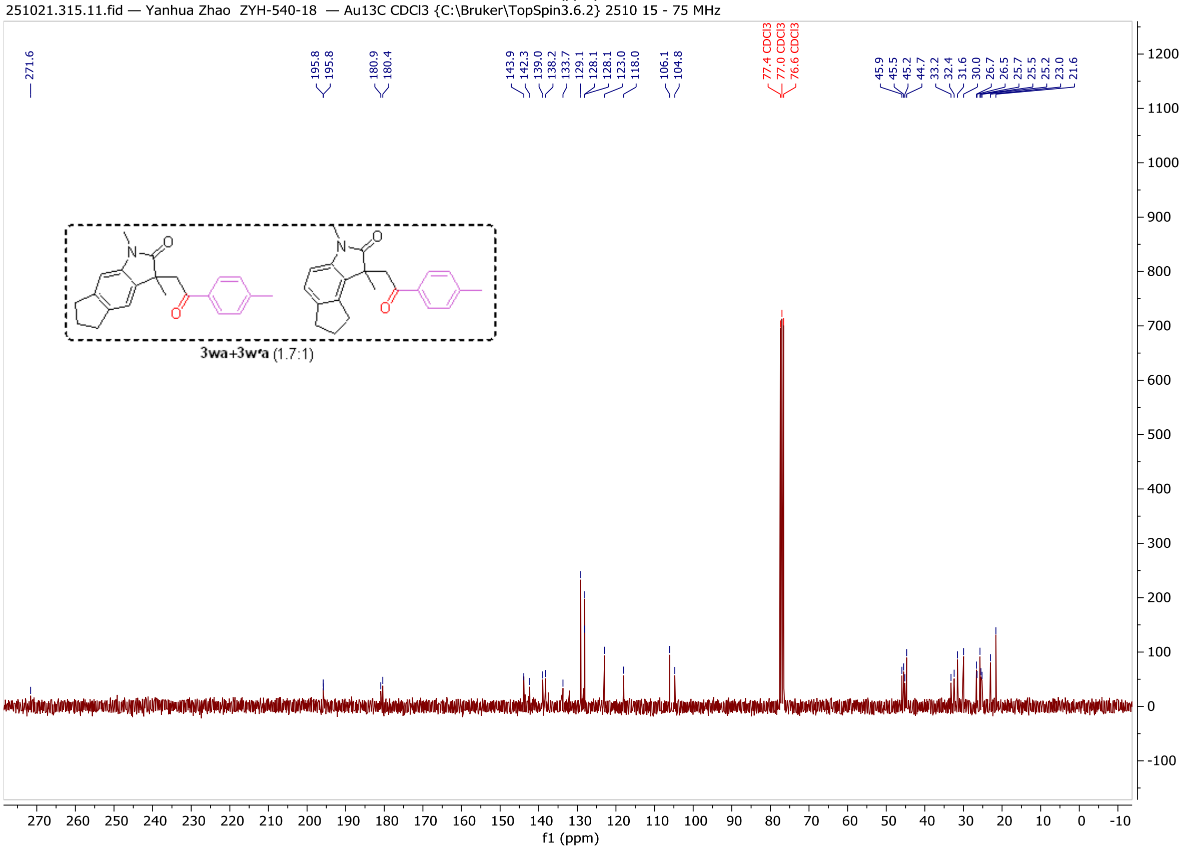

251023.f315.10.fid — Yanhua Zhao ZYH-544-7 — Au1H CDCl3 {C:\Bruker\TopSpin3.6.2} 2510 15 - 300 MHz

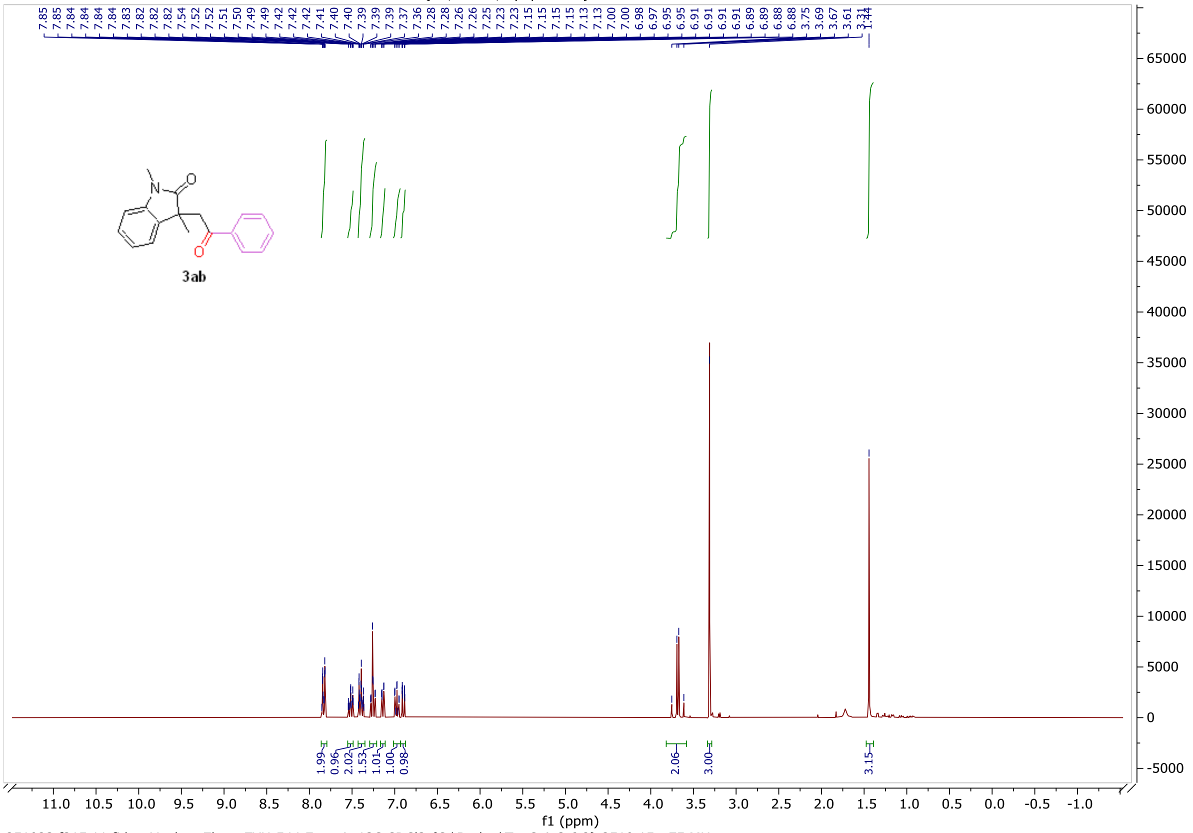

3ab

251023.f315.11.fid — Yanhua Zhao ZYH-544-7 — Au13C CDCl3 {C:\Bruker\TopSpin3.6.2} 2510 15 - 75 MHz

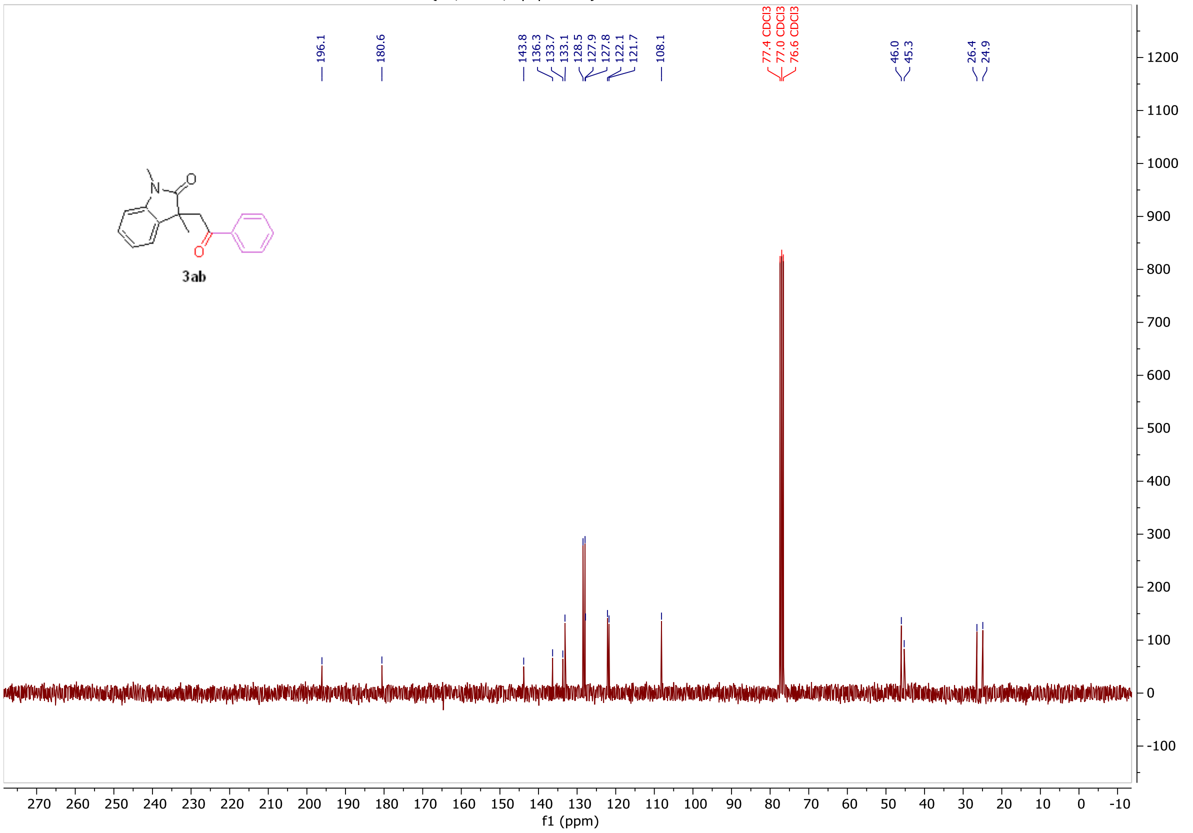

3ab

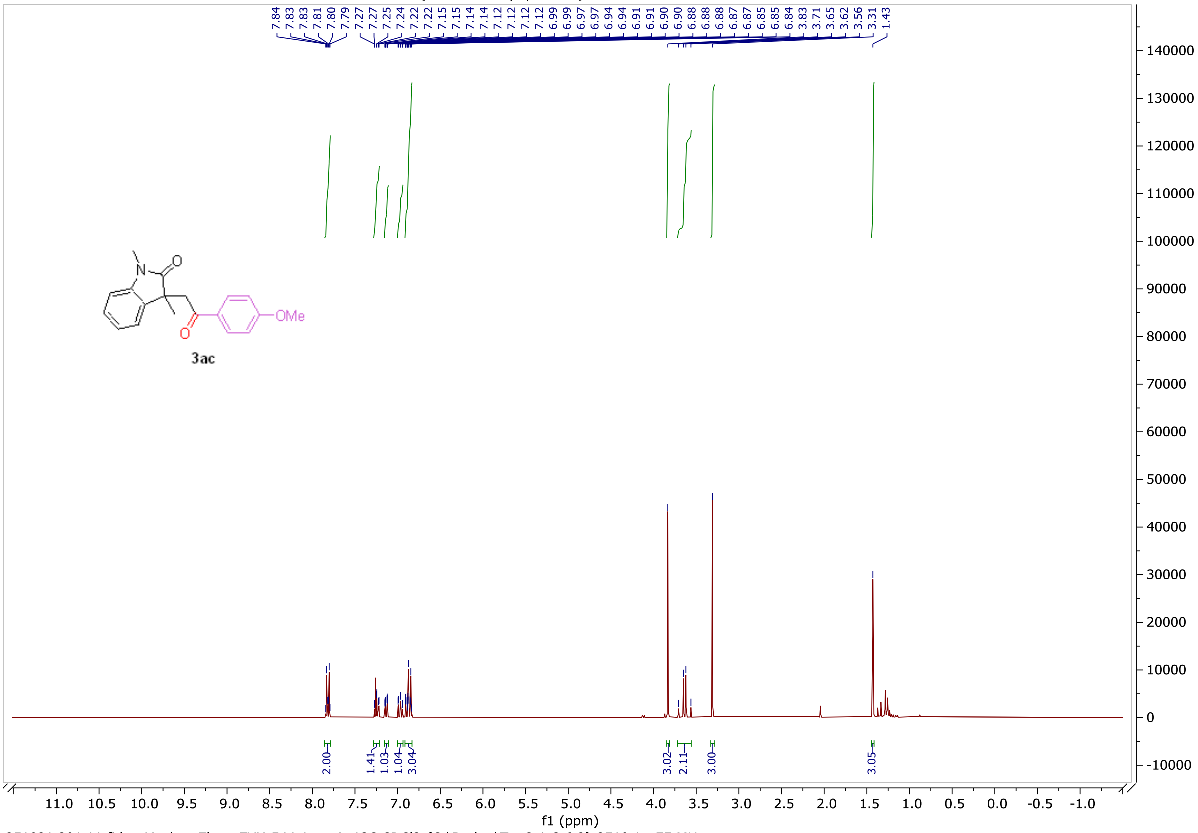

251021.301.10.fid — Yanhua Zhao ZYH-544-1 — Au1H CDCl3 {C:\Bruker\TopSpin3.6.2} 2510 1 - 300 MHz
7.84 7.83 7.83 7.81 7.80 7.79 7.27 7.27 7.25 7.24 7.24 7.22 7.22 7.15 7.15 7.14 7.14 7.12 7.12 7.12 6.99 6.99 6.97 6.97 6.94 6.94 6.91 6.91 6.90 6.90 6.88 6.88 6.88 6.87 6.87 6.85 6.85 6.84 3.83 3.71 3.65 3.62 3.56 3.31 1.43
3ac
OMe
2.00 1.41 1.03 1.04 3.04 3.02 2.11 3.00 3.05
f1 (ppm)

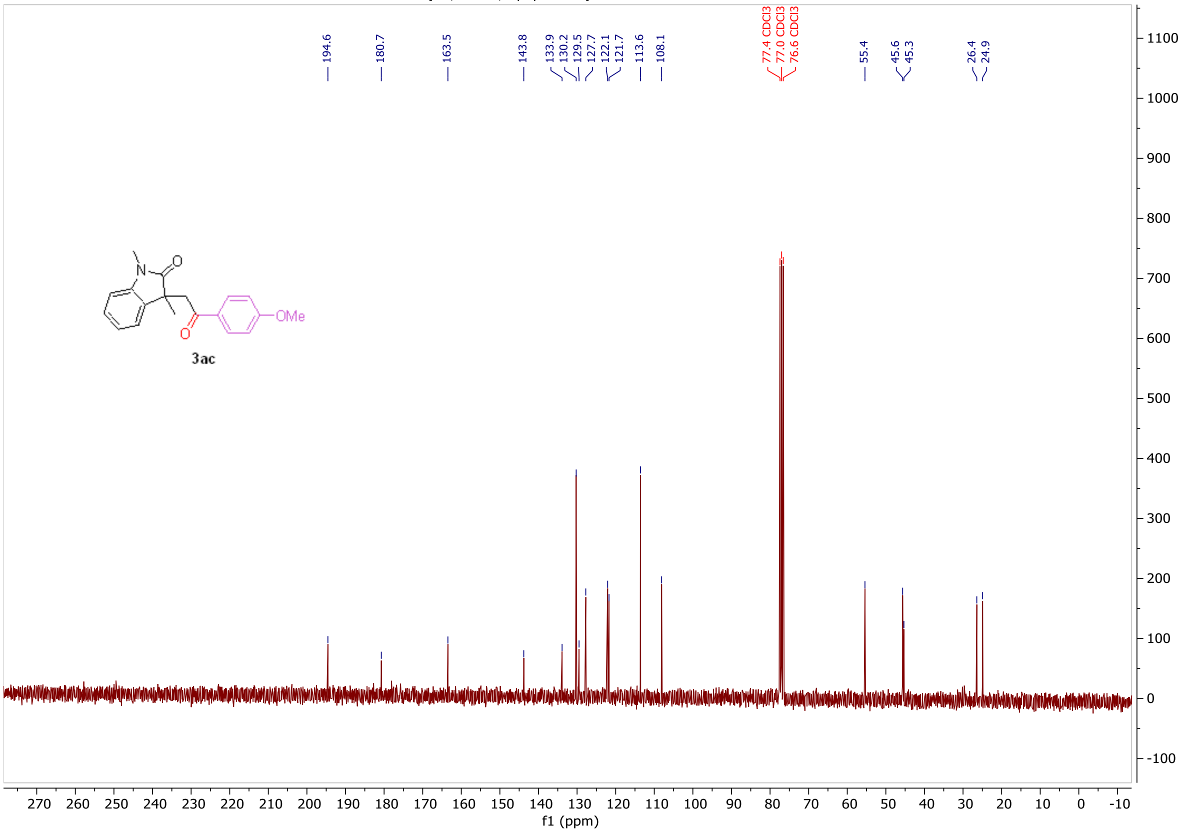

251021.301.11.fid — Yanhua Zhao ZYH-544-1 — Au13C CDCl3 {C:\Bruker\TopSpin3.6.2} 2510 1 - 75 MHz
194.6 180.7 163.5 143.8 133.9 130.2 129.5 127.7 122.1 121.7 113.6 108.1
77.4 CDCl3 77.0 CDCl3 76.6 CDCl3
55.4 45.6 45.3 26.4 24.9
3ac
OMe
f1 (ppm)

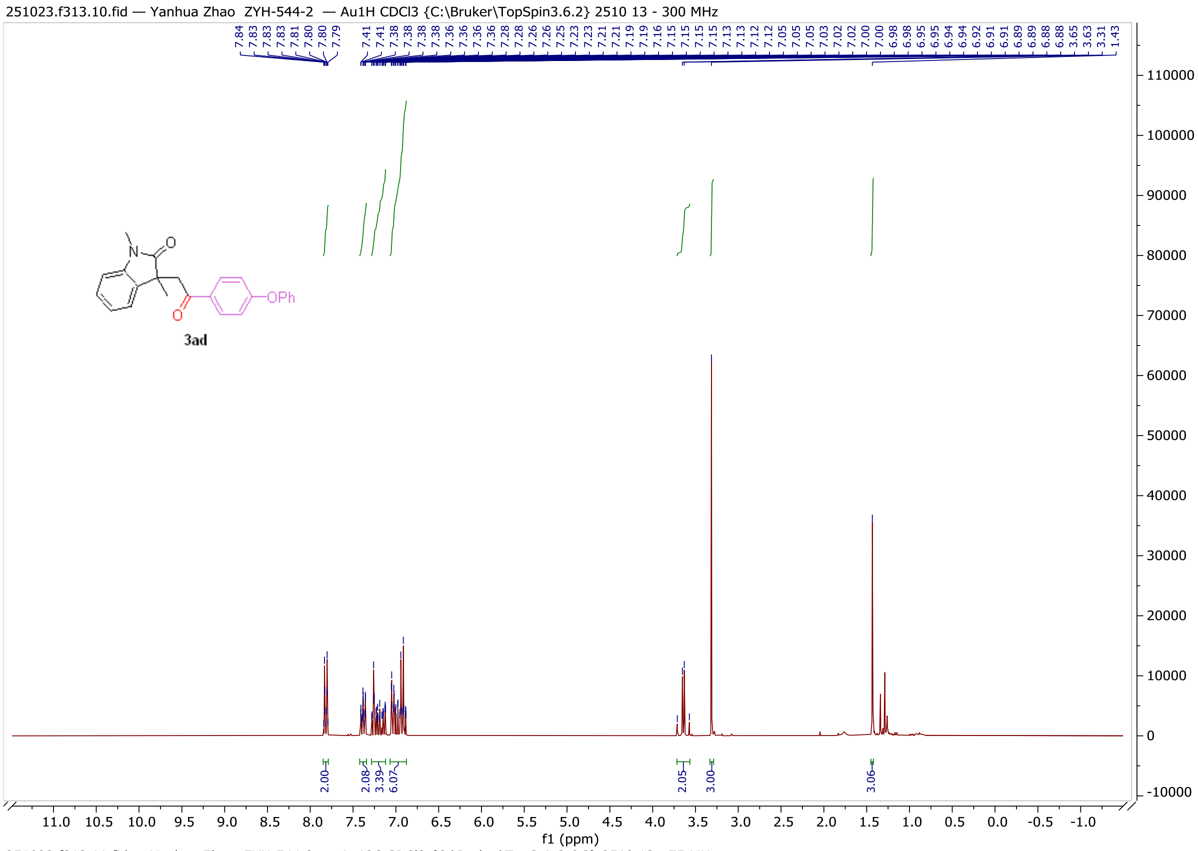
251023.f313.10.fid — Yanhua Zhao ZYH-544-2 — Au1H CDCl3 {C:\Bruker\TopSpin3.6.2} 2510 13 - 300 MHz
7.84 7.83 7.83 7.83 7.81 7.80 7.80 7.79 7.41 7.41 7.38 7.38 7.38 7.36 7.36 7.36 7.36 7.28 7.28 7.26 7.26 7.25 7.23 7.23 7.21 7.21 7.19 7.19 7.16 7.15 7.15 7.15 7.13 7.13 7.12 7.12 7.05 7.05 7.05 7.03 7.02 7.02 7.00 6.98 6.98 6.95 6.95 6.94 6.94 6.92 6.91 6.91 6.89 6.89 6.88 6.88 3.65 3.63 3.31 1.43
N
O
O
OPh
3ad
2.00
2.08
3.39
6.07
2.05
3.00
3.06
f1 (ppm)

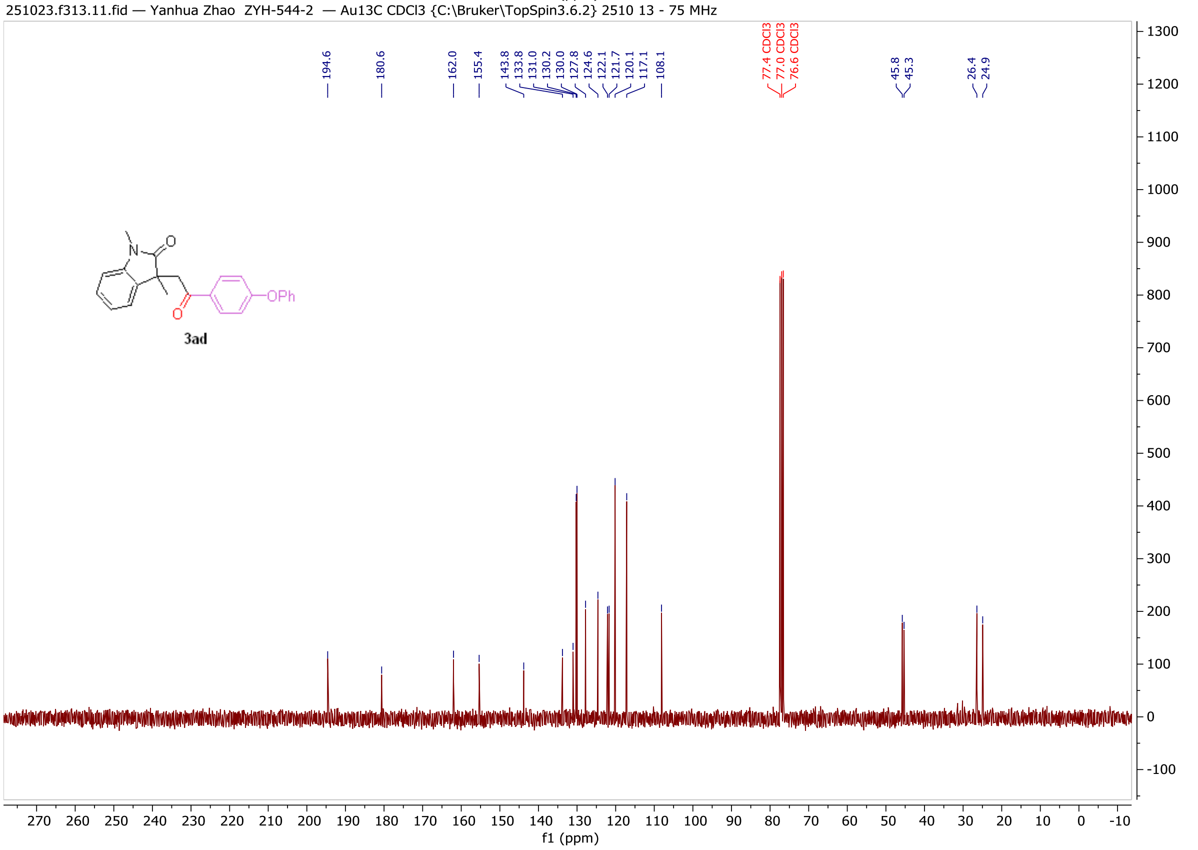
251023.f313.11.fid — Yanhua Zhao ZYH-544-2 — Au13C CDCl3 {C:\Bruker\TopSpin3.6.2} 2510 13 - 75 MHz
194.6
180.6
162.0
155.4
143.8 133.8 131.0 130.2 130.0 127.8 124.6 122.1 121.7 120.1 117.1
108.1
77.4 CDCl3 77.0 CDCl3 76.6 CDCl3
45.8 45.3
26.4 24.9
N
O
O
OPh
3ad
f1 (ppm)

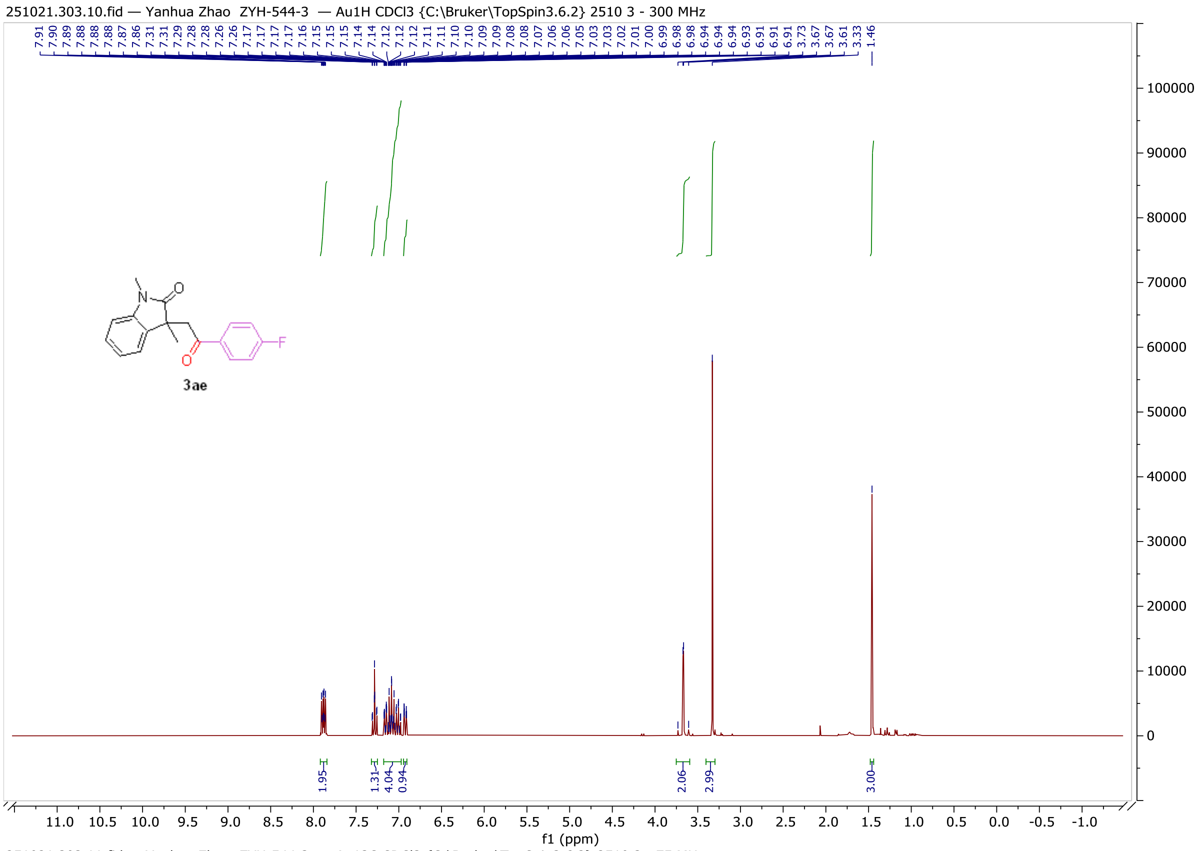
251021.303.10.fid — Yanhua Zhao ZYH-544-3 — Au1H CDCl3 {C:\Bruker\TopSpin3.6.2} 2510 3 - 300 MHz
3ae
1.95
1.31
4.04
0.94
2.06
2.99
3.00
f1 (ppm)

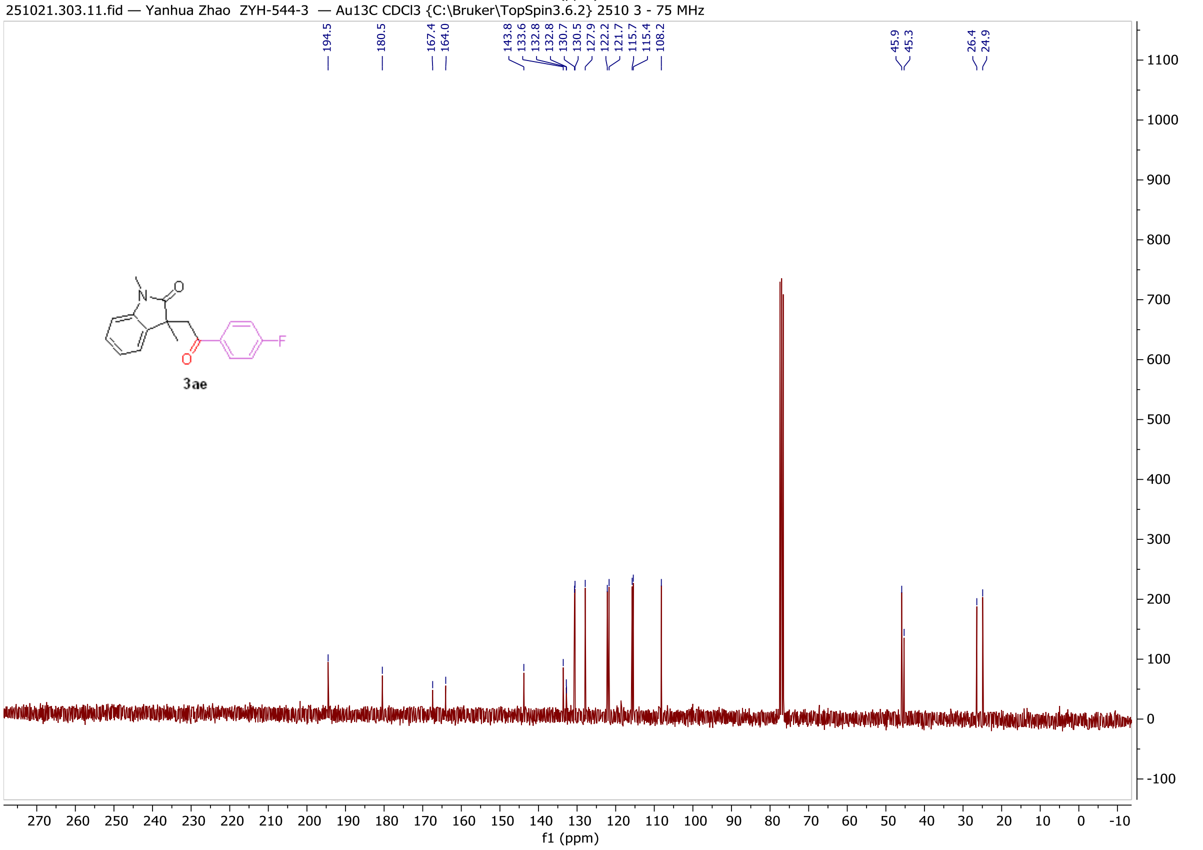
251021.303.11.fid — Yanhua Zhao ZYH-544-3 — Au13C CDCl3 {C:\Bruker\TopSpin3.6.2} 2510 3 - 75 MHz
194.5
180.5
167.4
164.0
143.8
133.6
132.8
132.8
130.7
130.5
127.9
122.2
121.7
115.7
115.4
108.2
45.9
45.3
26.4
24.9
3ae
f1 (ppm)

251021.303.12.fid — Yanhua Zhao ZYH-544-3 — Au19F CDCl3 {C:\Bruker\TopSpin3.6.2} 2510 3-282 MHz

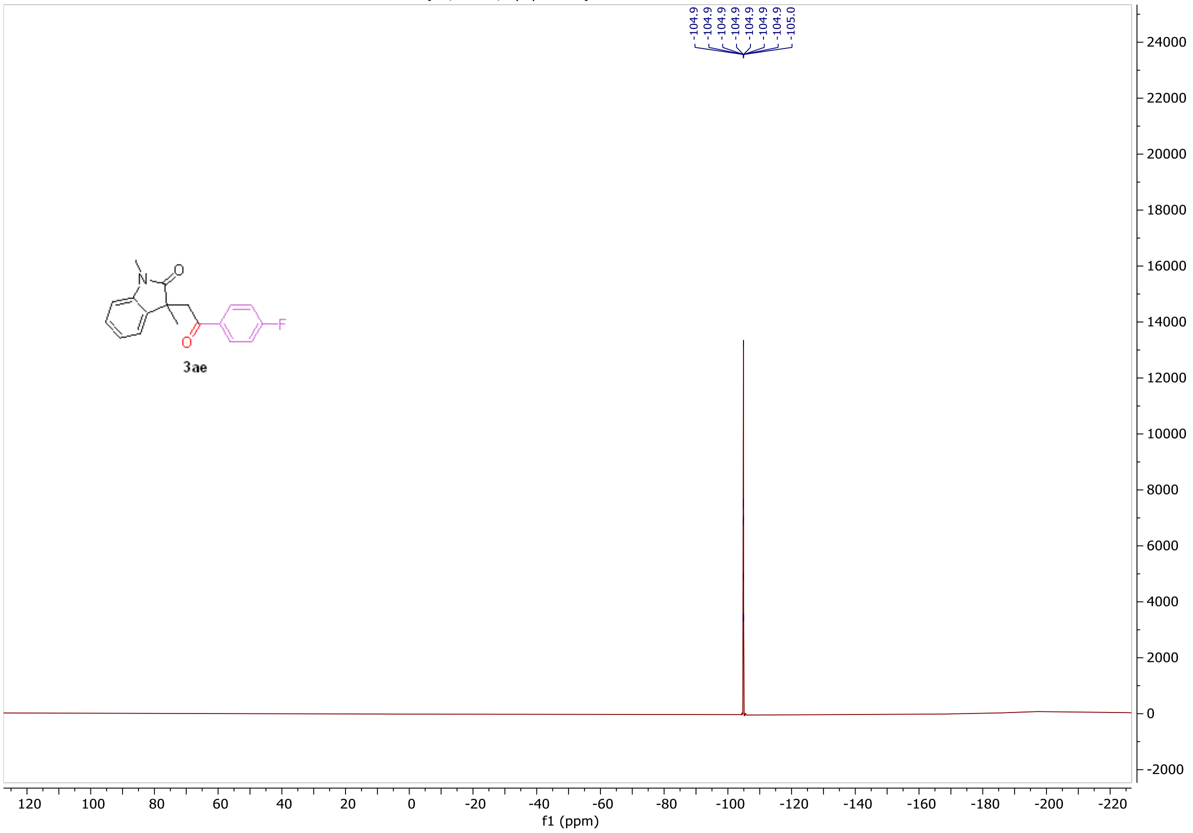

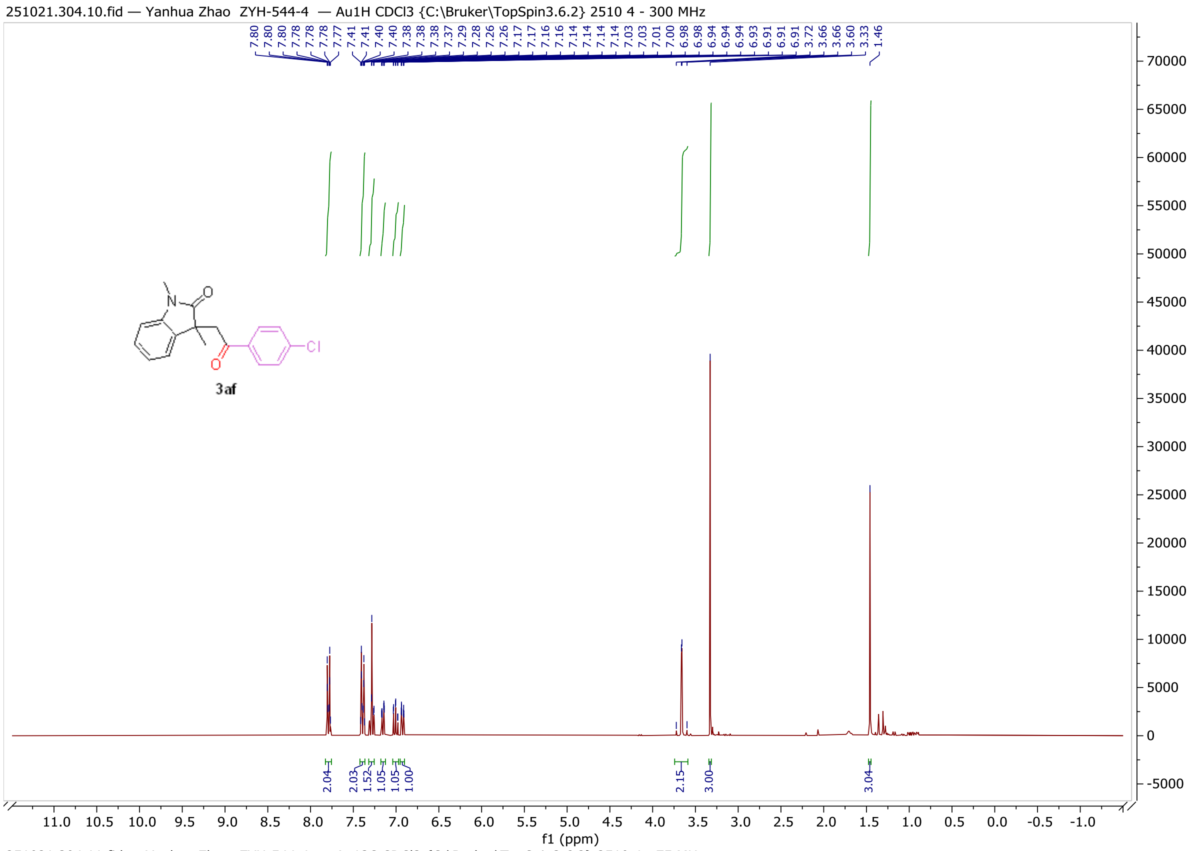

251021.304.10.fid — Yanhua Zhao ZYH-544-4 — Au1H CDCl3 {C:\Bruker\TopSpin3.6.2} 2510 4 - 300 MHz
3af
f1 (ppm)

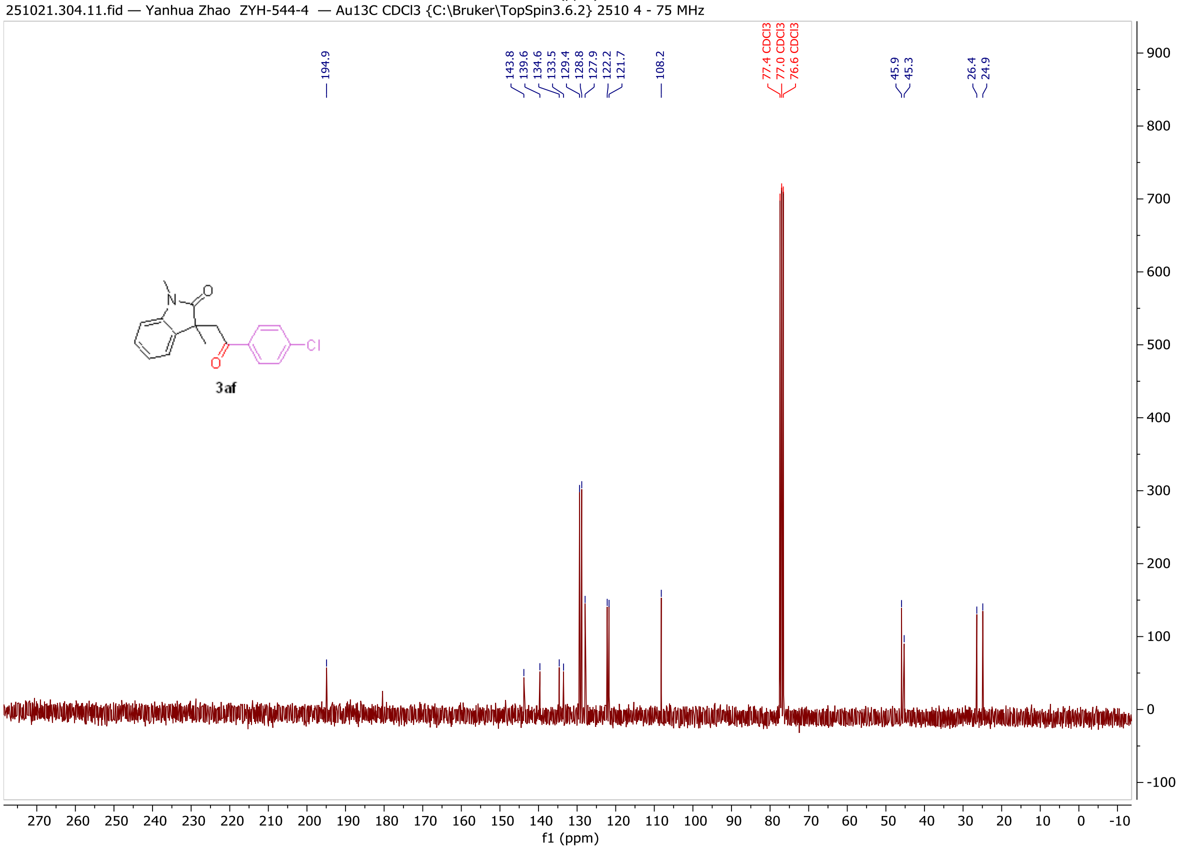

251021.304.11.fid — Yanhua Zhao ZYH-544-4 — Au13C CDCl3 {C:\Bruker\TopSpin3.6.2} 2510 4 - 75 MHz
194.9
143.8
139.6
134.6
133.5
129.4
128.8
127.9
122.2
121.7
108.2
77.4 CDCl3
77.0 CDCl3
76.6 CDCl3
45.9
45.3
26.4
24.9
3af
f1 (ppm)

251030.f314.10.fid — Yanhua Zhao ZYH-544-5 — Au1H CDCl3 {C:\Bruker\TopSpin3.6.2} 2510 14 - 300 MHz

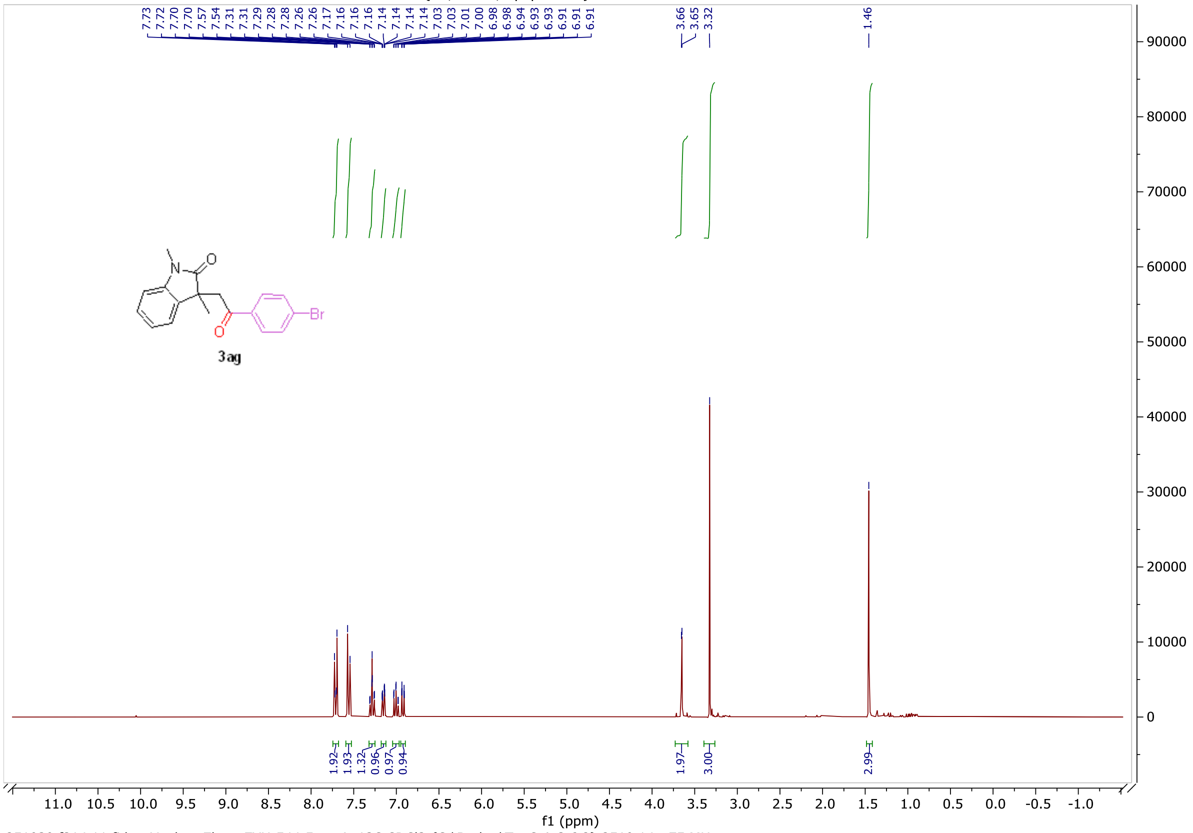

251030.f314.11.fid — Yanhua Zhao ZYH-544-5 — Au13C CDCl3 {C:\Bruker\TopSpin3.6.2} 2510 14 - 75 MHz

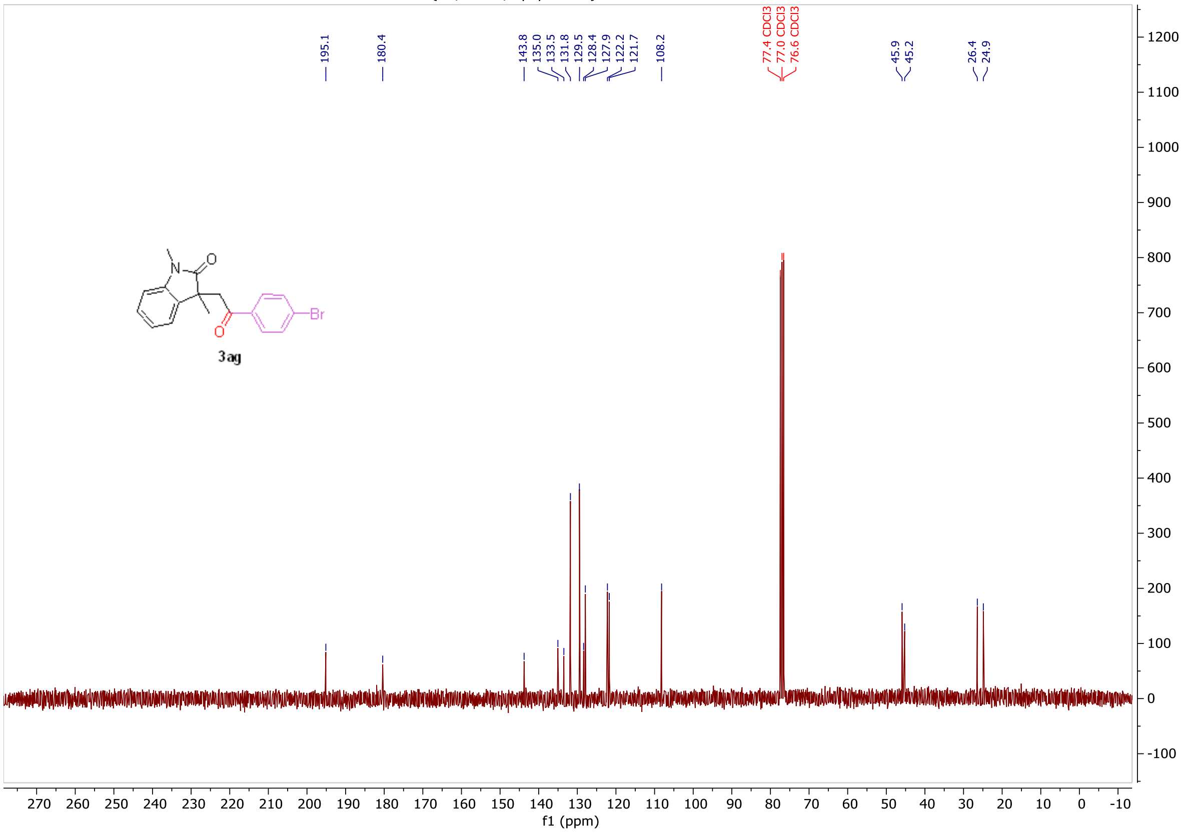

251023.f316.10.fid — Yanhua Zhao ZYH-544-8 — Au1H CDCl3 {C:\Bruker\TopSpin3.6.2} 2510 16 - 300 MHz

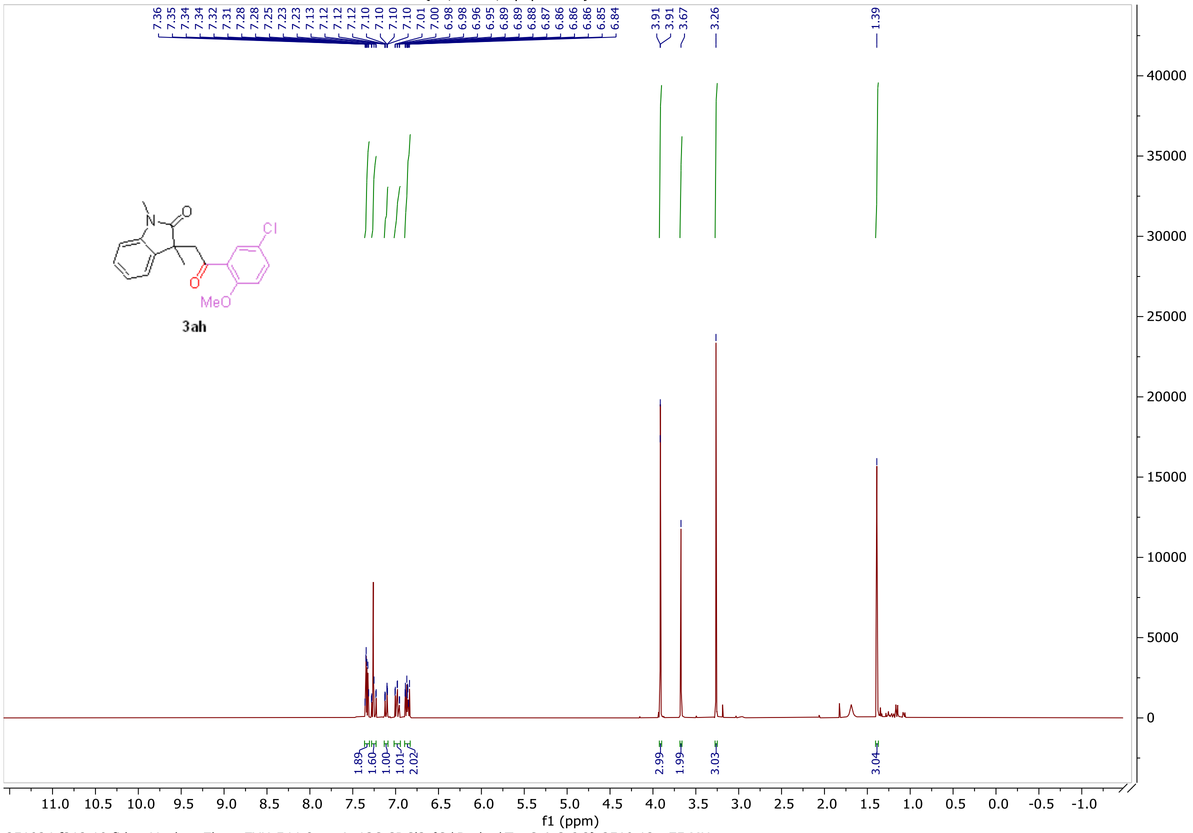

251024.f312.10.fid — Yanhua Zhao ZYH-544-8 — Au13C CDCl3 {C:\Bruker\TopSpin3.6.2} 2510 12 - 75 MHz

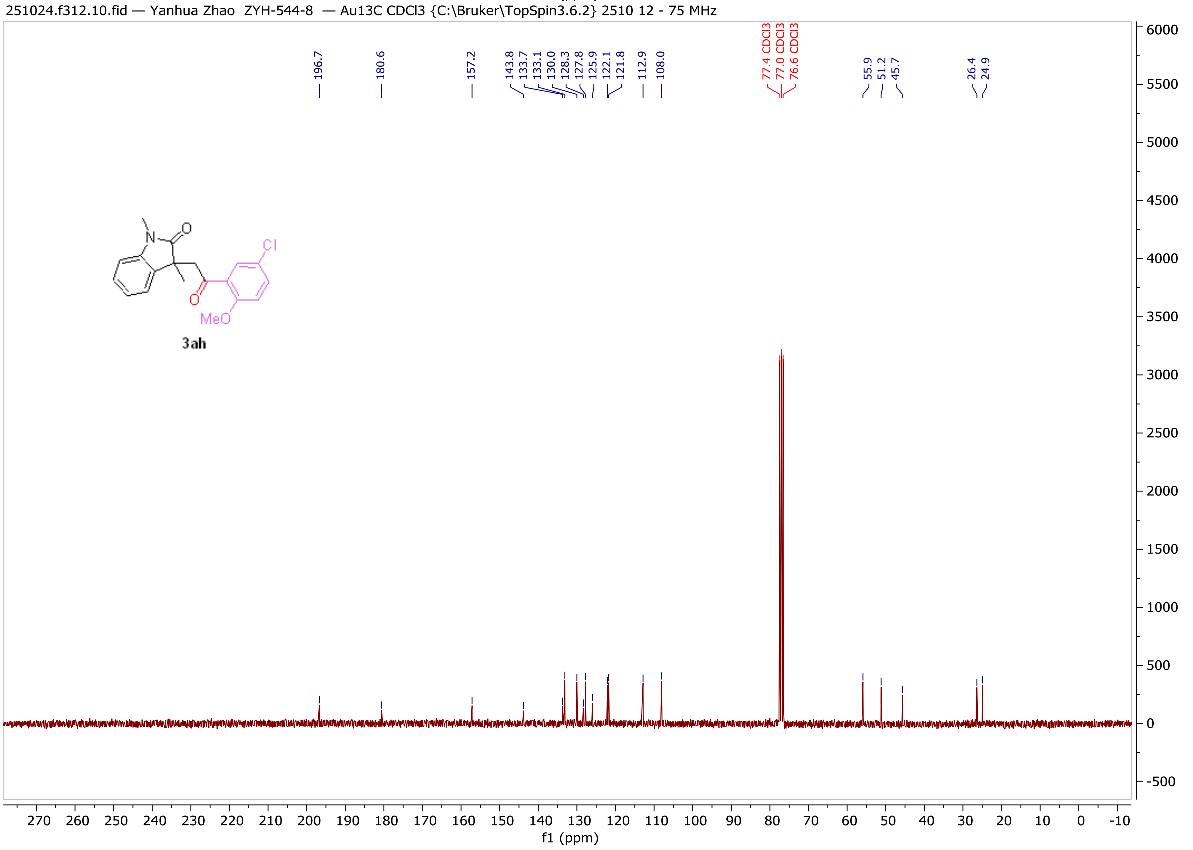

251023.f317.10.fid — Yanhua Zhao ZYH-544-10 — Au1H CDCl3 {C:\Bruker\TopSpin3.6.2} 2510 17 - 300 MHz

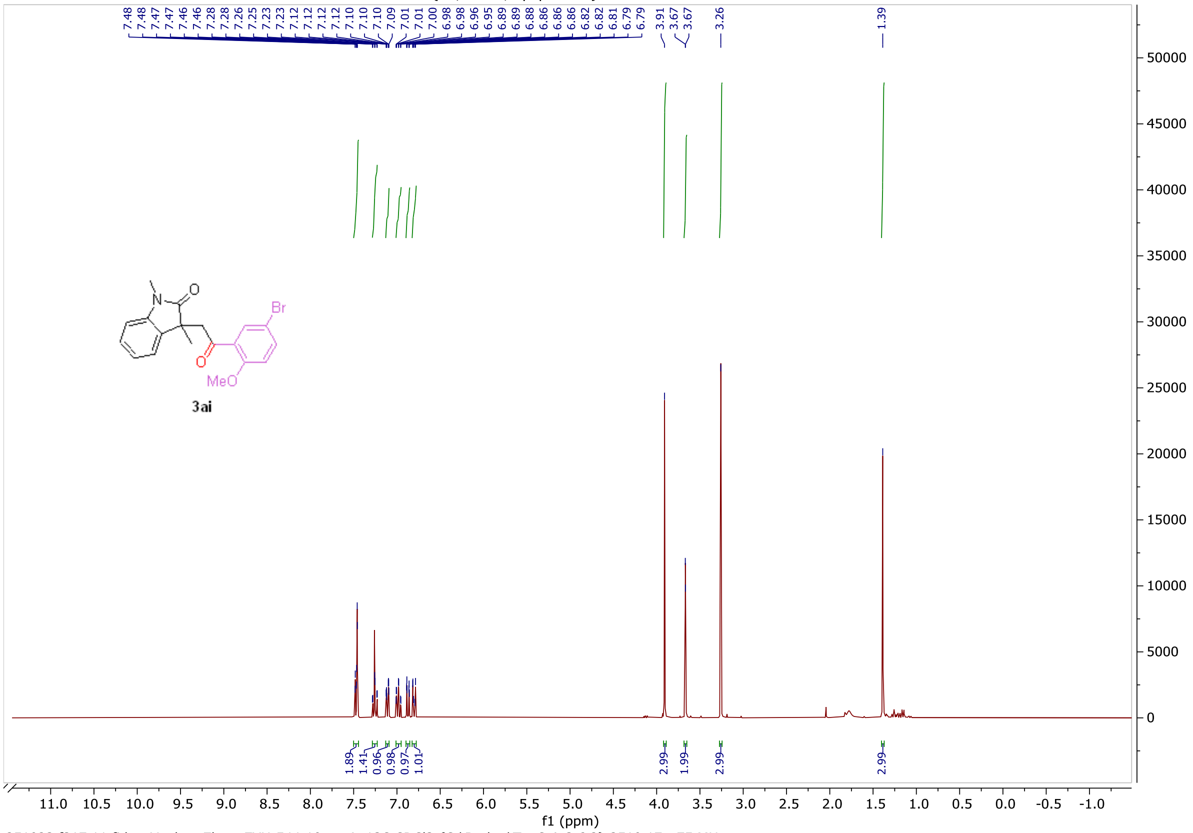

251023.f317.11.fid — Yanhua Zhao ZYH-544-10 — Au13C CDCl3 {C:\Bruker\TopSpin3.6.2} 2510 17 - 75 MHz

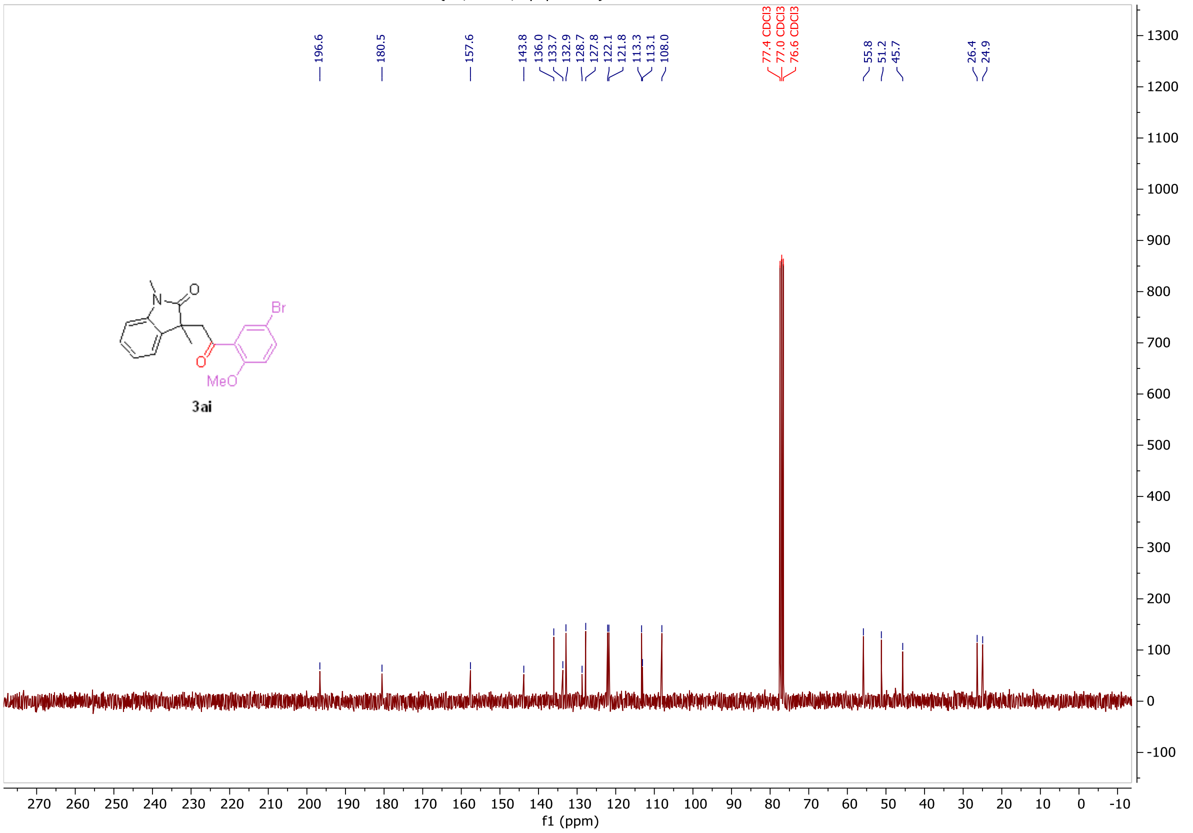

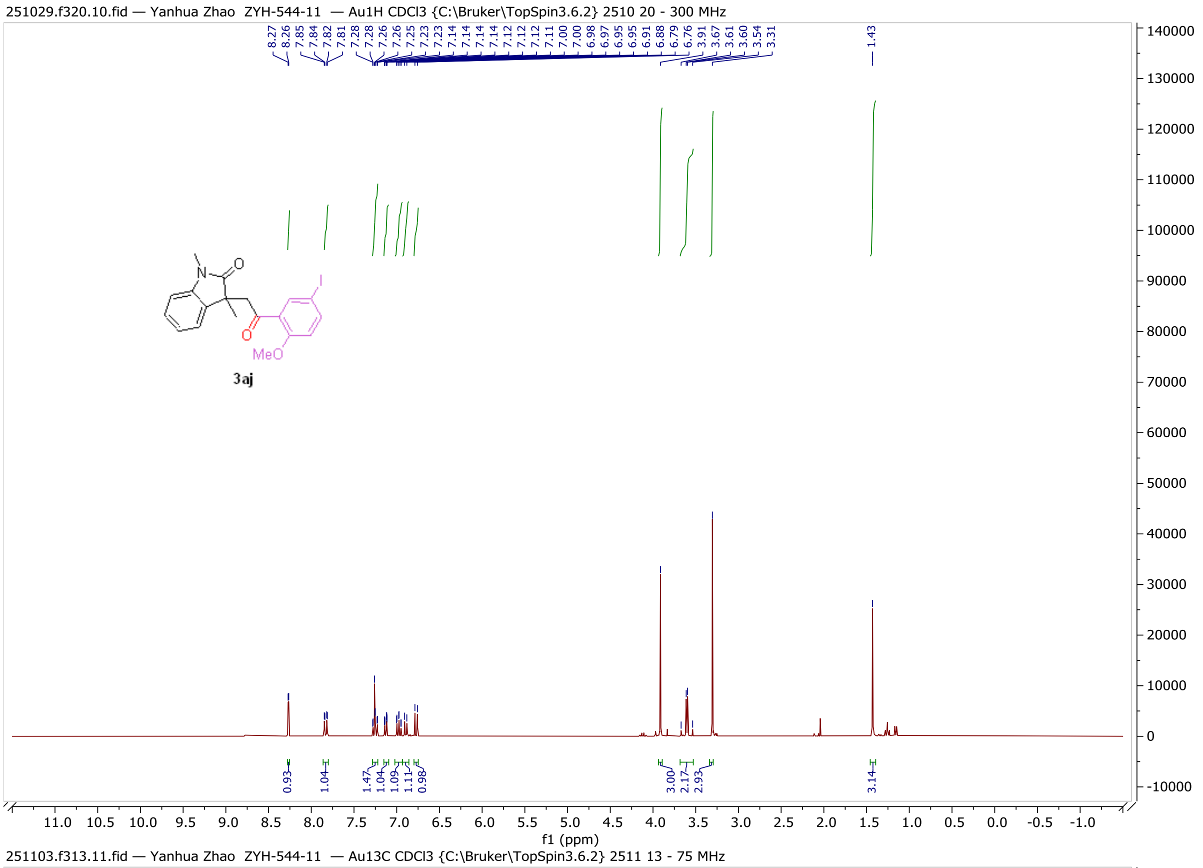
251029.f320.10.fid — Yanhua Zhao ZYH-544-11 — Au1H CDCl3 {C:\Bruker\TopSpin3.6.2} 2510 20 - 300 MHz
8.27 8.26 7.85 7.84 7.82 7.81 7.28 7.28 7.26 7.26 7.25 7.23 7.23 7.14 7.14 7.14 7.14 7.12 7.12 7.12 7.11 7.00 7.00 6.98 6.97 6.95 6.95 6.91 6.88 6.79 6.76 3.91 3.67 3.61 3.60 3.54 3.31
1.43
0.93 1.04 1.47 1.04 1.09 1.11 0.98 3.00 2.17 2.93 3.14
MeO
3aj
f1 (ppm)

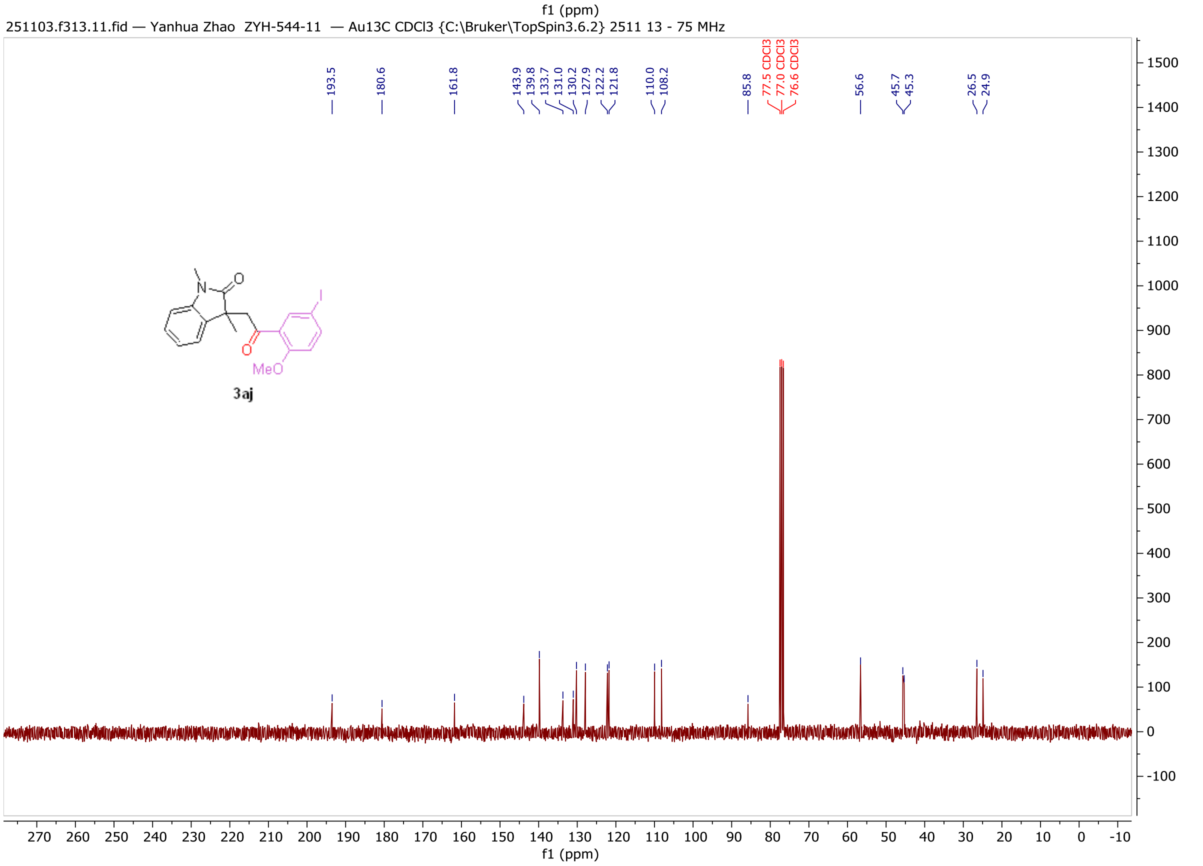
251103.f313.11.fid — Yanhua Zhao ZYH-544-11 — Au13C CDCl3 {C:\Bruker\TopSpin3.6.2} 2511 13 - 75 MHz
193.5 180.6 161.8 143.9 139.8 133.7 131.0 130.2 127.9 122.2 121.8 110.0 108.2 85.8
77.5 CDCl3 77.0 CDCl3 76.6 CDCl3
56.6 45.7 45.3 26.5 24.9
MeO
3aj
f1 (ppm)

251023.f319.10.fid — Yanhua Zhao ZYH-544-12 — Au1H CDCl3 {C:\Bruker\TopSpin3.6.2} 2510 19 - 300 MHz

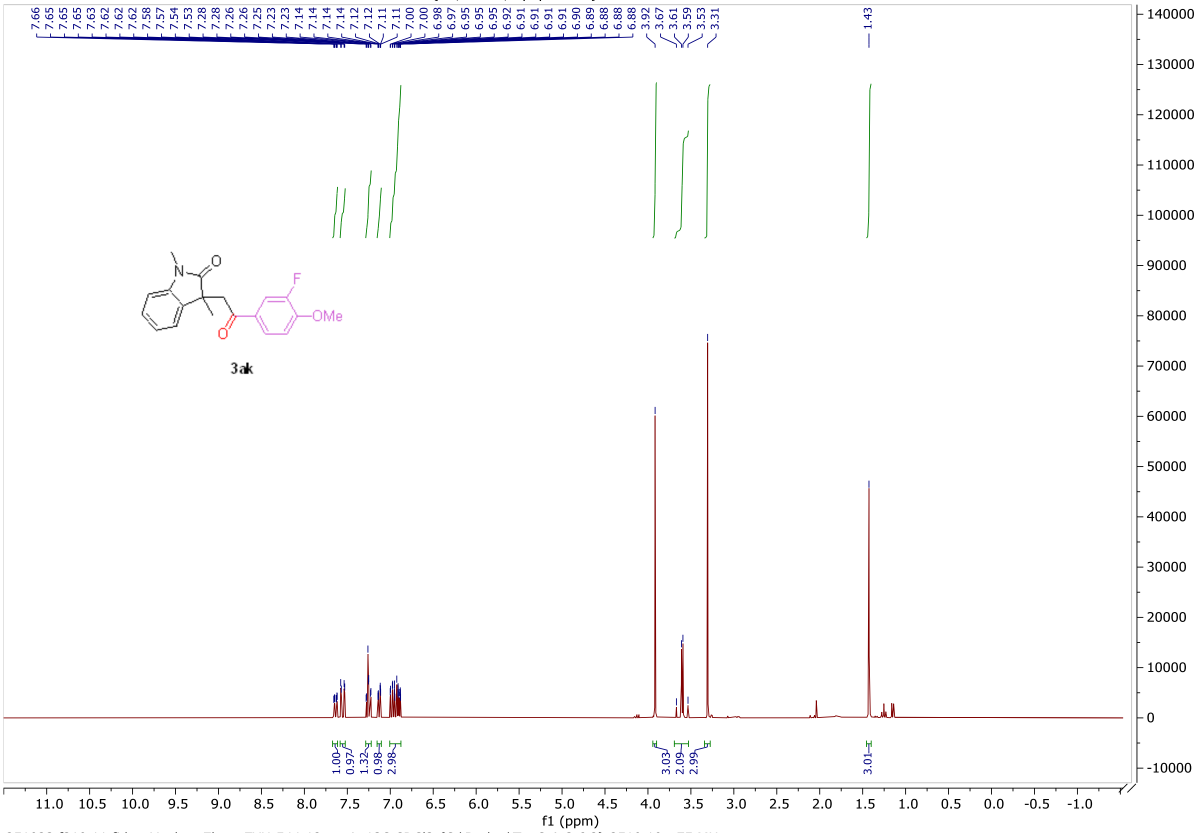

251023.f319.11.fid — Yanhua Zhao ZYH-544-12 — Au13C CDCl3 {C:\Bruker\TopSpin3.6.2} 2510 19 - 75 MHz

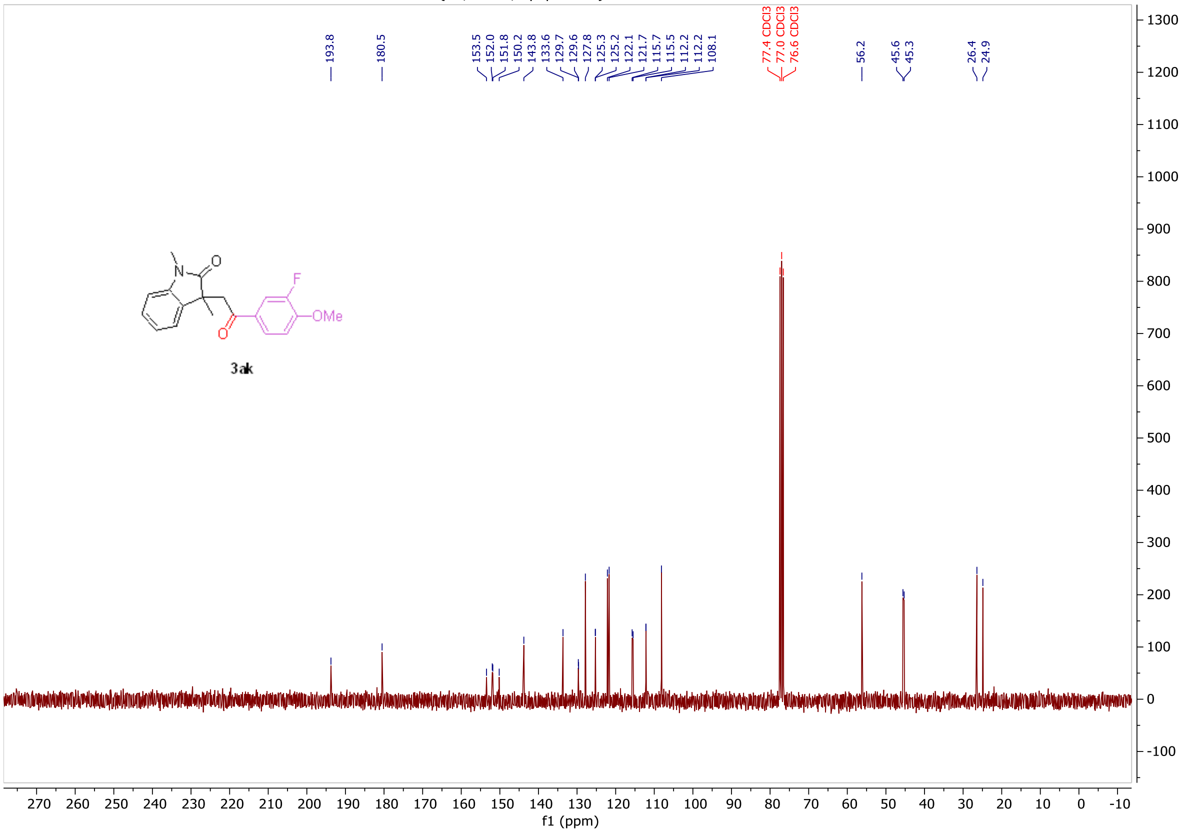

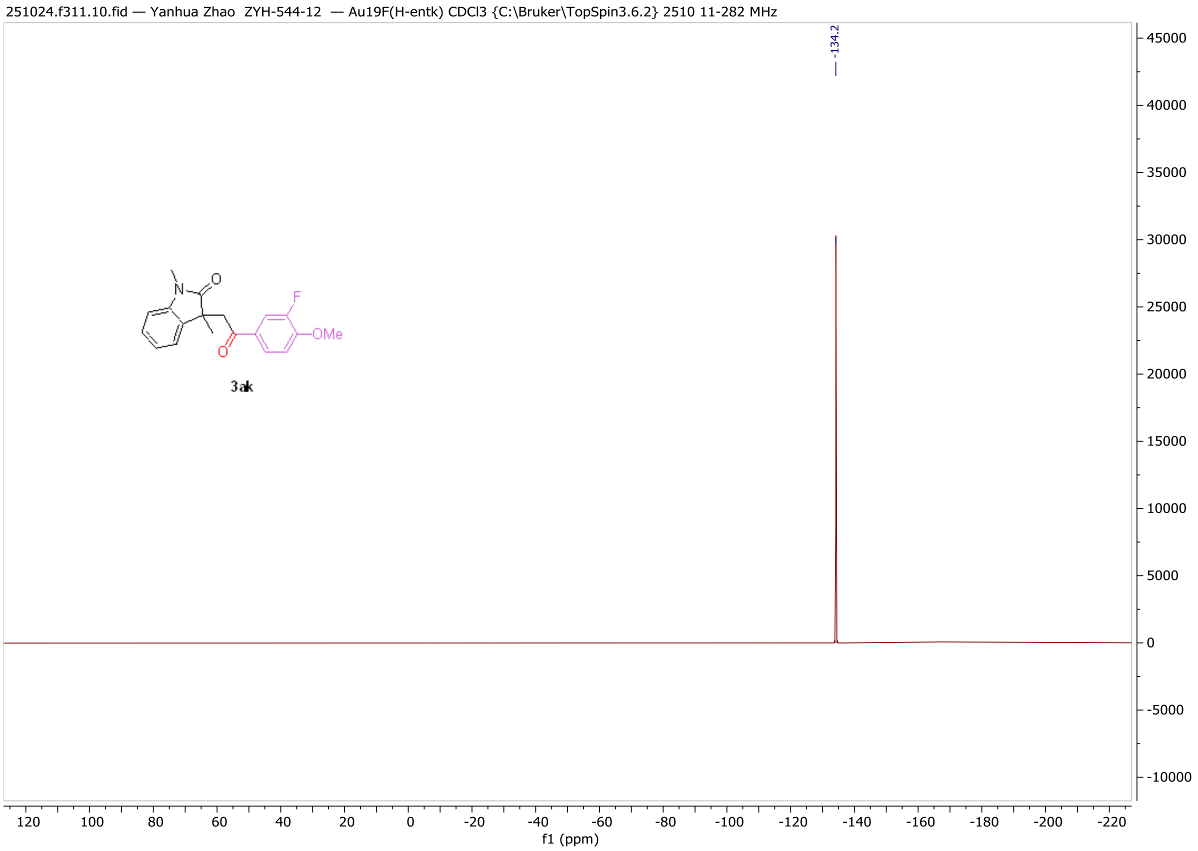
251024.f311.10.fid — Yanhua Zhao ZYH-544-12 — Au19F(H-entk) CDCl3 {C:\Bruker\TopSpin3.6.2} 2510 11-282 MHz
-134.2
3ak
f1 (ppm)

251024.327.10.fid — Yanhua Zhao ZYH-544-13 — Au1H CDCl3 {C:\Bruker\TopSpin3.6.2} 2510 27 - 300 MHz

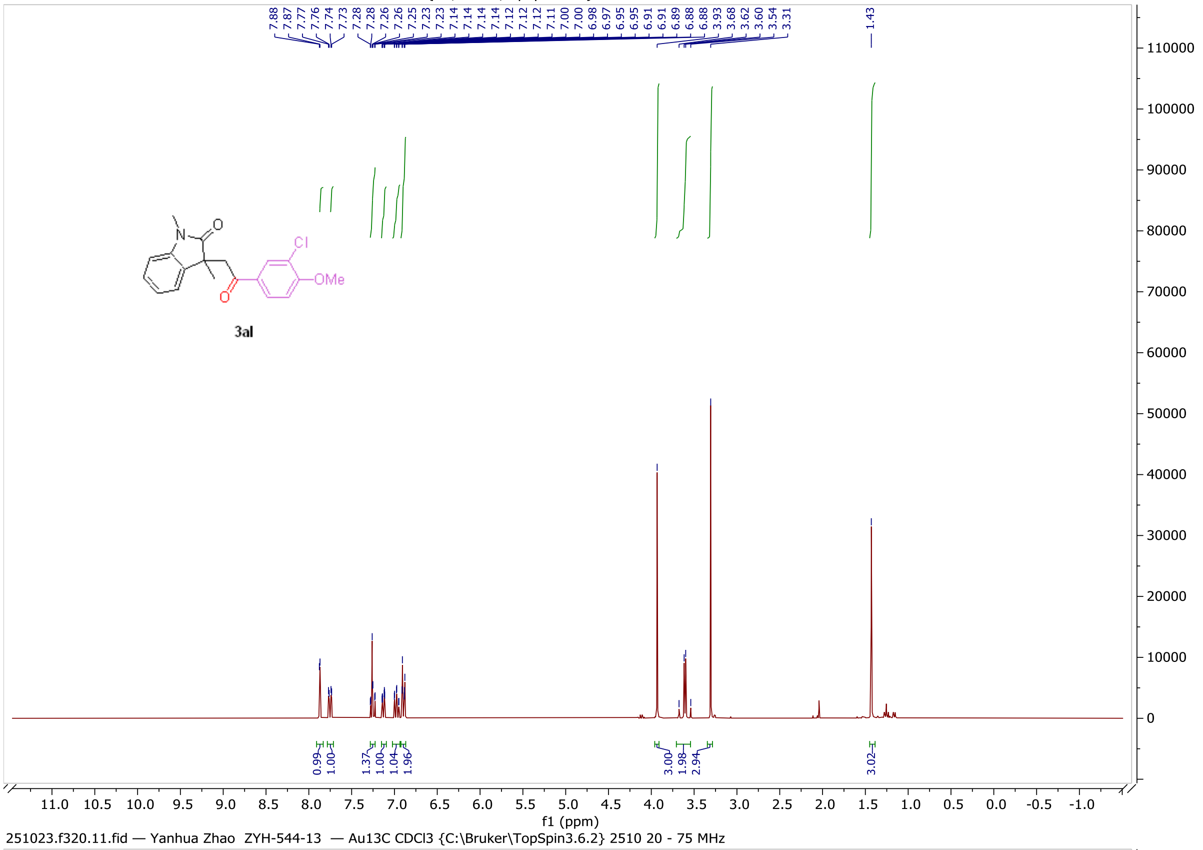

251023.f320.11.fid — Yanhua Zhao ZYH-544-13 — Au13C CDCl3 {C:\Bruker\TopSpin3.6.2} 2510 20 - 75 MHz

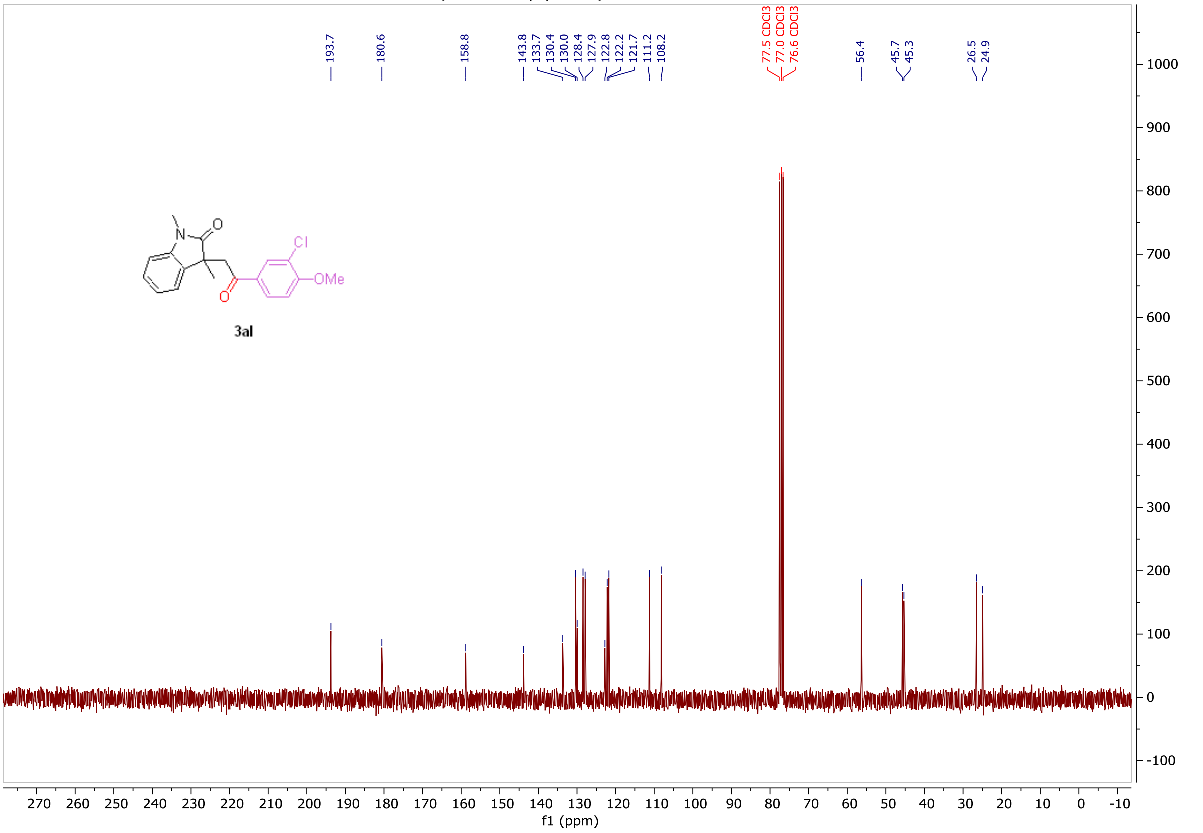

251104.f316.10.fid — Yanhua Zhao ZYH-544-14 — Au1H CDCl3 {C:\Bruker\TopSpin3.6.2} 2511 16 - 300 MHz

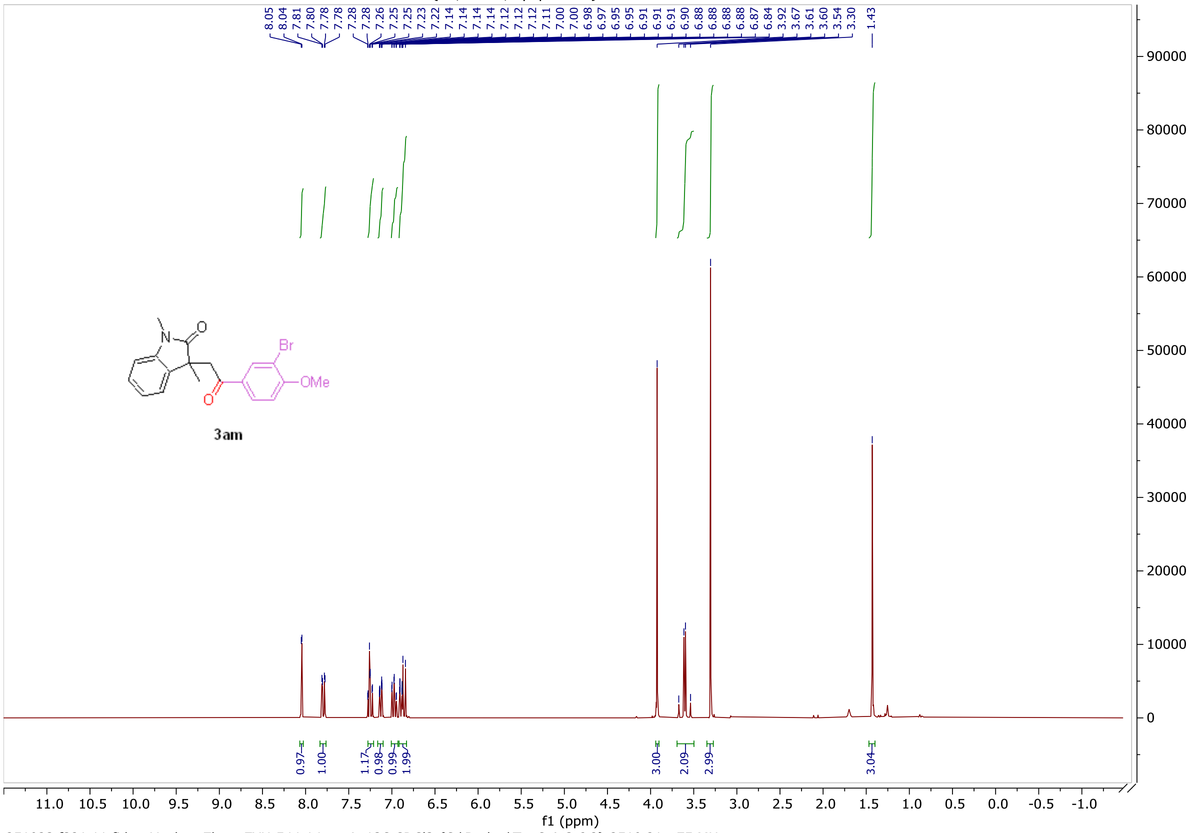

251023.f321.11.fid — Yanhua Zhao ZYH-544-14 — Au13C CDCl3 {C:\Bruker\TopSpin3.6.2} 2510 21 - 75 MHz

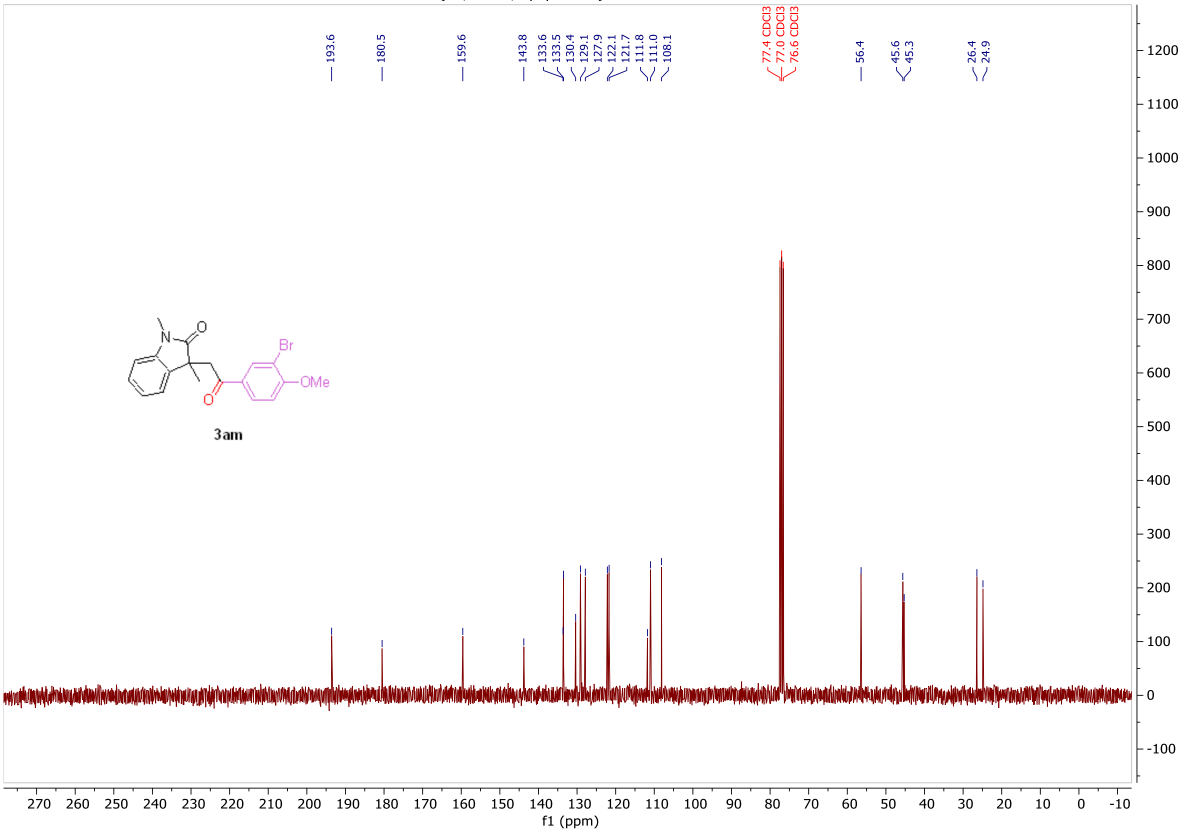

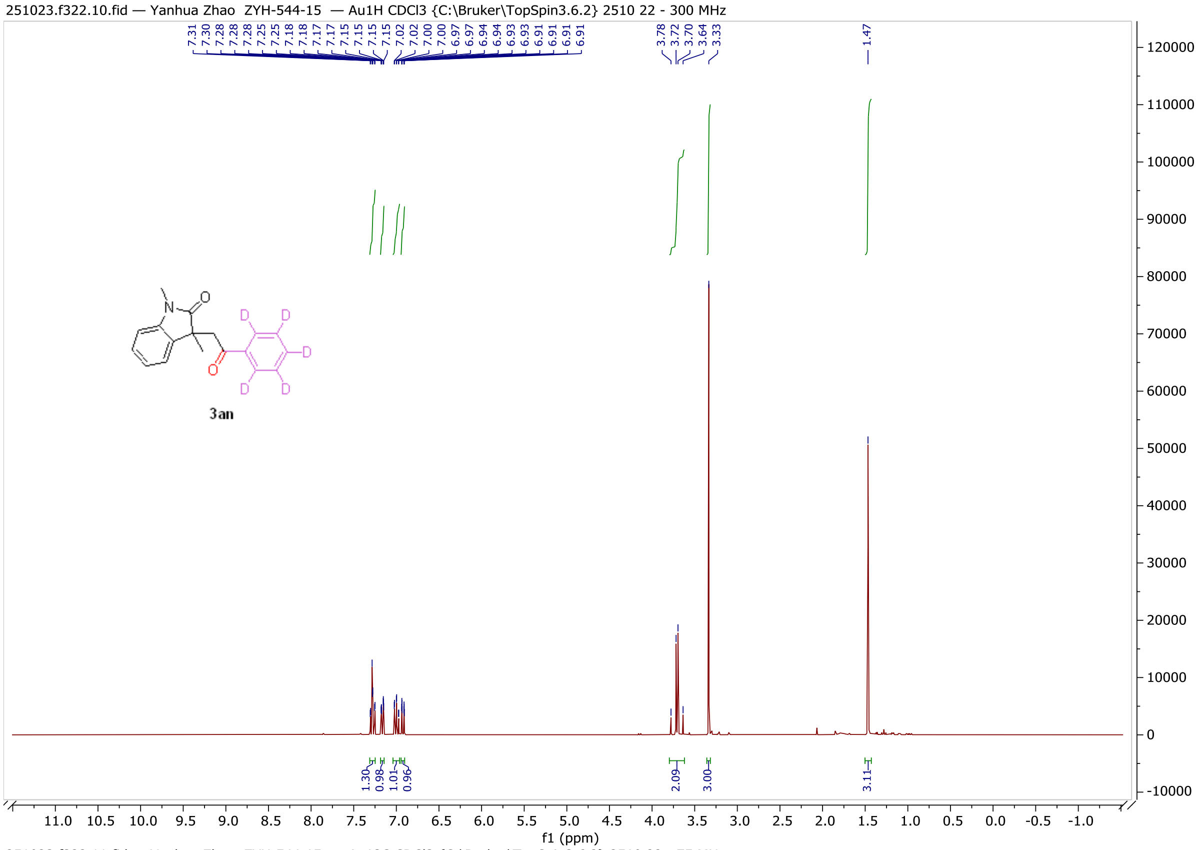
251023.f322.10.fid — Yanhua Zhao ZYH-544-15 — Au1H CDCl3 {C:\Bruker\TopSpin3.6.2} 2510 22 - 300 MHz
7.31 7.30 7.28 7.28 7.25 7.25 7.18 7.18 7.17 7.17 7.15 7.15 7.15 7.15 7.02 7.02 7.00 7.00 6.97 6.97 6.94 6.94 6.93 6.93 6.91 6.91 6.91 6.91 6.91
3.78 3.72 3.70 3.64 3.33
1.47
1.30 0.98 1.01 0.96
2.09 3.00 3.11
3an
f1 (ppm)

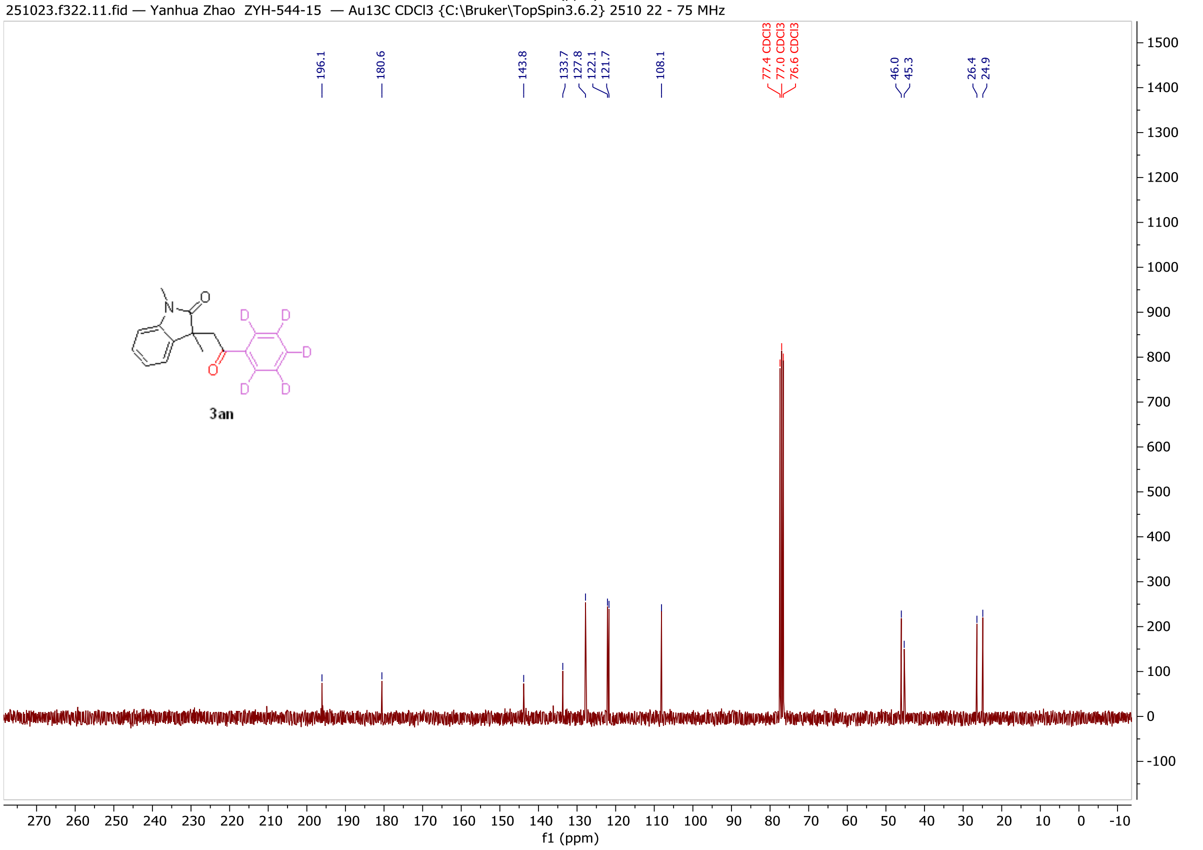
251023.f322.11.fid — Yanhua Zhao ZYH-544-15 — Au13C CDCl3 {C:\Bruker\TopSpin3.6.2} 2510 22 - 75 MHz
196.1
180.6
143.8
133.7 127.8 122.1 121.7
108.1
77.4 CDCl3 77.0 CDCl3 76.6 CDCl3
46.0 45.3
26.4 24.9
3an
f1 (ppm)

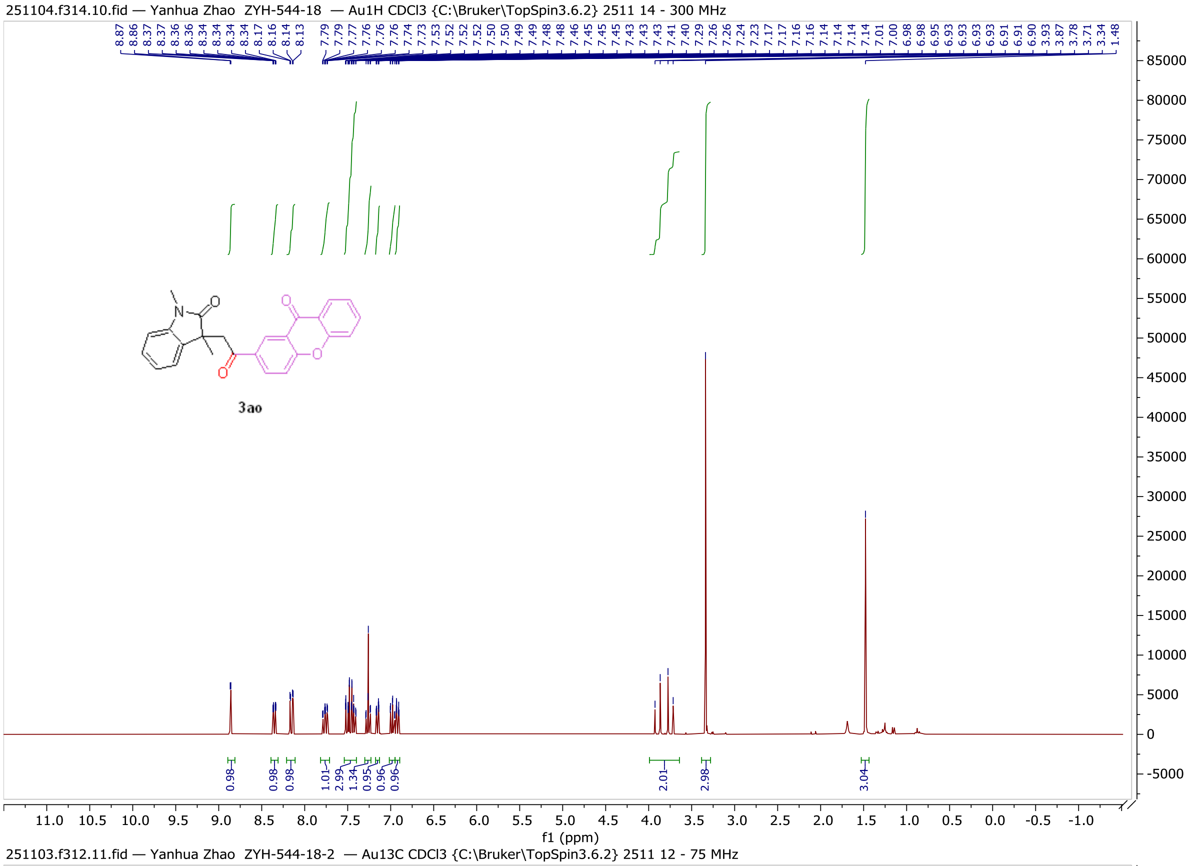
251104.f314.10.fid — Yanhua Zhao ZYH-544-18 — Au1H CDCl3 {C:\Bruker\TopSpin3.6.2} 2511 14 - 300 MHz
3ao
f1 (ppm)

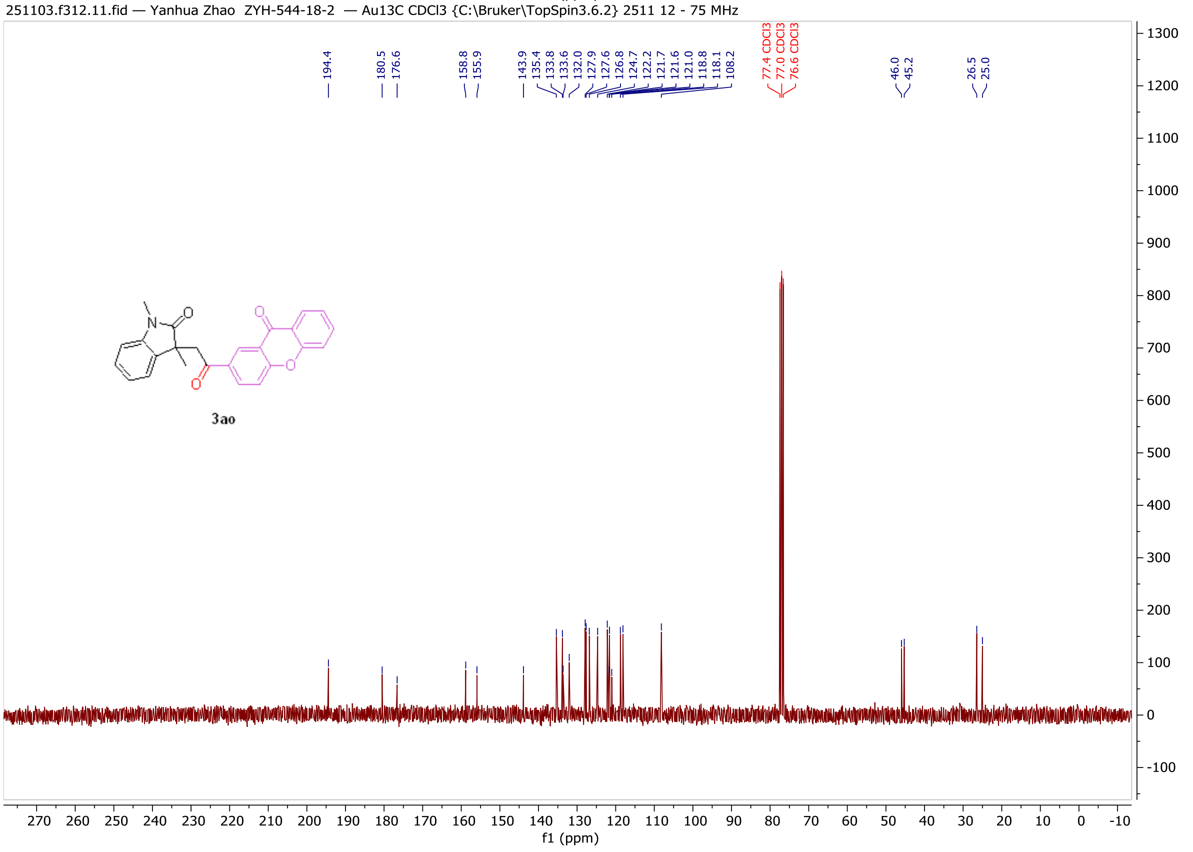
251103.f312.11.fid — Yanhua Zhao ZYH-544-18-2 — Au13C CDCl3 {C:\Bruker\TopSpin3.6.2} 2511 12 - 75 MHz
194.4
180.5
176.6
158.8
155.9
143.9
135.4
133.8
133.6
132.0
127.9
127.6
126.8
124.7
122.2
121.7
121.6
121.0
118.8
118.1
108.2
77.4 CDCl3
77.0 CDCl3
76.6 CDCl3
46.0
45.2
26.5
25.0
3ao
f1 (ppm)

251024.323.10.fid — Yanhua Zhao ZYH-544-19-2 — Au1H CDCl3 {C:\Bruker\TopSpin3.6.2} 2510 23 - 300 MHz

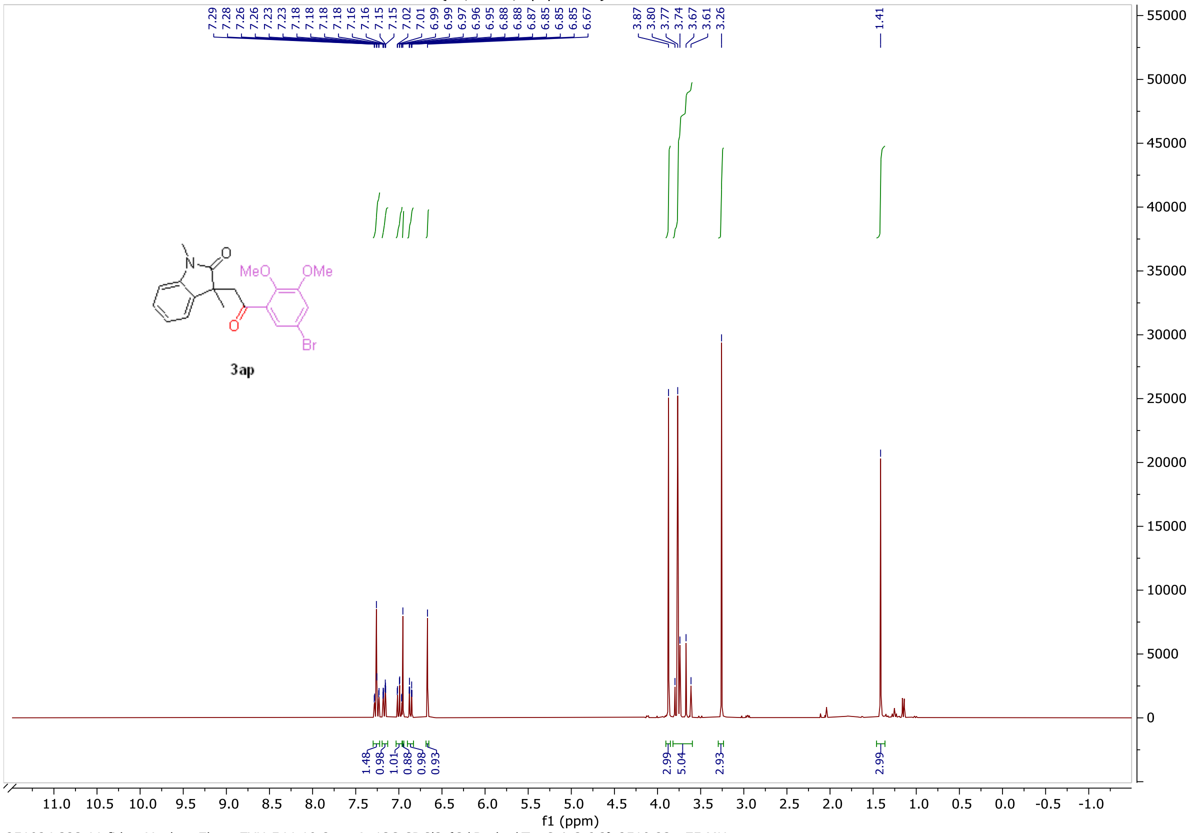

251024.323.11.fid — Yanhua Zhao ZYH-544-19-2 — Au13C CDCl3 {C:\Bruker\TopSpin3.6.2} 2510 23 - 75 MHz

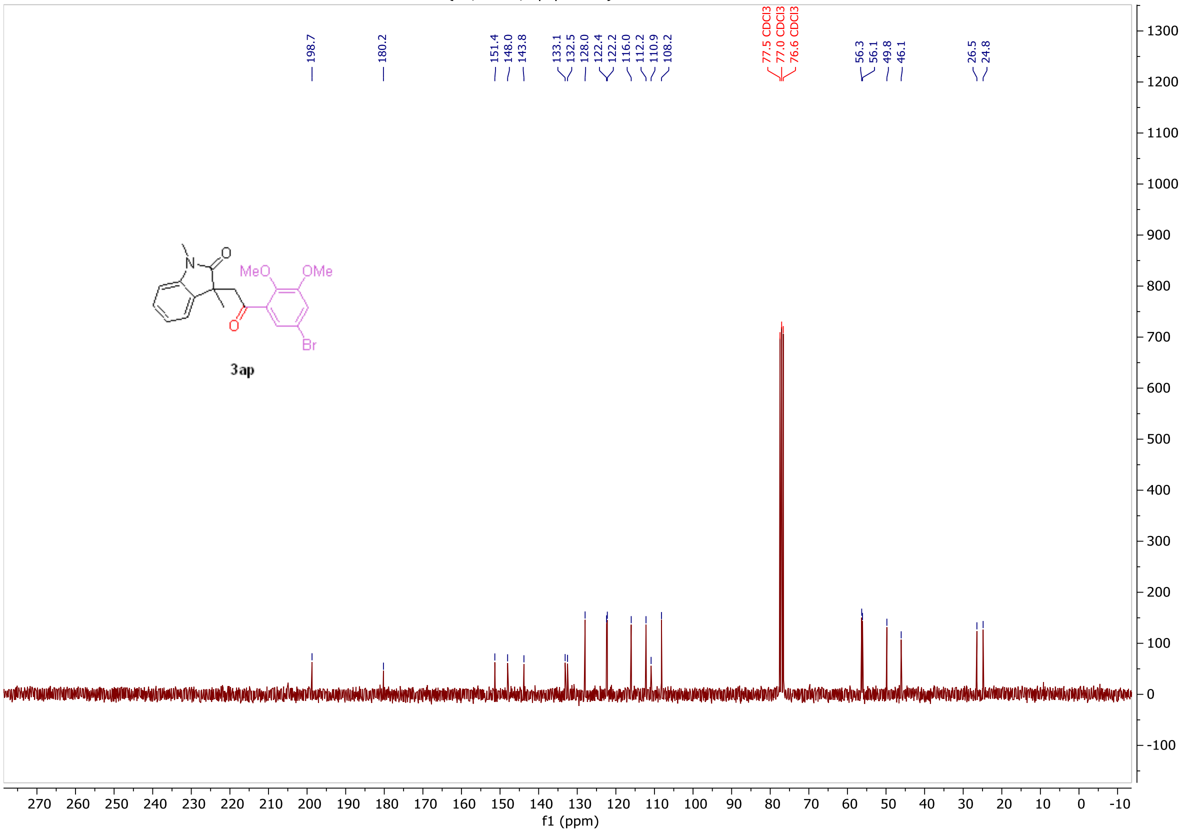

251024.324.10.fid — Yanhua Zhao ZYH-544-20 — Au1H CDCl3 {C:\Bruker\TopSpin3.6.2} 2510 24 - 300 MHz

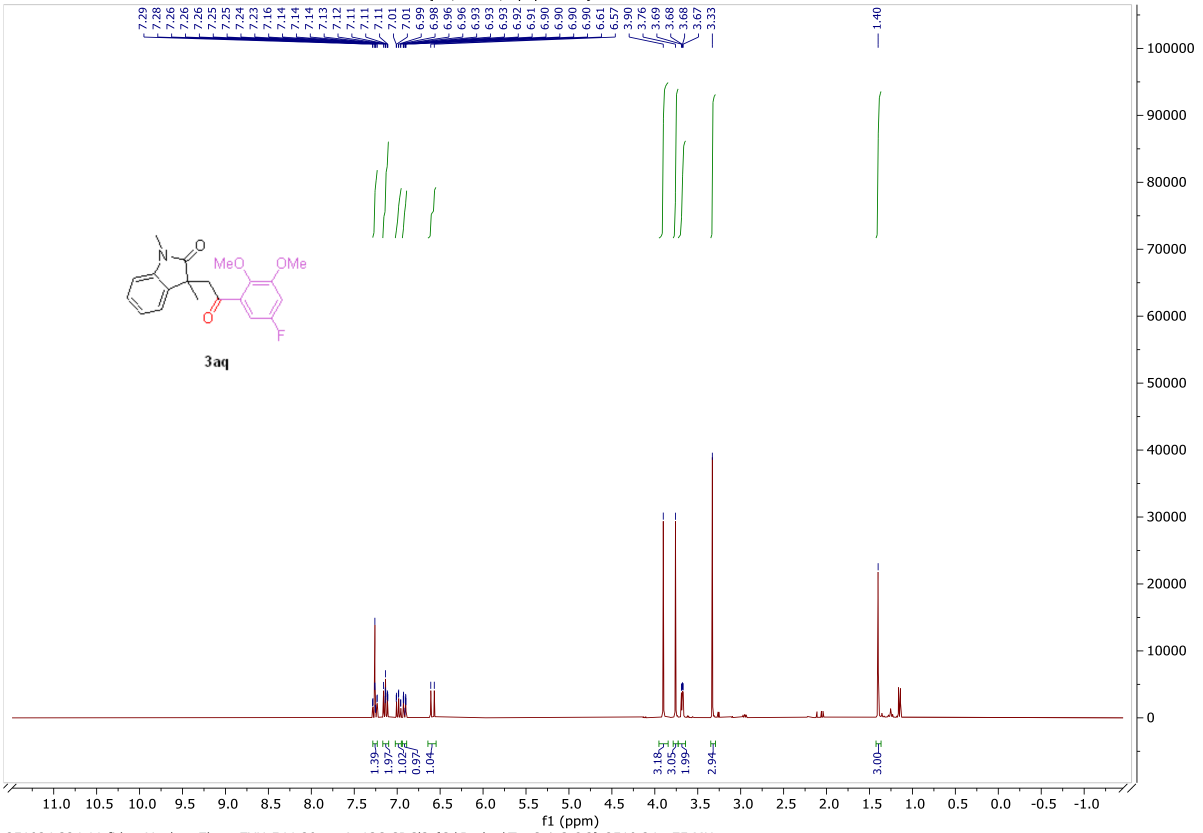

251024.324.11.fid — Yanhua Zhao ZYH-544-20 — Au13C CDCl3 {C:\Bruker\TopSpin3.6.2} 2510 24 - 75 MHz

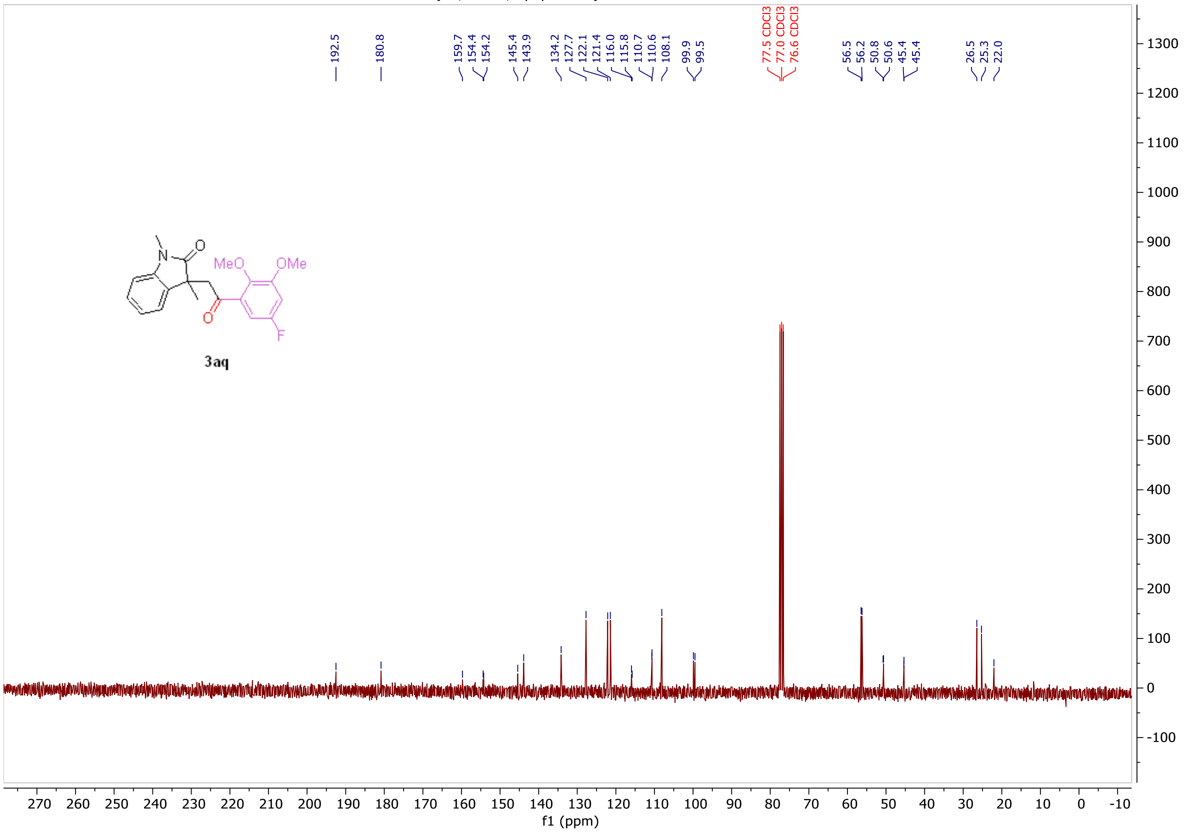

251024.324.12.fid — Yanhua Zhao ZYH-544-20 — Au19F CDCl3 {C:\Bruker\TopSpin3.6.2} 2510 24-282 MHz

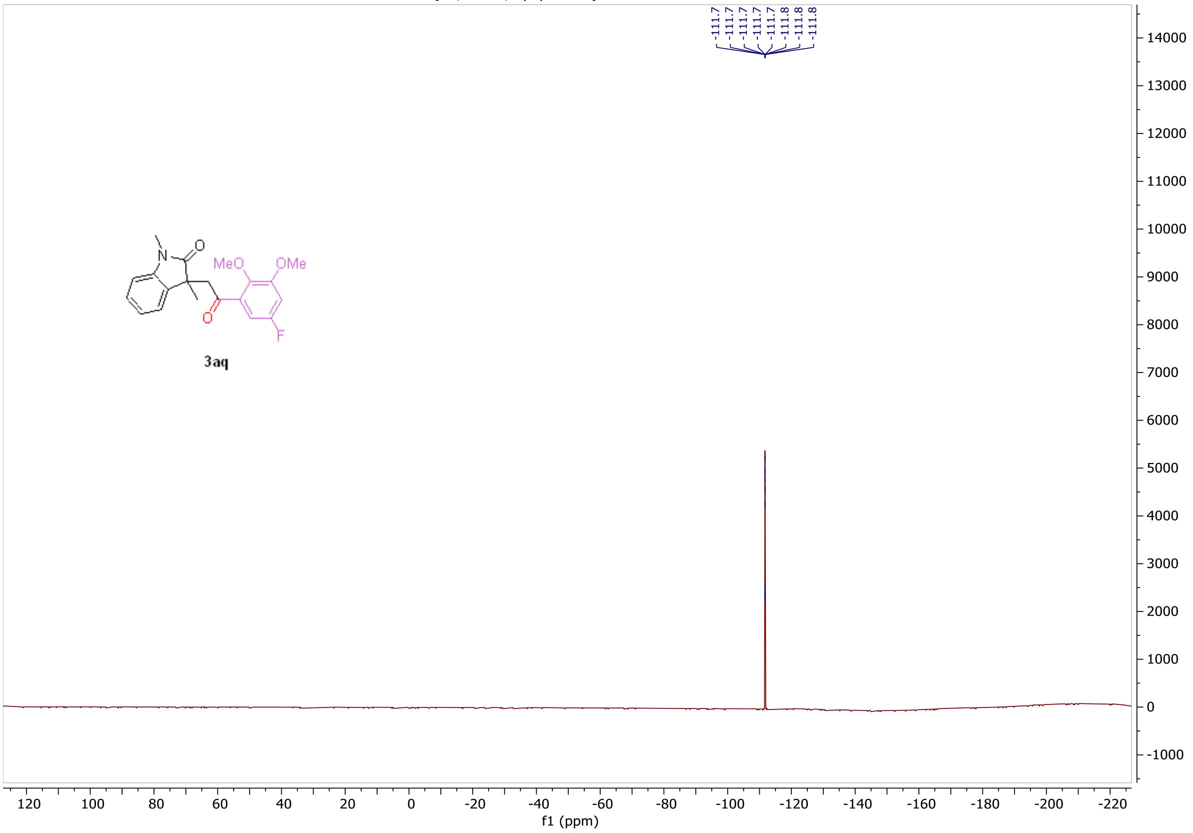

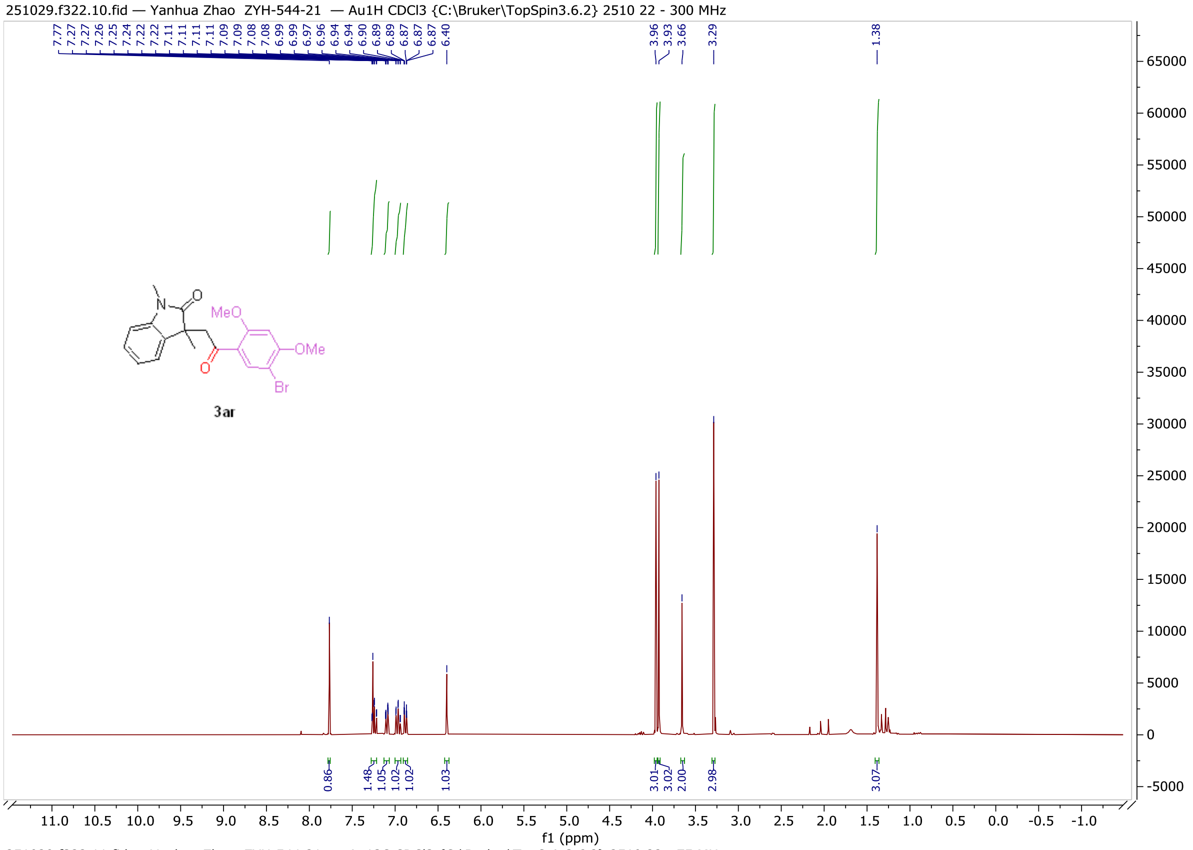
251029.f322.10.fid — Yanhua Zhao ZYH-544-21 — Au1H CDCl3 {C:\Bruker\TopSpin3.6.2} 2510 22 - 300 MHz
7.77 7.27 7.27 7.26 7.25 7.24 7.22 7.22 7.11 7.11 7.11 7.09 7.09 7.08 6.99 6.99 6.97 6.96 6.94 6.94 6.90 6.89 6.89 6.87 6.87 6.87 6.40
3.96 3.93 3.66 3.29
1.38
0.86 1.48 1.05 1.02 1.02 1.03
3.01 3.02 2.00 2.98
3.07
MeO
OMe
Br
3ar
f1 (ppm)

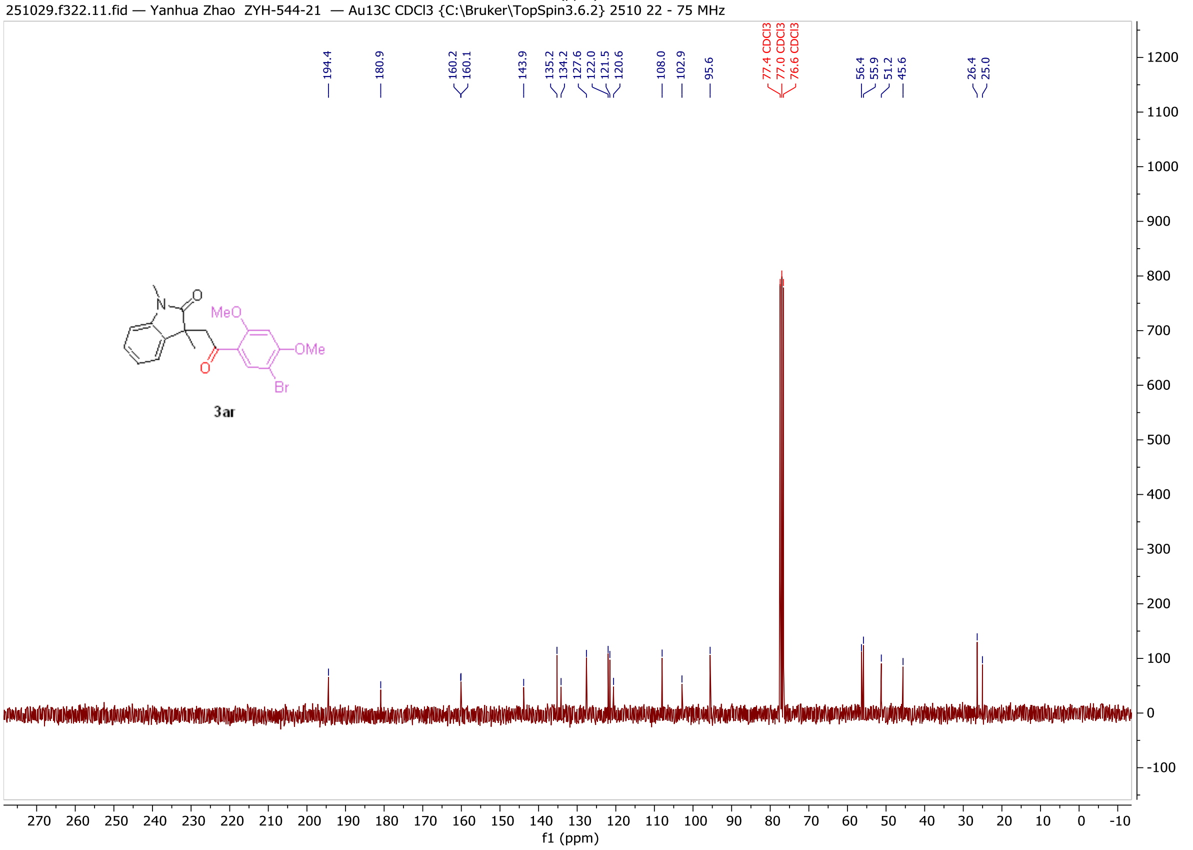
251029.f322.11.fid — Yanhua Zhao ZYH-544-21 — Au13C CDCl3 {C:\Bruker\TopSpin3.6.2} 2510 22 - 75 MHz
194.4
180.9
160.2 160.1
143.9
135.2 134.2 127.6 122.0 121.5 120.6
108.0
102.9
95.6
77.4 CDCl3 77.0 CDCl3 76.6 CDCl3
56.4 55.9 51.2 45.6
26.4 25.0
MeO
OMe
Br
3ar
f1 (ppm)

251030.f315.10.fid — Yanhua Zhao ZYH-544-22 — Au1H CDCl3 {C:\Bruker\TopSpin3.6.2} 2510 15 - 300 MHz

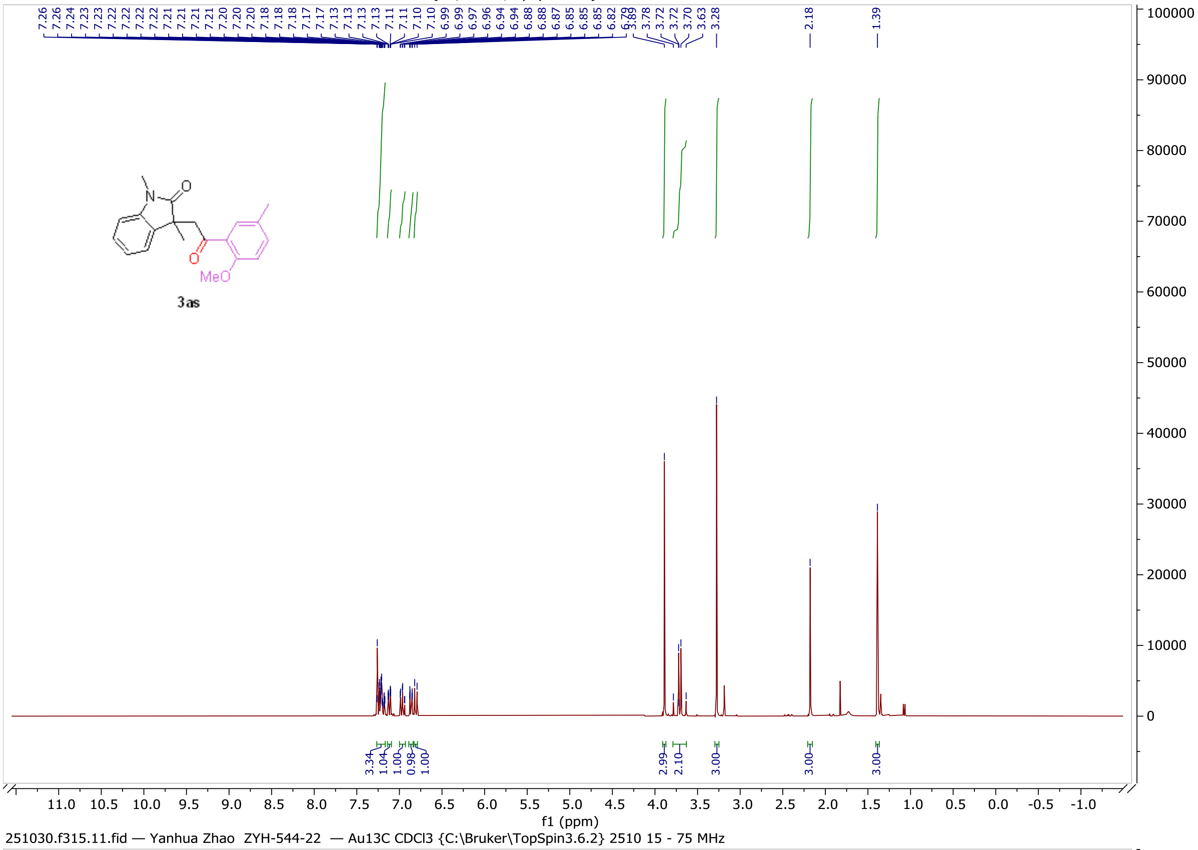

251030.f315.11.fid — Yanhua Zhao ZYH-544-22 — Au13C CDCl3 {C:\Bruker\TopSpin3.6.2} 2510 15 - 75 MHz

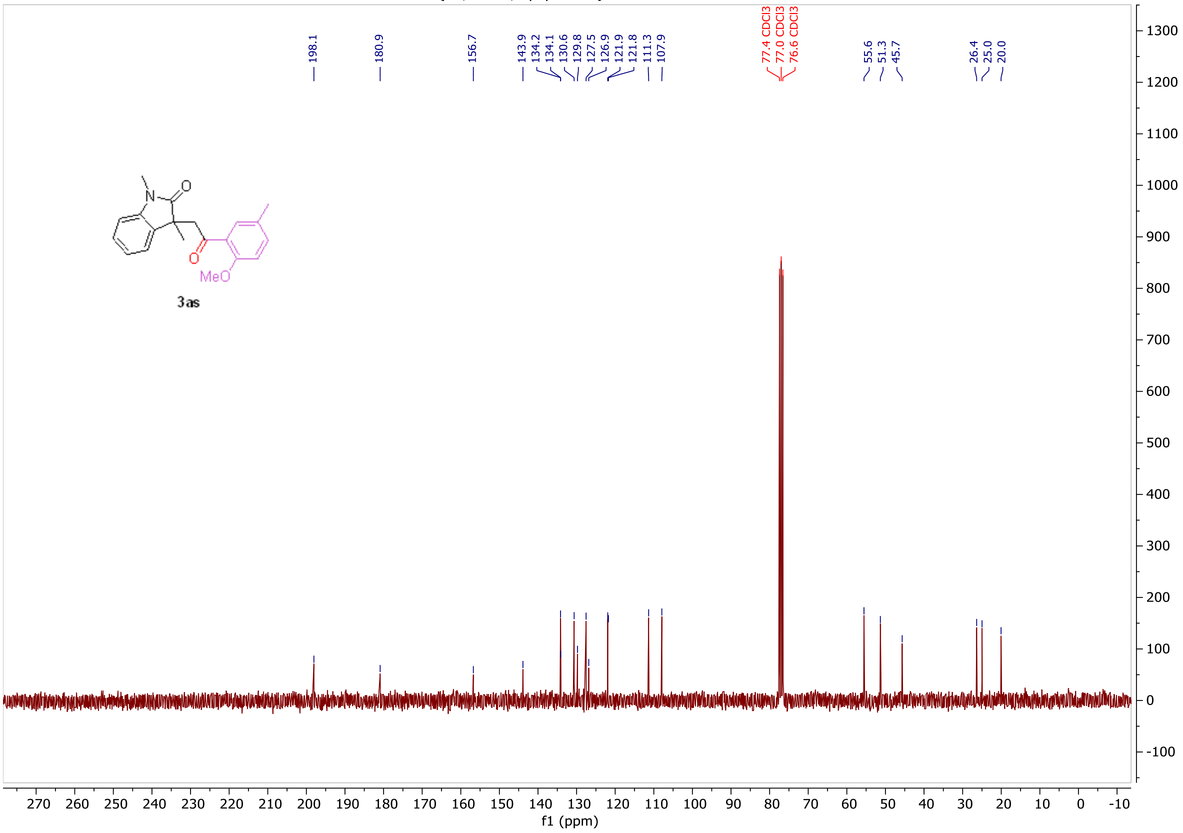

251030.f316.10.fid — Yanhua Zhao ZYH-544-23 — Au1H CDCl3 {C:\Bruker\TopSpin3.6.2} 2510 16 - 300 MHz

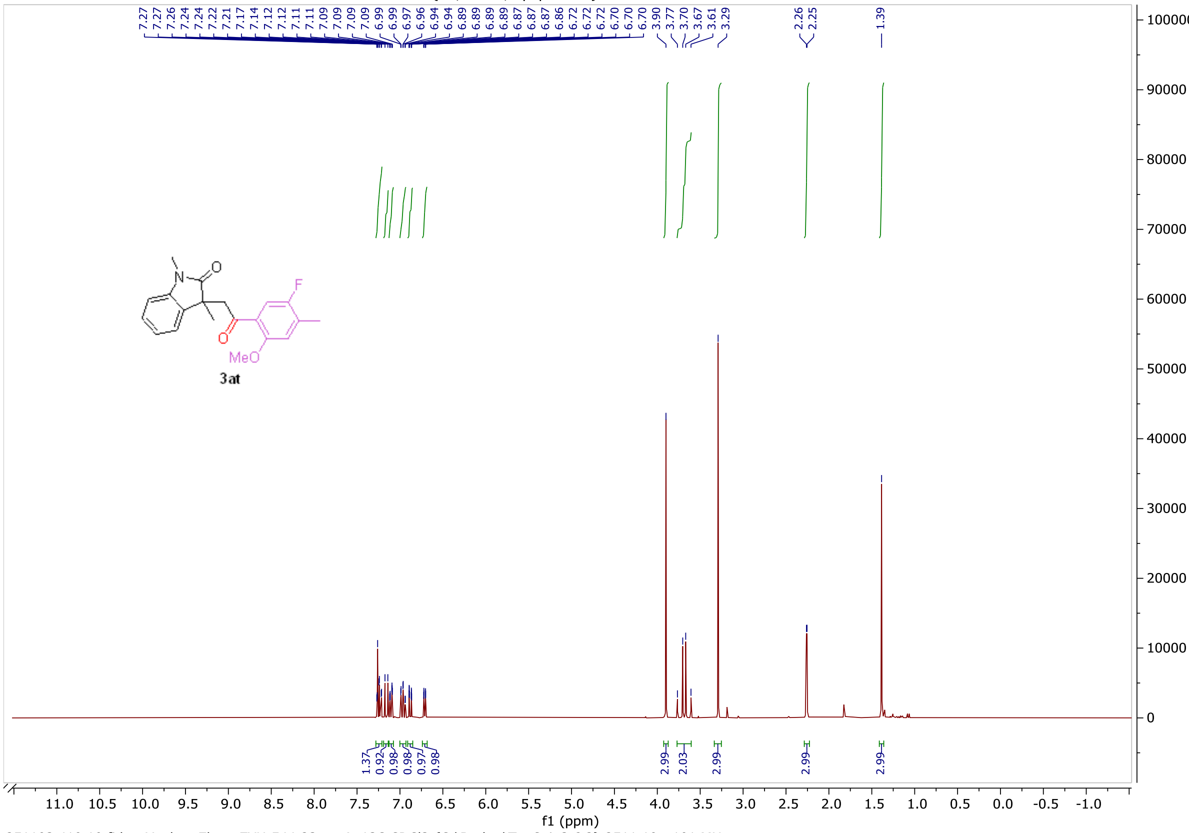

251103.419.10.fid — Yanhua Zhao ZYH-544-23 — Au13C CDCl3 {C:\Bruker\TopSpin3.6.2} 2511 19 - 101 MHz

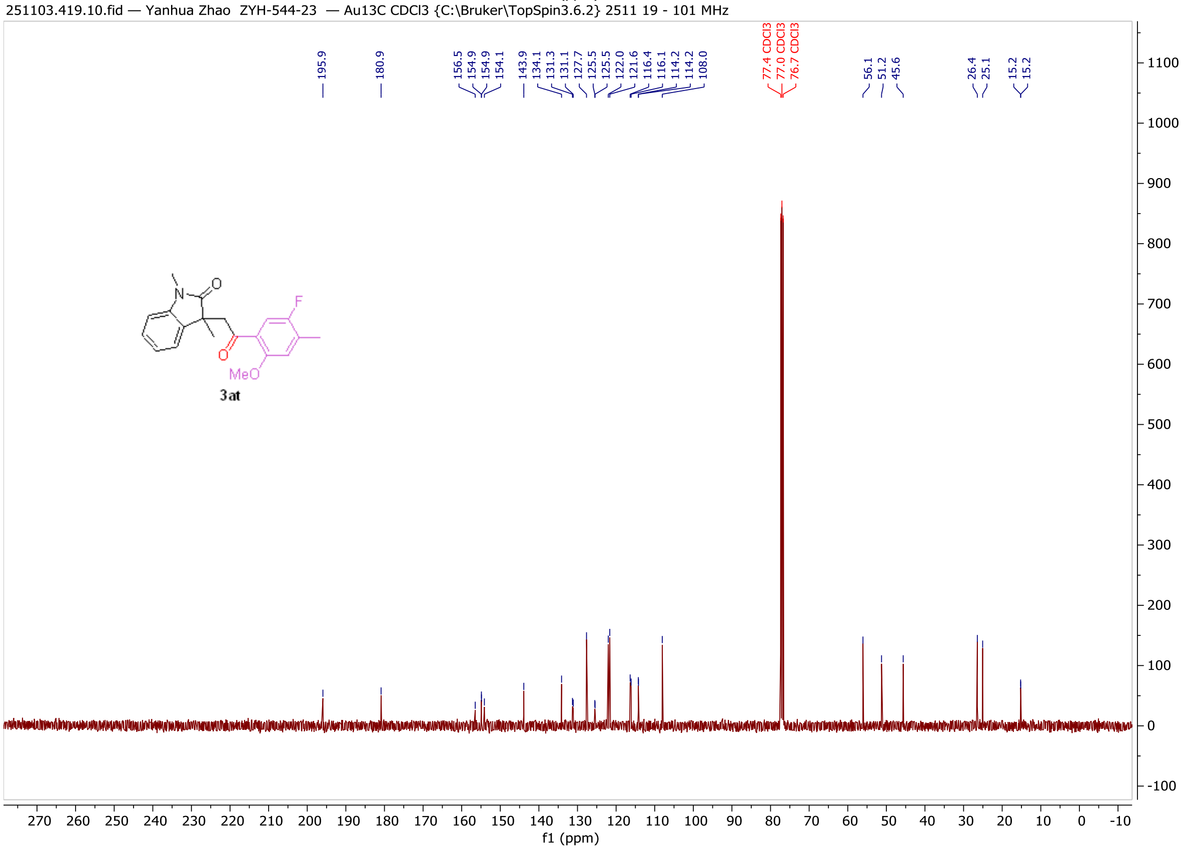

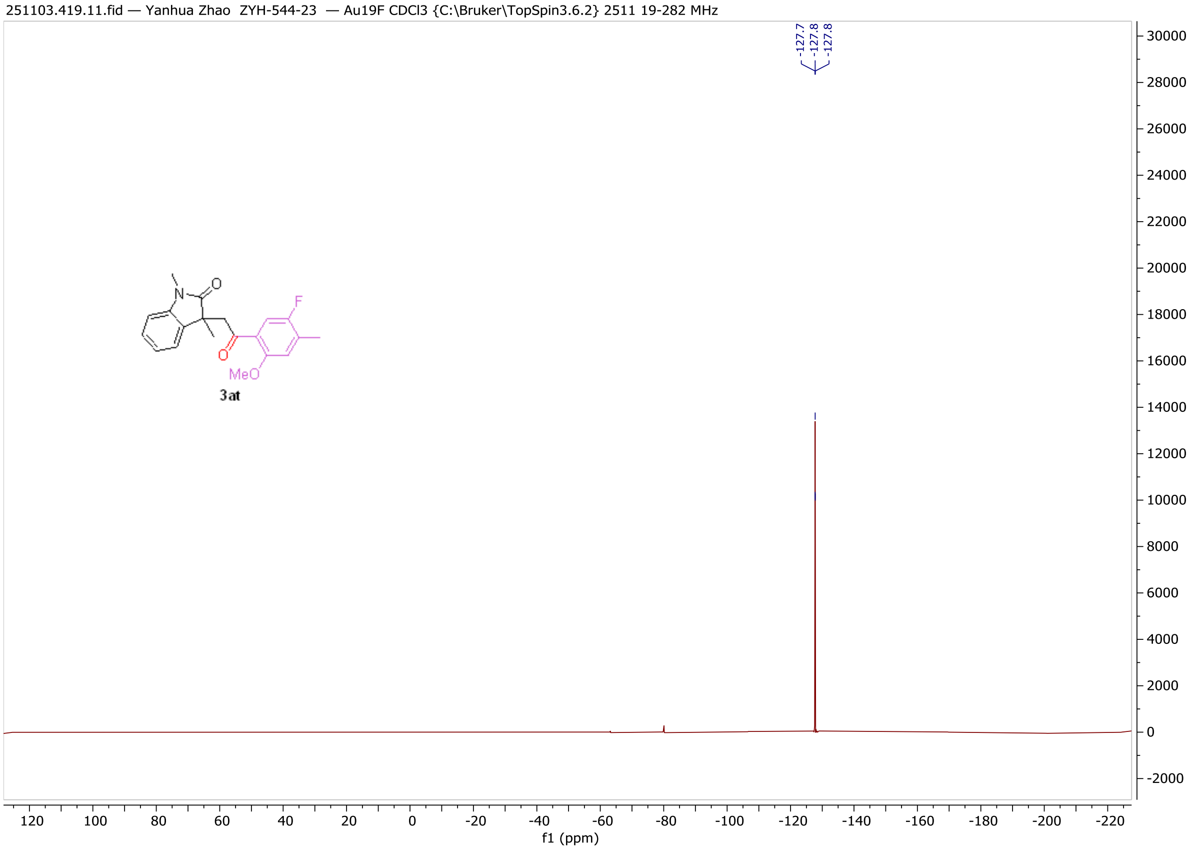
251103.419.11.fid — Yanhua Zhao ZYH-544-23 — Au19F CDCl3 {C:\Bruker\TopSpin3.6.2} 2511 19-282 MHz
-127.7
-127.8
-127.8
3at
f1 (ppm)

251030.f317.10.fid — Yanhua Zhao ZYH-544-24 — Au1H CDCl3 {C:\Bruker\TopSpin3.6.2} 2510 17 - 300 MHz

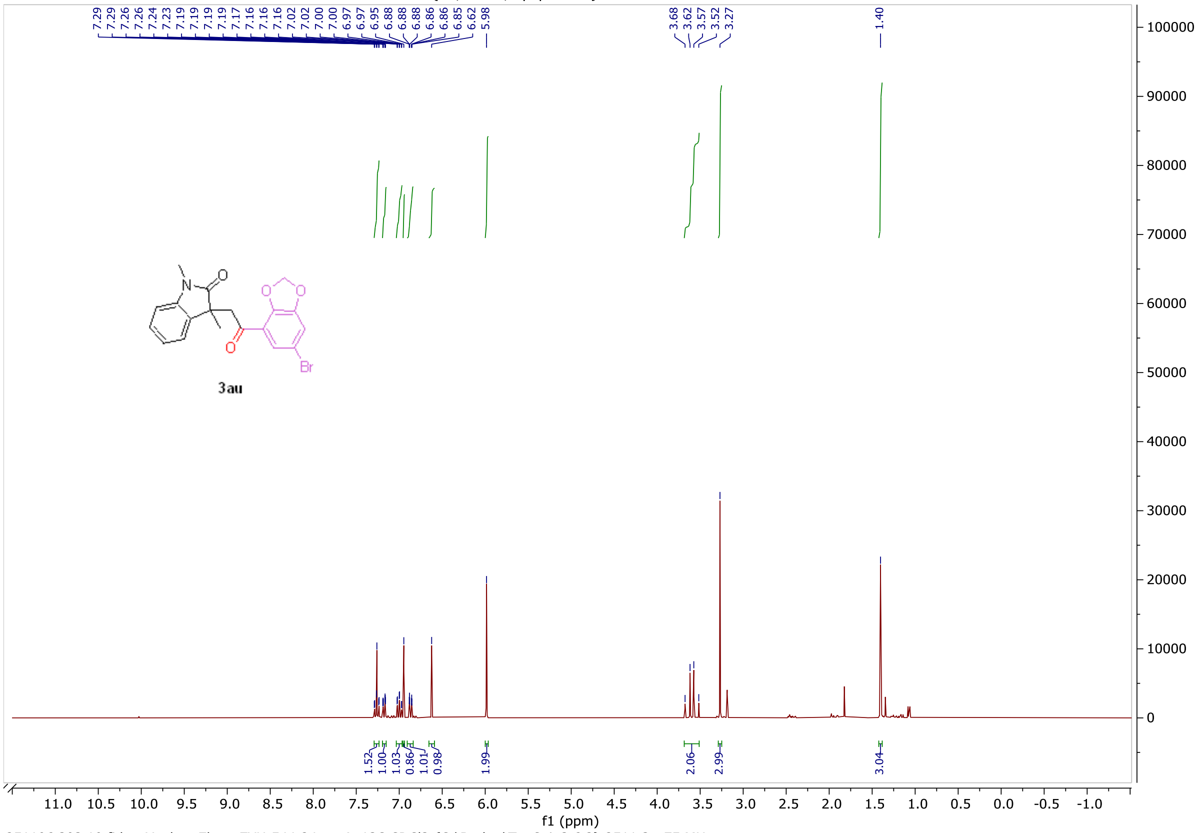

251106.303.10.fid — Yanhua Zhao ZYH-544-24 — Au13C CDCl3 {C:\Bruker\TopSpin3.6.2} 2511 3 - 75 MHz

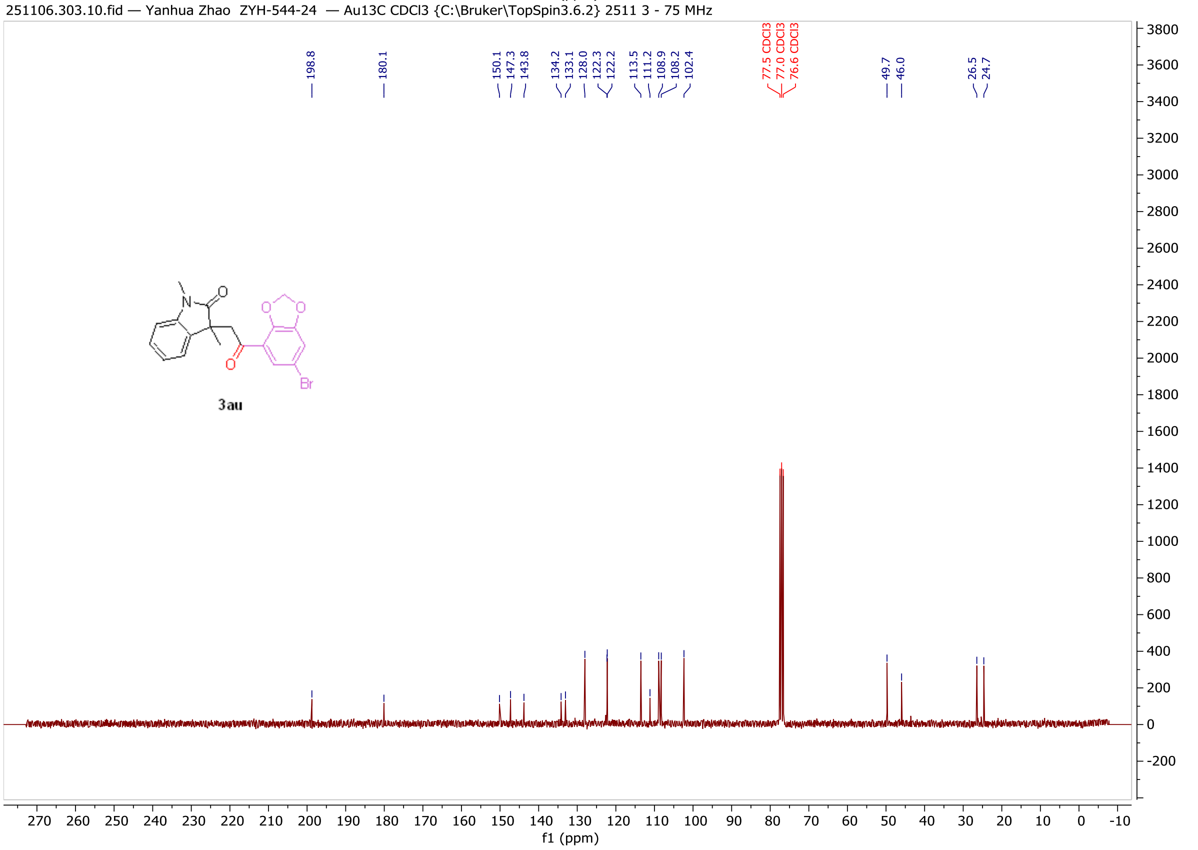

251125.347.10.fid — Yanhua Zhao, ZYH-544-7 — Au1H CDCl3 {C:\Bruker\TopSpin3.6.2} 2511 47 - 300 MHz

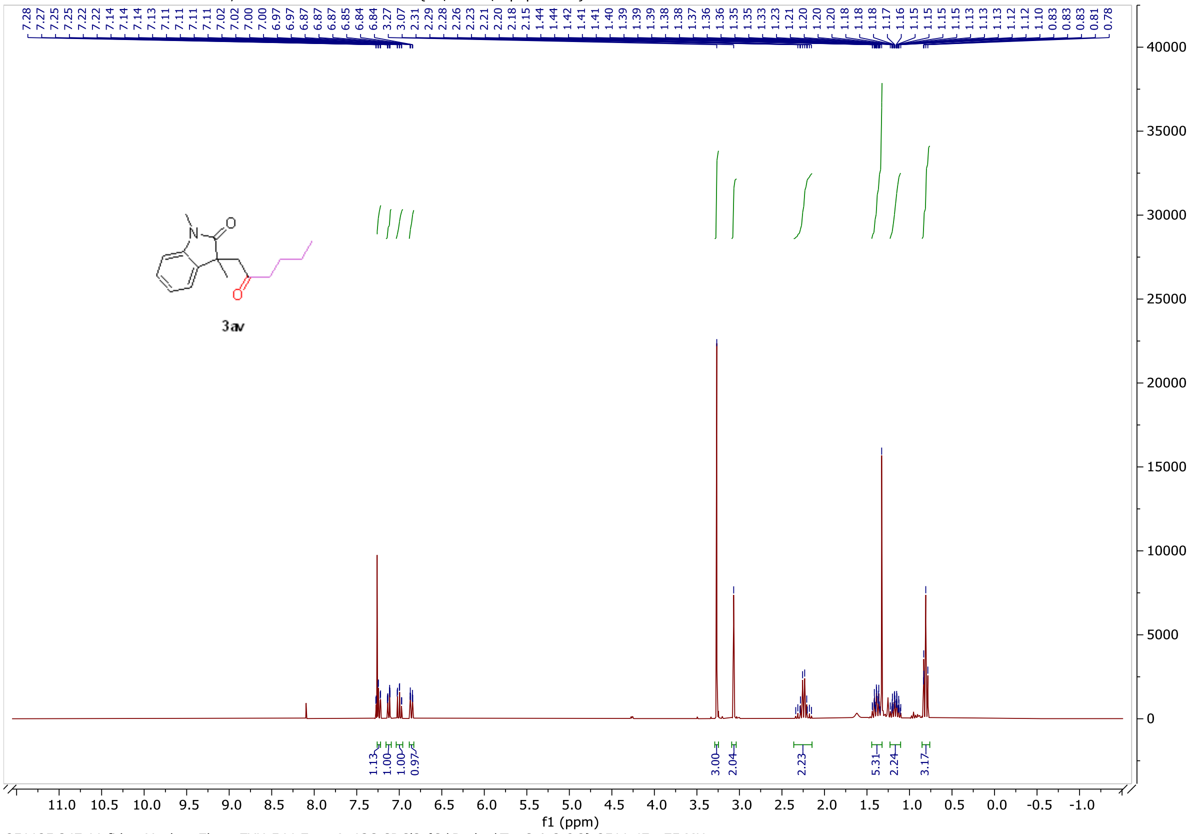

251125.347.11.fid — Yanhua Zhao, ZYH-544-7 — Au13C CDCl3 {C:\Bruker\TopSpin3.6.2} 2511 47 - 75 MHz

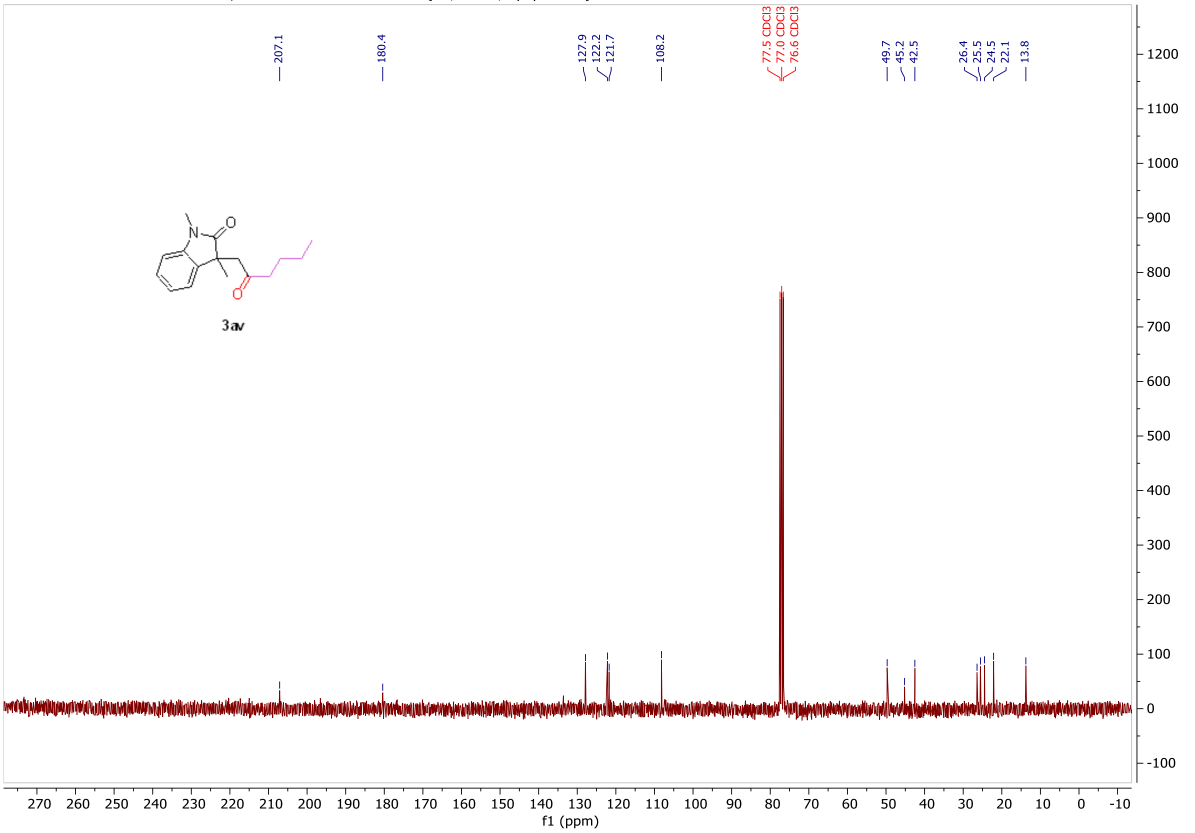

251125.346.10.fid — Yanhua Zhao, ZYH-544-6 — Au1H CDCl3 {C:\Bruker\TopSpin3.6.2} 2511 46 - 300 MHz

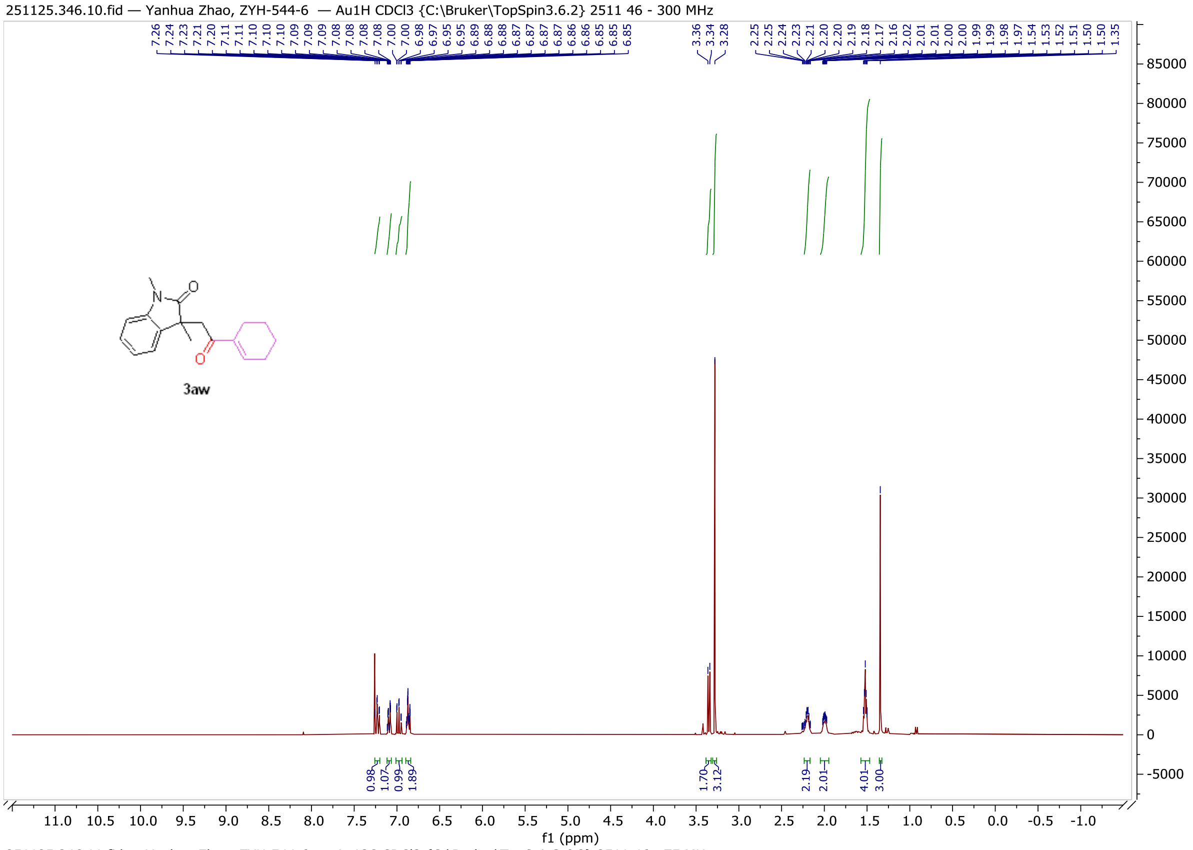

251125.346.11.fid — Yanhua Zhao, ZYH-544-6 — Au13C CDCl3 {C:\Bruker\TopSpin3.6.2} 2511 46 - 75 MHz

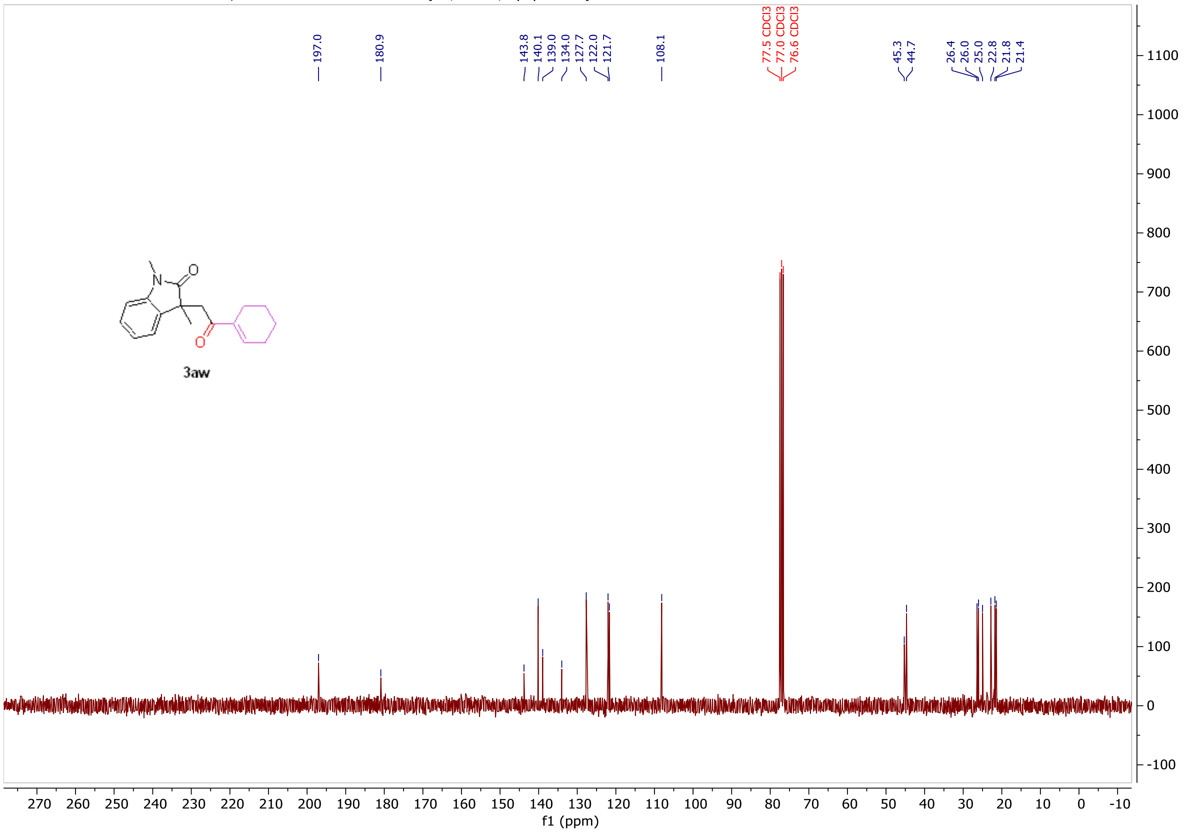

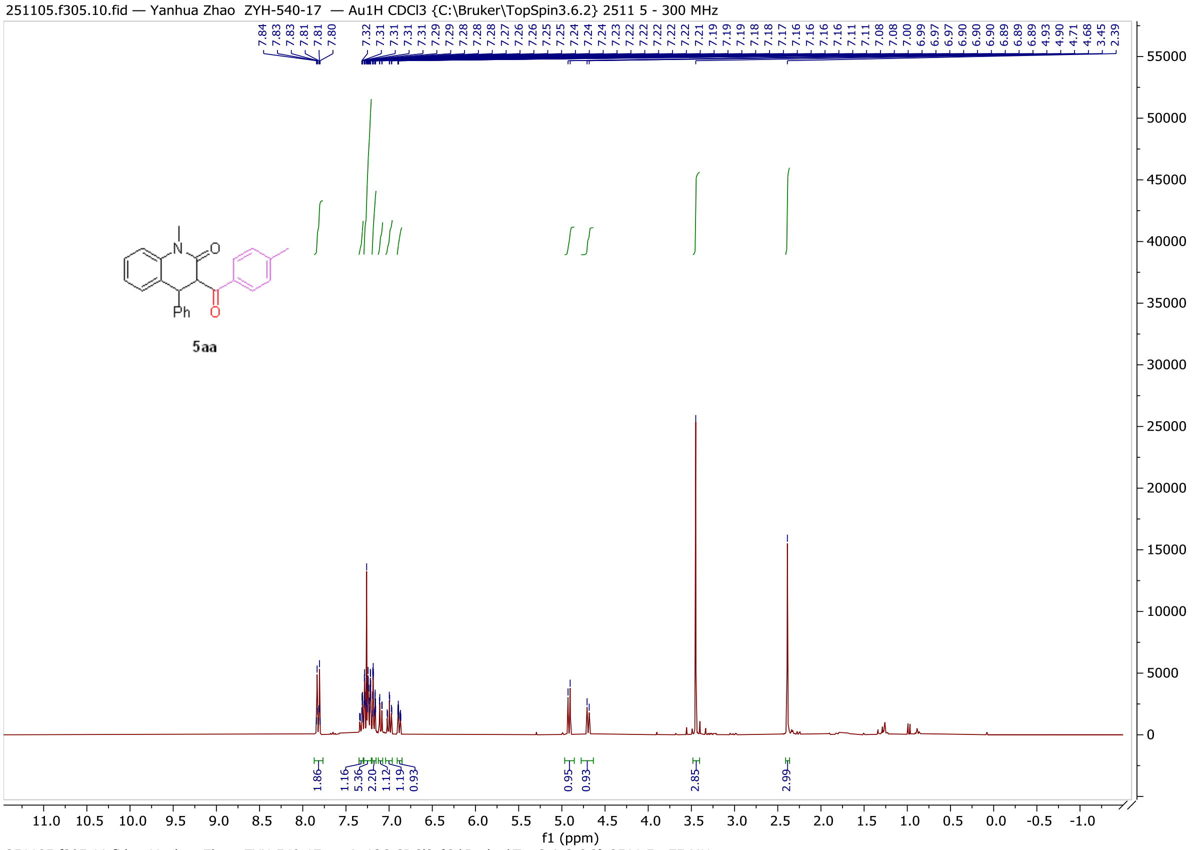

251105.f305.10.fid — Yanhua Zhao ZYH-540-17 — Au1H CDCl3 {C:\Bruker\TopSpin3.6.2} 2511 5 - 300 MHz
7.84 7.83 7.83 7.81 7.81 7.80 7.32 7.31 7.31 7.31 7.31 7.29 7.29 7.28 7.28 7.28 7.27 7.26 7.26 7.25 7.25 7.24 7.24 7.24 7.23 7.22 7.22 7.22 7.22 7.22 7.21 7.19 7.19 7.19 7.18 7.18 7.17 7.16 7.16 7.16 7.16 7.11 7.08 7.08 7.00 6.99 6.97 6.97 6.90 6.90 6.90 6.89 6.89 6.89 4.93 4.90 4.71 4.68 3.45 2.39
O
N
O
Ph
5aa
1.86 1.16 5.36 2.20 1.12 1.19 0.93 0.95 0.93 2.85 2.99
f1 (ppm)

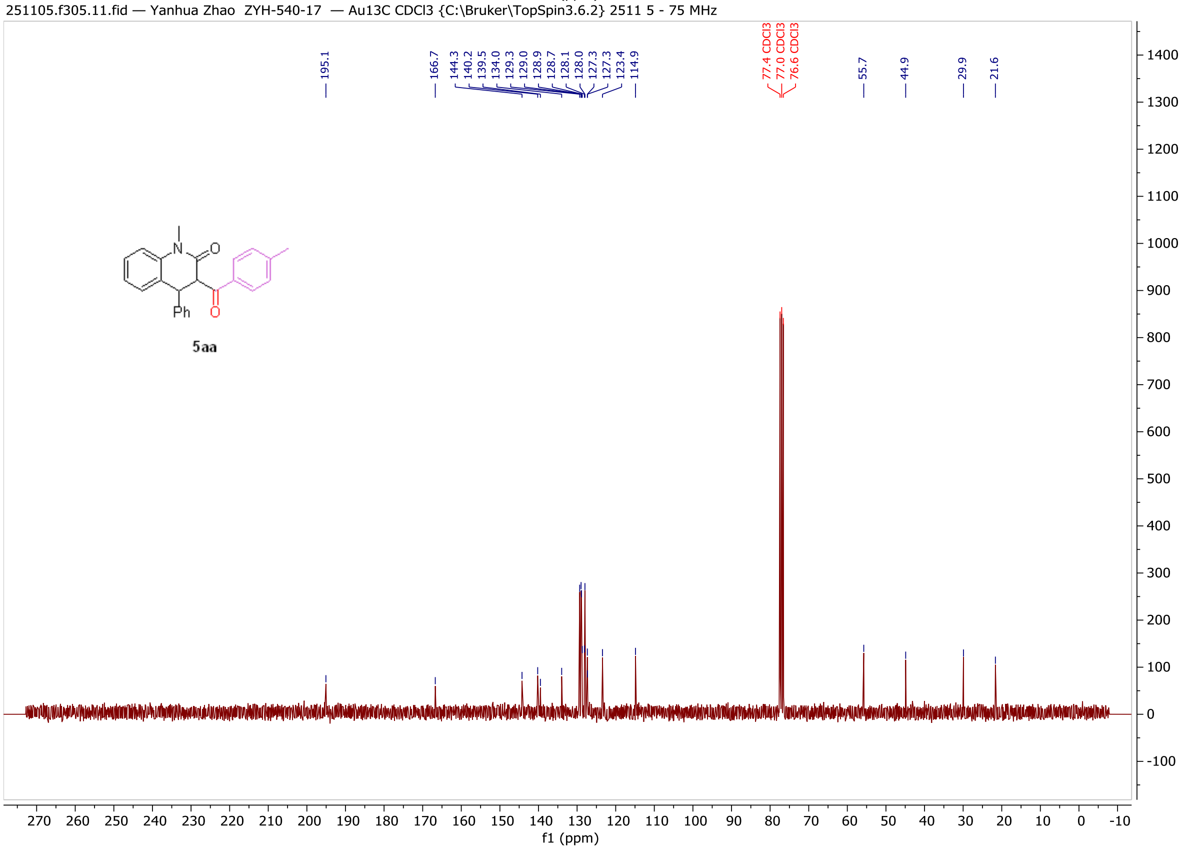

251105.f305.11.fid — Yanhua Zhao ZYH-540-17 — Au13C CDCl3 {C:\Bruker\TopSpin3.6.2} 2511 5 - 75 MHz
195.1 166.7 144.3 140.2 139.5 134.0 129.3 129.0 128.9 128.7 128.1 128.0 127.3 127.3 123.4 114.9
77.4 CDCl3 77.0 CDCl3 76.6 CDCl3
55.7 44.9 29.9 21.6
O
N
O
Ph
5aa
f1 (ppm)

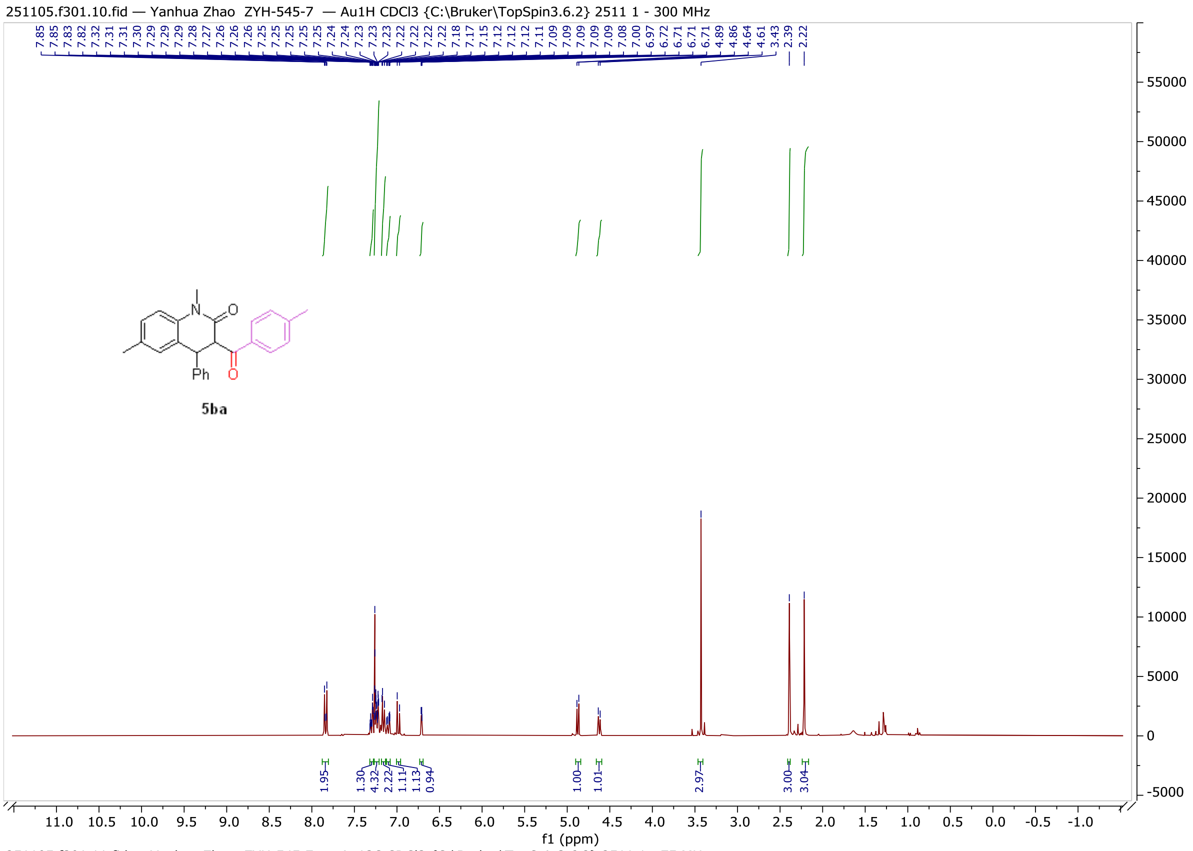
251105.f301.10.fid — Yanhua Zhao ZYH-545-7 — Au1H CDCl3 {C:\Bruker\TopSpin3.6.2} 2511 1 - 300 MHz
5ba
Ph
f1 (ppm)

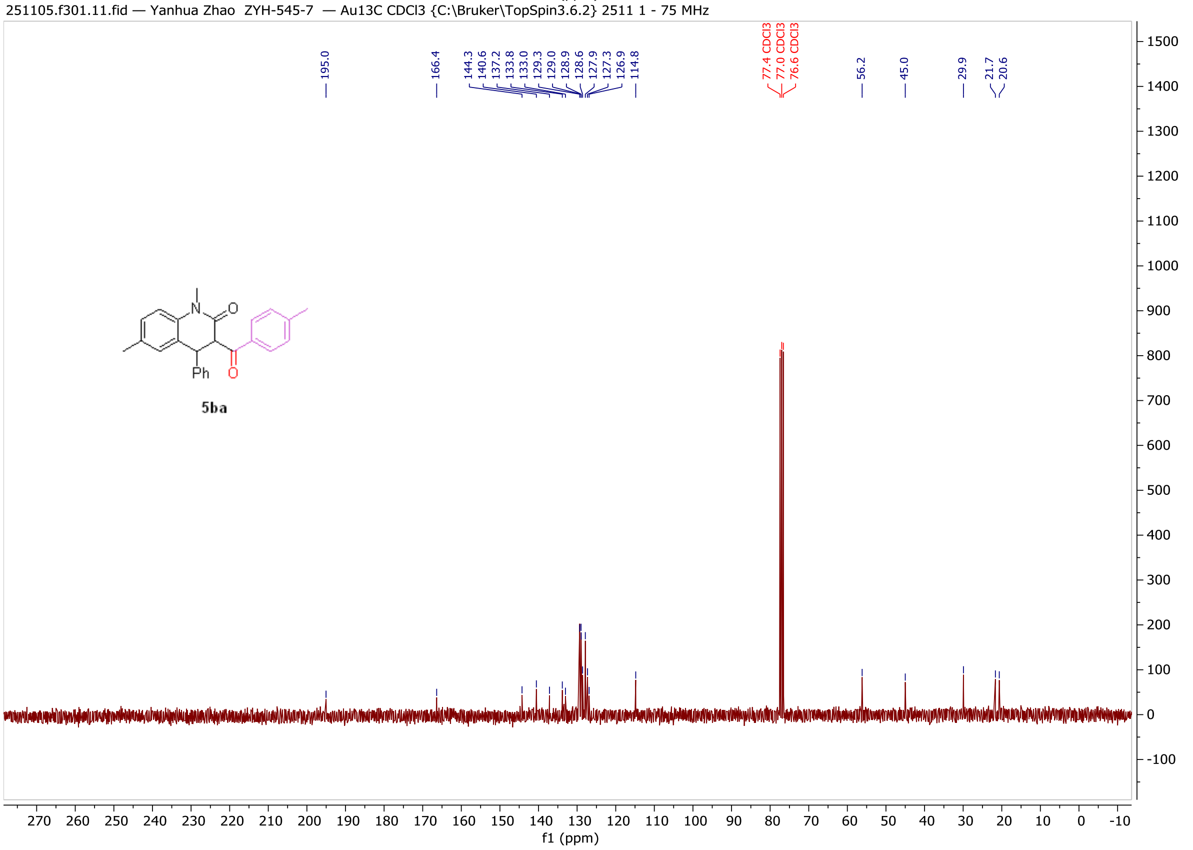
251105.f301.11.fid — Yanhua Zhao ZYH-545-7 — Au13C CDCl3 {C:\Bruker\TopSpin3.6.2} 2511 1 - 75 MHz
195.0
166.4
144.3
140.6
137.2
133.8
133.0
129.3
129.0
128.9
128.6
127.9
127.3
126.9
114.8
77.4 CDCl3
77.0 CDCl3
76.6 CDCl3
56.2
45.0
29.9
21.7
20.6
5ba
Ph
f1 (ppm)

251103.405.10.fid — Yanhua Zhao ZYH-545-1 — Au1H CDCl3 {C:\Bruker\TopSpin3.6.2} 2511 5 - 300 MHz

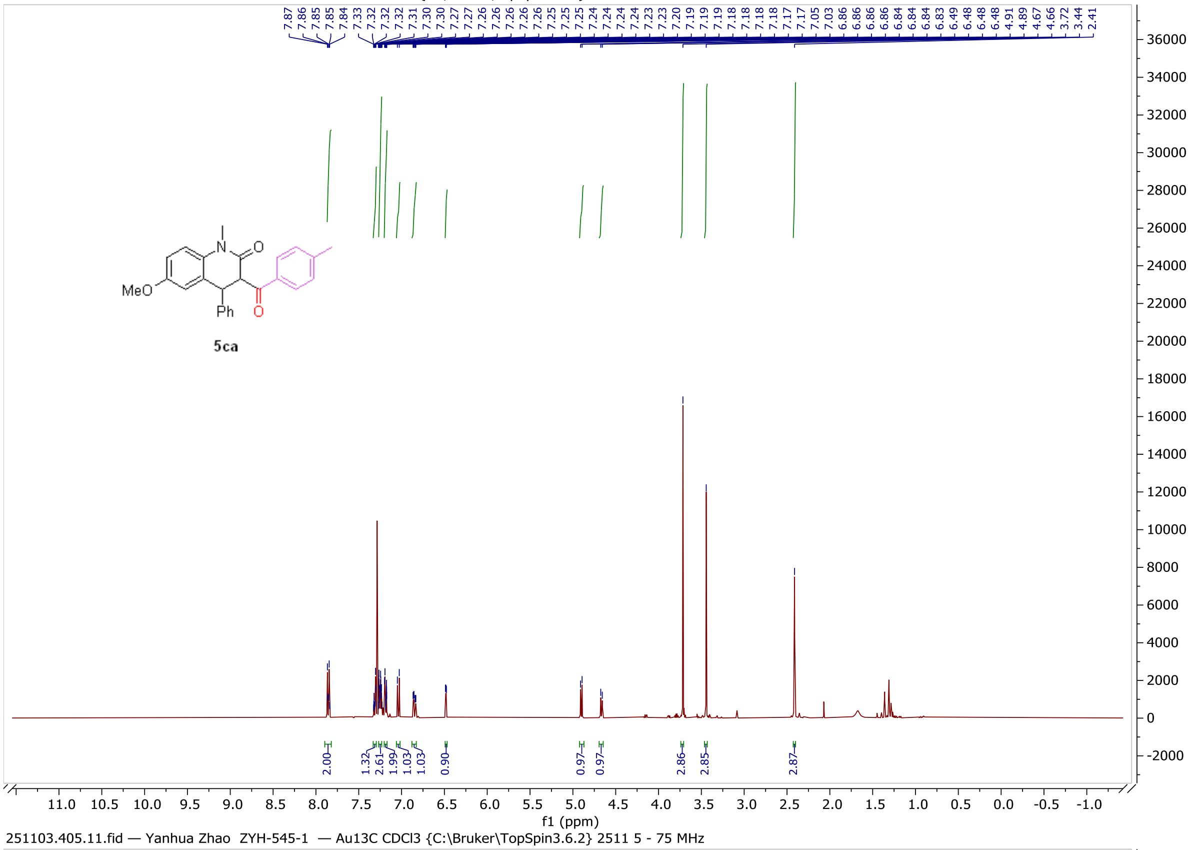

251103.405.11.fid — Yanhua Zhao ZYH-545-1 — Au13C CDCl3 {C:\Bruker\TopSpin3.6.2} 2511 5 - 75 MHz

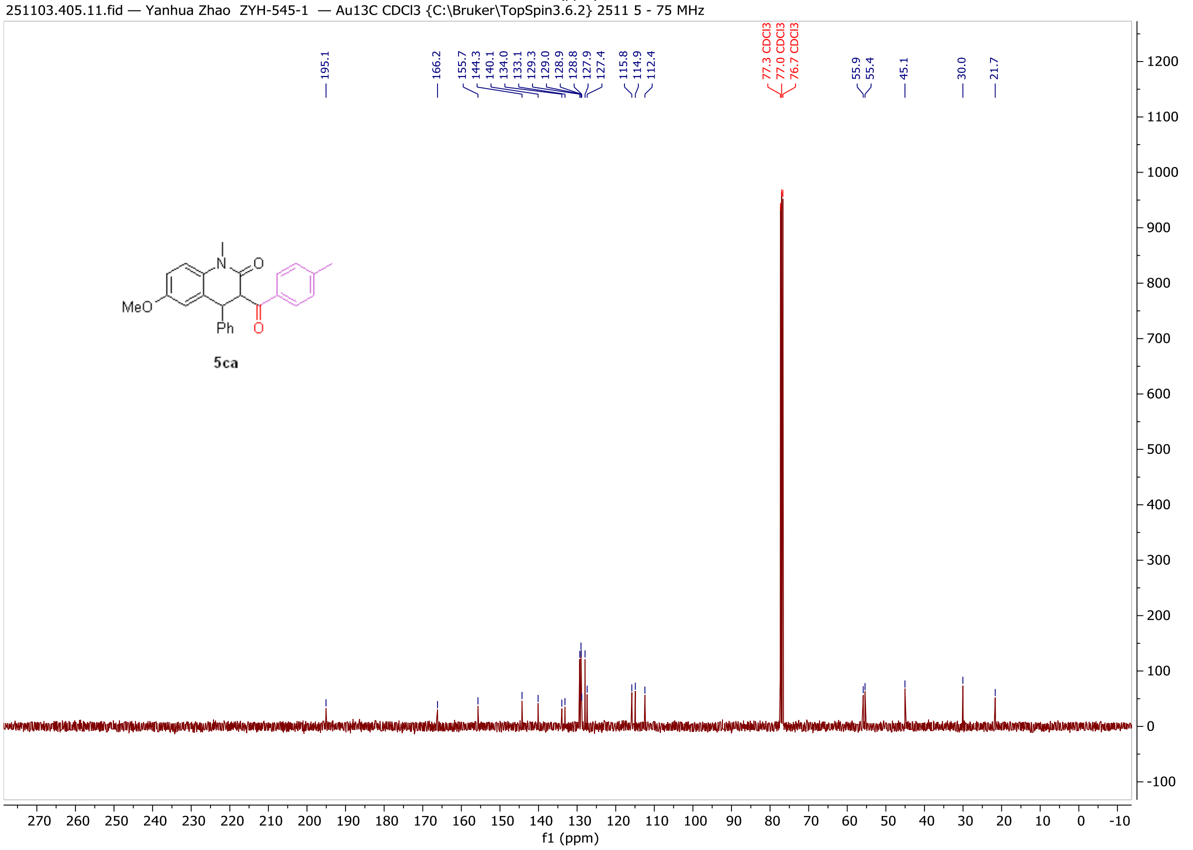

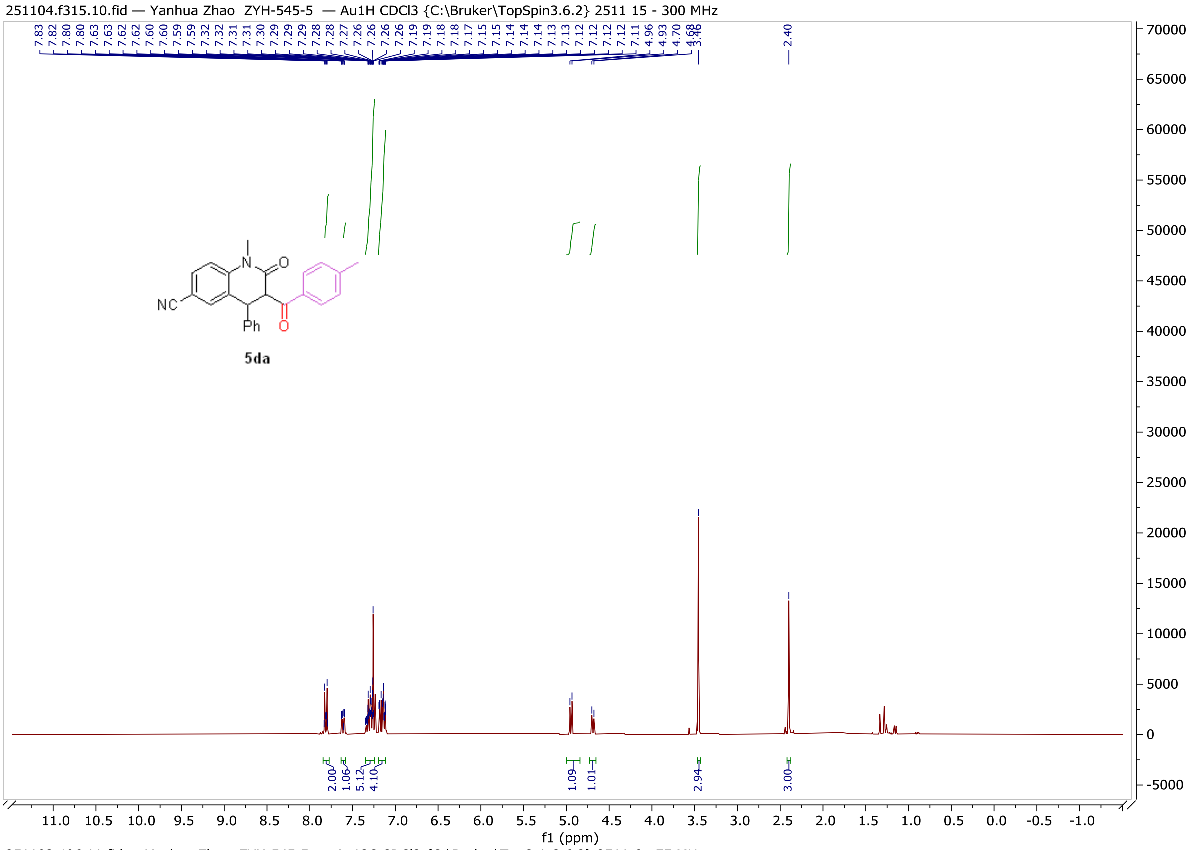
251104.f315.10.fid — Yanhua Zhao ZYH-545-5 — Au1H CDCl3 {C:\Bruker\TopSpin3.6.2} 2511 15 - 300 MHz
5da
f1 (ppm)

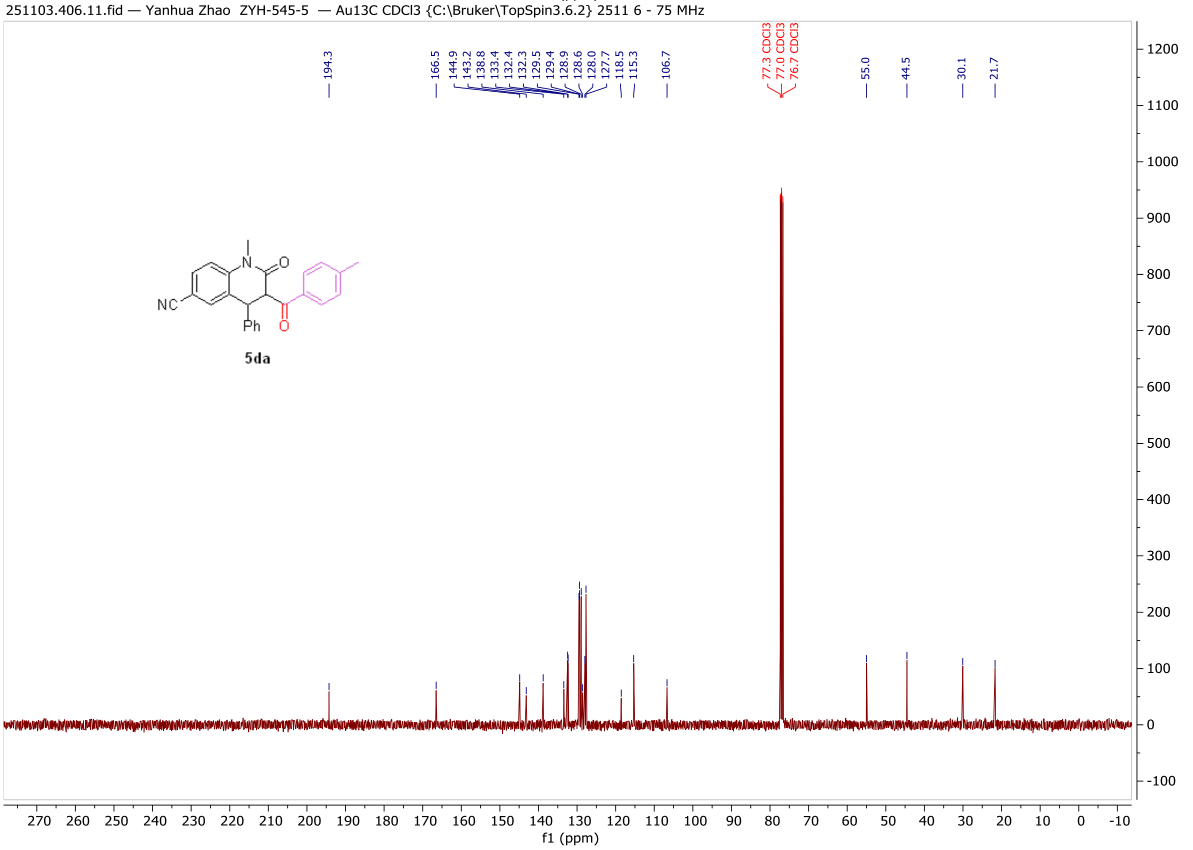
251103.406.11.fid — Yanhua Zhao ZYH-545-5 — Au13C CDCl3 {C:\Bruker\TopSpin3.6.2} 2511 6 - 75 MHz
194.3
166.5
144.9
143.2
138.8
133.4
132.4
132.3
129.5
129.4
128.9
128.6
128.0
127.7
118.5
115.3
106.7
77.3 CDCl3
77.0 CDCl3
76.7 CDCl3
55.0
44.5
30.1
21.7
5da
f1 (ppm)

251113.320.10.fid — Yanhua Zhao ZYH-545-8 — Au1H CDCl3 {C:\Bruker\TopSpin3.6.2} 2511 20 - 300 MHz

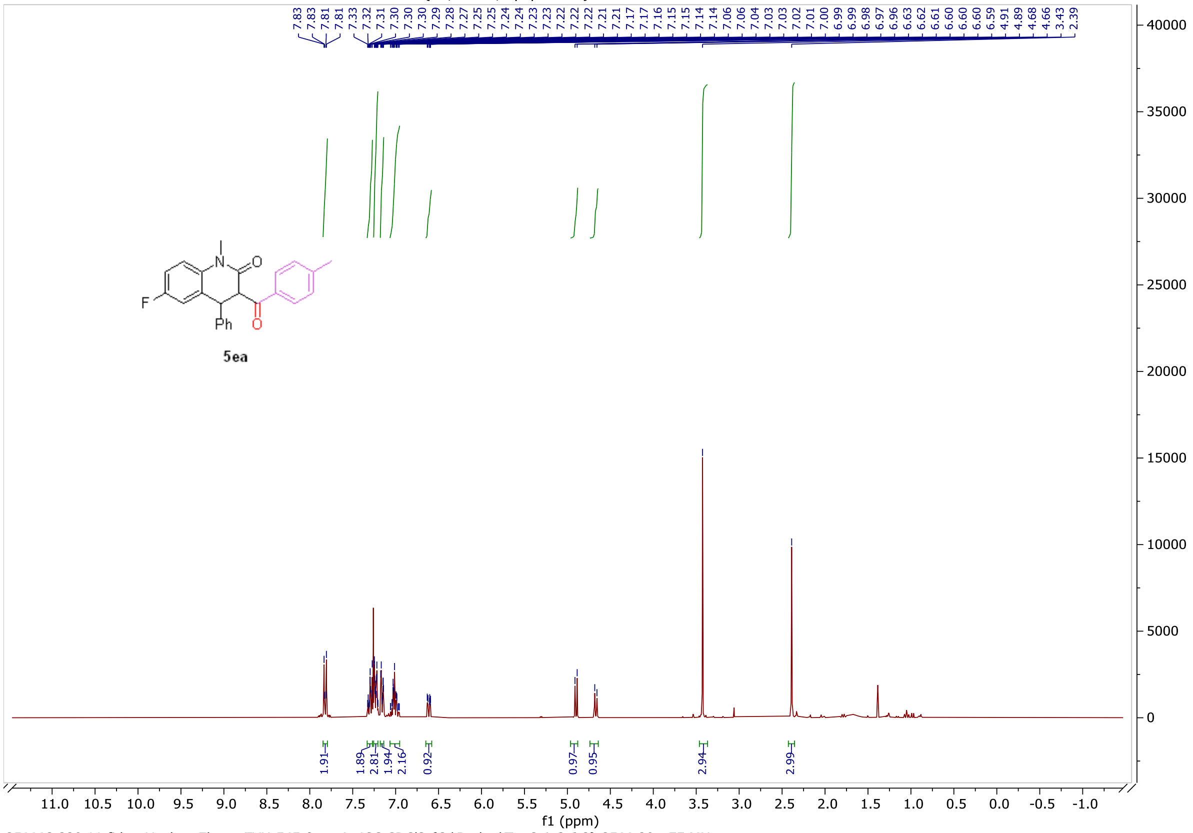

251113.320.11.fid — Yanhua Zhao ZYH-545-8 — Au13C CDCl3 {C:\Bruker\TopSpin3.6.2} 2511 20 - 75 MHz

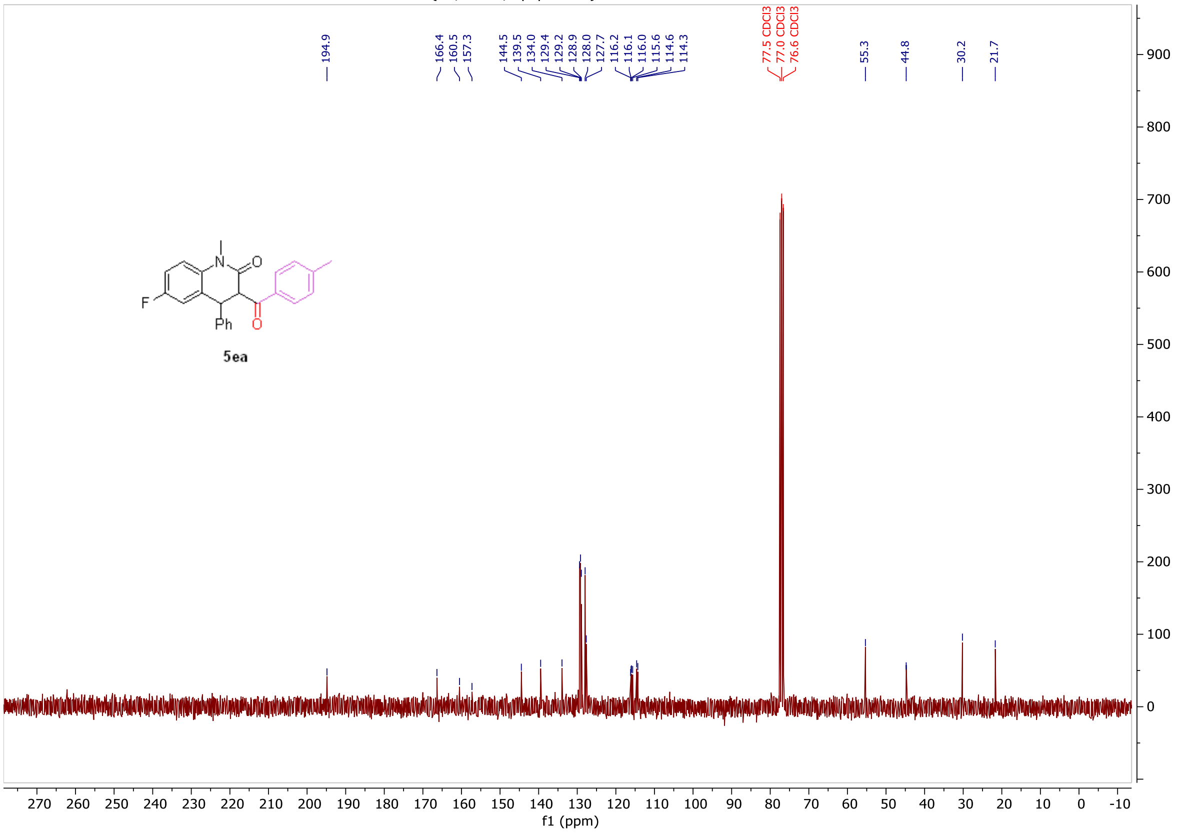

251113.320.12.fid — Yanhua Zhao ZYH-545-8 — Au19F CDCl3 {C:\Bruker\TopSpin3.6.2} 2511 20-282 MHz

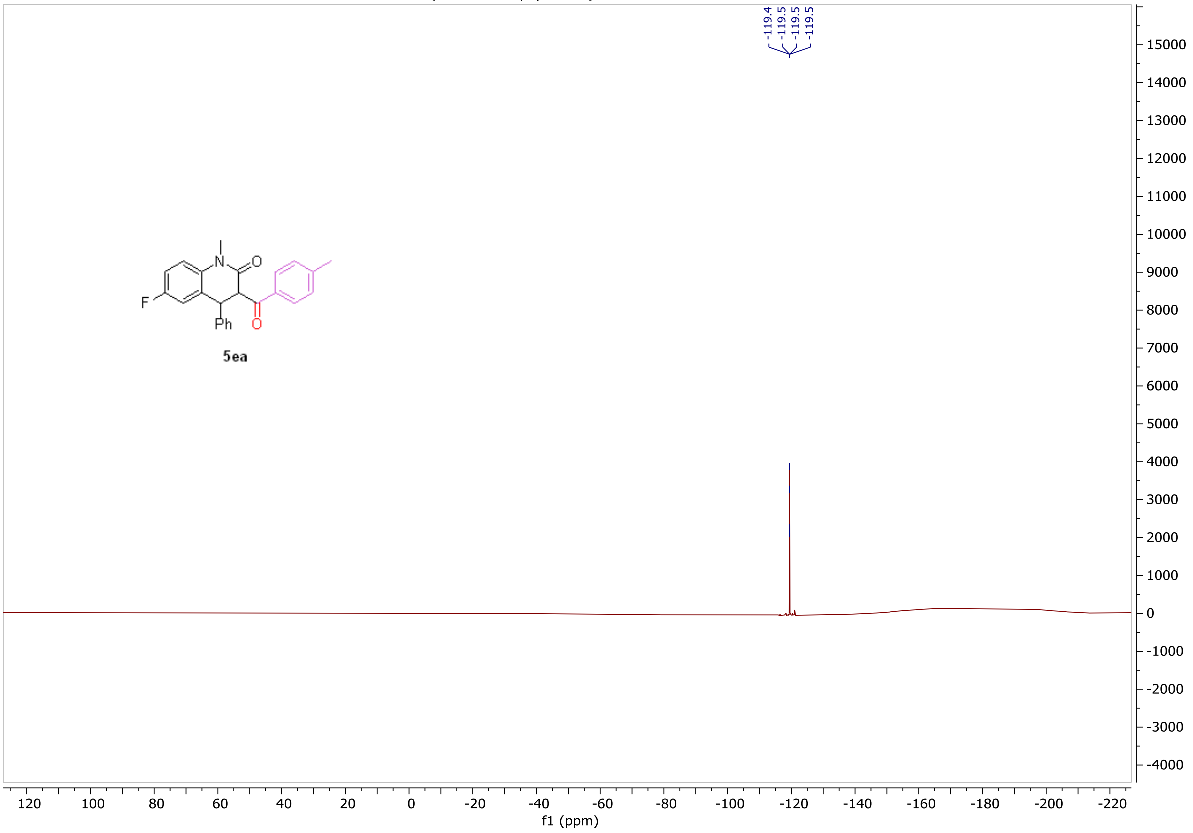

251107.310.10.fid — Yanhua Zhao ZYH-550-1 — Au1H CDCl3 {C:\Bruker\TopSpin3.6.2} 2511 10 - 300 MHz

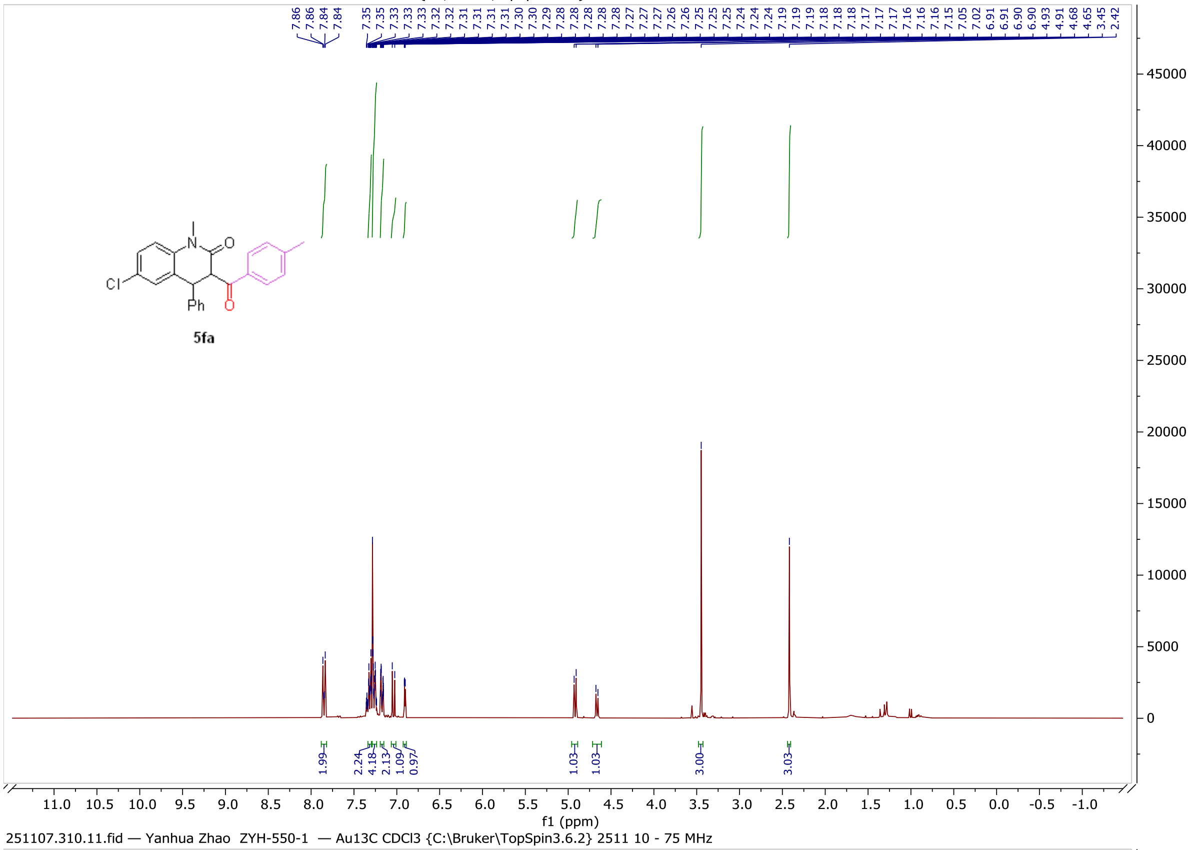

251107.310.11.fid — Yanhua Zhao ZYH-550-1 — Au13C CDCl3 {C:\Bruker\TopSpin3.6.2} 2511 10 - 75 MHz

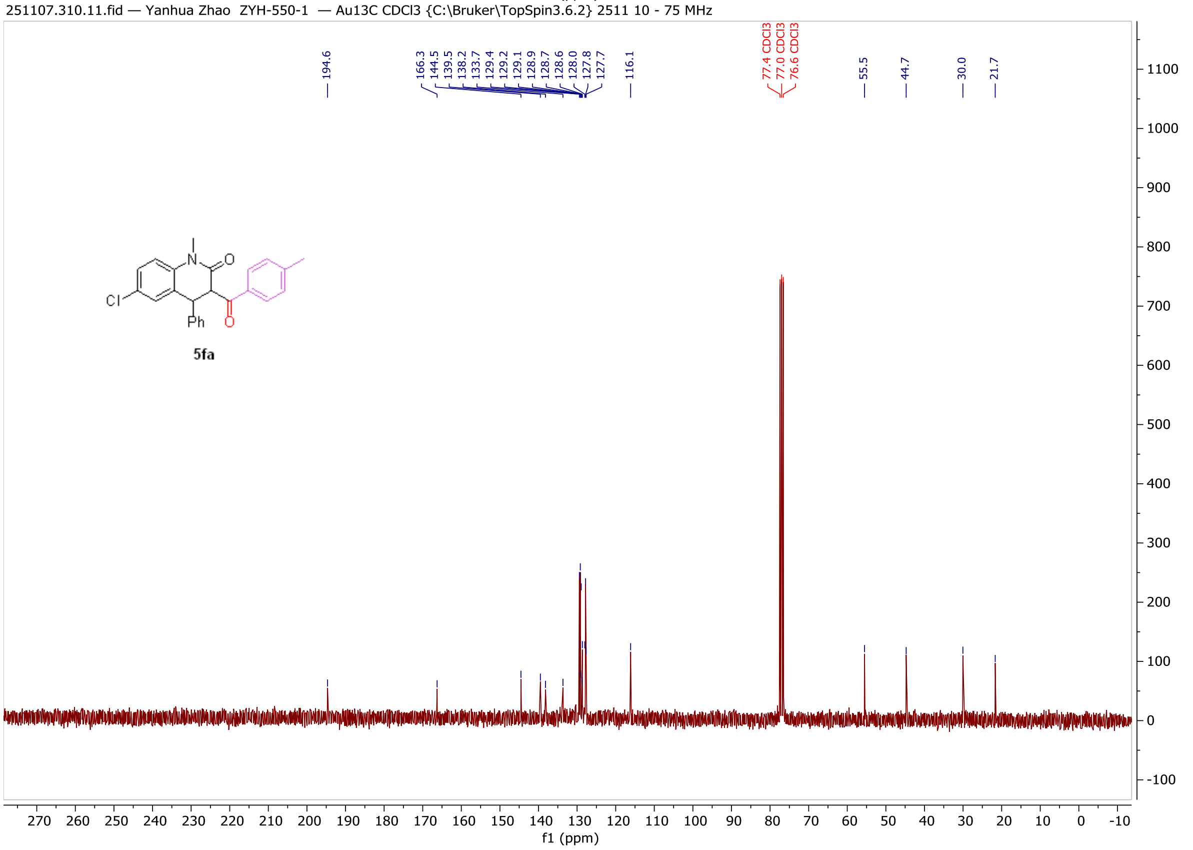

251107.311.10.fid — Yanhua Zhao ZYH-550-2 — Au1H CDCl3 {C:\Bruker\TopSpin3.6.2} 2511 11 - 300 MHz

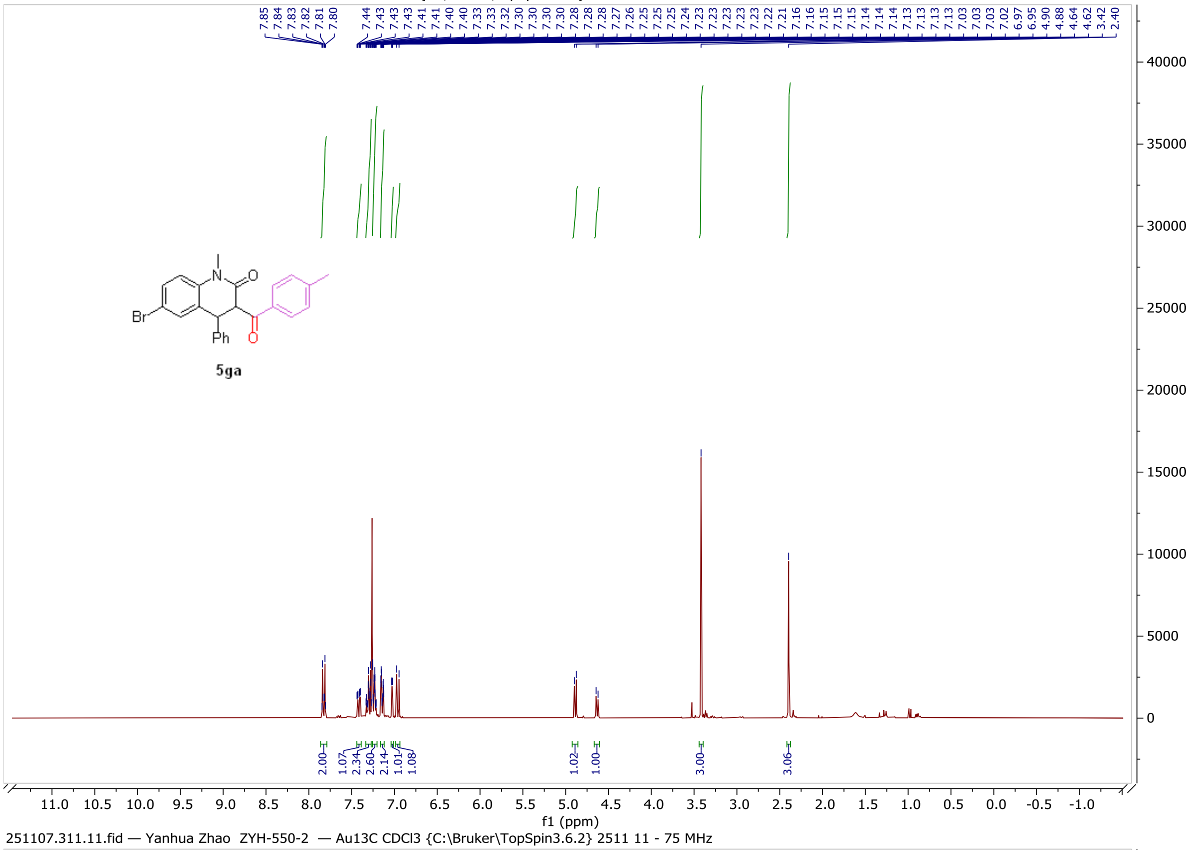

251107.311.11.fid — Yanhua Zhao ZYH-550-2 — Au13C CDCl3 {C:\Bruker\TopSpin3.6.2} 2511 11 - 75 MHz

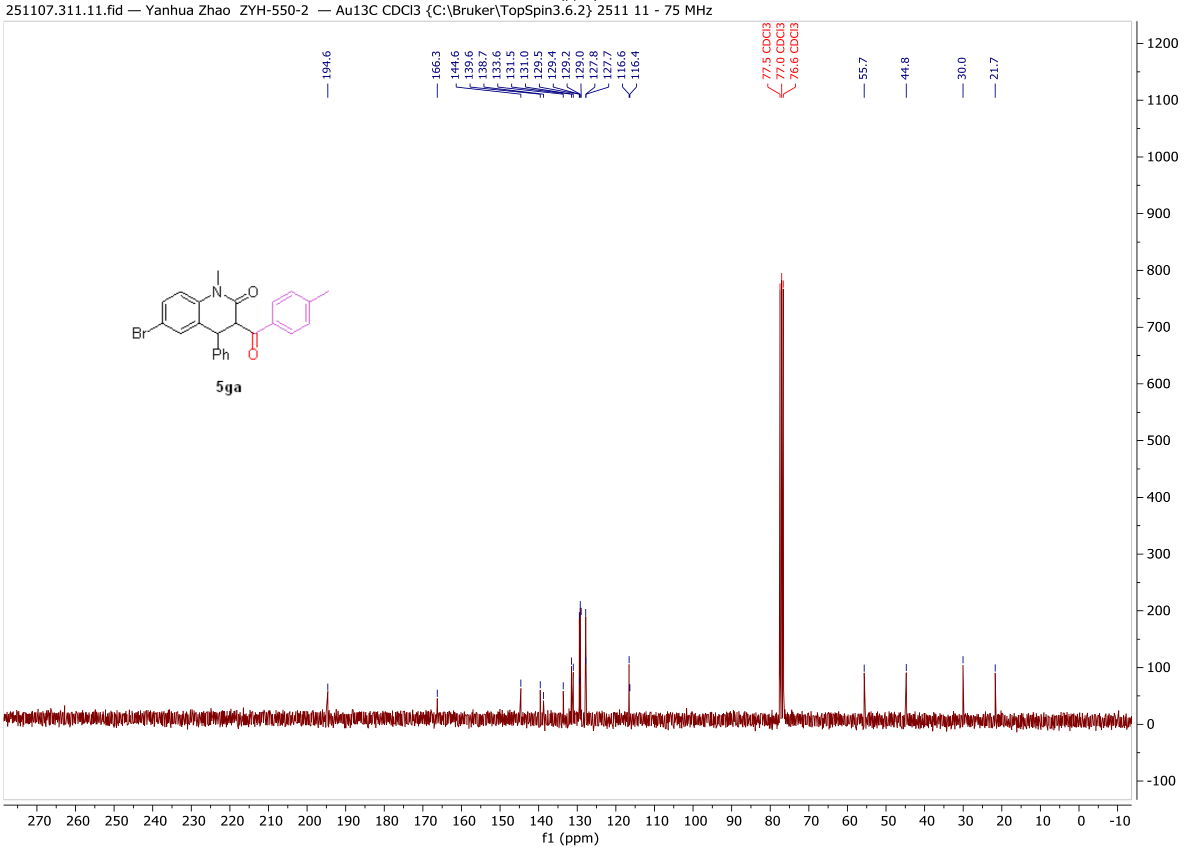

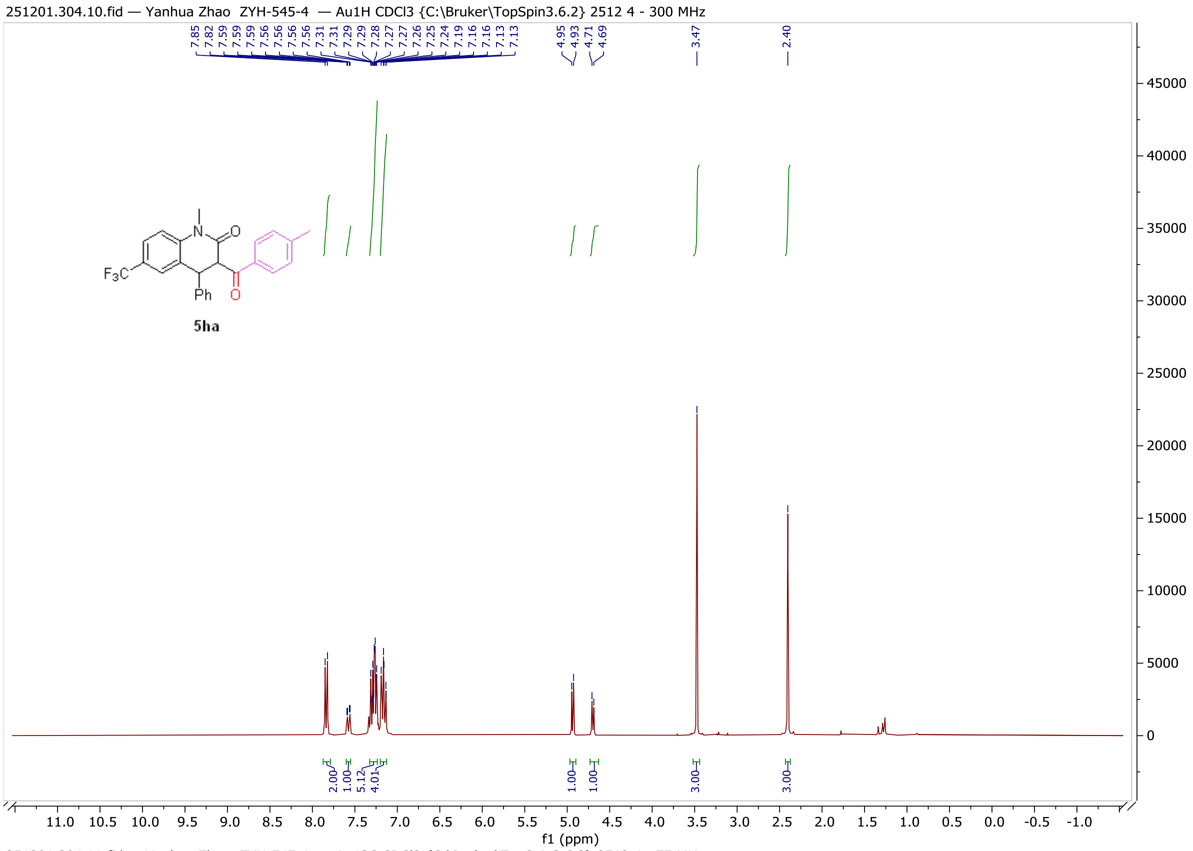
251201.304.10.fid — Yanhua Zhao ZYH-545-4 — Au1H CDCl3 {C:\Bruker\TopSpin3.6.2} 2512 4 - 300 MHz
7.85 7.82 7.59 7.59 7.56 7.56 7.56 7.56 7.31 7.31 7.29 7.28 7.27 7.27 7.26 7.25 7.24 7.19 7.16 7.16 7.13 7.13
4.95 4.93 4.71 4.69
3.47
2.40
F3C
N
O
Ph
O
5ha
2.00 1.00 5.12 4.01 1.00 1.00 3.00 3.00
f1 (ppm)

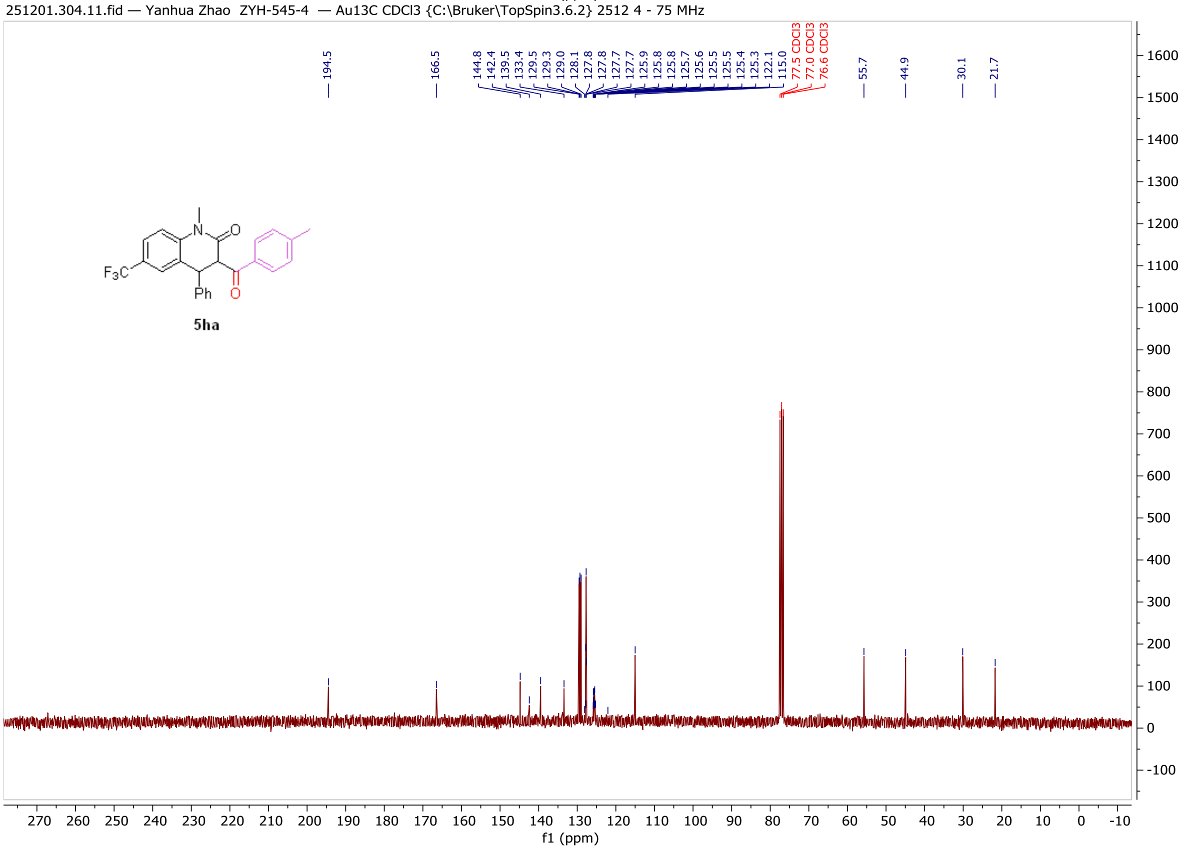
251201.304.11.fid — Yanhua Zhao ZYH-545-4 — Au13C CDCl3 {C:\Bruker\TopSpin3.6.2} 2512 4 - 75 MHz
194.5
166.5
144.8 142.4 139.5 133.4 129.5 129.3 129.0 128.1 127.8 127.8 127.7 127.7 125.9 125.8 125.7 125.6 125.5 125.5 125.4 125.3 122.1 115.0
77.5 CDCl3 77.0 CDCl3 76.6 CDCl3
55.7
44.9
30.1
21.7
F3C
N
O
Ph
O
5ha
f1 (ppm)

251201.304.12.fid — Yanhua Zhao ZYH-545-4 — Au19F CDCl3 {C:\Bruker\TopSpin3.6.2} 2512 4-282 MHz

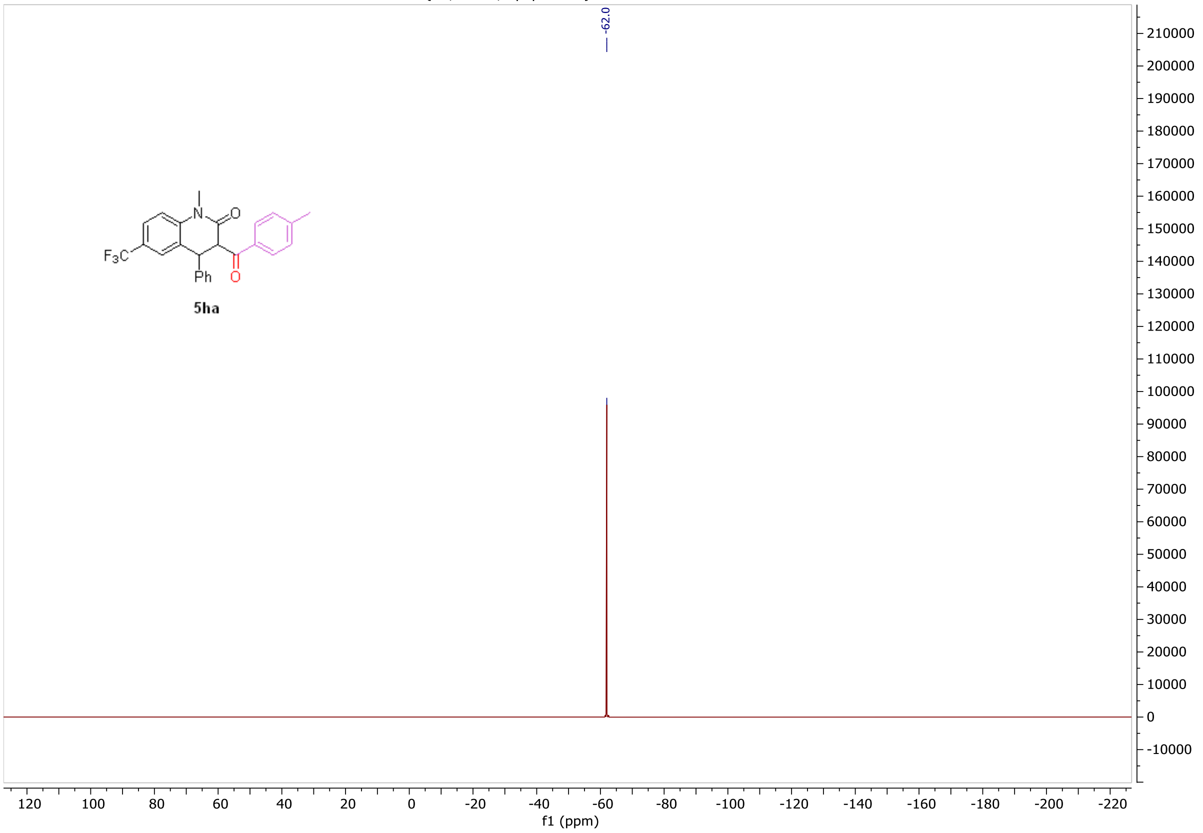

251105.f303.10.fid — Yanhua Zhao ZYH-545-9 — Au1H CDCl3 {C:\Bruker\TopSpin3.6.2} 2511 3 - 300 MHz

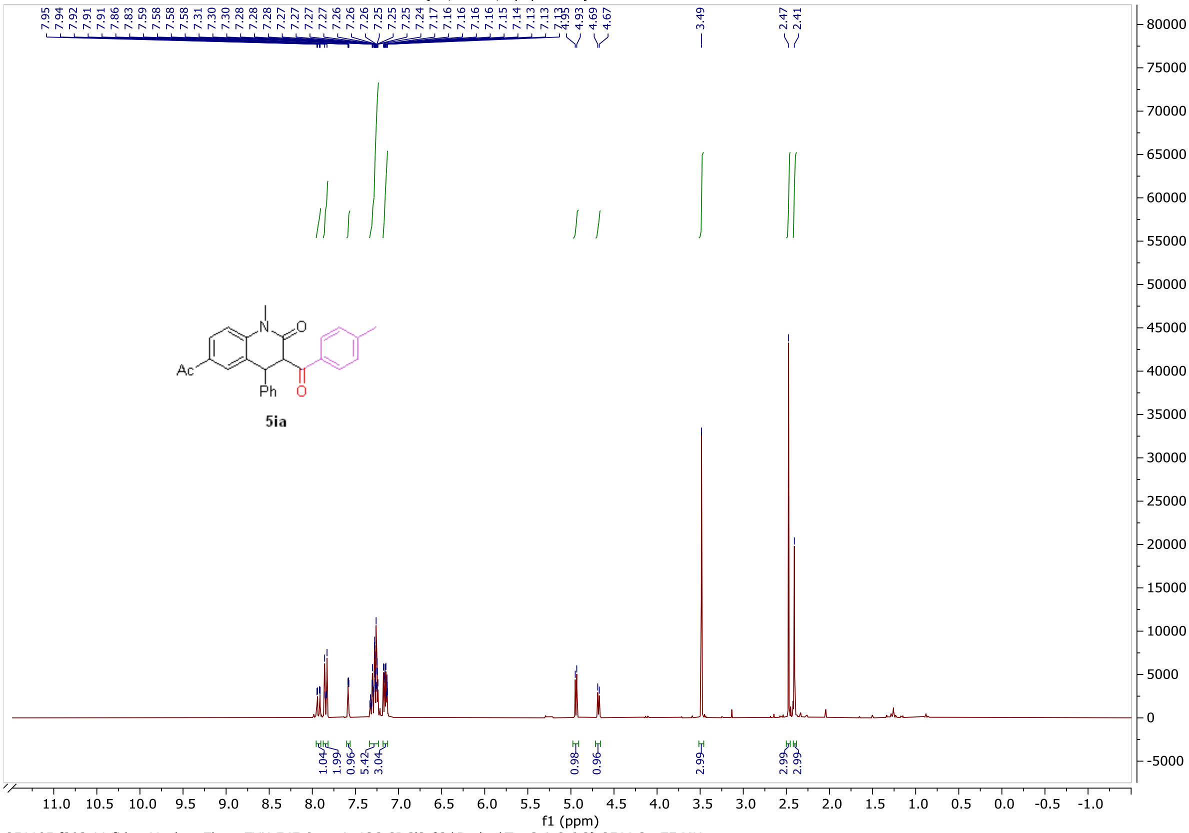

251105.f303.11.fid — Yanhua Zhao ZYH-545-9 — Au13C CDCl3 {C:\Bruker\TopSpin3.6.2} 2511 3 - 75 MHz

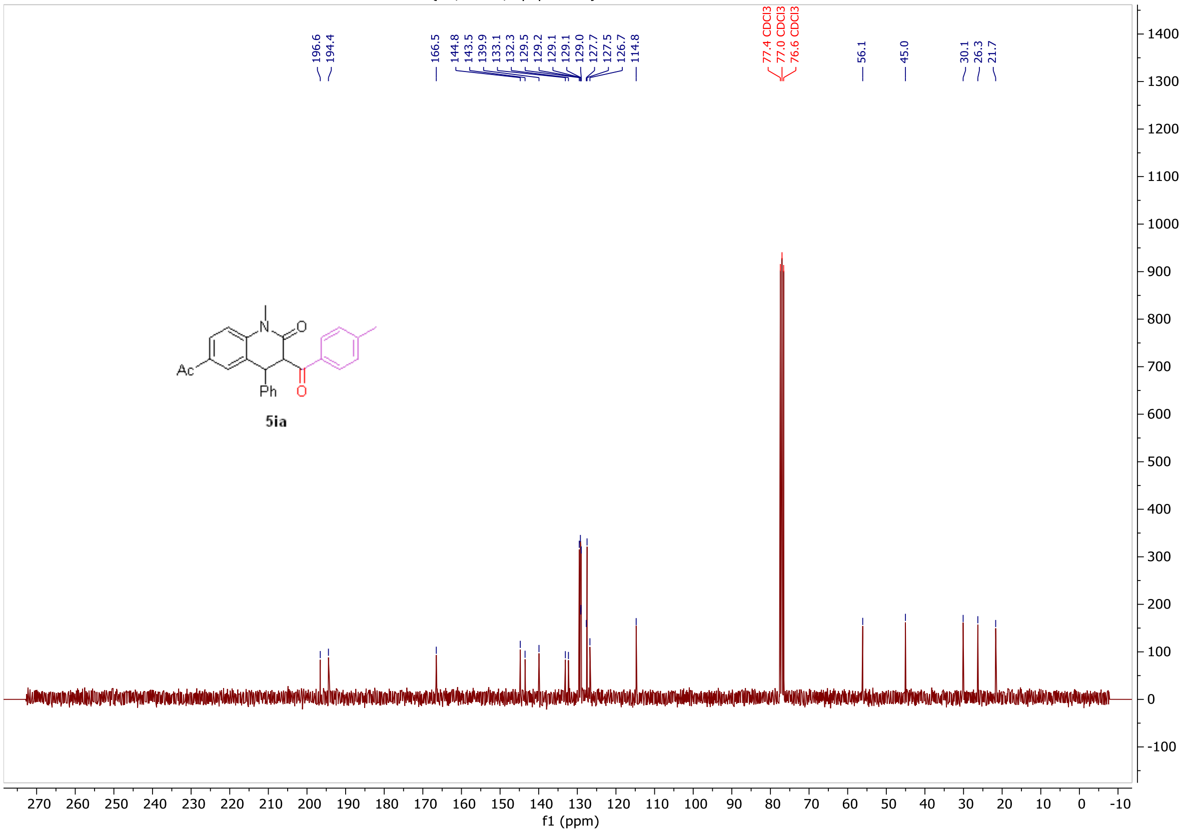

251107.312.10.fid — Yanhua Zhao ZYH-550-8 — Au1H CDCl3 {C:\Bruker\TopSpin3.6.2} 2511 12 - 300 MHz

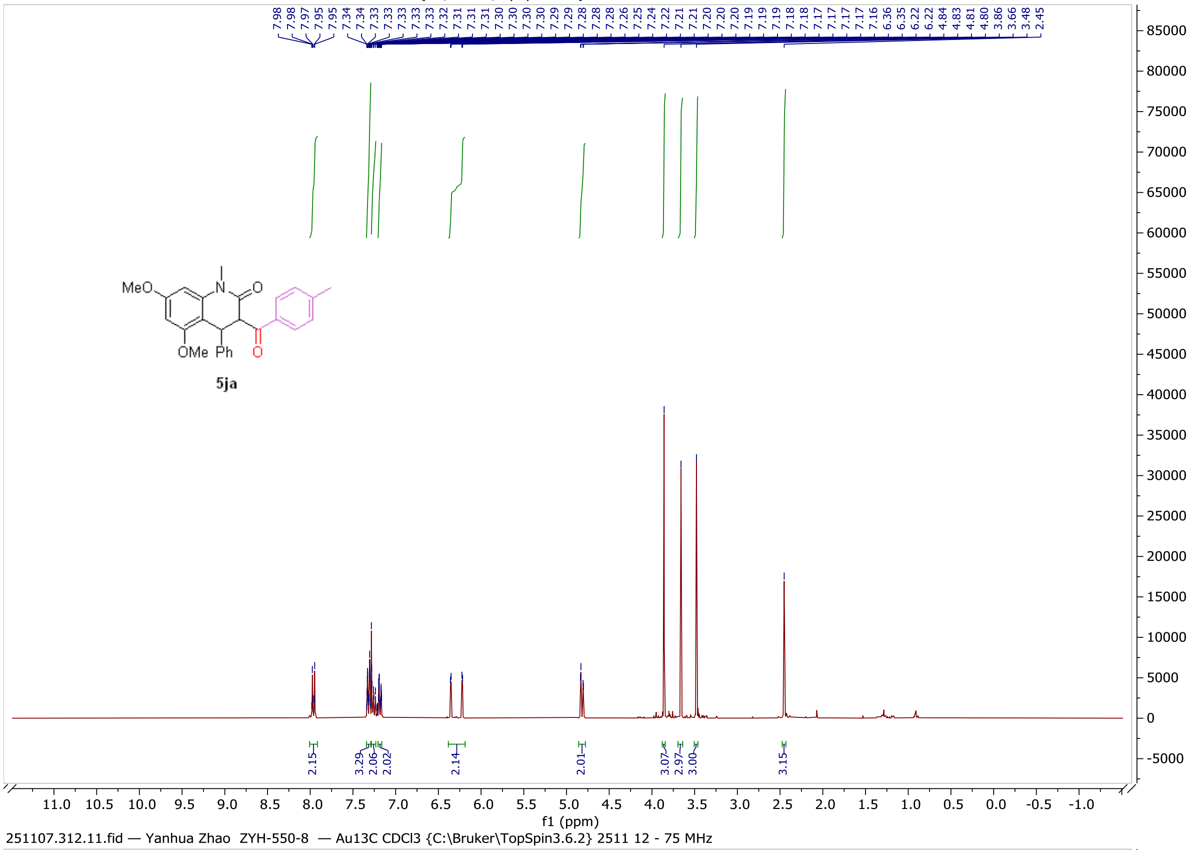

251107.312.11.fid — Yanhua Zhao ZYH-550-8 — Au13C CDCl3 {C:\Bruker\TopSpin3.6.2} 2511 12 - 75 MHz

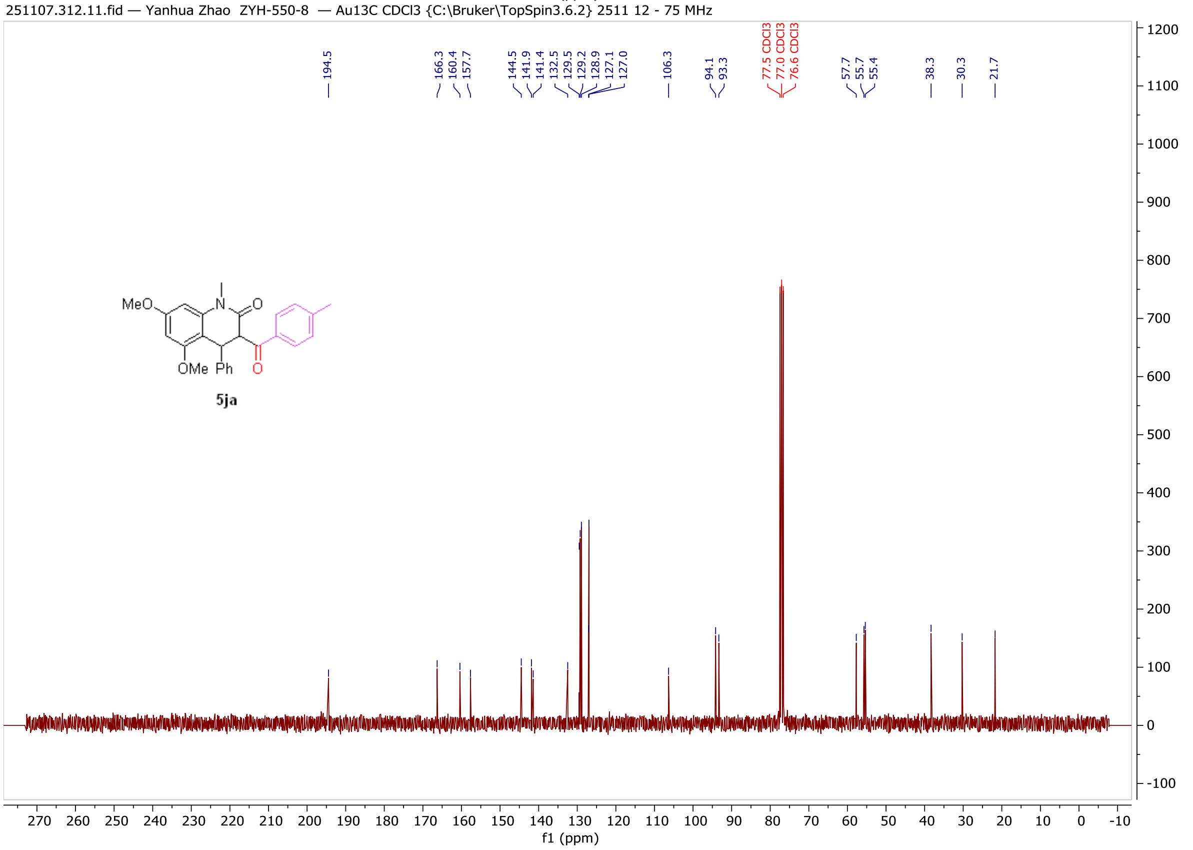

251201.305.10.fid — Yanhua Zhao ZYH-550-9 — Au1H CDCl3 {C:\Bruker\TopSpin3.6.2} 2512 5 - 300 MHz

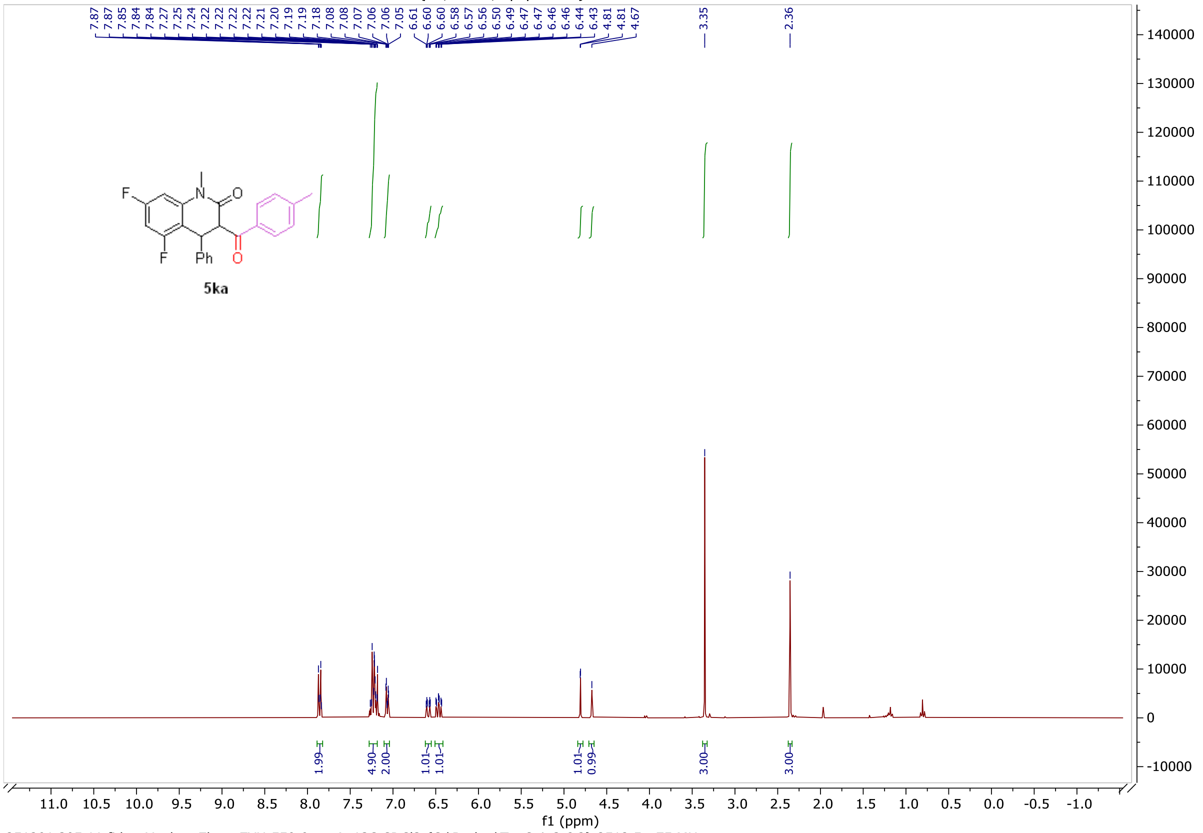

251201.305.11.fid — Yanhua Zhao ZYH-550-9 — Au13C CDCl3 {C:\Bruker\TopSpin3.6.2} 2512 5 - 75 MHz

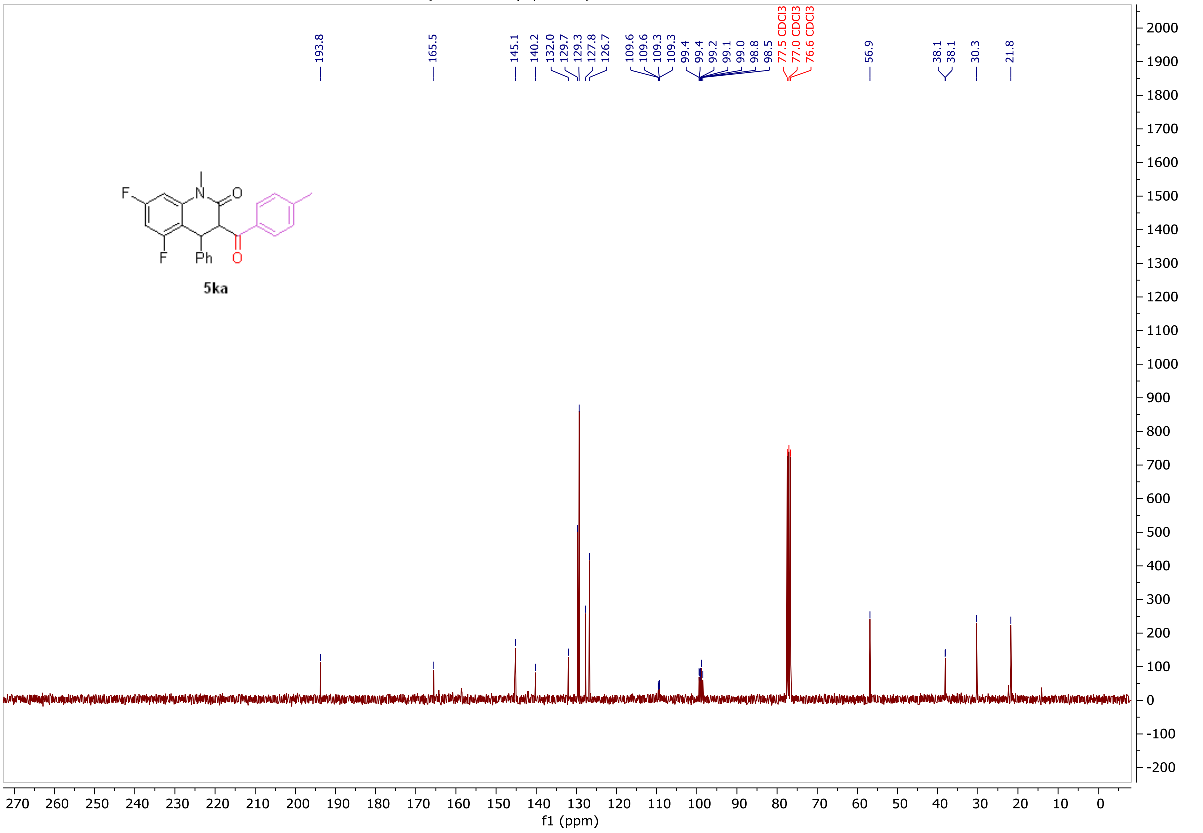

251201.305.12.fid — Yanhua Zhao ZYH-550-9 — Au19F CDCl3 {C:\Bruker\TopSpin3.6.2} 2512 5-282 MHz

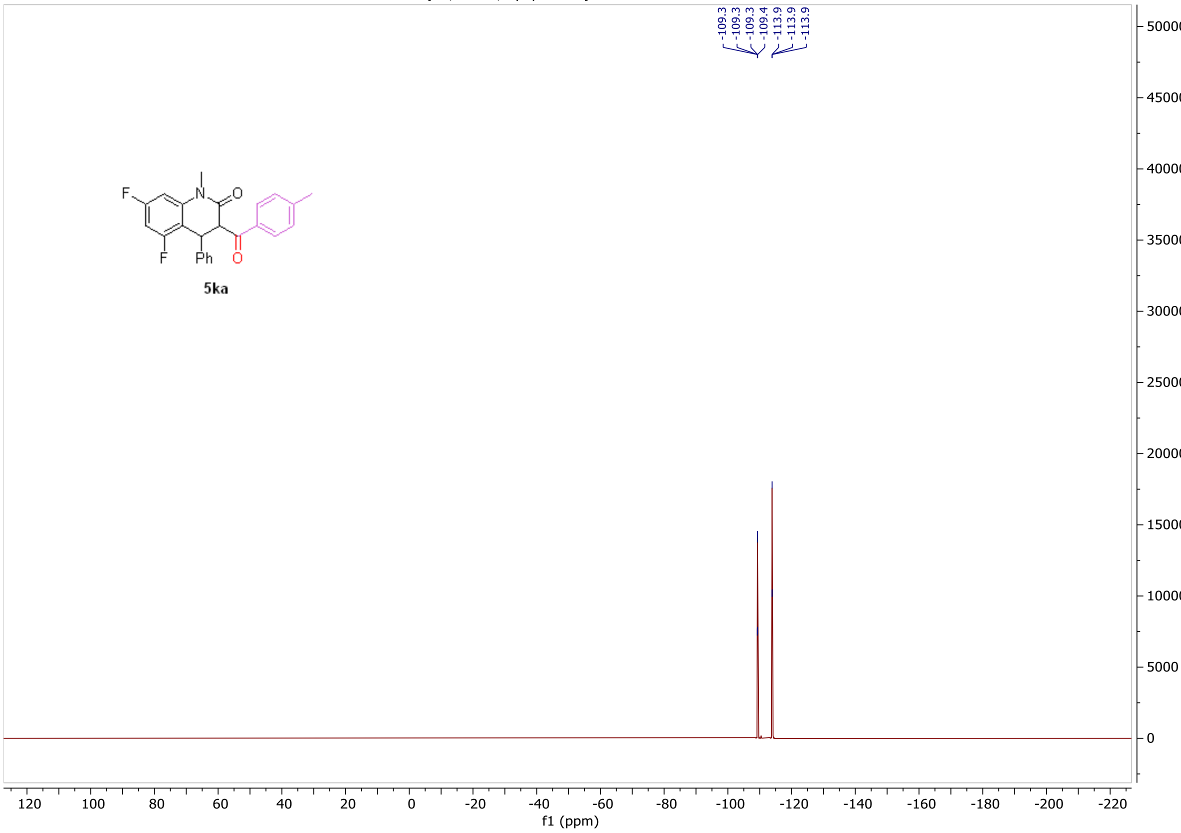

Supplement: SC-017-D5SC09434H-s001 [file SC-017-D5SC09434H-s001.pdf]
